# Comparison of fish biomass and fish carbon content associated with reef sites at the Rio Grande Valley artificial reef in the Gulf of Mexico

**DOI:** 10.1371/journal.pone.0350204

**Published:** 2026-06-04

**Authors:** Allison K. White, Md Saydur Rahman, Richard J. Kline

**Affiliations:** 1 School of Earth, Environmental, and Marine Sciences, University of Texas Rio Grande Valley, Brownsville, Texas, United States of America; 2 School of Integrated Biological and Chemical Sciences, University of Texas Rio Grande Valley, Brownsville, Texas, United States of America; MARE – Marine and Environmental Sciences Centre, PORTUGAL

## Abstract

As global fisheries stocks have decreased due to overfishing and climate change, artificial reefs have gained significant attention. In addition to providing or restoring habitat, artificial reefs may serve as carbon capture sinks and potential climate mitigation strategies. The Rio Grande Valley (RGV) Reef is an artificial reef located in the northwestern Gulf of Mexico off the coast of Texas. The RGV Reef area spans 1,650 acres and is comprised of hundreds of groupings of recycled and pre-formed materials ranging from low relief (1–2 ft) to high relief (8–30 ft) reef structures. This reef provides a variety of novel habitat stepping-stones for multiple fish species at different life stages and supports local fisheries. Since the deployment of reef structures, fish biomass and abundance are substantially higher than expected for unconsolidated soft-bottom habitats; however, quantifying fish biomass is necessary to evaluate the relative effectiveness of different structural types. For this study, a Simrad EK80 split-beam sonar was used to estimate fish biomass at the reef sites. Biomass was calculated using fish echo returns and length-weight relationships. The standing stock of fish carbon contained within the fish populations associated with the RGV Reef was assessed using literature-derived measurements. The total fish biomass within the RGV Reef area was estimated at 24.4 tons, equating to 2.81 metric tons of carbon within the fish population. This study provides important information on the factors influencing fish biomass in this reef and the amount of carbon held in fish biomass within a previously overlooked potential blue carbon ecosystem.

## 1. Introduction

As global fisheries stocks have declined due to overfishing and large-scale anthropogenic disturbances such as oil spills, habitat degradation, and climate change, artificial reefs have gained significant attention [1–3]. In addition to restoring or creating habitat for fish, artificial reefs also provide areas for recreational diving and commercial and recreational fishing [4,5]. The practice of placing structures in water to attract fish dates back to the 17^th^ century in Japan [2]. Despite early recognition that such structures attract fish, artificial habitat was not incorporated into fisheries management practices until the mid to late 1900s [2].

Artificial reefs are typically defined as intentionally placed submerged structures designed to mimic or replace natural reef habitats [6,7]. The material(s) used vary geographically and have evolved with advances in research. Common materials include concrete and stone due to their durability, availability, low cost, and nontoxicity [6,8,9]. Other artificial structures include offshore platforms, sunken vessels, and purpose-built reef modules designed to provide habitat for fish and other marine life [2,3,6,10,11]. Reef performance depends on design, materials, environmental conditions, and project objectives [6]. Artificial reefs may be particularly effective in soft-bottom environments where natural hard substrate is limited [12]. However, debate continues regarding whether artificial reefs increase fish production by promoting recruitment or attract existing fish, potentially increasing vulnerability to fishing pressure; however, the same points can be made for natural reefs [9,13,14]. Continued research is needed to assess their ecological effectiveness and optimize deployment strategies.

Carbon stored in marine ecosystems is commonly referred to as blue carbon [15–18]. Mangroves, seagrasses, and salt marshes are widely recognized as major blue carbon sinks [15,19,20], but other ecosystems have received comparatively little attention [21–23]. Artificial reefs may serve a dual purpose by providing habitat while also facilitating carbon accumulation. Hard-bodied encrusting organisms growing on reef structures sequester carbon within shells and skeletal material [24,25], while fish store carbon in their biomass through trophic interactions [26]. Marine fish contain approximately 43–45% carbon by dry body weight, a potentially important source of organic carbon in marine ecosystems [27,28]. When organisms die, organic carbon can enter the marine carbon cycle and may be sequestered through sedimentation and burial, contributing to long-term carbon storage [16,18,20,29]. Because artificial reefs are often placed in areas lacking natural reef structure, they can create new ecosystems with associated carbon storage potential as they mature and support increasingly complex biological communities. However, the contribution of fish biomass associated with artificial reefs to marine carbon storage remains largely unquantified.

Various age classes of fish exhibit differences in habitat preference [3,5,30–32]. Low-profile structures often provide refuge for juvenile fish, helping them to avoid predation [5,30,32–34]. Survival during early life stages strongly influences fish recruitment, and natural nursery habitats are increasingly threatened by anthropogenic disturbances such as dredging and trawling [32,34–37]. Artificial reefs may therefore play an important role in supporting early life stages and enhancing fisheries’ resilience. Fish at younger life stages often exhibit strong site fidelity, remaining within familiar habitats [30]. As they age and mature, they become more mobile and utilize larger, more complex habitats, while developing greater defenses against predation [32]. Habitat structure and reef age influence fish abundance and diversity, with older reef sites often supporting more complex communities [32]. Conversely, younger fish are frequently associated with newer, smaller reef structures that provide suitable conditions for growth and protection [30,32]. Understanding these relationships is important for designing artificial reefs that support multiple life stages and promote sustainable fish populations.

The western Gulf of Mexico (GoM) is a common region for artificial reef deployment due to the scarcity of natural structure in sand- and mud-dominated seafloor environments influenced by Mississippi River inputs [14,32]. Degradation of coastal ecosystems from anthropogenic activities such as trawling, dredging, and coastal development, along with tropical storm events, has further motivated the deployment of artificial reefs [5,30,32]. The GoM is also a major oil and gas production region, where offshore platforms have created additional fish habitat and opportunities to repurpose decommissioned infrastructure as artificial reefs [11,30]. The region supports substantial commercial and recreational fisheries, including reef-associated species such as red snapper (*Lutjanus campechanus*), gag (*Mycteroperca microlepis*), greater amberjack (*Seriola dumerili*), and red grouper (*Epinephelus morio*) [5,30,32,38,39]. Artificial reefs have been widely investigated for their potential to support the recovery and recruitment of these commercially important species; however, accurately quantifying fish abundance and biomass is essential for evaluating their ecological effectiveness.

Fish abundance on artificial reefs has traditionally been determined using scuba surveys, underwater cameras, or more invasive methods such as longline or trawl surveys [40,41]. However, in regions such as the western GoM, high turbidity and persistent nepheloid layers limit visibility and reduce the effectiveness of visual survey techniques [42]. These approaches may also influence fish behavior and introduce observational bias [43–45]. Hydroacoustic surveys provide a noninvasive alternative for estimating fish abundance and biomass across large spatial scales [31,43–51]. These methods sample the entire water column and provide continuous spatial data on fish distribution through ensonified fish counts and measurements [43–45]. Ensonification is the process of using a sonar transducer to emit sound waves into a volume of water, where they reflect off objects such as structures and fish, then return to the transducer for detection and analysis. Despite these advantages, hydroacoustic surveys may underestimate biomass near the seafloor due to the acoustic dead zone, where fish cannot be reliably distinguished from structures or substrate [43,52]. Fish orientation relative to the sonar transducer can also influence length and biomass estimates [52,53], making multiple transects necessary to obtain representative estimates.

Artificial reefs contribute to ecological resilience and regional economies by supporting fisheries and marine biodiversity. However, their potential contribution to carbon storage remains poorly quantified, particularly through fish biomass associated with artificial reef ecosystems. Understanding this component is important because reef-associated fish may represent a previously overlooked pathway for carbon storage within marine food webs. The objectives of this study were therefore to (1) estimate total fish biomass across an artificial reef area using hydroacoustic surveys, (2) compare biomass among reef sites with different structural materials and locations, and (3) estimate the amount of carbon contained within fish biomass associated with the reef system. We hypothesized that reef sites with greater vertical relief and larger footprint area would support higher fish biomass and therefore contain greater associated carbon stocks. By quantifying fish biomass and associated carbon stocks, this study provides new insights into the ecological roles of artificial reefs and evaluates their potential contribution to an underexplored component of blue carbon ecosystems.

## 2. Methodology

### 2.1. Study area

**Fig 1:**
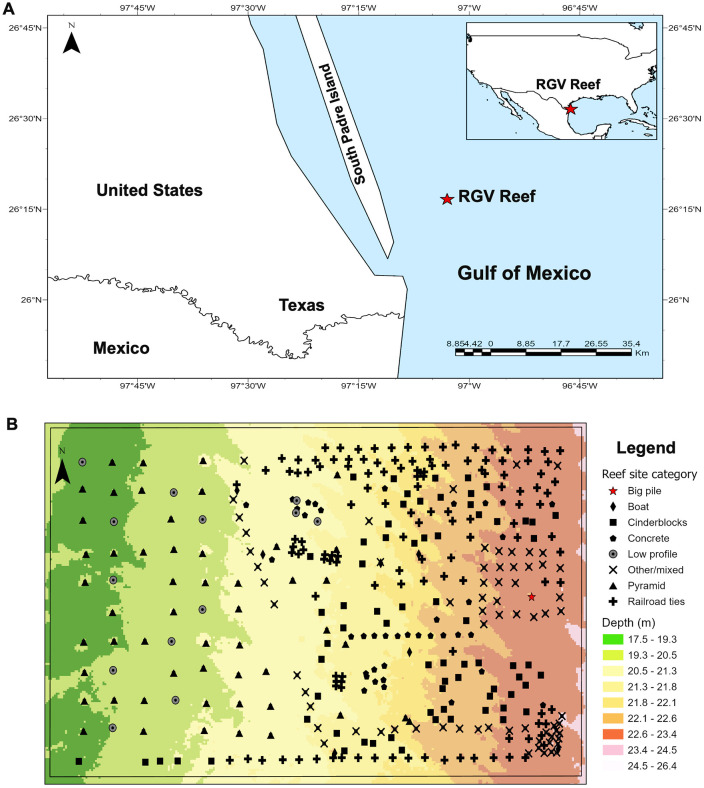
Study site in the northwestern Gulf of Mexico. **(A)** Location of Rio Grande Valley (RGV) Reef. **(B)** Map of RGV Reef structure.

**Fig 2:**
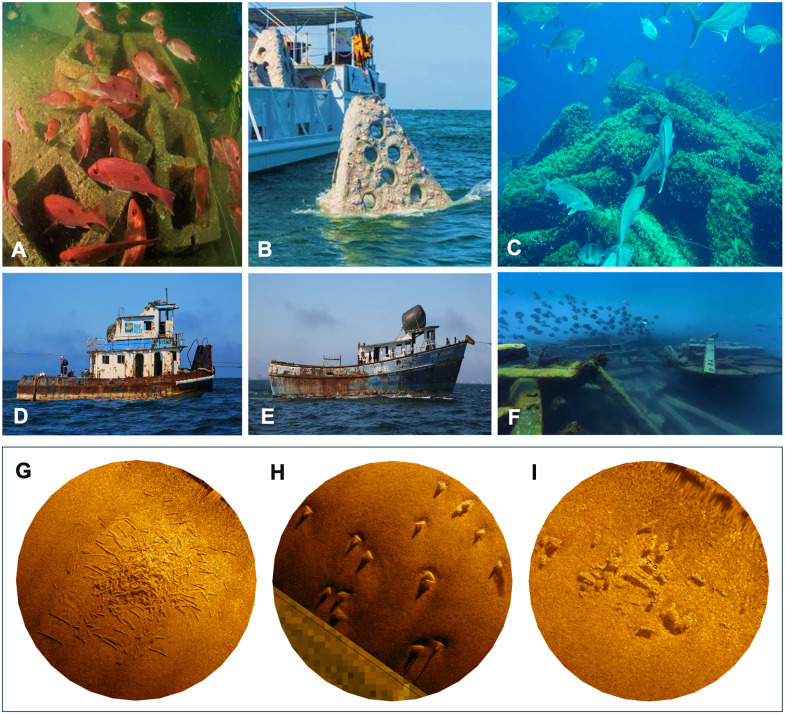
Rio Grande Valley Reef structure configurations. **(A)** Different groupings of cinderblock pallets, (B) prefabricated pyramids, (C) piles of concrete railroad ties in a range of sizes, (D and E) boats, and (F) mixed configurations of railroad ties, spools, and other opportunistically sourced materials. **(G)** High resolution side scan sonar images of a 250-ton railroad tie pile, (H) a 16-pyramid site, and (I) a mixed materials site with cinderblocks, culverts, highway dividers, and broken concrete from SonarWiz7.

This study was conducted at the Rio Grande Valley (RGV) artificial reef in the northern Gulf of Mexico (GoM) 10 nautical miles (18.5 km) off the coast of South Padre Island, Texas (Fig 1A). The RGV Reef area spans approximately 1,650 acres (6.7 km^2^) and was comprised of 477 reef sites at the time of data collection (Fig 1B). Reef structure configurations included railroad tie piles, boats, cinderblock pallets, prefabricated pyramid structures, and combinations of multiple concrete materials (Fig 2A-F, S1 Table). Reef sites varied in vertical relief, number of materials, location within the reef area, type of structures, “footprint” area, and age. Vertical relief ranged from low (0.3–1 m) to high (3–10 m), and footprint area ranged from small (2.87 m^2^) to large (7,014.54 m^2^). Reef structure types were identified using deployment records and verified using side-scan sonar imagery collected after deployment and during the study. Sites, defined as clusters of structures within 10–50 m of each other, were separated by at least 100 m. The RGV Reef was designed to provide habitat for multiple fish species during different life stages to support local fisheries, with a special interest in juvenile red snapper (*Lutjanus campechanus*). Before reef deployment, hydroacoustic surveys conducted in the area indicated minimal fish presence, consistent with low-relief, barren, unconsolidated sediment environments. Since the deployment of structure in 2016, anecdotal reports and previous research suggest increases in fish abundance and recruitment, particularly of commercially harvested snapper (*Lutjanus sp*.) [5,54]; however, quantifying fish biomass is necessary to evaluate the effectiveness of different structure types in recruiting fish.

### 2.2. Hydroacoustic data collection and processing

Acoustic surveys were conducted during the daytime in summer and early fall of 2023 and 2024. Because the study involved non-invasive acoustic observations and did not involve the capture, handling, or disturbance of organisms, no specific research permits or ethical approval were required. Sonar data were collected using a multiple-frequency echosounder (Simrad EK80 split-beam) operated at 38kHz and paired with a 200kHz single-beam sonar. The EK80 was configured to ping at 500W at 38kHz and 200W at 200kHz, with a pulse duration of 0.256ms. The device was deployed using a side-mount pole system specifically designed to mount the acoustic device to the side of the research vessel minimizing tilt and vibration while in motion. Positioning was obtained by a TriNav GPS (Digital Yacht GPS160 TriNav Sensor w/NMEA 0183, Boston, MA, USA). Calibration was conducted *in situ* following the standard sphere calibration method (1.50” tungsten sphere; Demer et al., [55]). Acoustic data were recorded using Simrad EK80 software, interfaced with a motion reference sensor (SMC IMU-108–30) to track motion and position. Sound-speed profiles were calculated using a SonTek CTD several times during the sampling trip [45]. Surveys consisted of straight-line passes over or near the structure within the reef area. The number and orientation of passes varied depending on the structure footprint and field conditions (e.g., currents, wind, drag). For sites with large footprints, such as boats or the Big Pile, multiple non-overlapping passes were conducted on the same day from different sides of the structure to sample the full site area adequately. In these cases, biomass estimates from three non-overlapping passes were combined to yield site-level biomass estimates. For smaller structures or when passes overlapped spatially, individual passes were treated separately, and a single representative pass was selected for each site to avoid double-counting. Some sites were surveyed on multiple days; therefore, to maintain independence among observations and minimize repeated sampling of the same fish populations, only one sampling day per site was retained for analysis. Specifically, the day with the highest estimated biomass for each site was selected to represent that site.

### 2.3. Data analysis

Simrad split-beam acoustic data (.raw files) were visualized and processed using Echoview 13.1 (Echoview Software Pty. Ltd., Tasmania, Australia). Raw data were cleaned by applying calibration settings and filters to minimize background and impulse noise caused by interference from other sonars in use at the time [43,45]. Additionally, echograms were visually reviewed to eliminate further unwanted noise caused by GPS dropouts, bubbles, and/or wave action [45]. A GPS filter was also applied to clean position data and retain matching fixes from the most reliable GPS source (GNGGA). Ping times and ping geometry were matched between the two frequencies (38kHz and 200kHz) to improve data quality [43]. The best bottom-candidate detection algorithm was used to determine the bottom line, and an offset line one meter above the bottom line was established to account for the acoustic dead zone [52]. Data below this offset line was not used in the analysis. A surface line was established at 4 meters above the water surface, and data above this line were not used in the analysis.

Fish targets were distinguished from non-biological echoes based on their acoustic characteristics and movement patterns within the echogram. Fish targets typically produced discrete point targets or coherent schools with consistent target strength values, distinctive visual characteristics, and predictable movement patterns. In contrast, reef structures produced continuous, stationary high-intensity backscatter adjacent to the seafloor [56–58]. Fish schools were identified using the specific fish school detection algorithm in Echoview with threshold values for expected backscatter and school size. After schools were delineated, the regions were masked from the 38 kHz echogram before individual target detection, thereby excluding fish within schools from individual fish detection and preventing potential double counting [56,43]. Individual fish targets were then identified using the split-beam single-target detection algorithm in Echoview, which isolates individual echoes that meet predefined criteria for pulse length, beam compensation, and target strength. This process generates fish tracks that represent individual fish movement within the acoustic beam. Because acoustic tracking is based on the position and characteristics of individual echoes rather than species identity, the algorithm distinguishes separate fish targets using spatial separation, echo intensity, and movement trajectories between successive pings, allowing multiple fish to be tracked even when species identity cannot be determined. To minimize repeated counting of the same individual, fish tracks were counted once per pass through the acoustic beam. Survey transects were conducted as discrete passes over reef sites rather than continuous circular sampling; therefore, the probability of detecting the same individual fish multiple times within a single transect was reduced. Additionally, for sites where multiple overlapping passes were conducted on a single sampling day, only a single representative pass (the one with the highest biomass estimate) was retained to avoid double counting. For larger footprint sites sampled using multiple non-overlapping passes, biomass estimates were combined to represent the full site. Detected targets were further evaluated by visual inspection of echograms to ensure that they represented coherent biological targets rather than noise, interference, or structure-associated artifacts. Direct visual ground-truthing was not conducted during this study; therefore, species identification was not attempted. Instead, acoustic targets were interpreted as fish based on established acoustic characteristics and movement behavior commonly used in fisheries hydroacoustic studies [43,45].

### 2.4. Reef site analysis

To assess the size of each reef site, the area of material coverage was measured using high-resolution side-scan sonar data collected with a vessel-mounted Bathyswath sonar (Edgetech 6205s, West Wareham, MA, USA). Individual sonar survey tracks were reviewed and processed using SonarWiz 7 (Chesapeake Technology, Inc., Los Altos, CA, United States) to isolate areas where reef structures were clearly visible. For each reef site, features were delineated within SonarWiz to represent the extent of the structure (Fig 2G-I). These features were exported from SonarWiz and imported into Google Earth Pro (Version 7.3.6.10201; Google Inc.; Mountain View, CA, United States), where they were georeferenced to a base map. The area of each reef site was calculated using the polygon measurement tool in Google Earth. Final site features were then imported into ArcGIS Pro (Version 3.4; Esri, Redlands, CA, USA) for further spatial analysis.

A 1 m resolution XYZ bathymetric map of the RGV Reef was imported into ArcGIS Pro. The XYZ text file was then converted to a point feature class using the “XY Table to Point” tool, with depth designated as the Z field. These points were interpolated using the Inverse Distance Weighted (IDW) geoprocessing tool to generate a continuous raster surface representing seafloor depth. Artificial reef structure polygons imported from Google Earth as a KMZ file were converted to a feature class, and attributes such as site area, structure type, and relief category were assigned using a table join. Fish data were imported as a CSV file containing latitude, longitude, fish counts, and biomass. Fish observations included both structure-associated (from the maximum day per site) and non-structure-associated data (compiled across all days). Structure-associated and non-structure-associated fish observations were summarized using different temporal approaches due to differences in sampling design. Structure-associated fish were analyzed at the site level, and because many structures were surveyed on multiple days, only the day with the highest biomass estimate was retained for each site to avoid repeated sampling of the same fish populations. In contrast, non-structure-associated fish were recorded opportunistically during transit between reef structures and were not repeatedly sampled at fixed locations. Therefore, these observations were compiled across sampling sites. Because these transects covered different spatial areas and did not overlap, the likelihood of recounting the same individuals was considered low.

Fish points were generated from the tabular data using the “XY Table to Point” tool. All fish observations were merged into a single layer and filtered to include only those located within the reef boundary. All spatial layers, including bathymetry, structure polygons, and fish data, were projected in State Plane NAD83 Texas South Central to ensure spatial alignment. Depth values at each fish observation point were extracted from the IDW raster using the “Extract Multi Values to Point” tool. Fish were then clustered using the Density-Based Spatial Clustering Applications with Noise (DBSCAN) algorithm with a 10-meter search radius and a minimum of 2 points per cluster. Clustered fish points were spatially joined to their cluster ID using a one-to-one intersection join. Summary statistics were calculated per cluster, including total biomass, fish count, and average depth.

To analyze spatial relationships with reef structures, centroids were calculated for each fish group using the Mean Center tool. In parallel, centroid points were created for each reef structure polygon using the “Feature to Point” tool with the “Inside” option enabled. The Near tool was used to calculate the distance from each fish group centroid to the nearest reef structure and to the reef boundary. The same procedure was applied to fish that were not part of any cluster (Cluster ID = −1) but were still located within the reef boundary. Attributes from the nearest structure were joined to each fish group centroid and individual fish using the “Join Field” tool, with the NEAR_FID field serving as the key. All outputs were exported to Excel and compiled into a single dataset for subsequent statistical analysis. A detailed ArcGIS workflow, including all geoprocessing steps and parameters, is provided in the Supporting Information (S1 File).

### 2.5. Fish weight and carbon evaluation

Because the hydroacoustic surveys used for this study did not allow for direct identification of individual fish species, species-specific length-weight relationships could not be applied to each detected target. Instead, generalized relationships derived from representative GoM fish species were used to estimate fish length and weight from target strength values [59]. These equations provide reasonable approximations of biomass when species composition is mixed or uncertain, which is common in hydroacoustic surveys of reef-associated fish communities. Although this approach may introduce some uncertainty due to interspecific differences in body morphology and acoustic reflectivity, it provides a practical method for estimating biomass at the community level and has been widely applied in fisheries acoustic studies [59–61]

The length of individual fish tracks was estimated from the mean target strength (TS) using the equation from Boswell & Wilson [59]:


$$\begin{array}{c} {\text{Target Strength}}_{\text{All}}\;=\text{ 20 }*\text{ log10}({\text{L}}_{\text{cm}})\;-\text{ 70}.\text{9} \end{array}$$

This equation was selected because it was derived from pooled data from common GoM fish species and provides a generalized predictive relationship between target strength and fish length for mixed reef fish assemblages.

The weight of an individual fish was estimated using the length following the equation of Bollinger & Kline [61].


$$\begin{array}{c} \text{Weight }=\;\text{0}.\text{0232 }*\;L^{\text{2}.\text{9088}} \end{array}$$

This equation was used because it represents average length-weight relationships for common GoM fish species and is appropriate for estimating the biomass of mixed reef fish assemblages in the region. The weight of each fish was converted to dry weight using the average fish moisture of 73.6% and then converted to carbon percent using the average whole body percent carbon of wild-caught marine fish (43.7%) from Czmanski et al. [27]. The number of fish within a fish school was estimated using the density number and corrected area outputs from Echoview. The density number is an estimate of fish per square nautical mile based upon the expected target strength of an individual fish from a specific species and the expected proportion of the species relative to the other species. To avoid overestimation of fish weight, it was assumed that all large schools were sardines with an expected target strength ranging from -48dB to -61dB and an expected mean weight of 0.02 kg [62]. This assumption was made based on previous observations by SCUBA divers and prior video surveys. Based on these observations, the majority of large fish schools in the area were sardines. The expected target strength and mean weight for sardines were derived from published literature values of large datasets. The density number was then converted to square meters by dividing by 3,429,904 m^2^. The resulting density was multiplied by the corrected school area. The resulting number provided an estimate of the school’s fish population. The weight of the fish school was estimated by multiplying the number of fish in a school by the mean expected weight. The carbon content per fish school was calculated using the same methods and literature values described above for individual fish tracks. To estimate the total fish biomass of the RGV Reef, the average fish biomass of each structure site type was taken and multiplied by the number of sites of each structure site type in the reef (Table 1). Reef structure types were consolidated into 8 categories for simplicity and further statistical analysis.

**Table 1:** Number of replicates of each structure type in the Rio Grande Valley (RGV) reef.

Structure type	Number of sites
Big pile	1
Boat	7
Cinderblocks	150
Concrete	49
Low profile	20
Other/mixed	67
Pyramid	52
Railroad ties	131

Table 1. Number of reef sites within the Rio Grande Valley (RGV) Reef categorized by structure type. Sites represent clusters of reef materials located within 10–50 m of one another and separated from other sites by at least 100 m. Structure categories were consolidated for analysis based on the primary material or configuration of each reef deployment.

### 2.6. Statistical analysis

Statistical analyses were conducted using R (Version 4.5.1, “Great Square Root”; R Core Team, 2025) within the RStudio integrated development environment (Version 2024.04.1 + 739; Posit PBC, Boston, MA, USA). The dependent variable for the statistical tests was continuous fish biomass, calculated from schools and individual fish tracks. Categorical independent variables included reef site structure type and relief. The Big Pile was excluded from the structural ANOVA because it had only a single replicate at the time of data collection; however, it was included in the relief analysis. Continuous independent variables included reef site area, number of materials, reef site age in years, average depth, distance to the nearest site, and distance to the boundary. The dataset was first imported and visually inspected for data quality and distribution. The fish biomass data exhibited variance greater than the mean, indicating overdispersion and necessitating transformation.

A log_10_ (x + 1) transformation was applied to fish biomass data to normalize the data and meet model assumptions. Differences in fish biomass among structure types and relief categories were then evaluated using one-way analysis of variance (ANOVA). Model assumptions were assessed by visual inspection of residual histograms, quantile–quantile plots, and residual-versus-fitted plots. The Shapiro–Wilk test was applied to test residual normality, and Bartlett’s and Levene’s tests were used to evaluate homogeneity of variance. When overall ANOVA results were significant (p < 0.05), post-hoc comparisons were performed using Tukey’s test to identify differences among structure types and relief categories.

To test for multicollinearity, relationships among categorical predictors (e.g., structure type and relief category) were assessed using Pearson’s Chi-squared test, and among numeric predictors using variance inflation factors (VIFs). A Principal Components Analysis (PCA) was conducted to investigate further multicollinearity and the relationship between relief category and structure type. Before analysis, categorical variables such as relief category and structure type were encoded as binary presence-absence matrixes, while continuous predictors, including average depth, distance to the reef boundary, distance to the nearest site, structure area, and site age, were standardized using z-scores to ensure equal weighting.

GAMs were fitted using the *gam()* function from the *mgcv* package with a Gaussian error structure and REML (Restricted Maximum Likelihood) smoothing parameter estimation. Smooth terms were applied to continuous predictors with a default basis dimension of k = 20. Model diagnostics were conducted using the *gam.check()* function to assess residuals, basis dimension adequacy, and convergence behavior. To identify the most parsimonious models, a model selection approach was used via the *dredge()* function from the *MuMIn* package, which evaluated all possible combinations of predictors. To assess spatial associations and content dependency, separate dredge analyses were conducted for: (i) all fish observations, (ii) fish located within 20 meters of a structure, and (iii) fish located more than 20 meters from a structure. Final models were compared based on AIC values, adjusted R^2^, deviance explained, and ecological interpretability. Visualization of model predictions and response curves was performed using the *ggplot2* and *gratia* packages. Smooth response curves were generated for continuous predictors and predicted values across factor levels, with 95% confidence intervals. Comparative visualizations were constructed to highlight differences in predicted fish weight across relief categories and distances to structure. Significance was determined at p < 0.05 and means reported are ± one standard error. The full R script used to for statistical analyses, modelling, and figure generation is provided in the Supporting Information (S2 File).

### 2.7. Sensitivity analysis

A sensitivity analysis was conducted to evaluate the influence of key assumptions on biomass estimates. Two sources of uncertainty were considered: (1) the assumed mean individual fish weight used for fish schools and (2) the length-weight relationship applied to individual fish tracks. For fish schools, the mean individual weight (0.02 kg) was varied by ±5%, and biomass was recalculated to assess the influence of this parameter on fish biomass. For fish tracks, sensitivity to length-weight parameterization was evaluated using alternative species-specific relationships representing plausible upper and lower bounds of growth relationships within the study region. Red snapper (W = 0.0135L³·⁰⁵) and grey triggerfish (W = 0.0361L²·⁷⁸) were selected based on their contrasting allometric scaling and common occurrence within the RGV Reef area. Biomass estimates were recalculated using these upper and lower bounds to assess sensitivity to these parameters.

## 3. Results

### 3.1. Fish measured from split beam data

Split-beam data (38kHz) was collected from 146.61 km of sonar transects recorded over the RGV Reef covering ~ 60% of the reef area. Regions of transects were isolated where structure was directly passed over and separated from areas of transit between structures. For fish associated with reef structures (≤ 20 m), a total of 1,839 individual fish tracks and 121 fish schools were detected using maximum counts per site. Calculated fish lengths ranged from 13 to 368 cm, with an average length of 51.99 ± 0.51 cm. A total of 1,037 individual fish tracks and 178 fish schools were detected >20 m from structure sites. Calculated lengths for fish between structure sites ranged from 12 to 262 cm, with an average length of 51.33 ± 0.76 cm.

### 3.2. Reef structure comparison

**Fig 3:**
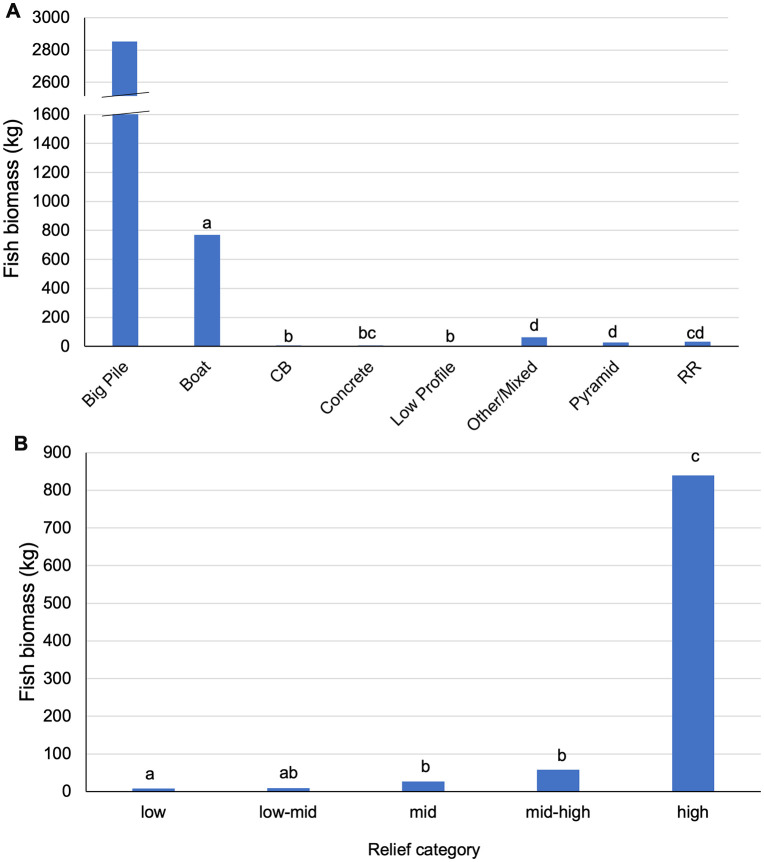
Average fish biomass. **(A)** Structure type and (B) relief category measured by split beam sonar in the RGV reef.

As predicted, average fish biomass differed among structure types (Fig 3A and S3 Table). The highest biomass was associated with the Big Pile (3435.4 kg), and the highest average fish biomass was associated with the boats (756.6 ± 475.4 kg). A one-way ANOVA detected significant differences in biomass among structure types (p < 0.001, F = 13.74, df = 6). The Big Pile was excluded from the ANOVA because it was a single structure. Post hoc tests revealed that fish biomass associated with boats was significantly (p < 0.01) greater than all other categories. Concrete, cinderblock, and low-profile sites did not differ significantly from each other. Biomass associated with railroad ties was significantly different from boats, cinderblocks, and low-profile categories (p < 0.01), but did not significantly differ from concrete, pyramids, or other/mixed categories. Pyramids, railroad ties, and other/mixed categories did not differ significantly from each other.

**Fig 4:**
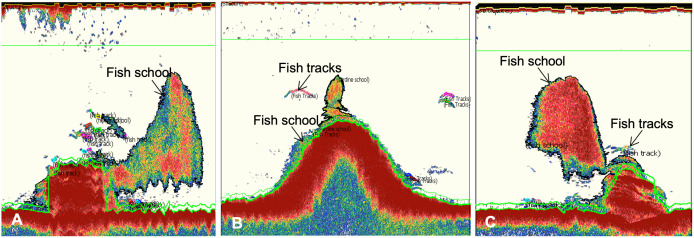
Echograms from a SIMRAD 38kHz split beam sonar visualized in Echoview software. These echograms show large aggregations of fish associated with larger reef structures like boats (A, C) and the Big Pile **(B)**, the largest pile of railroad ties on the reef.

Average fish biomass also differed by relief (Fig 3B and S3 Table). High relief structures were associated with the highest average fish biomass, and low relief structures were associated with the lowest average fish biomass (Fig 3B). The largest fish schools were often associated with high relief structures like the Big Pile and boats (Fig 4A-C). A one-way ANOVA showed significant differences in biomass among relief categories (p < 0.001, f = 21.43, df = 4). Post hoc tests revealed that fish biomass associated with high-relief structures was significantly (p < 0.01) greater than in all other categories. Mid-high structures had significantly (p < 0.01) higher biomass than low relief, but significantly (p < 0.01) lower than high relief. Low and low-mid structures supported the lowest biomass but did not differ significantly from one another. Low-mid, mid, and mid-high relief structures did not differ significantly from each other, but all were significantly (p < 0.01) lower than high relief structures.

### 3.3. Reef characteristic collinearity

A significant association was determined between structure type and relief category (Chi Squared: χ² = 1942, df = 28, p < 0.001). Variance Inflation Factors (VIF) confirmed collinearity, with values ranging from 1.81 to 21.31 (values above 10 indicate high correlation). The Principal Components Analysis (PCA) was run to determine the factor with the best explanatory value. The PCA results showed an increase in variance explained by removing structure type: 71.5% cumulative variance without structure versus 55.5% with structure. Therefore, relief category was retained in the models as the best explanatory variable, and structure type was removed.

### 3.4. Modeling the contribution of reef characteristics on fish biomass

**Fig 5:**
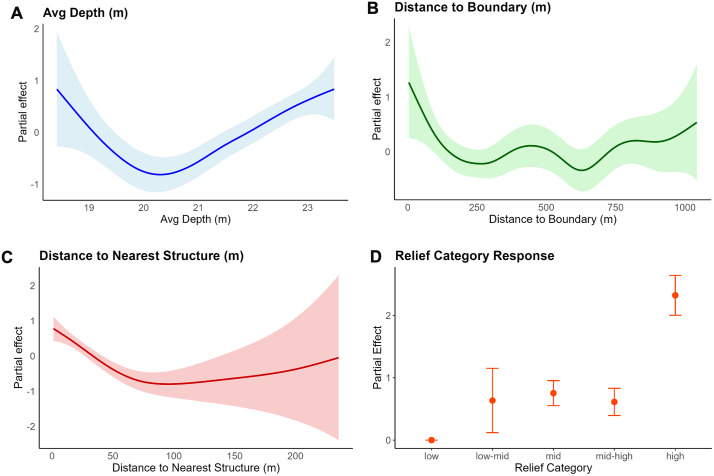
Partial effects of significant smoothed terms from the best fitting GAM selected via model dredging. The model predicts log-transformed fish biomass as a function of three smooth continuous predictors: (A) average depth (Avg_depth_m), (B) distance to the reef boundary (Dist_boundary_m), (C) distance to the nearest reef site (Dist_near_site_m), and (D) categorial relief. Each panel shows the estimated smooth effect (solid line) with 95% confidence intervals (shaded area) on the response scale. The plots highlight nonlinear relationships, with fish weight tending to decrease at intermediate depths, elevated biomass at the reef edge or greater distances from the boundary and showing a general decline with distance from reef sites.

The best-fitting generalized additive model (GAM) for all fish, henceforth referred to as the “all fish” model, accounted for 21.4% of the deviance, with an adjusted R^2^ of 0.197 (S2 Table). The significant factors identified were relief category, depth, distance to the reef boundary, and distance to the nearest reef site. Partial effect plots show fish biomass was lowest at intermediate depths, increased at the boundary or greater distances from it, and generally declined as distance from the reef site increased (Fig 5A-D and S4 Table).

**Fig 6:**
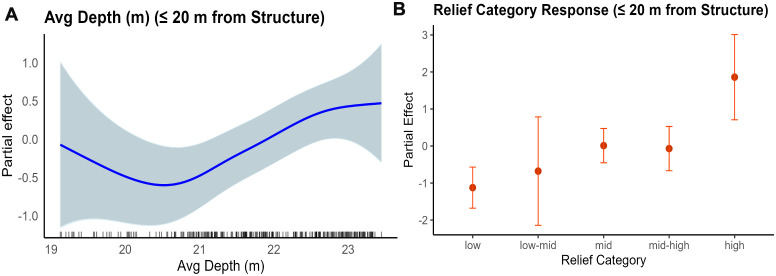
Partial effects of significant smoothed terms from the best fitting GAM for fish near structure (≤ 20 m) selected via model dredging. The model predicts log-transformed fish biomass as a function of three smoothed continuous predictors: (A) average depth (Avg_depth_m) and (B) categorical relief. Each panel shows the estimated smooth effect (solid line) with 95% confidence intervals (shaded area) on the response scale. The plots highlight nonlinear relationships, with fish biomass tending to decrease at intermediate depths and increase with increasing vertical relief.

The best-fitting GAM for fish near reef structure (≤ 20 m), henceforth referred to as the “near” model, explained 13.1% of the deviance, with an adjusted R^2^ of 0.107 (S2 Table). The significant factors were structure relief and depth. Partial effect plots show that fish biomass was lowest at intermediate depths, highest at the deepest depths, and increased as vertical relief increased (Fig 6A-B and S4 Table).

**Fig 7:**
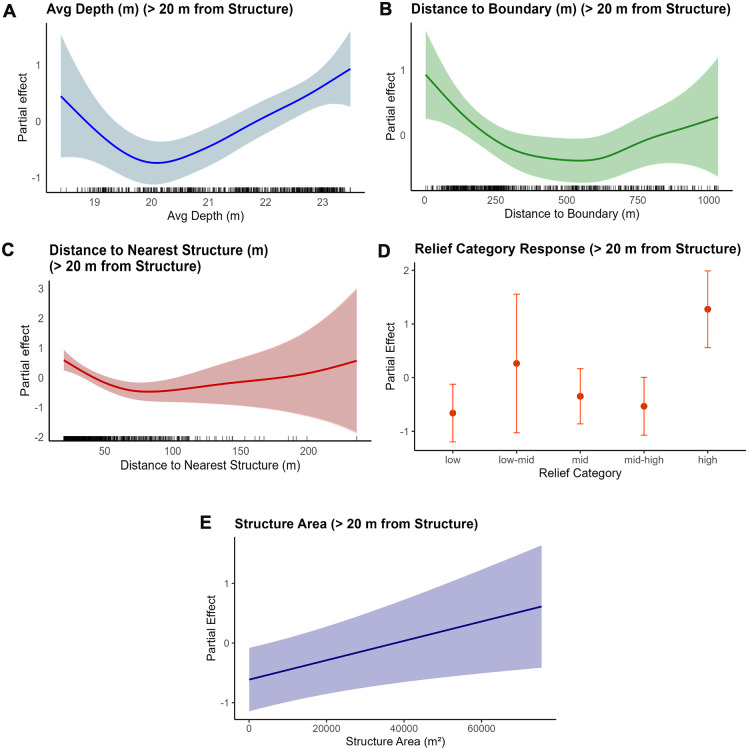
Partial effects of significant smoothed terms from the best fitting GAM for fish away from structure (>20 m) selected via model dredging. The model predicts log-transformed fish biomass as a function of three smoothed continuous predictors: (A) average depth (Avg_depth_m), (B) distance to the reef boundary (Dist_boundary_m), (C) distance to the nearest reef site (Dist_near_site_m), (D) categorical relief, and (E) structure area. Each panel shows the estimated smooth effect (solid line) with 95% confidence intervals (shaded area) on the response scale.

The best-fitting GAM for fish away from structure (> 20 m), henceforth referred to as the “away” model, explained 22.1% of the deviance, with an adjusted R^2^ of 0.2 (S2 Table). The significant factors were relief, depth, distance to the nearest site, distance to the reef boundary, and site area. Partial effect plots show fish biomass was lowest at intermediate depths, increased at the boundary or at greater distances from it, declined as distance from the reef site increased, and increased with larger site area (Fig 7A-E and S4 Table).

The relief category was significant in all three spatially grouped models, with different categories showing significance. In the “all fish” and “near” models, all relief categories showed significance in the positive direction except for low-mid. In the “away” model, only the low and high relief categories were significant. All models also showed depth as a significant factor based on p-values and effective degrees of freedom (edf values). Distance to structure was significant in the “all fish” and “away” models but not included in the “near” model. Distance to the reef boundary was not included in the “near” model and was only marginally significant in the “all fish” model but was more significant in the “away” model. Structure area was retained only in the “away” model and had a positive effect, with biomass increasing as structure area increased.

Visualizations of model predictions were created for spatial groupings based on the factors distance to structure, depth, relief category, distance to boundary, and structure area. The distance to structure prediction models showed that fish biomass was highest near reef structures and decreased as the distance from structure increased for all fish observations (a) and fish farther than 20 m from structure (b) (S1 Fig). The depth prediction models showed that fish biomass was lowest at intermediate depths for all fish (a) and fish farther than 20 m from structure (c). This trend was less pronounced but still present with fish within 20 m of structure (b) (S2 Fig). The relief prediction models showed that the relief category was positively associated with fish biomass (S3 Fig). Across all models, the highest fish biomass was associated with high relief structures. In general, as relief increased, fish biomass increased, although this was a slight variation in the pattern for fish farther than 20 m from the structure. The largest variation was seen in the low-mid category, despite having the fewest replicates (n = 7). The distance-to-boundary prediction models showed that fish biomass was slightly lower at intermediate distances from the boundary, with the greatest variation across the whole model. Distance to the boundary had no significant influence on fish within 20 m of the structure, due to the spatial constraints of the dataset (S4 Fig). The predicted effect of structure area was modeled for only fish farther than 20 m from the structure because it was the only significant factor in this spatial grouping. The model showed a positive relationship between increased fish biomass and slightly larger structure area (S5 Fig).

### 3.5. Reef-wide estimates of fish biomass and blue carbon

**Fig 8:**
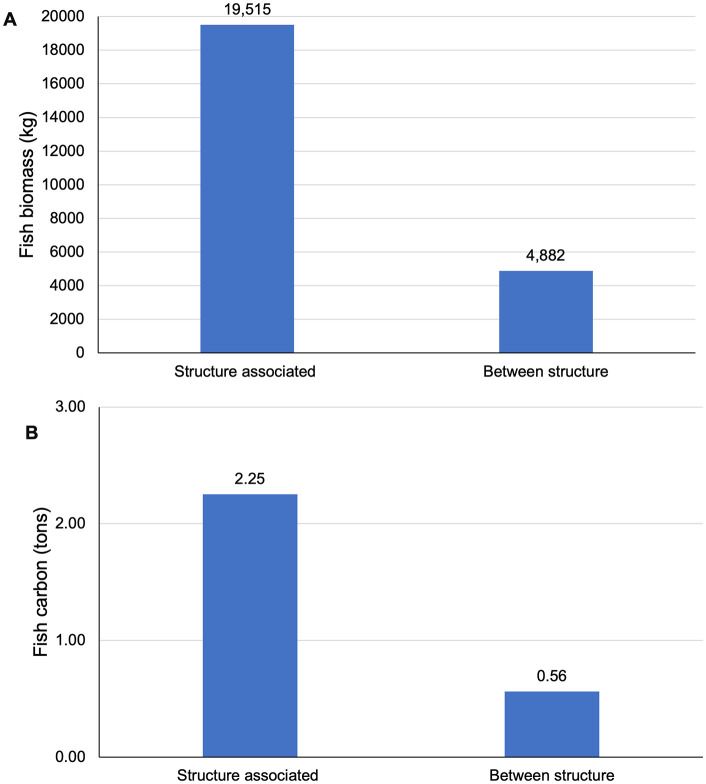
Total biomass and fish carbon. **(A)** Total extrapolated fish biomass (kg) in Rio Grande Valley (RGV) Reef area from structure associated fish (within 20 m of structure) and between structure (farther than 20 m from structure) from average biomass per structure site type and number of sites of each structure type. **(B)** Total fish blue carbon within the RGV Reef area from structure associated fish (within 20 m of structure) and fish between structures (farther than 20 m from structure).

Based on the average fish biomass per structure type and the number of sites of each type, the extrapolated total fish biomass was 19,514.9 kg from fish associated with reef sites (Fig 8A and S5 Table). The calculated biomass for fish between structure sites was 4,481.6 kg (Fig 8A and S5 Table). Combined, the total fish biomass within the RGV Reef area was estimated at around 24,396.5 kg. Based on values from Czamanski et al. [27] and the estimates of fish biomass in the present study, there were 2.25 and 0.56 metric tons of blue carbon structure-associated and fish between structures, respectively (Fig 8B and S5 Table). A total of 2.81 metric tons of blue carbon was held in the fish population within the RGV Reef area (Fig 8B and S5 Table).

## 4. Discussion

This study provides the first quantitative assessment of fish biomass associated with the RGV Reef using split-beam hydroacoustics. By converting sonar outputs (Sv and TS values) into estimates of fish length, biomass, and carbon content, this approach provides a scalable method for evaluating ecological and carbon-related functions of artificial reefs. Previous acoustic studies have used proxy values for biomass, such as backscatter, target strength, or nautical area scattering coefficient, which uses fish backscatter and area to estimate biomass density [43,45,60]. Consequently, similar studies are limited. Other studies have quantified fish biomass on artificial reefs using various methods, including trawling, scuba surveys, catch surveys, and videos [33,40,50]. Sonar surveys have some advantages over these techniques: they are not as spatially limited as scuba or video surveys, can cover much larger distances, and can penetrate the full water column. Additionally, sonar techniques are not limited to visibility constraints and present fewer avoidance behaviors in fish. Bollinger and Kline [61] found no significant difference in fish counts directly over structure conducted by side-scan sonar and scuba surveys, suggesting sonar is a reliable tool for quantifying fish abundance. Although scuba, catch, and video surveys introduce different limitations and challenges, they provide an opportunity to contextualize the present study and compare results.

### 4.1. Fish biomass measured from split-beam data

**Fig 9:**
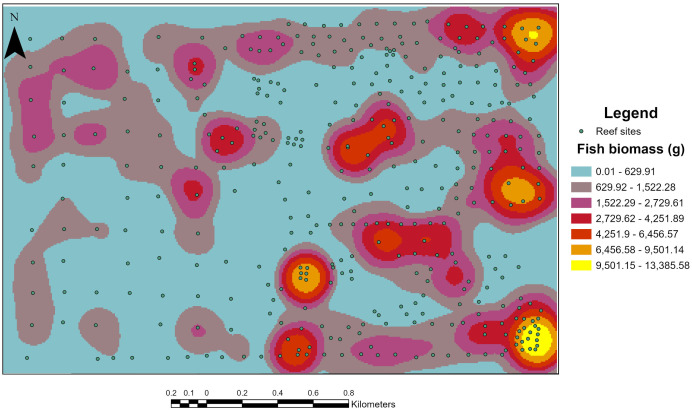
Fish populations at the Rio Grande Valley Reef are more prominent around reef structures and sparce in areas between structures. Fish hotspots are created around structure, particularly large, high relief structures like the Big Pile and boats.

Split-beam sonar transects covered a large portion of the reef area and directly visited each structure type within the reef complex. Fish biomass was strongly concentrated near reef structures, with clear spatial clustering evident in the heatmap (Fig 9). This pattern indicates that reef structures function as focal habitat features that aggregate fish biomass within the surrounding seascape. These structures provide shelter, foraging opportunities, and refuge from predators, and as a result, fish often concentrate in close proximity to these structures, producing spatially heterogeneous biomass distributions across the reef. Similarly, White et al. [45] found hotspots of fish biomass surrounding artificial reef structures, but observed more diffuse spatial distributions of fish within natural reefs. This contrast likely reflects differences in habitat configuration, as artificial reefs are often deployed in discrete patches within areas of sparce natural habitat, whereas natural reefs can provide more contiguous habitat, thereby shaping the spatial distribution of reef-associated fish communities.

### 4.2. Reef characteristics comparison

#### 4.2.1. Structure type.

Although structure type was not used in statistical modeling due to the collinearity with relief, boats had consistently higher fish biomass than other reef structure configurations, excluding the Big Pile, a unique pile of 6,500 tons of concrete railroad ties (Fig 3). This is consistent with numerous other studies that found metal ships have consistently higher fish biomass when compared to other structures, particularly low-lying concrete structures. Patxon et al. [63] found that ships used as artificial reef structures off the coast of the southeastern United States had higher fish abundance, biomass, and species richness than low-complexity artificial reef structures and naturally occurring rocky reef formations. Jaxion-Harm and Szedlmayer [14] measured red snapper in the northern Gulf of Mexico and found that large artificial reef structures (ships and oil platforms) supported larger fish than other artificial reef types; however, large numbers of small fish were observed in smaller structures such as pyramids. Conversely, Bollinger and Kline [61] calculated fish biomass associated with ships in the northwestern Gulf of Mexico and found that ships had the lowest fish biomass of the structures measured. However, this is likely due to the factors measured for the artificial reef structures. For example, the ship used in this study had lower relief than the oil platforms and a larger footprint area than any of the other sites.

#### 4.2.2. Vertical relief.

Vertical relief of structures was a significant predictor of fish biomass in the present study. The strong relationship between vertical relief and biomass suggests that taller structures provide more favorable habitat for fish communities (Fig 3B). High-relief structures in the RGV Reef included boats and large piles of concrete railroad ties (400 tons or more). Similarly, White et al. [45] found that vertical relief was positively associated with fish backscatter (a biomass metric); however, this relationship varied when modeled with interaction terms including distance and depth. They found that backscatter declined at distances greater than 100 m from low-relief structures but increased at distances greater than 450 m for high-relief structures [45]. Additionally, they found that the interaction between vertical relief and depth affected backscatter, with backscatter increasing with depth at reefs with vertical relief ranging from 5 to 22 m [45]. They concluded that the observed differences in depth and relief likely reflect differences in fish community composition and average fish body size. Patterson et al. [55] compared fish abundance on artificial reefs versus natural reefs and suggested that the greater vertical relief of artificial structures likely contributed to the higher abundance associated with artificial reefs. Similarly, Holland et al. [31] found that high vertical relief was an important factor in the formation of desirable fish habitat. The high-relief artificial reef structures surveyed by Holland et al. [31] ranged from 4 to 12 m in vertical relief, a range similar to that of the high-relief structures in the present study. The study by Holland et al. [31] suggested that affinity for higher vertical relief in fish might be influenced by increased access to foraging resources at higher levels in the water column, while still benefiting from refuge within structure.

#### 4.2.3. Distance to structure.

Distance to reef structure was a significant predictor of fish biomass. The decline in biomass with increasing distance from reef structures suggests that fish strongly associated with structural habitat, as indicated by the heatmap (Fig 9). However, the “away” model (fish farther than 20 m from structure) showed a less significant effect of distance, suggesting that distance becomes less relevant once fish are farther from structures (Fig 7C). Similar to the results of the present study, Boswell et al. [4] estimated fish biomass around oil platforms and found the greatest acoustic fish biomass within the first 10 m of reef structure. Similarly, Egerton et al. [64] conducted hydroacoustic surveys around oil platforms and found the highest fish backscatter and density within 25 m of the structures. Becker et al. [43] studied two types of artificial reef structures and found that distance was a significant predictor of biomass; however, the pattern differed between the two structure types. They found that for concrete modules, biomass steadily decreased with increasing distance up to 60 m; however, for steel structures, biomass remained consistent up to 60 m and then declined rapidly beyond 60 m [43]. This difference may be due to variation in vertical relief between the two structure types, highlighting interactions among artificial reef characteristics.

#### 4.2.4. Depth.

The present study also found that depth was a significant predictor of fish biomass; however, the depth model showed a more pronounced effect of depth for fish farther than 20 m from structure, indicating that depth had a more substantial influence on biomass farther from structure (Fig 7A). However, the depth gradient in this study was limited (ranging from 18.4 to 23.4 m). This pattern may also be an artifact of material placement, as high-relief structures were more frequently deployed in deeper reef areas. Patterson et al. [55] found significant differences in fish abundance with depth using ROV surveys in the northern Gulf of Mexico. Similarly, Garner et al. [65] conducted ROV surveys in the north-central Gulf of Mexico and found that the highest fish density was associated with deep natural reef sites; however, relief and complexity had a stronger effect. Becker et al. [43] also found depth to be a significant predictor of biomass, with the highest biomass values associated with intermediate depths for four artificial reefs off the coast of New South Wales, Australia. In addition, Becker et al. [43] found that distance to a structure, age of structure, and season were also significant predictors of fish biomass. Age was not a significant model factor in the present study, likely because the age range at the structure site was small (4–9 years), as the reef was established only in 2016. Future studies should investigate the relationships among seasonality, depth, and fish biomass, especially in relation to position in the water column, as a strong thermocline is present during the summer months and less pronounced in winter.

#### 4.2.5. Distance to the reef boundary.

Distance to the reef boundary was a significant predictor of fish biomass in the present study. Fish biomass was highest at the boundary edge and at the farthest distance from the boundary edge, with slightly lower biomass observed at intermediate distances. Higher biomass at habitat boundaries has been documented in numerous ecosystems, including reefs, and is often referred to as the “edge effect” [66–69]. A previous study investigating coral reef edge habitats found higher species richness and increased abundances of carnivorous and omnivorous species at the reef edge [66]. Similarly, Smith et al. [67] studied the edge effect in seagrass habitats and found that fish density was higher at the seaward edge than at the center of the habitat. Dorenbosch et al. [68] also found higher densities of reef-associated and generalist fish species near the reef edge than on seagrass beds. These patterns may result from foraging behavior in which fish forage outside of the reef area but seek refuge within the reef boundary. Sambrook et al. [66] found significant differences in fish abundance across feeding guilds, with planktivores showing a significant adverse edge effect, suggesting that feeding ecology plays a role in habitat use and distribution patterns within reefs. Additionally, species composition and life-stage partitioning may influence habitat use, with juveniles or competing species using edge habitat differently than adults or dominant competitors. The elevated biomass at the reef boundary in the present study aligns with established ecological theory and empirical evidence from multiple systems, suggesting that reef edges function as important foraging and refuge-linked hotspots that structure fish distributions across the seascape.

#### 4.2.6. Complexity.

Structural complexity was not directly investigated in the present study; however, it has been shown to be an important factor in artificial reef design and fish biomass. Paxton et al. [63] surveyed 30 reefs, including both natural and artificial, off the coast of North Carolina, USA. They found that intermediate reef complexity supported the highest fish abundance in natural reefs, but this pattern differed slightly in artificial reefs, where higher complexity was associated with the highest fish abundance. However, Paxton et al. [63] assessed complexity using rugosity, which combines height with surface area, and did not separate vertical relief from surface area, which might better explain the difference in biomass associated with metal ships versus low-lying concrete artificial reef structures. The present study examined vertical relief separately from structure type and found a significant relationship when decoupled from material category, as the two predictors were highly collinear. Lemoine et al. [70] found no significant effect of habitat complexity on fish biomass; however, this pattern might have been skewed by the presence of metal ships, as the effect of habitat complexity became more pronounced when ships were removed. Granneman and Steele [71] found that artificial reef structure with higher structural complexity had significantly higher biomass and fish density than nearby natural reefs. These results suggest complexity is important to consider when designing and planning future artificial reefs. Because structural complexity was not directly quantified in the present study, future work incorporating rugosity or three-dimensional structure metrics would improve understanding of how complexity influences fish biomass within this artificial reef system.

#### 4.2.7. Footprint area.

Although footprint area was not a significant factor in the “all fish” model of the present study, for the subset of fish farther than 20 m from structure, area became a significant term, suggesting larger reef areas may play an important role in influencing fish biomass at a distance (Fig 7E). Although this was not found in the study by Bollinger and Kline [61], where the site with the largest footprint, a ship with a mean surface area of 3,600 m^2^, had the lowest biomass per reefing area, this is likely due to other reef characteristics, including distance offshore and vertical relief. Lemoine et al. [70], however, did find an increase in fish abundance relative to footprint area, suggesting this can influence fish communities but may be location-specific and affected by the total amount of habitat, not just the size of individual reef sites.

#### 4.2.8. Model performance.

Although the GAM models identified several significant predictors of fish biomass, the proportion of deviance explained by the best-fitting models was moderate, suggesting that additional factors likely influence fish distributions within the reef system. Variables not included in the present analysis—such as species-specific habitat preferences, prey availability, current patterns, seasonal variability, and behavioral aggregation dynamics—may contribute to the remaining unexplained variability. This study captured instantaneous temporal and spatial patterns; additional processes, such as diel movement, spawning aggregations, or seasonal migrations, likely also influence biomass patterns; however, they were not captured in this study. Future studies integrating acoustic surveys with environmental measurements and species-level observations could help further explain these factors influencing the distribution of fish biomass.

### 4.3. Total fish biomass estimation

The total extrapolated fish biomass within the RGV Reef area was 24,396.5 kg based on the average fish biomass associated with each structure site type, the number of structures within each site type, and the total fish biomass measured between structure sites. Of this, structure-associated fish accounted for 19,515 kg across an estimated 252,400 m^2^ of reef structure area. These values provide a system-level estimate of fish biomass supported by the artificial reef ecosystem. To contextualize these estimates, the results were compared with previously published studies that quantified fish biomass on natural and artificial reefs. Paxton et al. [63] measured 43,570 kg of fish across 30 reef sites, including a combination of natural and artificial reefs, covering an area of 29,520 m^2^ spanning 246 scuba-based belt transects (Table 2). In their study, fish were visually identified, and biomass was calculated using the length-weight relationship (*W = aL*^*b*^) where L is length, and W is weight. Using similar methodologies, Lemoine et al. [70] estimated 2,162 kg of fish across 23 reef sites, including both natural and artificial reef structures, covering an area of 8,640 m^2^ from 72 belt transects (Table 2). Coker et al. [72] also used similar survey methods to estimate fish biomass across 94 reef sites over a larger spatial extent (2,000 km^2^) and reported biomass densities ranging from 0.0006 to 2.1 kg m^-2^ (Table 2). Because these studies used different sampling approaches, survey extents, and analytical methods, their raw biomass estimates are not directly comparable. To provide a more consistent basis for comparison, biomass values from each study were standardized by reef area to produce biomass density estimates (kg m^-2^), providing a valuable basis for comparison. Biomass density was calculated by dividing the fish biomass by the area of reef habitat surveyed, typically defined by the spatial extent of diver belt surveys conducted over reef structures. This area-standardized metric provides a commonly used approach for comparing fish biomass across reef systems that differ in size, sampling design, and methodology.

**Table 2:** Reef-wide scaled fish biomass comparison.

Study	Location	Fish Biomass	Number of Sites	Area	Scaled Biomass	Reef Type	Methodology
Present study	Texas Gulf coast, USA	19,515 kg	477 reef sites	252,400 m2	0.08 kg m-2	Artificial	Hydroacoustics
Paxton et al. [63]	North Carolina coast, USA	43,570 kg	30 reef sites	29,520 m2	1.48 kg m-2	Natural & artificial	Scuba transects
Lemoine et al. [70]	North Carolina coast, USA	2,162 kg	23 reef sites	8,640 m2	0.25 kg m-2	Natural & artificial	Scuba transects
Coker et al. [72]	Red Sea, Saudi Arabia	2.7 t ha − 1 ± 0.31	94 reef sites	2000 km2	0.0006–2.1 kg m-2	Natural	Belt transects

**Table 2.** Comparison of reef-associated fish biomass estimates across artificial and natural reef studies. Reported biomass values from each study were standardized to biomass density (kg m ⁻ ²) by dividing the total estimated fish biomass by the surveyed reef area to facilitate comparison among studies with different sampling methods and spatial scales. Methodologies included hydroacoustic surveys and diver-based visual transects. Scaled biomass values represent approximate biomass density estimates and should be interpreted in the context of methodological differences among studies.

When standardized by reef structure area, the present study estimated 0.08 kg m^-2^ of structure-associated fish biomass, within the range reported by other studies. Paxton et al. [63] and Lemoine et al. [70] estimated biomass densities of 1.48 kg m^-2^ and 0.25 kg m^-2^, respectively, while estimates reported by Coker et al. [72] ranged from 0.0006 to 2.1 kg m^-2^ (Table 2). Although methodological differences among these studies limit strict quantitative comparisons, the area-standardized values provide a useful contextual framework, indicating that the fish biomass supported by the RGV Reef falls within the range observed in other natural and artificial reef habitats. Methodological differences among studies also reflect the range of available tools for estimating reef-associated fish biomass. Diver-based visual surveys allow species-level identification and direct length estimation but are constrained by visibility, depth limitations, and spatial coverage. In contrast, hydroacoustic surveys can rapidly sample larger areas of the water column and are not limited by visibility conditions, but they do not allow direct species identification. Many hydroacoustic studies estimate relative fish biomass using acoustic backscatter metrics such as the nautical area scattering coefficient (NASC) [43,45], which serves as a proxy for biomass density within a study but cannot be easily converted to absolute biomass or carbon estimations and is not easily communicated to broader audiences.

In the present study, acoustic target strength measurements were converted to estimates of fish length and biomass using published target strength-length relationships and generalized length-weight equations. This approach allows hydroacoustic observations to be translated into biologically interpretable metrics such as biomass and carbon content, facilitating comparison with studies that use visual survey methods. However, it is important to note that these estimates rely on generalized relationships rather than species-specific parameters. Therefore, to evaluate sensitivity to assumptions used in the estimation of biomass, sensitivity analyses were conducted. For fish schools specifically, the expected individual fish weight was varied by ±5%, resulting in relatively small changes in total reef-wide biomass (−4.5% to +1.8%) and structure-associated biomass (±2%). These results indicate that while uncertainty in assumed fish weight can influence biomass estimates, the overall magnitude and spatial patterns remain robust. For fish tracks, sensitivity analysis of length-weight parameter uncertainty revealed a moderate effect on biomass estimates. The application of lower-bound parameters (reflective of fish aggregations with more grey triggerfish) resulted in a ~ 9% decrease in total biomass, whereas upper-bound parameters (reflective of fish aggregations with more red snapper) produced an increase of approximately 17%. This asymmetry indicates that uncertainty in length-weight relationships is skewed towards higher biomass estimates, likely reflecting the nonlinear influence of allometric scaling at larger body sizes. As a result, the baseline model used in the present study, derived from averaging of multiple GoM species, appears to provide a conservative estimate of fish biomass. Despite the observed variability and uncertainty, the magnitude of change remains relatively constrained, suggesting that the parameters used for biomass calculations yield a robust and ecologically realistic approximation for fish biomass.

### 4.4. Artificial reefs as restoration tools

Artificial reefs are important conservation tools to promote fisheries. Prior observations and sonar surveys suggested relatively sparce fish populations in the RGV Reef area before the deployment of structures. Granneman and Steele [71] found that physically similar artificial reefs and natural reefs held similar fish assemblages. This phenomenon was supported by the results of Lemoine et al. [70], who found that fish biomass was similar on natural rocky reefs and on low-lying concrete artificial reef structures. Granneman and Steele [71] also found identical fish assemblages on artificial reefs and natural reefs with similar physical characteristics, such as vertical relief and rugosity. These results support the present study and the use of artificial reefs to mimic or replace natural reefs in areas where natural reefs are degraded or where natural rigid substrate is lacking. Artificial reefs can also increase connectivity between habitats, which has significant potential in degraded or patchy habitats [50]. In addition to providing habitat in degraded areas, artificial reefs also have the potential to serve as stepping-stone habitat as species move poleward and deeper as pressures from urbanization and climate impacts increase [50]. Additionally, artificial reefs often exhibit greater structural complexity than natural reefs, which is associated with higher fish biomass, as previously mentioned. Enhanced structural complexity also provides greater surface area, supporting the colonization of sessile organisms and invertebrates. Granneman and Steele [71] found a positive relationship between fish biomass and invertebrate density on reef structures, with significantly higher invertebrate densities observed on artificial reefs compared to natural reefs. These findings underscore the interconnected relationships among reef-associated organisms and highlight the broader community-level benefits of artificial reef structures.

### 4.5. Contribution of artificial reef fish to blue carbon ecosystems

There has been a longstanding debate on whether coral reef ecosystems act as net carbon sinks or sources. However, artificial reefs are often developed in areas where reefs are degraded or previously absent, providing a novel ecosystem where carbon can accumulate for short-term storage in organisms’ bodies or long-term storage in sediment. ‘Fish carbon’ is emerging as a promising field of study to account for the contribution of marine vertebrates to organic carbon storage through numerous processes, including defecation, trophic integration, vertical mixing, death, and burial [73,74]. The present study estimated 0.18 t C ha ⁻ ¹ in standing fish stock associated with structure (Table 3). The above-ground carbon of established blue carbon ecosystems, such as seagrass beds, ranges from 0.2 to 0.425 t C ha ⁻ ¹ (Table 3, [75,76]) Additionally, the carbon stored within sponges, soft corals, and invertebrates on reefs ranges from 0.2–0.8 t C ha ⁻ ¹ (Table 3, [77]). This similarity highlights that fish communities supported by artificial reefs represent a dynamic pool of short-term standing stock organic carbon stored in living biomass. While these individual fish contribute to a relatively short-term carbon reservoir due to mortality, predation, and movement, this biomass is continually replenished through recruitment and growth, maintaining a standing stock of carbon over time that can contribute to longer-term sequestration through processes such as mortality, burial, and trophic transfer. Although mechanisms and turnover rates differ from those in vegetated blue carbon ecosystems, the creation of reef habitat promotes the formation of a carbon-rich biological community that would not otherwise occur, underscoring the potential of artificial reefs to enhance local carbon pools and contribute to broader marine carbon dynamics.

**Table 3:** Reef-wide scaled carbon comparison.

Species	Location	Surface carbon	Study
Fish	Texas Gulf coast, USA	0.25 t C ha ⁻ ¹	Present study
Seagrasses	Laguna Madre, Texas, USA	0.2 - 0.4 t C ha ⁻ ¹	Arellano-Méndez et al. [78], Kaldy & Dunton [79]
Sponges, soft corals, invertebrates	Australia & Belize	0.2–0.8 t C ha ⁻ ¹	Wilkinson & Cheshire [80]

**Table 3.** Comparison of surface carbon standing stock estimates among marine organisms and ecosystems reported in previous studies. Values represent approximate areal carbon densities (t C ha ⁻ ¹) associated with living biomass and are presented to contextualize the fish-derived carbon estimates calculated for the Rio Grande Valley (RGV) Reef in the present study.

In addition to storing carbon in their biomass, fish further influence carbon dynamics through vertical movements. Davison et al. [81] estimated that mesopelagic fish contribute between 10% and 40% of carbon export through diel vertical migrations. At night, mesopelagic fish travel to surface waters to feed, then return to deep waters during the day, actively transporting carbon to deep layers through respiration and excretion, a mechanism often overlooked and underrepresented [74,82]. Trueman et al. [83] showed that over half of the biomass of deep demersal fish communities is supported by biological nutrient flux and represents a significant net carbon sink. Fecal pellets are also being investigated for their contribution to organic carbon burial in marine sediments [74,78,79]. Mariani et al. [80] suggested that overfishing prevents blue carbon sequestration by reducing natural carbon accumulation through deadfall. This study recognized the potential for standing fish stocks to serve as blue carbon sinks, with significant implications for reef ecosystems, particularly artificial reefs. After death, large fish carcasses sink and become incorporated into sediment [80]. The death of large fish and other marine animals is another pathway of carbon sequestration that can be enhanced through the development of artificial reefs, which provide more habitat for fish and support recovering fisheries. Taken collectively, the carbon stored within fish biomass associated with artificial reefs, the comparable magnitudes to the above-ground seagrass and benthic invertebrate carbon pools, and the substantial export of organic matter through fish movements and deadfall all underscore the multifaceted role of artificial reefs in blue carbon pathways. These structures not only support fish but also enable the formation of carbon-rich communities, stimulate vertical and horizontal carbon redistribution, and enhance opportunities for long-term sequestration in deep waters and sediments. As such, artificial reefs may serve as important, though currently undervalued, contributors to marine carbon budgets, especially in regions where natural habitats have been lost or degraded.

## 5. Limitations

Several limitations should be considered when interpreting these results. First, the acoustic methods used in this study cannot directly identify species; therefore, biomass estimates were derived from generalized relationships derived in the published literature. Additionally, the sonar used in this study was constrained by the acoustic dead zone, which reduced detection of fish closely associated with structure, likely leading to an underestimation of biomass. Second, carbon estimates were calculated from biomass values and averages of wild-caught marine fish species, rather than being directly measured. Third, this study provides a snapshot of time and does not capture seasonal or interannual variability in fish distributions. In addition, structure-associated and non-structure-associated fish were summarized using different temporal approaches, where structure-associated fish were based on a single sampling day per site and non-structure-associated observations were compiled across multiple sampling days. This difference in temporal aggregation may introduce some bias when comparing spatial groupings. However, because non-structure-associated observations were collected across non-overlapping transects spanning a large spatial extent, the likelihood of repeatedly sampling the same individuals is low, and any resulting bias is expected to be limited. Finally, structural complexity was approximated using categorical variables such as relief and structure type rather than detailed three-dimensional measurements. Further studies integrating acoustic surveys with ground truthing, species identification, and seasonal sampling would improve understanding of how artificial reefs influence fish biomass and carbon storage over time. Despite these limitations, the present study provides a reasonable estimate of fish biomass and associated carbon within standing fish populations associated with the RGV Reef, offering a useful baseline for future assessments of artificial reef ecosystems.

## 6. Conclusions and future research directions

This study used hydroacoustic measurements, along with literature-derived values and equations, to estimate fish biomass and carbon content in the standing fish stock associated with the RGV Reef. Based on collected data, the total estimated fish biomass was 24.4 metric tons of fish and 2.81 metric tons of fish carbon. These findings demonstrate that the RGV Reef is a crucial habitat for reef-associated fish species, supporting substantial fish populations relative to baseline conditions for soft-bottom habitats, and suggesting that artificial reef deployment enhances fish populations and helps to support recovering fisheries. The RGV Reef also provides an area for carbon to accumulate as a standing stock in fish biomass, and through the biological pump and sedimentation, representing a potential pathway for increasing carbon in the sediment. These processes highlight the reef’s potential to capture, process, and store organic carbon, underscoring its potential role as an emerging blue carbon ecosystem. The present study provides a quantitative estimate of standing fish biomass and associated carbon within the RGV Reef, demonstrating that artificial reefs can support substantial biological carbon pools. While these findings highlight the potential for reef-associated fish communities to contribute to marine carbon cycling through processes such as trophic transfer, vertical movement, and eventual deposition, this study does not directly quantify long-term carbon sequestration. Therefore, the role of artificial reefs in sedimentary carbon storage and long-term blue carbon dynamics remains an important area for future research. As such, artificial reefs may represent a previously underexplored component of marine carbon systems, though additional work is needed to determine their contributions to long-term sequestration. This research offers an important foundation for incorporating fish carbon associated with artificial reef systems into the broader global blue carbon initiative [73,74,81,80]. Combining these ideas with other research on sessile filter-feeding organisms, soft corals, and benthic organisms within an artificial reef provides a more comprehensive understanding of the marine carbon cycle and the roles of these organisms in carbon sequestration. This phenomenon will not only advance reef conservation and fisheries management but also strengthen arguments for including these structures in carbon accounting frameworks. The results of this study provide a foundation for policymakers, conservationists, and fisheries managers to incorporate artificial reefs into sustainable marine resource planning. By leveraging artificial reef structures as blue carbon assets, we can strengthen efforts to build climate resilience, enhance fisheries, and restore marine habitats.

Future research should build on this foundation by quantifying carbon dynamics within reef-associated sediments to assess burial rates and long-term sequestration potential in oligotrophic reef systems. Broader investigations across other artificial reef sites could further evaluate the consistency of these processes and their connectivity with natural blue carbon ecosystems. Such work will be essential for refining estimates of artificial reef contributions to global carbon budgets and for strengthening the case for their inclusion in climate mitigation strategies. By increasing fish biomass, artificial reefs enhance trophic transfer and stimulate carbon cycling in marine environments, supporting broader goals of blue carbon initiatives. Given the increasing importance of nature-based solutions in mitigating climate change, integrating artificial reef structures into coastal management strategies may offer dual benefits: promoting marine biodiversity while supporting processes that may enhance carbon sequestration and long-term carbon storage. Future research should explore long-term carbon retention in these systems, as well as the roles of different reef materials and configurations in optimizing carbon storage and ecological benefits.

## Supporting information

S1 Fig Partial effects of distance to the nearest structure on log-transformed fish weight across three spatial groupings predicted from generalized additive models (GAMs). Panel (A) shows predictions for all fish observations and panel (B) includes only fish located farther than 20 meters from structure. Fish only within 20 m of structure are not shown because distance to structure was not a significant term in the model. Each curve represents the mode led smooth effect of depth, holding other variables constant. Shaded regions indicate 95% confidence intervals. Differences affect strength and shape illustrate how proximity to structure influences fish biomass.
(DOCX)

S2 Fig Partial effects of average depth on log-transformed fish biomass across three spatial groupings predicted from generalized additive models (GAMs). Panel (A) shows predictions for all fish observations, panel (B) includes only fish located within 20 meters of structure, and panel (C) includes only fish located farther than 20 meters from structure. Each curve represents the model smooth effect of depth, holding other variables constant. Shaded regions indicate 95% confidence intervals. Differences in curve shape and effect magnitude highlight variability in the relationship between depth and fish biomass depending on proximity to structure.
(DOCX)

S3 Fig Partial effects of relief category on log-transformed fish biomass across three spatial groupings predicted from generalized additive models (GAMs). Panel (A) shows predictions for all fish observations, panel (B) includes only fish located within 20 meters of structure, and panel (C) includes only fish located farther than 20 meters from structure. Relief categories are ordered from low to high relief. Points represent model predicted means, and error bars reflect 95% confidence intervals.
(DOCX)

S4 Fig Partial effects of distance to the reef boundary on log-transformed fish biomass across two spatial groupings predicted from generalized additive models (GAMs). Panel (A) shows predictions for all fish observations and panel (B) includes only fish located farther than 20 meters from structure. Fish only within 20 m of structure are not shown because distance to structure was not a significant term in the model. Each curve represents the modeled smooth effect of distance, holding other variables constant. Shaded regions indicate 95% confidence intervals. Differences affect strength and shape illustrate how proximity to the boundary influences fish biomass.
(DOCX)

S5 Fig Partial effects of structure area on log-transformed fish biomass for only fish located farther than 20 meters from structure predicted from the generalized additive model (GAM). The curve represents the mode led smooth effect of distance, holding other variables constant. Shaded regions indicate 95% confidence intervals.
(DOCX)

S1 Table Reef site configurations in the Rio Grande Valley Reef. (DOCX)

S2 Table Model comparison overview for all three models based on spatial groupings of fish. (DOCX)

S3 Table Dataset used to calculate average fish biomass by structure type and relief category presented in Figure 3. (DOCX)

S4 Table Complete dataset used for statistical and spatial analyses presented in Figures 5–7. Variables include fish cluster identification number, fish biomass, distance, depth, nearest site, structure characteristics, and values used for all statistical and spatial analyses.
(DOCX)

S5 Table Dataset used to for total biomass and fish carbon calculations for the entire RGV Reef area presented in Figure 8. (DOCX)

S1 File ArcGIS spatial analysis workflow for fish biomass analysis. (PDF)

S2 File R scripts for statistical analyses, modelling, and figure generation. (PDF)

## Abbreviations

AIC: Akaike information criterion
ANOVA: analysis of variance
FADs: fish aggregation devices
GAM: generalized additive model
GoM: Gulf of Mexico
PCA: principal components analysis
RVG: Rio Grande Valley
REML: restricted maximum likelihood
VIF: variance inflation factor
