# Supplementary material for: Comparison of fish biomass and fish carbon content associated with reef sites at the Rio Grande Valley artificial reef in the Gulf of Mexico
Source: PLoS One. 2026 Jun 4;21(6):e0350204. doi: 10.1371/journal.pone.0350204 (PMC13235911; doi:10.1371/journal.pone.0350204)
Supplement: S2 Fig — Panel (A) shows predictions for all fish observations, panel (B) includes only fish located within 20 meters of structure, and panel (C) includes only fish located farther than 20 meters from structure. Each curve represents the model smooth effect of depth, holding other variables constant. Shaded regions indicate 95% confidence intervals. Differences in curve shape and effect magnitude highlight variability in the relationship between depth and fish biomass depending on proximity to structure. (DOCX) [file pone.0350204.s002.docx]

**A**


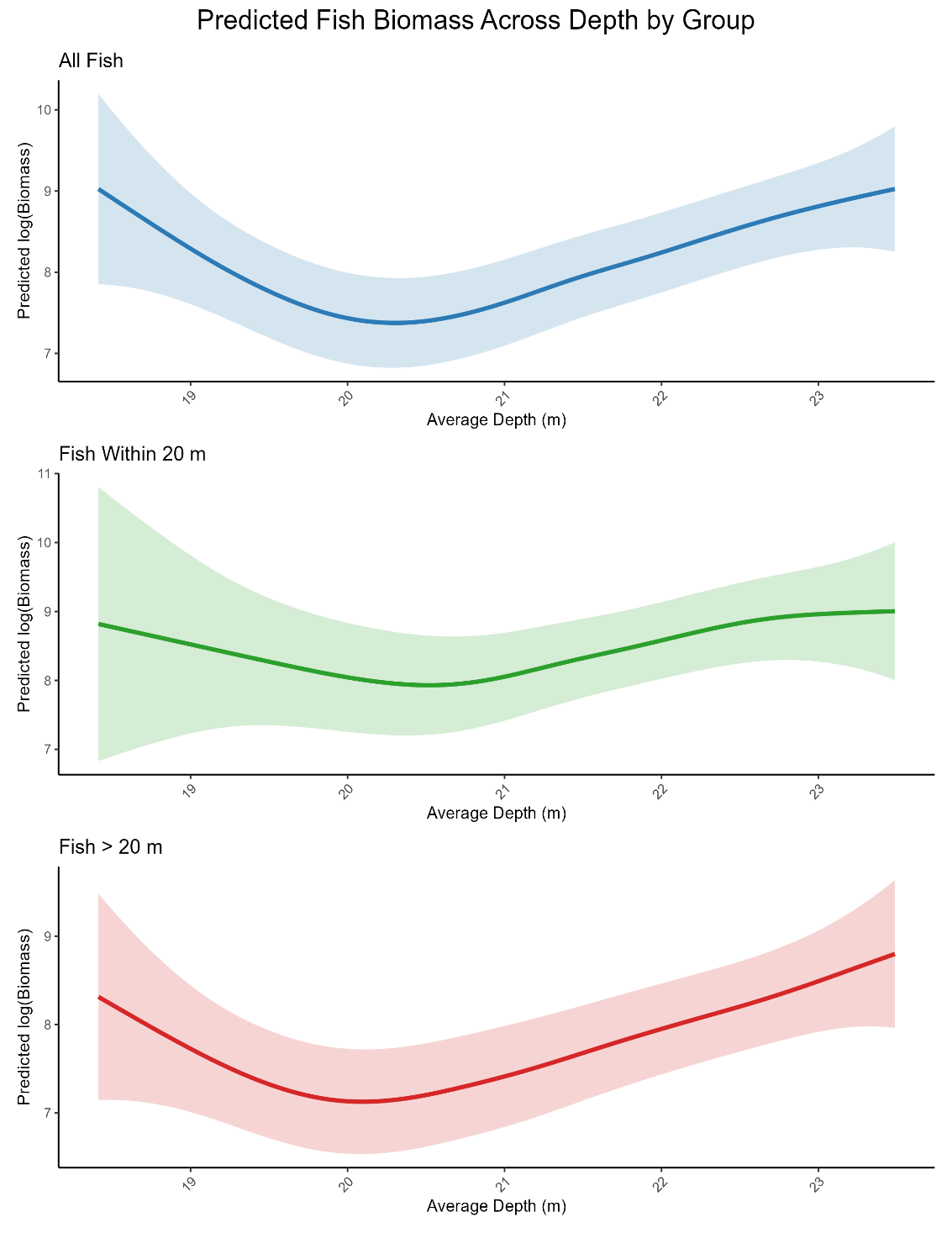


**C**

**B**

**S2 Fig.** **Partial effects of average depth on log-transformed fish biomass across three spatial groupings predicted from generalized additive models (GAMs).** Panel (A) shows predictions for all fish observations, panel (B) includes only fish located within 20 meters of structure, and panel (C) includes only fish located farther than 20 meters from structure. Each curve represents the model smooth effect of depth, holding other variables constant. Shaded regions indicate 95% confidence intervals. Differences in curve shape and effect magnitude highlight variability in the relationship between depth and fish biomass depending on proximity to structure.
