# Supplementary material for: Comparison of fish biomass and fish carbon content associated with reef sites at the Rio Grande Valley artificial reef in the Gulf of Mexico
Source: PLoS One. 2026 Jun 4;21(6):e0350204. doi: 10.1371/journal.pone.0350204 (PMC13235911; doi:10.1371/journal.pone.0350204)
Supplement: S3 Fig — Panel (A) shows predictions for all fish observations, panel (B) includes only fish located within 20 meters of structure, and panel (C) includes only fish located farther than 20 meters from structure. Relief categories are ordered from low to high relief. Points represent model predicted means, and error bars reflect 95% confidence intervals. (DOCX) [file pone.0350204.s003.docx]

**A**


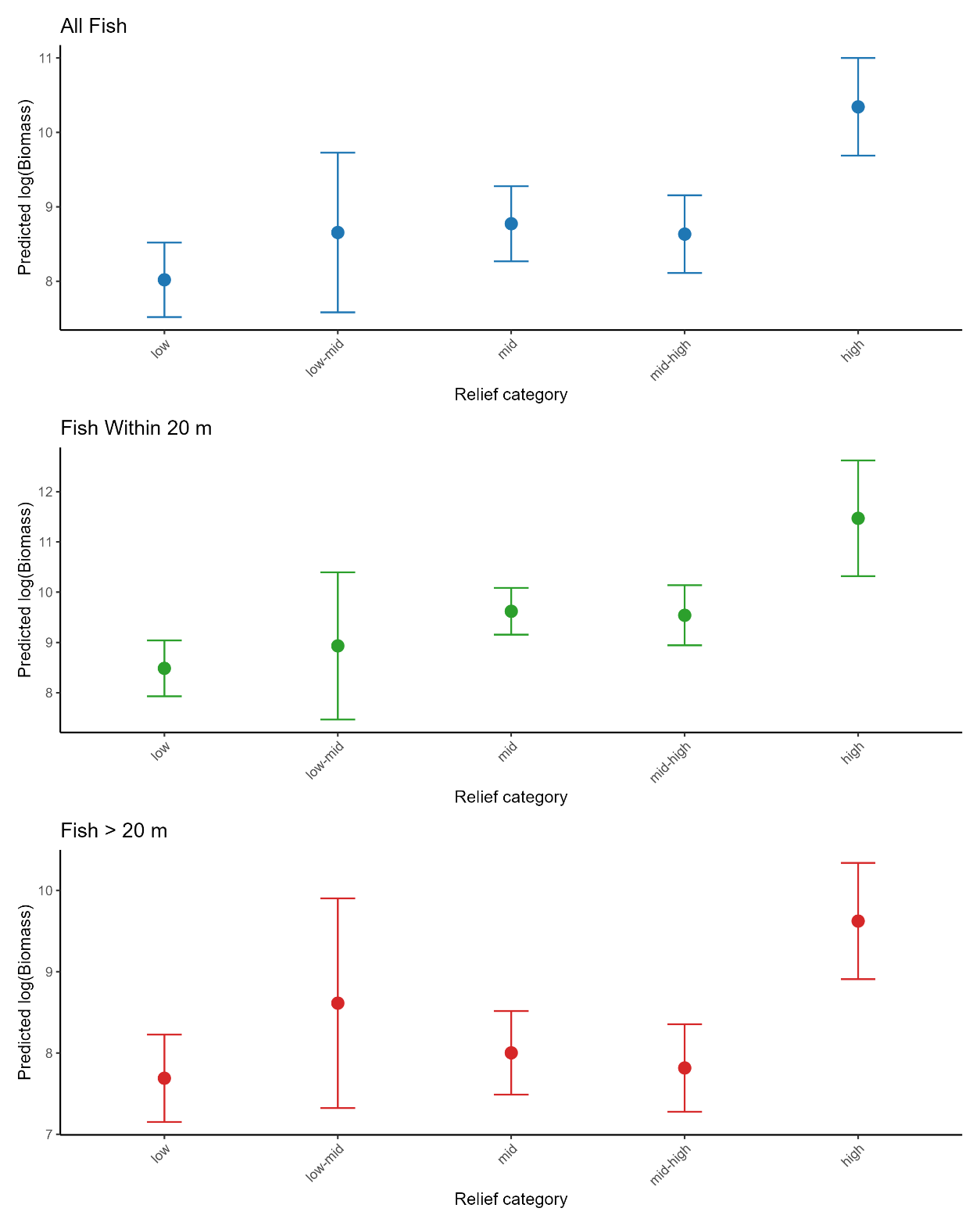


**C**

**B**

**S3 Fig. Partial effects of relief category on log-transformed fish biomass across three spatial groupings predicted from generalized additive models (GAMs).** Panel (A) shows predictions for all fish observations, panel (B) includes only fish located within 20 meters of structure, and panel (C) includes only fish located farther than 20 meters from structure. Relief categories are ordered from low to high relief. Points represent model predicted means, and error bars reflect 95% confidence intervals.
