# Supplementary material for: Comparison of fish biomass and fish carbon content associated with reef sites at the Rio Grande Valley artificial reef in the Gulf of Mexico
Source: PLoS One. 2026 Jun 4;21(6):e0350204. doi: 10.1371/journal.pone.0350204 (PMC13235911; doi:10.1371/journal.pone.0350204)
Supplement: S4 Fig — Panel (A) shows predictions for all fish observations and panel (B) includes only fish located farther than 20 meters from structure. Fish only within 20 m of structure are not shown because distance to structure was not a significant term in the model. Each curve represents the modeled smooth effect of distance, holding other variables constant. Shaded regions indicate 95% confidence intervals. Differences affect strength and shape illustrate how proximity to the boundary influences fish biomass. (DOCX) [file pone.0350204.s004.docx]

**A**


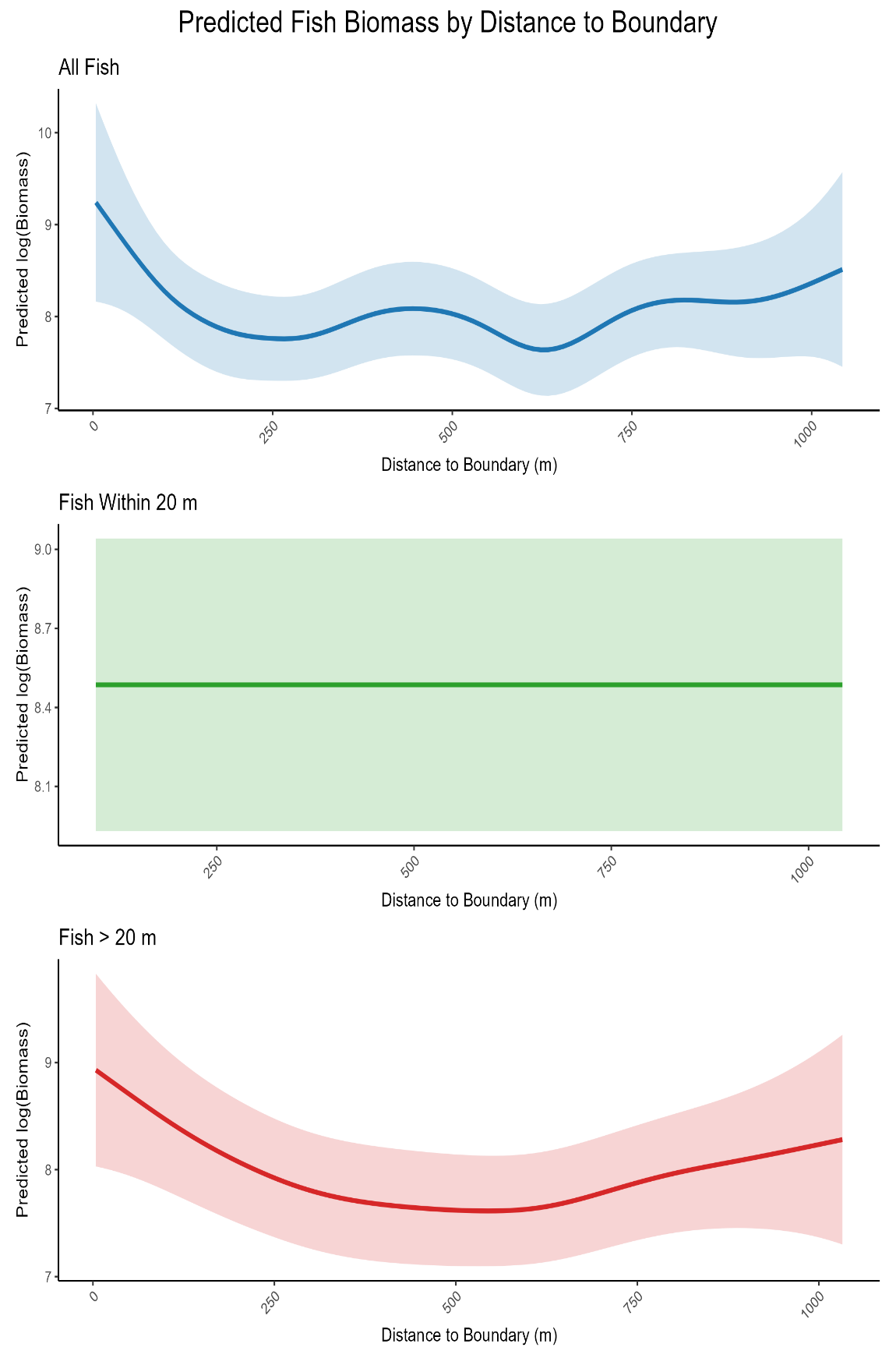

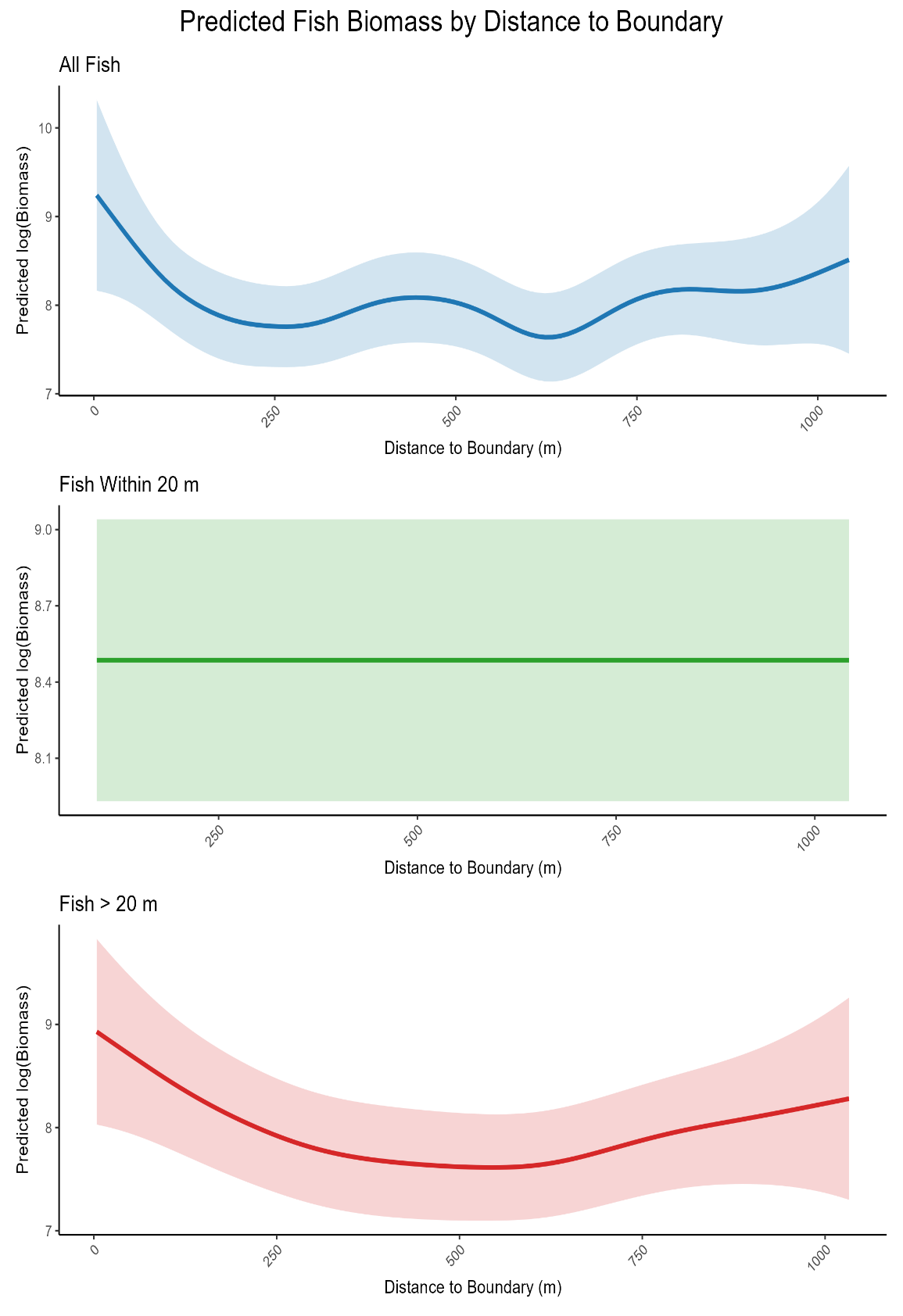


**B**

**S4 Fig. Partial effects of distance to the reef boundary on log-transformed fish biomass across two spatial groupings predicted from generalized additive models (GAMs).** Panel (A) shows predictions for all fish observations and panel (B) includes only fish located farther than 20 meters from structure. Fish only within 20 m of structure are not shown because distance to structure was not a significant term in the model. Each curve represents the modeled smooth effect of distance, holding other variables constant. Shaded regions indicate 95% confidence intervals. Differences affect strength and shape illustrate how proximity to the boundary influences fish biomass.
