# Supplementary material for: Comparison of fish biomass and fish carbon content associated with reef sites at the Rio Grande Valley artificial reef in the Gulf of Mexico
Source: PLoS One. 2026 Jun 4;21(6):e0350204. doi: 10.1371/journal.pone.0350204 (PMC13235911; doi:10.1371/journal.pone.0350204)
Supplement: S5 Fig — The curve represents the mode led smooth effect of distance, holding other variables constant. Shaded regions indicate 95% confidence intervals. (DOCX) [file pone.0350204.s005.docx]

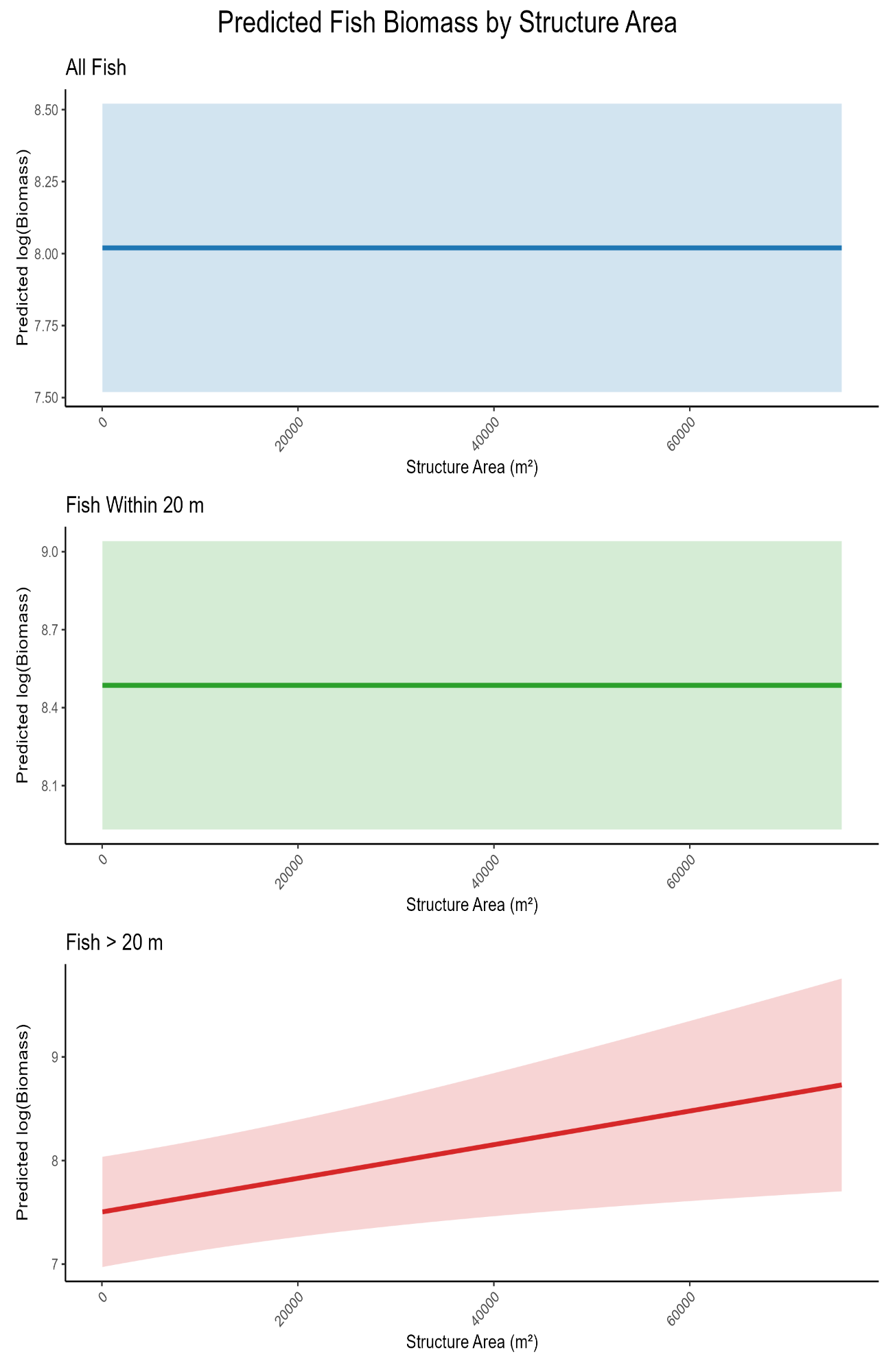


**S5 Fig. Partial effects of structure area on log-transformed fish biomass for only fish located farther than 20 meters from structure predicted from the generalized additive model (GAM).** The curve represents the mode led smooth effect of distance, holding other variables constant. Shaded regions indicate 95% confidence intervals.
