# Supplementary material for: Comparison of fish biomass and fish carbon content associated with reef sites at the Rio Grande Valley artificial reef in the Gulf of Mexico
Source: PLoS One. 2026 Jun 4;21(6):e0350204. doi: 10.1371/journal.pone.0350204 (PMC13235911; doi:10.1371/journal.pone.0350204)
Supplement: S1 Table — (DOCX) [file pone.0350204.s006.docx]

| **Structure site type name** | **Description of structure in site** | **Replicates in reef** |
| --- | --- | --- |
| 1CB | 1 pallet cinderblock | 73 |
| 10T Concrete | 10 ton concrete | 16 |
| 16LP | 16 low profile modules | 6 |
| 16MX | 16 low profile modules and 16 pyramids | 6 |
| 16PY | 16 pyramids | 6 |
| 1MX | 1 low profile modules and 1 pyramid | 5 |
| 1PY | 1 pyramid | 7 |
| 2T Concrete | 2 ton concrete | 15 |
| 200T RR | 200 ton rr ties | 1 |
| 25T RR | 25 tons rr ties | 2 |
| 250T RR | 250 tons rr ties | 22 |
| 3CB | 3 pallets cinderblock | 65 |
| 30CB | 30 pallets cinderblock | 3 |
| 4LP | 4 low profile modules | 6 |
| 4MX | 4 low profile modules and 4 pyramids | 6 |
| 4PY | 4 pyramids | 9 |
| 50T RR | 50 tons rr ties | 2 |
| 500T RR | 500 tons rr ties | 2 |
| Big Pile | big pile | 1 |
| Boats | boat | 7 |
| CCA Ridge | CCA ridge | 1 |
| CB Ridge | cinderblock ridge | 1 |

**S1 Table. Reef site configurations in the Rio Grande Valley Reef.**

| CB | miscellaneous cinderblocks | 8 |
| --- | --- | --- |
| CB, Concrete | cinderblock, concrete | 3 |
| CB, Culvert, Concrete | cinderblock, culverts, concrete | 1 |
| CB, Culvert, HD | cinderblock, culverts, highway divider | 1 |
| CB, Culvert, HD, Concrete | cinderblock, culverts, highway divider, concrete | 30 |
| CB, PY | cinderblock, pyramids | 7 |
| CB, WM, Concrete | cinderblock, water mill, concrete | 7 |
| CB, WM, HD, Concrete | cinderblock, water mill, highway divider, concrete | 2 |
| Concrete | concrete | 2 |
| HD, RR | highway divider, rr ties | 16 |
| HD, RR, Concrete | highway divider, rr ties, concrete | 2 |
| Limestone | limestone | 16 |
| Low Profile | low profile | 8 |
| Octoreef | octoreef | 6 |
| RR Ridge | rr tie ridge | 1 |
| RR Ties | rr ties | 3 |
| RR, Concrete | rr ties, concrete | 29 |
| RR, Concrete, CB | rr ties, concrete, cinderblock | 50 |
| RR, Spools | rr ties, spools | 11 |
| RR, Spools, Concrete | rr ties, spools, concrete | 7 |
| RR, Spools, Concrete, Culvert | rr ties, spools, concrete, culverts | 2 |
| RR, Spools, Culvert | rr ties, spools, culverts | 3 |

**S1 Table cont………………….**
