# Supplementary material for: Comparison of fish biomass and fish carbon content associated with reef sites at the Rio Grande Valley artificial reef in the Gulf of Mexico
Source: PLoS One. 2026 Jun 4;21(6):e0350204. doi: 10.1371/journal.pone.0350204 (PMC13235911; doi:10.1371/journal.pone.0350204)
Supplement: S2 Table — (DOCX) [file pone.0350204.s007.docx]

**S2 Table. Model comparison overview for all three models based on spatial groupings of fish.**

| **Feature** | **best_gam**  **(all fish)** | **best_gam20 (≤ 20 m)** | **best_gamaway20**  **(> 20m)** |
| --- | --- | --- | --- |
| n | 806 | 255 | 551 |
| Formula | relief + s(depth) + s(dist_near) + s(dist_boundary) | relief + s(depth) | relief + s(depth) + s(dist_near) + s(dist_boundary) + area |
| Relief category | All terms sig. except “low-mid” | All terms sig. except “low-mid” | Only “low” and “high” terms sig |
| Depth (Avg_depth_m) | *** (edf 3.8)  p = 6.5e-07 | * (edf 2.7)  p = 0.0619 | *** (edf 3.3)  p = 0.000137 |
| Dist to structure | *** (edf 3.1) | NS (excluded in model) | ** (edf 2.97) |
| Dist to boundary | * | NS (excluded) | *** (edf 2.99) |
| Structure area | Not included | Not included | * (positive effect) |
| Adj. R² | 0.197 | 0.107 | 0.2 |
| Deviance explained | 21.4% | 13.1% | 22.1% |
