# Supplementary material for: Comparison of fish biomass and fish carbon content associated with reef sites at the Rio Grande Valley artificial reef in the Gulf of Mexico
Source: PLoS One. 2026 Jun 4;21(6):e0350204. doi: 10.1371/journal.pone.0350204 (PMC13235911; doi:10.1371/journal.pone.0350204)
Supplement: S3 Table — (DOCX) [file pone.0350204.s008.docx]

**S3 Table. Dataset used to calculate average fish biomass by structure type and relief category presented in Figure 3.**

| Site | Structure type | Relief | Fish Biomass (kg) | |  | Figure 3a |  |
| --- | --- | --- | --- | --- | --- | --- | --- |
| Big Pile | Big Pile | high | 2852.40 |  |  | **Row Labels** | **Average of Fish Biomass (kg)** |
| Andy Faskin | Boat | high | 22.95 |  |  | Big Pile | 2852.397155 |
| Billy Kenon | Boat | high | 1155.38 |  |  | Boat | 769.3090542 |
| EMR Captain Bary | Boat | high | 190.14 |  |  | CB | 7.226338756 |
| Murray Meggison | Boat | high | 251.63 |  |  | Concrete | 6.535998837 |
| RGV Shrimper | Boat | high | 130.30 |  |  | Low Profile | 1.734498137 |
| RGV Tug | Boat | high | 3447.47 |  |  | Other/Mixed | 62.31885889 |
| SPI Ceviche | Boat | high | 187.29 |  |  | Pyramid | 27.54891388 |
| 1-6 Block16 | CB | low | 0.84 |  |  | RR | 32.29429435 |
| 1-6 Block19 | CB | low | 3.11 |  |  | **Grand Total** | **65.32937912** |
| 1-8 Block27 | CB | low | 1.82 |  |  |  |  |
| 1-8 Block33 | CB | low | 10.29 |  |  |  |  |
| 1-8 Block36 | CB | low | 8.09 |  |  |  |  |
| 1-8 Block37 | CB | low | 0.91 |  |  | Figure 3b |  |
| 1-8 Block40 and part of patch 80 | CB | low | 33.45 |  |  | **Row Labels** | **Average of Fish Biomass (kg)** |
| 1-8 Block41 | CB | low | 2.05 |  |  | high | 839.8725989 |
| 1-8 Block42 | CB | low | 0.77 |  |  | low | 7.810498629 |
| 1-8 Block44 | CB | low | 1.91 |  |  | low-mid | 8.822806196 |
| 1-8 Block54 | CB | low | 2.14 |  |  | mid | 27.16921008 |
| 1-8 Block63 | CB | low | 4.25 |  |  | mid-high | 58.38350522 |
| 1-8 Block65 | CB | low | 1.18 |  |  | **Grand Total** | **65.32937912** |
| 1-8 Block71 | CB | low | 1.15 |  |  |  |  |
| 30-mixed Block1 | CB | low-mid | 1.89 |  |  |  |  |
| 30-mixed Block3 | CB | low-mid | 26.81 |  |  |  |  |
| 3-4 Block10 | CB | low | 3.32 |  |  |  |  |
| 3-4 block2 | CB | low | 0.69 |  |  |  |  |
| 3-4 Block40 | CB | low | 1.06 |  |  |  |  |
| 3-4 Block5 | CB | low | 9.21 |  |  |  |  |
| 3-4 Block8 | CB | low | 1.15 |  |  |  |  |
| 3-6 Block10 | CB | low | 1.28 |  |  |  |  |
| 3-6 Block2 | CB | low | 0.04 |  |  |  |  |
| 3-6 Block8 | CB | low | 1.03 |  |  |  |  |
| 3-6 Block9 | CB | low | 2.79 |  |  |  |  |
| 3-8 block 14 | CB | low | 6.79 |  |  |  |  |
| 3-8 Block10 | CB | low | 3.53 |  |  |  |  |
| 3-8 Block11 | CB | low | 5.85 |  |  |  |  |
| 3-mixed Block ridge | CB | low | 66.09 |  |  |  |  |
| 3-mixed Block21 | CB | low | 10.48 |  |  |  |  |
| 3-mixed block22 | CB | low | 18.90 |  |  |  |  |
| 3-mixed Block24 | CB | low | 8.44 |  |  |  |  |
| 3-mixed Block27 | CB | low | 4.26 |  |  |  |  |
| 3-mixed Block28 | CB | low | 0.02 |  |  |  |  |
| 3-mixed Block3 | CB | low | 5.05 |  |  |  |  |
| 3-mixed Block30 | CB | low | 0.08 |  |  |  |  |
| 3-mixed Block32 | CB | low | 19.84 |  |  |  |  |
| 3-mixed Block39 | CB | low | 7.26 |  |  |  |  |
| Block UK4 | CB | low | 5.42 |  |  |  |  |
| Block UK5 | CB | low | 5.82 |  |  |  |  |
| Patch 104 | Other/Mixed | low | 47.13 |  |  |  |  |
| Patch 87 | Other/Mixed | low | 60.51 |  |  |  |  |
| Patch 94 | Other/Mixed | mid-high | 10.59 |  |  |  |  |
| Patch 83 | Other/Mixed | mid-high | 11.03 |  |  |  |  |
| Patch 100 | Other/Mixed | mid-high | 77.27 |  |  |  |  |
| Patch 101 | Other/Mixed | mid-high | 2.75 |  |  |  |  |
| Patch 68 | Other/Mixed | mid-high | 1.20 |  |  |  |  |
| Patch 69 | Other/Mixed | mid-high | 6.04 |  |  |  |  |
| Patch 70 | Other/Mixed | mid-high | 5.08 |  |  |  |  |
| Patch 71 | Other/Mixed | mid-high | 13.25 |  |  |  |  |
| Patch 72 | Other/Mixed | mid-high | 13.99 |  |  |  |  |
| Patch 74 | Other/Mixed | mid-high | 23.10 |  |  |  |  |
| Patch 77 | Other/Mixed | mid-high | 32.87 |  |  |  |  |
| Patch 78 | Other/Mixed | mid-high | 3.52 |  |  |  |  |
| Patch 82 | Other/Mixed | mid-high | 1.82 |  |  |  |  |
| Patch 84 | Other/Mixed | mid-high | 11.87 |  |  |  |  |
| Patch 85 | Other/Mixed | mid-high | 7.98 |  |  |  |  |
| Patch 86 | Other/Mixed | mid-high | 1.44 |  |  |  |  |
| Patch 89 | Other/Mixed | mid-high | 22.15 |  |  |  |  |
| Patch 91 | Other/Mixed | mid-high | 10.67 |  |  |  |  |
| Patch 95 | Other/Mixed | mid-high | 0.13 |  |  |  |  |
| Patch 97 | Other/Mixed | mid-high | 5.36 |  |  |  |  |
| Patch 98 | Other/Mixed | mid-high | 6.21 |  |  |  |  |
| Patch 99 | Other/Mixed | mid-high | 119.02 |  |  |  |  |
| Patch 52 | Pyramid | mid | 42.84 |  |  |  |  |
| Patch 55 | Pyramid | mid | 10.67 |  |  |  |  |
| Patch 56 | Pyramid | mid | 5.01 |  |  |  |  |
| Patch 58 | Pyramid | mid | 45.02 |  |  |  |  |
| Patch 60 | Pyramid | mid | 8.13 |  |  |  |  |
| Patch 102 | Other/Mixed | mid-high | 7.73 |  |  |  |  |
| Patch 88 | Other/Mixed | mid-high | 2.48 |  |  |  |  |
| Patch 93 | Other/Mixed | mid-high | 2.23 |  |  |  |  |
| Patch 64 | Other/Mixed | mid-high | 4.31 |  |  |  |  |
| Concrete 10 ton13 | Concrete | low | 6.65 |  |  |  |  |
| Concrete 10 ton4 | Concrete | low | 1.12 |  |  |  |  |
| Concrete 10 ton6 | Concrete | low | 14.55 |  |  |  |  |
| Concrete 10 ton7 | Concrete | low | 36.18 |  |  |  |  |
| Concrete 10 ton8 | Concrete | low | 8.85 |  |  |  |  |
| Concrete 10 ton9 | Concrete | low | 1.99 |  |  |  |  |
| Concrete 2 ton10 | Concrete | low | 6.13 |  |  |  |  |
| Concrete 2 ton11 | Concrete | low | 0.02 |  |  |  |  |
| Concrete 2 ton12 | Concrete | low | 4.13 |  |  |  |  |
| Concrete 2 ton3 | Concrete | low | 5.28 |  |  |  |  |
| Concrete 2 ton6 | Concrete | low | 3.75 |  |  |  |  |
| Concrete 2 ton7 | Concrete | low | 5.02 |  |  |  |  |
| Tile 200 ton1 | Concrete | low | 2.47 |  |  |  |  |
| Hwy Dividers1 | RR | mid | 12.55 |  |  |  |  |
| Hwy Dividers33 | RR | mid | 10.13 |  |  |  |  |
| Hwy Dividers34 and 35 | RR | mid | 4.13 |  |  |  |  |
| Hwy Dividers4 and 8 | RR | mid | 2.87 |  |  |  |  |
| Hwy Dividers5 | RR | mid | 1.66 |  |  |  |  |
| Hwy Dividers6 | RR | mid | 0.91 |  |  |  |  |
| Hwy Dividers11 | RR | mid | 11.98 |  |  |  |  |
| LS-15 | Concrete | low | 1.00 |  |  |  |  |
| LS-2 | Concrete | low | 0.90 |  |  |  |  |
| LPM-2 | Low Profile | low | 0.20 |  |  |  |  |
| Patch 1 | Low Profile | low | 2.66 |  |  |  |  |
| Patch 15 | Low Profile | low | 1.69 |  |  |  |  |
| Patch 22 | Low Profile | low | 0.49 |  |  |  |  |
| Patch 4 | Low Profile | low | 1.53 |  |  |  |  |
| Patch 47 | Low Profile | low | 3.85 |  |  |  |  |
| Patch 10 | Pyramid | mid | 15.60 |  |  |  |  |
| Patch 11 | Pyramid | mid | 80.33 |  |  |  |  |
| Patch 16 | Pyramid | mid | 30.90 |  |  |  |  |
| Patch 18 | Pyramid | mid | 35.71 |  |  |  |  |
| Patch 19 | Pyramid | mid | 6.28 |  |  |  |  |
| Patch 21 | Pyramid | mid | 47.31 |  |  |  |  |
| Patch 25 | Pyramid | mid | 42.13 |  |  |  |  |
| Patch 31 | Pyramid | mid | 6.27 |  |  |  |  |
| Patch 32 | Pyramid | mid | 24.99 |  |  |  |  |
| Patch 35 | Pyramid | mid | 59.74 |  |  |  |  |
| Patch 48 | Pyramid | mid | 35.76 |  |  |  |  |
| Patch 5 | Pyramid | mid | 21.30 |  |  |  |  |
| Patch 51 | Pyramid | mid | 4.58 |  |  |  |  |
| Patch 6 | Pyramid | mid | 19.44 |  |  |  |  |
| Patch 7 | Pyramid | mid | 66.63 |  |  |  |  |
| Octoreefs1 | Pyramid | mid | 2.01 |  |  |  |  |
| Octoreefs2 | Pyramid | mid | 3.60 |  |  |  |  |
| Octoreefs4 | Pyramid | mid | 4.64 |  |  |  |  |
| Patch 14 | Pyramid | mid | 19.87 |  |  |  |  |
| Patch 17 | Pyramid | mid | 50.26 |  |  |  |  |
| Patch 20 | Pyramid | mid | 12.95 |  |  |  |  |
| Patch 24 | Pyramid | mid | 28.07 |  |  |  |  |
| Patch 27 | Pyramid | mid | 18.32 |  |  |  |  |
| Patch 29 | Pyramid | mid | 15.64 |  |  |  |  |
| Patch 3 | Pyramid | mid | 21.21 |  |  |  |  |
| Patch 33 | Pyramid | mid | 65.40 |  |  |  |  |
| Patch 36 | Pyramid | mid | 14.67 |  |  |  |  |
| Patch 41 | Pyramid | mid | 20.61 |  |  |  |  |
| Patch 45 | Pyramid | mid | 9.29 |  |  |  |  |
| Patch 46 | Pyramid | mid | 5.23 |  |  |  |  |
| Patch 53 | Pyramid | mid | 9.03 |  |  |  |  |
| Patch 54 | Pyramid | mid | 0.78 |  |  |  |  |
| Patch 57 | Pyramid | mid | 99.18 |  |  |  |  |
| Patch 59 | Pyramid | mid | 24.56 |  |  |  |  |
| Patch 8 | Pyramid | mid | 68.02 |  |  |  |  |
| 25 | RR | low-mid | 3.30 |  |  |  |  |
| 50 | RR | low-mid | 3.30 |  |  |  |  |
| 200 | RR | mid-high | 13.18 |  |  |  |  |
| 250-1 | RR | mid-high | 181.79 |  |  |  |  |
| 250-10 | RR | mid-high | 28.77 |  |  |  |  |
| 250-11 | RR | mid-high | 21.80 |  |  |  |  |
| 250-13 | RR | mid-high | 0.35 |  |  |  |  |
| 250-18 | RR | mid-high | 26.06 |  |  |  |  |
| 250-19 | RR | mid-high | 6.17 |  |  |  |  |
| 250-2 | RR | mid-high | 72.80 |  |  |  |  |
| 250-20 | RR | mid-high | 1.26 |  |  |  |  |
| 250-21 | RR | mid-high | 17.47 |  |  |  |  |
| 250-22 | RR | mid-high | 6.77 |  |  |  |  |
| 250-3 | RR | mid-high | 102.91 |  |  |  |  |
| 250-4 | RR | mid-high | 105.94 |  |  |  |  |
| 250-5 | RR | mid-high | 158.32 |  |  |  |  |
| 250-6 | RR | mid-high | 16.87 |  |  |  |  |
| 250-7 | RR | mid-high | 10.78 |  |  |  |  |
| 250-8 | RR | mid-high | 31.10 |  |  |  |  |
| 500t_ties1 | RR | high | 106.42 |  |  |  |  |
| 500t_ties2 | RR | high | 54.74 |  |  |  |  |
| CCA Corner | RR | mid | 42.54 |  |  |  |  |
| D-16 | RR | mid | 2.11 |  |  |  |  |
| RR tie ridge | RR | mid | 305.10 |  |  |  |  |
| 100ton_mixed-12 | RR | mid | 7.40 |  |  |  |  |
| 100ton_mixed-13 | RR | mid | 4.81 |  |  |  |  |
| 100ton_mixed-14 | RR | mid | 23.10 |  |  |  |  |
| 100ton_mixed-15 | RR | mid | 14.22 |  |  |  |  |
| 100ton_mixed-16 | RR | mid | 85.08 |  |  |  |  |
| 100ton_mixed-17 | RR | mid | 35.37 |  |  |  |  |
| 100ton_mixed-18 | RR | mid | 143.33 |  |  |  |  |
| 100ton_mixed-22 | RR | mid | 4.53 |  |  |  |  |
| 100ton_mixed-27 | RR | mid | 13.93 |  |  |  |  |
| 100ton_mixed-32 | RR | mid | 2.10 |  |  |  |  |
| 100ton_mixed-33 | RR | mid | 1.01 |  |  |  |  |
| 100ton_mixed-48 | RR | mid | 28.50 |  |  |  |  |
| 100ton_mixed-5 | RR | mid | 19.71 |  |  |  |  |
| 100ton_mixed-6 | RR | mid | 13.43 |  |  |  |  |
| Mix_1 | RR | mid | 22.56 |  |  |  |  |
| Mix_11 | RR | mid | 4.31 |  |  |  |  |
| Mix_12 | RR | mid | 1.30 |  |  |  |  |
| Mix_13 | RR | mid | 8.47 |  |  |  |  |
| Mix_14 | RR | mid | 9.99 |  |  |  |  |
| Mix_16 | RR | mid | 85.78 |  |  |  |  |
| Mix_2 | RR | mid | 47.78 |  |  |  |  |
| Mix_24 | RR | mid | 9.36 |  |  |  |  |
| Mix_25 | RR | mid | 9.47 |  |  |  |  |
| Mix_27 | RR | mid | 18.89 |  |  |  |  |
| Mix_28 | RR | mid | 4.11 |  |  |  |  |
| Mix_29 | RR | mid | 33.30 |  |  |  |  |
| Mix_3 | RR | mid | 142.48 |  |  |  |  |
| Mix_30 | RR | mid | 4.64 |  |  |  |  |
| Mix_31 | RR | mid | 11.73 |  |  |  |  |
| Mix_34 | RR | mid | 0.91 |  |  |  |  |
| Mix_35 | RR | mid | 0.86 |  |  |  |  |
| Mix_36 | RR | mid | 0.84 |  |  |  |  |
| Mix_37 | RR | mid | 0.22 |  |  |  |  |
| Mix_38 | RR | mid | 0.64 |  |  |  |  |
| Mix_39 | RR | mid | 17.33 |  |  |  |  |
| Mix_4 | RR | mid | 15.31 |  |  |  |  |
| Mix_40 | RR | mid | 108.20 |  |  |  |  |
| Mix_41 | RR | mid | 2.00 |  |  |  |  |
| Mix_42 | RR | mid | 18.25 |  |  |  |  |
| Mix_44 | RR | mid | 5.44 |  |  |  |  |
| Mix_45 | RR | mid | 29.50 |  |  |  |  |
| Mix_5 | RR | mid | 18.28 |  |  |  |  |
| Mix_6 | RR | mid | 29.08 |  |  |  |  |
| Mix_7 | RR | mid | 0.41 |  |  |  |  |
| D-10 | Other/Mixed | mid-high | 102.90 |  |  |  |  |
| D-11 | Other/Mixed | mid-high | 143.85 |  |  |  |  |
| D-14 | Other/Mixed | mid-high | 3.31 |  |  |  |  |
| D-15 | Other/Mixed | mid-high | 0.05 |  |  |  |  |
| D-2 | Other/Mixed | mid-high | 43.13745 |  |  |  |  |
| D-20 | Other/Mixed | mid-high | 25.80 |  |  |  |  |
| D-22 | Other/Mixed | mid-high | 69.57 |  |  |  |  |
| D-3 | Other/Mixed | mid-high | 45.02 |  |  |  |  |
| D-8 | Other/Mixed | mid-high | 28.05 |  |  |  |  |
| D-1 | Other/Mixed | mid-high | 98.73 |  |  |  |  |
| D-21 | Other/Mixed | mid-high | 101.59 |  |  |  |  |
| D-23 | Other/Mixed | mid-high | 159.41 |  |  |  |  |
| D-25 | Other/Mixed | mid-high | 184.706 |  |  |  |  |
| D-26 big spool | Other/Mixed | mid-high | 211.14 |  |  |  |  |
| D-19 | Other/Mixed | mid-high | 304.54 |  |  |  |  |
| D-24 | Other/Mixed | mid-high | 776.62 |  |  |  |  |
| D-4 | Other/Mixed | mid-high | 46.93 |  |  |  |  |
| D-5 | Other/Mixed | mid-high | 9.62 |  |  |  |  |
