# Supplementary material for: Comparison of fish biomass and fish carbon content associated with reef sites at the Rio Grande Valley artificial reef in the Gulf of Mexico
Source: PLoS One. 2026 Jun 4;21(6):e0350204. doi: 10.1371/journal.pone.0350204 (PMC13235911; doi:10.1371/journal.pone.0350204)
Supplement: S4 Table — Variables include fish cluster identification number, fish biomass, distance, depth, nearest site, structure characteristics, and values used for all statistical and spatial analyses. (DOCX) [file pone.0350204.s009.docx]

**S4 Table.** **Complete dataset used for statistical and spatial analyses presented in Figures 5-7.** Variables include fish cluster identification number, fish biomass, distance, depth, nearest site, structure characteristics, and values used for all statistical and spatial analyses.

| OBJECTID | CLUSTER_ID | | Weight_g | | Count_fish | Avg_depth_m | | Dist_boundary_m | | Dist_near_site_m | | Near_site | | structure_length_m | | structure_area_m | | structure | | | material | | | material_category | | material_category_number | | | relief_category | | site_age | number_materials | | log_weight | | material2 | | structure2 |
| --- | --- | --- | --- | --- | --- | --- | --- | --- | --- | --- | --- | --- | --- | --- | --- | --- | --- | --- | --- | --- | --- | --- | --- | --- | --- | --- | --- | --- | --- | --- | --- | --- | --- | --- | --- | --- | --- | --- |
| 2 | 1 | | 8054.819 | | 5 | 21.69349 | | 74.95346 | | 52.68512 | | Hwy Dividers 4 and 8 | | 265.8981 | | 3820.08 | | 6 pieces concrete highway divider, 60 tons rr ties | | | highway divider, rr ties | | | highway divider, rr ties | | HD, RR | | | mid | | 9 | 2 | | 9.994026 | | RR Ties | | Boat |
| 3 | 2 | | 1797.778 | | 2 | 21.6844 | | 86.88681 | | 24.91139 | | Hwy Dividers7 | | 94.88432 | | 318.1473 | | 3 pieces concrete highway divider, 60 tons rr ties | | | highway divider, rr ties | | | highway divider, rr ties | | HD, RR | | | mid | | 9 | 2 | | 8.494307 | | RR Ties | | Boat |
| 4 | 3 | | 4744.227 | | 5 | 21.6904 | | 90.44702 | | 31.57959 | | Hwy Dividers7 | | 94.88432 | | 318.1473 | | 3 pieces concrete highway divider, 60 tons rr ties | | | highway divider, rr ties | | | highway divider, rr ties | | HD, RR | | | mid | | 9 | 2 | | 9.464684 | | RR Ties | | Boat |
| 5 | 4 | | 585.3751 | | 2 | 21.81086 | | 107.4447 | | 13.18619 | | Hwy Dividers30 | | 187.8667 | | 1699.327 | | 3 pieces concrete highway divider, 60 tons rr ties | | | highway divider, rr ties | | | highway divider, rr ties | | HD, RR | | | mid | | 9 | 2 | | 7.372253 | | RR Ties | | Boat |
| 6 | 5 | | 10202.91 | | 7 | 21.68537 | | 116.0858 | | 38.91102 | | Block UK3 | | 118.0795 | | 531.1807 | | unknown size pallet of cinderblocks | | | cinderblocks | | | UK pallets cinderblocks | | CB | | | low | | 8 | 1 | | 10.23043 | | Cinderblocks | | Boat |
| 7 | 6 | | 43115.9 | | 9 | 21.70137 | | 129.2849 | | 37.37215 | | Hwy Dividers31 | | 115.1263 | | 771.9108 | | 3 pieces concrete highway divider, 60 tons rr ties | | | highway divider, rr ties | | | highway divider, rr ties | | HD, RR | | | mid | | 9 | 2 | | 11.67165 | | RR Ties | | Boat |
| 8 | 7 | | 16317.78 | | 6 | 21.8766 | | 133.5898 | | 46.82313 | | Hwy Dividers32 | | 150.4288 | | 1266.096 | | 3 pieces concrete highway divider, 60 tons rr ties | | | highway divider, rr ties | | | highway divider, rr ties | | HD, RR | | | mid | | 9 | 2 | | 10.70001 | | RR Ties | | Boat |
| 9 | 8 | | 1883.396 | | 3 | 22.16757 | | 144.6025 | | 36.285 | | Hwy Dividers32 | | 150.4288 | | 1266.096 | | 3 pieces concrete highway divider, 60 tons rr ties | | | highway divider, rr ties | | | highway divider, rr ties | | HD, RR | | | mid | | 9 | 2 | | 8.540832 | | RR Ties | | Boat |
| 10 | 9 | | 1230.914 | | 4 | 22.17457 | | 148.0489 | | 52.62381 | | Hwy Dividers32 | | 150.4288 | | 1266.096 | | 3 pieces concrete highway divider, 60 tons rr ties | | | highway divider, rr ties | | | highway divider, rr ties | | HD, RR | | | mid | | 9 | 2 | | 8.115512 | | RR Ties | | Boat |
| 11 | 10 | | 9448.933 | | 4 | 22.25842 | | 161.1613 | | 67.84342 | | Hwy Dividers33 | | 148.5237 | | 726.2203 | | 3 pieces concrete highway divider, 60 tons rr ties | | | highway divider, rr ties | | | highway divider, rr ties | | HD, RR | | | mid | | 9 | 2 | | 10.15366 | | RR Ties | | Boat |
| 12 | 11 | | 2586.7 | | 3 | 22.3178 | | 169.4132 | | 78.32357 | | Hwy Dividers33 | | 148.5237 | | 726.2203 | | 3 pieces concrete highway divider, 60 tons rr ties | | | highway divider, rr ties | | | highway divider, rr ties | | HD, RR | | | mid | | 9 | 2 | | 8.858138 | | RR Ties | | Boat |
| 13 | 12 | | 527.301 | | 2 | 22.365 | | 172.4859 | | 80.89889 | | Hwy Dividers 10 and 23 | | 287.499 | | 4688.678 | | 6 pieces concrete highway divider, 60 tons rr ties | | | highway divider, rr ties | | | highway divider, rr ties | | HD, RR | | | mid | | 9 | 2 | | 7.267771 | | RR Ties | | Boat |
| 14 | 13 | | 1019.072 | | 2 | 22.37442 | | 177.3942 | | 72.11501 | | Hwy Dividers 10 and 23 | | 287.499 | | 4688.678 | | 6 pieces concrete highway divider, 60 tons rr ties | | | highway divider, rr ties | | | highway divider, rr ties | | HD, RR | | | mid | | 9 | 2 | | 7.926647 | | RR Ties | | Boat |
| 15 | 14 | | 253178.9 | | 28 | 22.81022 | | 232.9726 | | 21.85774 | | 250-20 | | 547.0743 | | 17094.68 | | 250 ton pile rr ties | | | rr ties | | | 250T RR | | 250T RR | | | mid-high | | 6 | 1 | | 13.44185 | | RR Ties | | Boat |
| 16 | 15 | | 20537.41 | | 6 | 22.79143 | | 237.3534 | | 13.21208 | | 250-20 | | 547.0743 | | 17094.68 | | 250 ton pile rr ties | | | rr ties | | | 250T RR | | 250T RR | | | mid-high | | 6 | 1 | | 10.93 | | RR Ties | | Boat |
| 17 | 16 | | 3496.812 | | 2 | 22.80235 | | 243.9664 | | 41.5288 | | 250-20 | | 547.0743 | | 17094.68 | | 250 ton pile rr ties | | | rr ties | | | 250T RR | | 250T RR | | | mid-high | | 6 | 1 | | 9.159607 | | RR Ties | | Boat |
| 18 | 17 | | 5241.854 | | 5 | 22.95098 | | 260.1985 | | 37.71344 | | Patch 86 | | 317.8525 | | 4563.282 | | mixed patch w/ 1 6 in palet cinderblocks  2 8 in palet cinderblocks  2 10x10 box culverts  3 pieces concrete highway divider  low profile chunks of concrete  low profile chunks of concrete | | | cinderblocks, culverts, highway divider, concrete | | | cinderblocks, culverts, highway divider, concrete | | CB, Culvert, HD, Concrete | | | mid-high | | 9 | 4 | | 9.564431 | | Other / Mixed | | RR Ties |
| 19 | 18 | | 13469.05 | | 10 | 23.0109 | | 262.7005 | | 36.22661 | | 250-17 | | 397.0561 | | 10737.79 | | 250 ton pile rr ties | | | rr ties | | | 250T RR | | 250T RR | | | mid-high | | 6 | 1 | | 10.50815 | | RR Ties | | RR Ties |
| 20 | 19 | | 9669.928 | | 3 | 23.31956 | | 161.2687 | | 12.29732 | | Patch 89 | | 246.0308 | | 3705.774 | | mixed patch w/ 2 8 in palet cinderblocks  3 pieces concrete highway divider  5 round culverts  low profile chunks of concrete | | | cinderblocks, culverts, highway divider, concrete | | | cinderblocks, culverts, highway divider, concrete | | CB, Culvert, HD, Concrete | | | mid-high | | 9 | 4 | | 10.17678 | | Other / Mixed | | RR Ties |
| 21 | 20 | | 23756.92 | | 4 | 23.22975 | | 149.2651 | | 16.22194 | | Patch 90 | | 112.6175 | | 860.7581 | | mixed patch w/ 2 8 in palet cinderblocks   low profile chunks of concrete   concrete watermill structure | | | cinderblocks, water mill, concrete | | | cinderblocks, water mill, concrete | | CB, WM, Concrete | | | mid-high | | 9 | 3 | | 11.07563 | | Other / Mixed | | Other / Mixed |
| 22 | 21 | | 726.9971 | | 7 | 23.34628 | | 105.2171 | | 30.39114 | | Patch 88 | | 84.63844 | | 476.493 | | mixed patch w/ 2 8 in palet cinderblocks  low profile chunks of concrete  concrete watermill structure | | | cinderblocks, water mill, concrete | | | cinderblocks, water mill, concrete | | CB, WM, Concrete | | | mid-high | | 9 | 3 | | 7.588922 | | Other / Mixed | | Other / Mixed |
| 23 | 22 | | 74096.56 | | 2 | 23.2949 | | 26.85115 | | 110.6884 | | Murray Meg | | 206.0817 | | 2375.573 | | boat | | | boat | | | boat | | Boats | | | high | | 6 | 1 | | 12.21312 | | Boat | | Other / Mixed |
| 24 | 23 | | 222395.5 | | 634 | 23.23702 | | 116.3917 | | 27.67176 | | Murray Meg | | 206.0817 | | 2375.573 | | boat | | | boat | | | boat | | Boats | | | high | | 6 | 1 | | 13.31221 | | Boat | | Other / Mixed |
| 25 | 24 | | 11918.81 | | 6 | 23.0516 | | 131.5683 | | 19.50668 | | Patch 101 | | 299.1166 | | 4868.212 | | mixed patch w/ 1 6 in palet cinderblocks  2 8 in palet cinderblocks  3 pieces concrete highway divider  5 round culverts  low profile chunks of concrete | | | cinderblocks, culverts, highway divider, concrete | | | cinderblocks, culverts, highway divider, concrete | | CB, Culvert, HD, Concrete | | | mid-high | | 9 | 4 | | 10.38587 | | Other / Mixed | | Other / Mixed |
| 26 | 25 | | 15099.08 | | 6 | 22.99689 | | 52.09887 | | 84.67101 | | Patch 101 | | 299.1166 | | 4868.212 | | mixed patch w/ 1 6 in palet cinderblocks  2 8 in palet cinderblocks  3 pieces concrete highway divider  5 round culverts  low profile chunks of concrete | | | cinderblocks, culverts, highway divider, concrete | | | cinderblocks, culverts, highway divider, concrete | | CB, Culvert, HD, Concrete | | | mid-high | | 9 | 4 | | 10.62239 | | Other / Mixed | | Other / Mixed |
| 27 | 26 | | 3002.983 | | 3 | 22.99069 | | 61.22751 | | 79.95935 | | Patch 101 | | 299.1166 | | 4868.212 | | mixed patch w/ 1 6 in palet cinderblocks  2 8 in palet cinderblocks  3 pieces concrete highway divider  5 round culverts  low profile chunks of concrete | | | cinderblocks, culverts, highway divider, concrete | | | cinderblocks, culverts, highway divider, concrete | | CB, Culvert, HD, Concrete | | | mid-high | | 9 | 4 | | 9.007361 | | Other / Mixed | | Other / Mixed |
| 28 | 27 | | 272894.6 | | 144 | 23.26114 | | 144.5197 | | 5.988165 | | Patch 102 | | 120.6119 | | 660.5829 | | mixed patch w/ 2 8 in palet cinderblocks  low profile chunks of concrete  concrete watermill structure | | | cinderblocks, water mill, concrete | | | cinderblocks, water mill, concrete | | CB, WM, HD, Concrete | | | mid-high | | 9 | 4 | | 13.51684 | | Other / Mixed | | Other / Mixed |
| 29 | 28 | | 10897.36 | | 11 | 23.28267 | | 133.5059 | | 7.866192 | | Patch 91 | | 211.001 | | 2526.413 | | mixed patch w/ 2 8 in palet cinderblocks  2 10x10 box culverts  3 pieces concrete highway divider  low profile chunks of concrete | | | cinderblocks, culverts, highway divider, concrete | | | cinderblocks, culverts, highway divider, concrete | | CB, Culvert, HD, Concrete | | | mid-high | | 9 | 4 | | 10.29628 | | Other / Mixed | | Other / Mixed |
| 30 | 29 | | 4206.115 | | 2 | 23.036 | | 42.59364 | | 96.70431 | | Patch 101 | | 299.1166 | | 4868.212 | | mixed patch w/ 1 6 in palet cinderblocks  2 8 in palet cinderblocks  3 pieces concrete highway divider  5 round culverts  low profile chunks of concrete | | | cinderblocks, culverts, highway divider, concrete | | | cinderblocks, culverts, highway divider, concrete | | CB, Culvert, HD, Concrete | | | mid-high | | 9 | 4 | | 9.344295 | | Other / Mixed | | Other / Mixed |
| 31 | 30 | | 29231.4 | | 4 | 22.98703 | | 79.84274 | | 63.15925 | | Patch 98 | | 175.0049 | | 1736.466 | | mixed patch w/ 1 6 in palet cinderblocks   2 8 in palet cinderblocks   2 10x10 box culverts  3 pieces concrete highway divider  low profile chunks of concrete | | | cinderblocks, culverts, highway divider, concrete | | | cinderblocks, culverts, highway divider, concrete | | CB, Culvert, HD, Concrete | | | mid-high | | 9 | 4 | | 11.283 | | Other / Mixed | | Other / Mixed |
| 32 | 31 | | 23722.09 | | 23 | 23.19638 | | 91.67284 | | 47.83307 | | Patch 102 | | 120.6119 | | 660.5829 | | mixed patch w/ 2 8 in palet cinderblocks  low profile chunks of concrete  concrete watermill structure | | | cinderblocks, water mill, concrete | | | cinderblocks, water mill, concrete | | CB, WM, HD, Concrete | | | mid-high | | 9 | 4 | | 11.07416 | | Other / Mixed | | Other / Mixed |
| 33 | 32 | | 46214.85 | | 12 | 23.1805 | | 107.3441 | | 27.27074 | | Patch 94 | | 173.9244 | | 1804.343 | | mixed patch w/ 1 6 in palet cinderblocks  2 8 in palet cinderblocks  5 round culverts  low profile chunks of concrete  low profile chunks of concrete | | | cinderblocks, culverts, concrete | | | cinderblocks, culverts, concrete | | CB, Culvert, Concrete | | | mid-high | | 9 | 3 | | 11.74106 | | Other / Mixed | | Other / Mixed |
| 34 | 33 | | 10785.39 | | 2 | 23.33348 | | 137.2322 | | 7.042558 | | Patch 94 | | 173.9244 | | 1804.343 | | mixed patch w/ 1 6 in palet cinderblocks  2 8 in palet cinderblocks  5 round culverts  low profile chunks of concrete  low profile chunks of concrete | | | cinderblocks, culverts, concrete | | | cinderblocks, culverts, concrete | | CB, Culvert, Concrete | | | mid-high | | 9 | 3 | | 10.28595 | | Other / Mixed | | Other / Mixed |
| 35 | 34 | | 6973.579 | | 3 | 23.1601 | | 171.1408 | | 28.04351 | | Patch 95 | | 194.3622 | | 2266.444 | | mixed patch w/ 1 6 in palet cinderblocks  2 8 in palet cinderblocks  2 10x10 box culverts  3 pieces concrete highway divider  low profile chunks of concrete | | | cinderblocks, culverts, highway divider, concrete | | | cinderblocks, culverts, highway divider, concrete | | CB, Culvert, HD, Concrete | | | mid-high | | 9 | 4 | | 9.849884 | | Other / Mixed | | Other / Mixed |
| 36 | 35 | | 12656.37 | | 5 | 23.15984 | | 174.7932 | | 1.289343 | | Patch 93 | | 84.33348 | | 335.0815 | | mixed patch w/ 2 8 in palet cinderblocks   low profile chunks of concrete   concrete watermill structure | | | cinderblocks, water mill, concrete | | | cinderblocks, water mill, concrete | | CB, WM, Concrete | | | mid-high | | 9 | 3 | | 10.44592 | | Other / Mixed | | Other / Mixed |
| 37 | 36 | | 2741.9 | | 2 | 23.21056 | | 84.38982 | | 50.68702 | | Patch 94 | | 173.9244 | | 1804.343 | | mixed patch w/ 1 6 in palet cinderblocks  2 8 in palet cinderblocks  5 round culverts  low profile chunks of concrete  low profile chunks of concrete | | | cinderblocks, culverts, concrete | | | cinderblocks, culverts, concrete | | CB, Culvert, Concrete | | | mid-high | | 9 | 3 | | 8.916406 | | Other / Mixed | | Other / Mixed |
| 38 | 37 | | 10049.15 | | 6 | 23.20938 | | 67.98155 | | 71.69474 | | Patch 102 | | 120.6119 | | 660.5829 | | mixed patch w/ 2 8 in palet cinderblocks  low profile chunks of concrete  concrete watermill structure | | | cinderblocks, water mill, concrete | | | cinderblocks, water mill, concrete | | CB, WM, HD, Concrete | | | mid-high | | 9 | 4 | | 10.21524 | | Other / Mixed | | Other / Mixed |
| 39 | 38 | | 29712.94 | | 3 | 23.36762 | | 29.24249 | | 126.9772 | | Patch 99 | | 275.534 | | 4359.957 | | mixed patch w/ 1 6 in palet cinderblocks  2 8 in palet cinderblocks  3 pieces concrete highway divider  5 round culverts  low profile chunks of concrete | | | cinderblocks, culverts, highway divider, concrete | | | cinderblocks, culverts, highway divider, concrete | | CB, Culvert, HD, Concrete | | | mid-high | | 9 | 4 | | 11.29934 | | Other / Mixed | | Other / Mixed |
| 40 | 39 | | 52918.99 | | 2 | 23.16021 | | 177.7039 | | 11.65553 | | Patch 97 | | 276.2059 | | 4294.15 | | mixed patch w/ 2 8 in palet cinderblocks  3 pieces concrete highway divider  5 round culverts  low profile chunks of concrete  low profile chunks of concrete | | | cinderblocks, culverts, highway divider, concrete | | | cinderblocks, culverts, highway divider, concrete | | CB, Culvert, HD, Concrete | | | mid-high | | 9 | 4 | | 11.87652 | | Other / Mixed | | Other / Mixed |
| 41 | 40 | | 2594.946 | | 2 | 22.68488 | | 134.4386 | | 48.10016 | | Hwy Dividers11 | | 121.8719 | | 955.7722 | | 3 pieces concrete highway divider, 60 tons rr ties, 10 tons concrete | | | highway divider, rr ties, concrete | | | highway divider, rr ties, concrete | | HD, RR, Concrete | | | mid | | 9 | 3 | | 8.861321 | | RR Ties | | Other / Mixed |
| 42 | 41 | | 11055.46 | | 2 | 22.46517 | | 129.98 | | 43.8422 | | Hwy Dividers 34 and 35 | | 232.4753 | | 2215.741 | | 6 pieces concrete highway divider, 60 tons rr ties | | | highway divider, rr ties | | | highway divider, rr ties | | HD, RR | | | mid | | 9 | 2 | | 10.31068 | | RR Ties | | Other / Mixed |
| 43 | 42 | | 9173.62 | | 3 | 22.45315 | | 126.5117 | | 27.14887 | | Hwy Dividers 34 and 35 | | 232.4753 | | 2215.741 | | 6 pieces concrete highway divider, 60 tons rr ties | | | highway divider, rr ties | | | highway divider, rr ties | | HD, RR | | | mid | | 9 | 2 | | 10.12409 | | RR Ties | | Other / Mixed |
| 44 | 43 | | 5782.806 | | 4 | 22.57082 | | 123.7303 | | 15.88552 | | Hwy Dividers 10 and 23 | | 287.499 | | 4688.678 | | 6 pieces concrete highway divider, 60 tons rr ties | | | highway divider, rr ties | | | highway divider, rr ties | | HD, RR | | | mid | | 9 | 2 | | 9.662644 | | RR Ties | | Other / Mixed |
| 45 | 44 | | 2370.539 | | 3 | 22.34001 | | 118.8649 | | 25.87421 | | Hwy Dividers 10 and 23 | | 287.499 | | 4688.678 | | 6 pieces concrete highway divider, 60 tons rr ties | | | highway divider, rr ties | | | highway divider, rr ties | | HD, RR | | | mid | | 9 | 2 | | 8.770873 | | RR Ties | | Other / Mixed |
| 46 | 45 | | 4597.973 | | 2 | 21.99096 | | 96.85969 | | 14.16268 | | Hwy Dividers32 | | 150.4288 | | 1266.096 | | 3 pieces concrete highway divider, 60 tons rr ties | | | highway divider, rr ties | | | highway divider, rr ties | | HD, RR | | | mid | | 9 | 2 | | 9.433371 | | RR Ties | | Other / Mixed |
| 47 | 46 | | 42281.02 | | 1527 | 21.8213 | | 95.5072 | | 33.70003 | | Hwy Dividers32 | | 150.4288 | | 1266.096 | | 3 pieces concrete highway divider, 60 tons rr ties | | | highway divider, rr ties | | | highway divider, rr ties | | HD, RR | | | mid | | 9 | 2 | | 11.65209 | | RR Ties | | Other / Mixed |
| 48 | 47 | | 3579.023 | | 2 | 21.79247 | | 80.01283 | | 40.0244 | | Hwy Dividers30 | | 187.8667 | | 1699.327 | | 3 pieces concrete highway divider, 60 tons rr ties | | | highway divider, rr ties | | | highway divider, rr ties | | HD, RR | | | mid | | 9 | 2 | | 9.182845 | | RR Ties | | Other / Mixed |
| 49 | 48 | | 1881.935 | | 2 | 21.7226 | | 81.14255 | | 44.36634 | | Hwy Dividers7 | | 94.88432 | | 318.1473 | | 3 pieces concrete highway divider, 60 tons rr ties | | | highway divider, rr ties | | | highway divider, rr ties | | HD, RR | | | mid | | 9 | 2 | | 8.540056 | | RR Ties | | Other / Mixed |
| 50 | 49 | | 17324.91 | | 4 | 21.54873 | | 225.637 | | 35.17407 | | Patch 76 | | 275.5071 | | 4518.707 | | mixed patch w/ 1 6 in palet cinderblocks  2 8 in palet cinderblocks  2 10x10 box culverts  3 pieces concrete highway divider  low profile chunks of concrete | | | cinderblocks, culverts, highway divider, concrete | | | cinderblocks, culverts, highway divider, concrete | | CB, Culvert, HD, Concrete | | | mid-high | | 9 | 4 | | 10.7599 | | Other / Mixed | | Other / Mixed |
| 51 | 50 | | 15557.68 | | 2 | 21.39906 | | 10.56114 | | 100.1612 | | 500T_1 | | 411.7361 | | 10206.17 | | 500 rr ties | | | rr ties | | | 500T RR | | 500T RR | | | high | | 6 | 1 | | 10.65231 | | RR Ties | | Other / Mixed |
| 52 | 51 | | 75247.42 | | 3 | 21.46667 | | 230.9104 | | 27.06257 | | Block UK4 | | 100.2363 | | 651.2407 | | unknown size pallet of cinderblocks | | | cinderblocks | | | UK pallets cinderblocks | | CB | | | low | | 8 | 1 | | 12.22854 | | Cinderblocks | | Other / Mixed |
| 53 | 52 | | 7761.848 | | 3 | 21.48931 | | 188.8728 | | 52.43131 | | RGV Shrimper | | 217.0793 | | 1568.787 | | boat | | | boat | | | boat | | Boats | | | high | | 10 | 1 | | 9.956976 | | Boat | | Other / Mixed |
| 54 | 53 | | 3163.059 | | 3 | 21.54234 | | 230.7718 | | 48.43571 | | Patch 77 | | 311.3754 | | 5600.178 | | mixed patch w/ 1 6 in palet cinderblocks  2 8 in palet cinderblocks  2 10x10 box culverts  3 pieces concrete highway divider  low profile chunks of concrete | | | cinderblocks, culverts, highway divider, concrete | | | cinderblocks, culverts, highway divider, concrete | | CB, Culvert, HD, Concrete | | | mid-high | | 9 | 4 | | 9.059295 | | Other / Mixed | | Other / Mixed |
| 55 | 54 | | 2908.326 | | 4 | 22.21638 | | 655.427 | | 24.12553 | | 3-8 Block5 | | 181.4628 | | 1507.007 | | 3 8 in palet cinderblocks | | | cinderblocks | | | 3 pallets cinderblocks | | 3CB | | | low | | 8 | 1 | | 8.975333 | | Cinderblocks | | Other / Mixed |
| 56 | 55 | | 20561.46 | | 75 | 22.95845 | | 238.1145 | | 62.27889 | | D-16 | | 527.3876 | | 16841.85 | | Concrete Railroad Ties | | | rr ties | | | RR Ties | | RR Ties | | | mid | | 5 | 1 | | 10.93117 | | RR Ties | | Other / Mixed |
| 57 | 56 | | 160538.4 | | 675 | 23.10888 | | 338.6254 | | 54.55192 | | Big Pile | | 771.4804 | | 34964.52 | | big pile | | | big pile | | | big pile | | Big Pile | | | high | | 8 | 1 | | 12.98629 | | Big Pile | | Other / Mixed |
| 58 | 57 | | 48801.56 | | 41 | 23.09358 | | 201.2303 | | 60.64318 | | D-22 | | 446.7276 | | 12921.52 | | Spools, concrete railroad ties | | | rr ties, spools | | | RR, Spools | | RR, Spools | | | mid-high | | 5 | 2 | | 11.79552 | | Other / Mixed | | Other / Mixed |
| 59 | 58 | | 15932.34 | | 2 | 23.15488 | | 106.5856 | | 37.42915 | | 250-1 | | 403.1612 | | 12376.16 | | 250 ton pile rr ties | | | rr ties | | | 250T RR | | 250T RR | | | mid-high | | 6 | 1 | | 10.67611 | | RR Ties | | Other / Mixed |
| 60 | 59 | | 29646 | | 5 | 23.03497 | | 115.8143 | | 31.88427 | | 250-2 | | 524.274 | | 14719.58 | | 250 ton pile rr ties | | | rr ties | | | 250T RR | | 250T RR | | | mid-high | | 6 | 1 | | 11.29708 | | RR Ties | | Other / Mixed |
| 61 | 60 | | 20392.41 | | 2 | 23.04475 | | 101.8777 | | 47.66967 | | 250-2 | | 524.274 | | 14719.58 | | 250 ton pile rr ties | | | rr ties | | | 250T RR | | 250T RR | | | mid-high | | 6 | 1 | | 10.92292 | | RR Ties | | Other / Mixed |
| 62 | 61 | | 18800.96 | | 9 | 23.07101 | | 212.9817 | | 20.70552 | | Mix_16 | | 338.3012 | | 3776.111 | | 25 ton RR Ties, 25 ton Concrete, 6 Pallets CinderBlock | | | rr ties, concrete, cinderblocks | | | RR, Concrete, CB | | RR, Concrete, CB | | | mid | | 6 | 3 | | 10.84166 | | RR Ties | | Other / Mixed |
| 63 | 62 | | 9887.615 | | 3 | 23.03513 | | 163.347 | | 35.6366 | | Patch 106 | | 177.4416 | | 1670.948 | | mixed patch w/ 2 8 in palet cinderblocks  2 8 in palet cinderblocks  low profile chunks of concrete  concrete watermill structure | | | cinderblocks, water mill, highway divider, concrete | | | cinderblocks, water mill, highway divider, concrete | | CB, WM, HD, Concrete | | | mid-high | | 9 | 4 | | 10.19904 | | Other / Mixed | | Other / Mixed |
| 64 | 63 | | 71286.1 | | 53 | 22.90683 | | 141.9355 | | 15.17264 | | 250-2 | | 524.274 | | 14719.58 | | 250 ton pile rr ties | | | rr ties | | | 250T RR | | 250T RR | | | mid-high | | 6 | 1 | | 12.17446 | | RR Ties | | Other / Mixed |
| 65 | 64 | | 60133.39 | | 8 | 22.97002 | | 134.0655 | | 17.62086 | | 250-2 | | 524.274 | | 14719.58 | | 250 ton pile rr ties | | | rr ties | | | 250T RR | | 250T RR | | | mid-high | | 6 | 1 | | 12.00432 | | RR Ties | | Other / Mixed |
| 66 | 65 | | 3501.825 | | 2 | 23.07611 | | 137.6863 | | 32.90713 | | 250-1 | | 403.1612 | | 12376.16 | | 250 ton pile rr ties | | | rr ties | | | 250T RR | | 250T RR | | | mid-high | | 6 | 1 | | 9.16104 | | RR Ties | | Other / Mixed |
| 67 | 66 | | 32906.64 | | 13 | 23.25526 | | 103.8307 | | 26.66014 | | 250-1 | | 403.1612 | | 12376.16 | | 250 ton pile rr ties | | | rr ties | | | 250T RR | | 250T RR | | | mid-high | | 6 | 1 | | 11.40143 | | RR Ties | | Other / Mixed |
| 68 | 67 | | 44985.29 | | 14 | 22.93328 | | 131.8941 | | 13.02705 | | 250-3 | | 533.8506 | | 12928.94 | | 250 ton pile rr ties | | | rr ties | | | 250T RR | | 250T RR | | | mid-high | | 6 | 1 | | 11.71409 | | RR Ties | | Other / Mixed |
| 69 | 68 | | 18079.51 | | 8 | 23.1377 | | 126.0964 | | 30.10494 | | 250-1 | | 403.1612 | | 12376.16 | | 250 ton pile rr ties | | | rr ties | | | 250T RR | | 250T RR | | | mid-high | | 6 | 1 | | 10.80253 | | RR Ties | | Other / Mixed |
| 70 | 69 | | 3917.706 | | 3 | 22.73685 | | 117.6711 | | 39.1318 | | 250-3 | | 533.8506 | | 12928.94 | | 250 ton pile rr ties | | | rr ties | | | 250T RR | | 250T RR | | | mid-high | | 6 | 1 | | 9.273262 | | RR Ties | | Other / Mixed |
| 71 | 70 | | 16074.02 | | 5 | 22.90861 | | 137.261 | | 21.11939 | | 250-3 | | 533.8506 | | 12928.94 | | 250 ton pile rr ties | | | rr ties | | | 250T RR | | 250T RR | | | mid-high | | 6 | 1 | | 10.68496 | | RR Ties | | Other / Mixed |
| 72 | 71 | | 7907.146 | | 4 | 22.56973 | | 133.2138 | | 34.65165 | | 250-4 | | 412.6682 | | 12133.37 | | 250 ton pile rr ties | | | rr ties | | | 250T RR | | 250T RR | | | mid-high | | 6 | 1 | | 9.975522 | | RR Ties | | Other / Mixed |
| 73 | 72 | | 55941.77 | | 6 | 22.50413 | | 105.4182 | | 34.64675 | | 250-5 | | 449.1978 | | 13227.3 | | 250 ton pile rr ties | | | rr ties | | | 250T RR | | 250T RR | | | mid-high | | 6 | 1 | | 11.93207 | | RR Ties | | Other / Mixed |
| 74 | 73 | | 60745.73 | | 17 | 22.56907 | | 127.6193 | | 6.704041 | | 250-6 | | 420.3532 | | 11466.76 | | 250 ton pile rr ties | | | rr ties | | | 250T RR | | 250T RR | | | mid-high | | 6 | 1 | | 12.01445 | | RR Ties | | Other / Mixed |
| 75 | 74 | | 17212.33 | | 3 | 22.5329 | | 98.3824 | | 28.06516 | | 250-6 | | 420.3532 | | 11466.76 | | 250 ton pile rr ties | | | rr ties | | | 250T RR | | 250T RR | | | mid-high | | 6 | 1 | | 10.75338 | | RR Ties | | Other / Mixed |
| 76 | 75 | | 23616.09 | | 3 | 22.39396 | | 132.5915 | | 33.77395 | | 250-6 | | 420.3532 | | 11466.76 | | 250 ton pile rr ties | | | rr ties | | | 250T RR | | 250T RR | | | mid-high | | 6 | 1 | | 11.06968 | | RR Ties | | Other / Mixed |
| 77 | 76 | | 21546.08 | | 1031 | 19.35636 | | 4.00077 | | 185.1378 | | 3-4 Block1 | | 47.30023 | | 137.8848 | | 3 4 in palet cinderblocks | | | cinderblocks | | | 3 pallets cinderblocks | | 3CB | | | low | | 8 | 1 | | 10.97795 | | Cinderblocks | | Other / Mixed |
| 78 | 77 | | 3389.913 | | 152 | 19.11147 | | 165.6587 | | 57.13281 | | Patch 46 | | 48.74995 | | 136.2459 | | single pyramid | | | pyramid | | | 1PY | | 1PY | | | mid | | 8 | 1 | | 9.12856 | | Pyramids | | Other / Mixed |
| 79 | 78 | | 15078.67 | | 5 | 20.18522 | | 773.3993 | | 63.57977 | | Patch 20 | | 804.1207 | | 27295.57 | | pyramids | | | pyramids | | | 16PY | | 16PY | | | mid | | 8 | 1 | | 10.62104 | | Pyramids | | Other / Mixed |
| 80 | 79 | | 10097.96 | | 5 | 19.98707 | | 586.7247 | | 47.5244 | | Patch 14 | | 44.59184 | | 105.6696 | | single pyramid | | | pyramid | | | 1PY | | 1PY | | | mid | | 8 | 1 | | 10.22009 | | Pyramids | | Other / Mixed |
| 81 | 80 | | 2267.452 | | 11 | 18.98205 | | 227.6407 | | 30.4998 | | Patch 2 | | 40.38134 | | 77.17003 | | single pyramid | | | pyramid | | | 1PY | | 1PY | | | mid | | 8 | 1 | | 8.726412 | | Pyramids | | Other / Mixed |
| 82 | 81 | | 8706.659 | | 4 | 19.61173 | | 196.665 | | 21.04531 | | Patch 36 | | 312.6145 | | 5909.587 | | pyramids | | | pyramids | | | 4PY | | 4PY | | | mid | | 8 | 1 | | 10.07184 | | Pyramids | | Other / Mixed |
| 83 | 82 | | 17398.54 | | 43 | 19.30625 | | 166.7113 | | 87.477 | | Patch 41 | | 628.1935 | | 23819.9 | | pyramids | | | pyramids | | | 16PY | | 16PY | | | mid | | 8 | 1 | | 10.76414 | | Pyramids | | Other / Mixed |
| 84 | 83 | | 63212.81 | | 3013 | 20.43272 | | 518.2298 | | 112.0703 | | Patch 45 | | 249.6864 | | 3879.805 | | pyramids | | | pyramids | | | 4PY | | 4PY | | | mid | | 8 | 1 | | 12.05426 | | Pyramids | | Other / Mixed |
| 85 | 84 | | 12570.25 | | 419 | 20.68838 | | 716.1451 | | 83.13848 | | Patch 57 | | 242.1795 | | 3523.531 | | cinderblocks, pyramids | | | cinderblocks, pyramids | | | cinderblocks, pyramids | | CB, PY | | | mid | | 9 | 2 | | 10.43909 | | Pyramids | | Other / Mixed |
| 86 | 85 | | 5646.332 | | 162 | 20.51627 | | 1019.447 | | 109.9789 | | Patch 58 | | 357.3882 | | 8363.916 | | mixed patch w/ 1 8 in palet cinderblocks  1 8 in palet cinderblocks  4 pyramids | | | cinderblocks, pyramids | | | cinderblocks, pyramids | | CB, PY | | | mid | | 9 | 2 | | 9.638761 | | Pyramids | | Other / Mixed |
| 87 | 86 | | 6440.496 | | 2 | 20.16366 | | 813.5946 | | 103.9315 | | Patch 24 | | 248.2686 | | 3252.476 | | pyramids | | | pyramids | | | 4PY | | 4PY | | | mid | | 8 | 1 | | 9.770361 | | Pyramids | | Other / Mixed |
| 88 | 87 | | 31369.88 | | 1344 | 19.20366 | | 463.7834 | | 109.2846 | | Patch 8 | | 617.3887 | | 25733.57 | | pyramids | | | pyramids | | | 16PY | | 16PY | | | mid | | 8 | 1 | | 11.3536 | | Pyramids | | Other / Mixed |
| 89 | 88 | | 1842.275 | | 23 | 18.50671 | | 232.7427 | | 62.32501 | | Patch 11 | | 608.4765 | | 23407.14 | | mixed; pyramids, lpm | | | pyramids, low profile | | | 16MX | | 16MX | | | mid | | 8 | 2 | | 8.518756 | | Pyramids | | Other / Mixed |
| 90 | 89 | | 1517.68 | | 2 | 19.59907 | | 266.0483 | | 105.1251 | | Patch 3 | | 324.2377 | | 5122.985 | | pyramids | | | pyramids | | | 4PY | | 4PY | | | mid | | 8 | 1 | | 8.324938 | | Pyramids | | Other / Mixed |
| 91 | 90 | | 538.0533 | | 2 | 19.79667 | | 407.3753 | | 29.6206 | | Patch 9 | | 454.9512 | | 13059.44 | | low profile | | | low profile | | | 16LP | | 16LP | | | low | | 8 | 1 | | 7.287958 | | Low Profile | | Other / Mixed |
| 92 | 91 | | 4878.432 | | 4 | 20.73699 | | 569.7204 | | 39.15323 | | Patch 66 | | 329.4691 | | 6463.169 | | mixed patch w/ 1 6 in palet cinderblocks  2 8 in palet cinderblocks  2 10x10 box culverts  3 pieces concrete highway divider  low profile chunks of concrete | | | cinderblocks, culverts, highway divider, concrete | | | cinderblocks, culverts, highway divider, concrete | | CB, Culvert, HD, Concrete | | | mid-high | | 9 | 4 | | 9.492579 | | Other / Mixed | | Other / Mixed |
| 93 | 92 | | 1777.793 | | 2 | 20.69046 | | 259.1709 | | 79.48654 | | Patch 64 | | 284.3085 | | 4381.324 | | mixed patch w/ 2 8 in palet cinderblocks  3 pieces concrete highway divider  low profile chunks of concrete  concrete watermill structure  concrete watermill structure | | | cinderblocks, water mill, highway divider, concrete | | | cinderblocks, water mill, highway divider, concrete | | CB, WM, HD, Concrete | | | mid-high | | 9 | 4 | | 8.483128 | | Other / Mixed | | Other / Mixed |
| 94 | 93 | | 4465.25 | | 2 | 18.93042 | | 61.11458 | | 153.0902 | | Patch 6 | | 163.488 | | 1152.274 | | mixed; pyramids, lpm | | | pyramids, low profile | | | 1MX | | 1MX | | | mid | | 8 | 2 | | 9.40408 | | Pyramids | | Other / Mixed |
| 95 | 94 | | 32086.06 | | 1398 | 19.16465 | | 485.0248 | | 84.42073 | | Patch 18 | | 658.7559 | | 29101.67 | | mixed; pyramids, lpm | | | pyramids, low profile | | | 16MX | | 16MX | | | mid | | 8 | 2 | | 11.37618 | | Pyramids | | Other / Mixed |
| 96 | 95 | | 3678.583 | | 8 | 20.39402 | | 137.6736 | | 104.5474 | | Patch 64 | | 284.3085 | | 4381.324 | | mixed patch w/ 2 8 in palet cinderblocks  3 pieces concrete highway divider  low profile chunks of concrete  concrete watermill structure  concrete watermill structure | | | cinderblocks, water mill, highway divider, concrete | | | cinderblocks, water mill, highway divider, concrete | | CB, WM, HD, Concrete | | | mid-high | | 9 | 4 | | 9.210283 | | Other / Mixed | | Other / Mixed |
| 97 | 96 | | 2500.352 | | 12 | 20.66393 | | 157.5188 | | 48.26734 | | Patch 64 | | 284.3085 | | 4381.324 | | mixed patch w/ 2 8 in palet cinderblocks  3 pieces concrete highway divider  low profile chunks of concrete  concrete watermill structure  concrete watermill structure | | | cinderblocks, water mill, highway divider, concrete | | | cinderblocks, water mill, highway divider, concrete | | CB, WM, HD, Concrete | | | mid-high | | 9 | 4 | | 8.824187 | | Other / Mixed | | Other / Mixed |
| 98 | 97 | | 447.4081 | | 3 | 20.6545 | | 671.444 | | 85.6961 | | Patch 67 | | 339.7311 | | 8294.548 | | mixed patch w/ 1 6 in palet cinderblocks  2 8 in palet cinderblocks  2 10x10 box culverts  3 pieces concrete highway divider  low profile chunks of concrete | | | cinderblocks, culverts, highway divider, concrete | | | cinderblocks, culverts, highway divider, concrete | | CB, Culvert, HD, Concrete | | | mid-high | | 9 | 4 | | 7.103471 | | Other / Mixed | | Other / Mixed |
| 99 | 98 | | 21404.79 | | 4 | 19.93896 | | 581.2188 | | 19.12649 | | Patch 33 | | 188.9853 | | 1770.835 | | pyramids | | | pyramids | | | 4PY | | 4PY | | | mid | | 8 | 1 | | 10.97137 | | Pyramids | | Other / Mixed |
| 100 | 99 | | 11159.97 | | 36 | 20.16805 | | 67.22347 | | 32.23929 | | 3-4 Block2 | | 81.21312 | | 364.3771 | | 3 4 in palet cinderblocks | | | cinderblocks | | | 3 pallets cinderblocks | | 3CB | | | low | | 8 | 1 | | 10.32009 | | Cinderblocks | | Other / Mixed |
| 101 | 100 | | 3692.652 | | 2 | 19.18818 | | 137.6163 | | 89.94854 | | 3-4 Block1 | | 47.30023 | | 137.8848 | | 3 4 in palet cinderblocks | | | cinderblocks | | | 3 pallets cinderblocks | | 3CB | | | low | | 8 | 1 | | 9.2141 | | Cinderblocks | | RR Ties |
| 102 | 101 | | 5584.415 | | 3 | 19.07694 | | 183.6002 | | 72.87936 | | Patch 46 | | 48.74995 | | 136.2459 | | single pyramid | | | pyramid | | | 1PY | | 1PY | | | mid | | 8 | 1 | | 9.627735 | | Pyramids | | RR Ties |
| 103 | 102 | | 2374.554 | | 38 | 19.18336 | | 23.08186 | | 188.6841 | | Patch 21 | | 721.9896 | | 30699.53 | | mixed; pyramids, lpm | | | pyramids, low profile | | | 16MX | | 16MX | | | mid | | 8 | 2 | | 8.772565 | | Pyramids | | RR Ties |
| 104 | 103 | | 14668.79 | | 627 | 19.48249 | | 70.15417 | | 144.1894 | | Patch 16 | | 409.546 | | 6681.443 | | mixed; pyramids, lpm | | | pyramids, low profile | | | 4MX | | 4MX | | | mid | | 8 | 2 | | 10.59348 | | Pyramids | | RR Ties |
| 105 | 104 | | 73398.8 | | 12 | 23.07354 | | 323.4841 | | 46.38826 | | Big Pile | | 771.4804 | | 34964.52 | | big pile | | | big pile | | | big pile | | Big Pile | | | high | | 8 | 1 | | 12.20366 | | Big Pile | | RR Ties |
| 106 | 105 | | 1694.074 | | 2 | 22.9943 | | 336.5961 | | 43.28604 | | Big Pile | | 771.4804 | | 34964.52 | | big pile | | | big pile | | | big pile | | Big Pile | | | high | | 8 | 1 | | 8.434892 | | Big Pile | | RR Ties |
| 107 | 106 | | 382939.1 | | 14443 | 23.15079 | | 269.4531 | | 32.45484 | | Big Pile | | 771.4804 | | 34964.52 | | big pile | | | big pile | | | big pile | | Big Pile | | | high | | 8 | 1 | | 13.85563 | | Big Pile | | RR Ties |
| 108 | 107 | | 30254.61 | | 7 | 23.18702 | | 155.6589 | | 29.57487 | | D-20 | | 455.1934 | | 10831.77 | | Spools, concrete railroad ties | | | rr ties, spools, concrete | | | RR, Spools, Concrete | | RR, Spools, Concrete | | | mid-high | | 5 | 3 | | 11.3174 | | Other / Mixed | | RR Ties |
| 109 | 108 | | 174711.7 | | 34 | 23.24285 | | 112.9677 | | 30.55722 | | D-21 | | 566.7995 | | 13511.86 | | Spools, concrete railroad ties | | | rr ties, spools, concrete | | | RR, Spools, Concrete | | RR, Spools, Concrete | | | mid-high | | 5 | 3 | | 13.07089 | | Other / Mixed | | RR Ties |
| 110 | 109 | | 164101.8 | | 2 | 22.68858 | | 420.3397 | | 31.08507 | | D-10 | | 898.0723 | | 48165.23 | | 1 large spool 300 ties | | | rr ties, spools | | | RR, Spools | | RR, Spools | | | mid-high | | 5 | 2 | | 13.00824 | | Other / Mixed | | RR Ties |
| 111 | 110 | | 76794.28 | | 563 | 23.06091 | | 257.7498 | | 57.67362 | | D-22 | | 446.7276 | | 12921.52 | | Spools, concrete railroad ties | | | rr ties, spools | | | RR, Spools | | RR, Spools | | | mid-high | | 5 | 2 | | 12.24889 | | Other / Mixed | | RR Ties |
| 112 | 111 | | 704.3783 | | 21 | 23.09736 | | 267.916 | | 46.99854 | | D-23 | | 821.8087 | | 31780.67 | | Spools, broken concrete | | | rr ties, spools, concrete | | | RR, Spools, Concrete | | RR, Spools, Concrete | | | mid-high | | 5 | 3 | | 7.557316 | | Other / Mixed | | RR Ties |
| 113 | 112 | | 4343.889 | | 2 | 23.11756 | | 278.8044 | | 46.9891 | | D-23 | | 821.8087 | | 31780.67 | | Spools, broken concrete | | | rr ties, spools, concrete | | | RR, Spools, Concrete | | RR, Spools, Concrete | | | mid-high | | 5 | 3 | | 9.376525 | | Other / Mixed | | RR Ties |
| 114 | 113 | | 3976.118 | | 2 | 22.73012 | | 400.5242 | | 29.52673 | | D-10 | | 898.0723 | | 48165.23 | | 1 large spool 300 ties | | | rr ties, spools | | | RR, Spools | | RR, Spools | | | mid-high | | 5 | 2 | | 9.288061 | | Other / Mixed | | RR Ties |
| 115 | 114 | | 4172.59 | | 2 | 22.84347 | | 368.9451 | | 38.06905 | | D-10 | | 898.0723 | | 48165.23 | | 1 large spool 300 ties | | | rr ties, spools | | | RR, Spools | | RR, Spools | | | mid-high | | 5 | 2 | | 9.336292 | | Other / Mixed | | RR Ties |
| 116 | 115 | | 293.7421 | | 2 | 22.54459 | | 529.8249 | | 54.33064 | | D-11 | | 810.1454 | | 38470.51 | | 1 large spool 300 ties | | | rr ties, spools | | | RR, Spools | | RR, Spools | | | mid-high | | 5 | 2 | | 6.682702 | | Other / Mixed | | RR Ties |
| 117 | 116 | | 44497.72 | | 2 | 22.69814 | | 424.8896 | | 20.00123 | | D-10 | | 898.0723 | | 48165.23 | | 1 large spool 300 ties | | | rr ties, spools | | | RR, Spools | | RR, Spools | | | mid-high | | 5 | 2 | | 11.70319 | | Other / Mixed | | RR Ties |
| 118 | 117 | | 7455.672 | | 2 | 23.00261 | | 244.939 | | 12.2161 | | D-8 | | 779.046 | | 37066.62 | | railroadties, Largest Spool Medium Spool | | | rr ties, spools | | | RR, Spools | | RR, Spools | | | mid-high | | 5 | 2 | | 9.91673 | | Other / Mixed | | RR Ties |
| 119 | 118 | | 10766.34 | | 3 | 22.86668 | | 312.1642 | | 18.84737 | | D-4 | | 843.0299 | | 33942.2 | | Large Spool, ties, culverts | | | rr ties, spools, culverts | | | RR, Spools, Culvert | | RR, Spools, Culvert | | | mid-high | | 5 | 3 | | 10.28418 | | Other / Mixed | | RR Ties |
| 120 | 119 | | 26999.84 | | 3 | 22.82067 | | 283.0495 | | 37.75554 | | D-4 | | 843.0299 | | 33942.2 | | Large Spool, ties, culverts | | | rr ties, spools, culverts | | | RR, Spools, Culvert | | RR, Spools, Culvert | | | mid-high | | 5 | 3 | | 11.20359 | | Other / Mixed | | RR Ties |
| 121 | 120 | | 309675 | | 6 | 22.91376 | | 250.7882 | | 33.53065 | | D-5 | | 945.796 | | 41554.73 | | Large Spool, ties, culverts | | | rr ties, spools, culverts | | | RR, Spools, Culvert | | RR, Spools, Culvert | | | mid-high | | 5 | 3 | | 13.64328 | | Other / Mixed | | RR Ties |
| 122 | 121 | | 26294.49 | | 584 | 21.68088 | | 860.2147 | | 53.50055 | | 67.1 | | 274.4974 | | 5166.366 | | 67 rr tie | | | rr ties | | | 67 rr tie | | 67T RR | | | low-mid | | 8 | 1 | | 11.17711 | | RR Ties | | RR Ties |
| 123 | 122 | | 20181.06 | | 9 | 21.66571 | | 868.9391 | | 63.50397 | | SPI Ceviche | | 171.3348 | | 1110.387 | | boat | | | boat | | | boat | | Boats | | | high | | 5 | 1 | | 10.9125 | | Boat | | RR Ties |
| 124 | 123 | | 170.6918 | | 2 | 21.85576 | | 677.7375 | | 17.37736 | | 3-mixed Block38 | | 107.2563 | | 600.9986 | | 3 pallets mixed size ciderblocks | | | cinderblocks | | | 3 pallets cinderblocks | | 3CB | | | low | | 8 | 1 | | 6.139859 | | Cinderblocks | | RR Ties |
| 125 | 124 | | 428.5147 | | 2 | 22.09807 | | 732.9749 | | 39.59612 | | 3-4 Block7 | | 73.60568 | | 275.3223 | | 3 4 in palet cinderblocks | | | cinderblocks | | | 3 pallets cinderblocks | | 3CB | | | low | | 8 | 1 | | 7.060325 | | Cinderblocks | | RR Ties |
| 126 | 125 | | 755.9186 | | 2 | 22.0318 | | 710.9747 | | 66.29523 | | 3-8 Block15 | | 140.2468 | | 835.0621 | | 3 8 in palet cinderblocks | | | cinderblocks | | | 3 pallets cinderblocks | | 3CB | | | low | | 8 | 1 | | 7.627934 | | Cinderblocks | | RR Ties |
| 127 | 126 | | 1223.643 | | 5 | 21.99238 | | 729.2064 | | 64.34609 | | EMR Capt Berry | | 205.433 | | 2309.324 | | boat | | | boat | | | boat | | Boats | | | high | | 6 | 1 | | 8.109588 | | Boat | | RR Ties |
| 128 | 127 | | 21708.69 | | 7 | 21.89573 | | 846.6121 | | 13.94823 | | 67.1 | | 274.4974 | | 5166.366 | | 67 rr tie | | | rr ties | | | 67 rr tie | | 67T RR | | | low-mid | | 8 | 1 | | 10.98547 | | RR Ties | | RR Ties |
| 129 | 128 | | 40098.22 | | 26 | 21.64663 | | 840.9918 | | 46.36454 | | SPI Ceviche | | 171.3348 | | 1110.387 | | boat | | | boat | | | boat | | Boats | | | high | | 5 | 1 | | 11.59909 | | Boat | | RR Ties |
| 130 | 129 | | 4934.909 | | 2 | 21.85619 | | 839.8601 | | 49.51104 | | 67.1 | | 274.4974 | | 5166.366 | | 67 rr tie | | | rr ties | | | 67 rr tie | | 67T RR | | | low-mid | | 8 | 1 | | 9.50409 | | RR Ties | | RR Ties |
| 131 | 130 | | 56702.43 | | 11 | 22.32309 | | 763.4017 | | 27.12253 | | 67.2 | | 309.6253 | | 5393.584 | | 67 rr tie | | | rr ties | | | 67 rr tie | | 67T RR | | | low-mid | | 8 | 1 | | 11.94557 | | RR Ties | | RR Ties |
| 132 | 131 | | 7289.725 | | 2 | 21.65602 | | 795.0786 | | 39.97929 | | D-27 | | 1459.357 | | 75504.47 | | Spools, culverts and railroad ties | | | rr ties, spools, culverts | | | RR, Spools, Culvert | | RR, Spools, Culvert | | | mid-high | | 5 | 3 | | 9.894221 | | Other / Mixed | | RR Ties |
| 133 | 132 | | 3513.565 | | 50 | 21.78773 | | 736.3909 | | 75.17559 | | 3-mixed Block38 | | 107.2563 | | 600.9986 | | 3 pallets mixed size ciderblocks | | | cinderblocks | | | 3 pallets cinderblocks | | 3CB | | | low | | 8 | 1 | | 9.164387 | | Cinderblocks | | RR Ties |
| 134 | 133 | | 29678.54 | | 3 | 22.26358 | | 813.6409 | | 34.90318 | | 25.1 | | 288.2691 | | 3637.474 | | 25 rr tie | | | rr ties | | | 25 rr tie | | 25T RR | | | low-mid | | 8 | 1 | | 11.29818 | | RR Ties | | RR Ties |
| 135 | 134 | | 10857.5 | | 16 | 21.79712 | | 880.3271 | | 65.82613 | | SPI Ceviche | | 171.3348 | | 1110.387 | | boat | | | boat | | | boat | | Boats | | | high | | 5 | 1 | | 10.29261 | | Boat | | RR Ties |
| 136 | 135 | | 20138.36 | | 15 | 21.62304 | | 812.8725 | | 31.38538 | | SPI Ceviche | | 171.3348 | | 1110.387 | | boat | | | boat | | | boat | | Boats | | | high | | 5 | 1 | | 10.91038 | | Boat | | RR Ties |
| 137 | 136 | | 4401.579 | | 4 | 22.72011 | | 555.6604 | | 62.23794 | | 3-6 Block5 | | 42.86508 | | 123.3765 | | 3 6 in palet cinderblocks | | | cinderblocks | | | 3 pallets cinderblocks | | 3CB | | | low | | 8 | 1 | | 9.389719 | | Cinderblocks | | RR Ties |
| 138 | 137 | | 2906.386 | | 2 | 23.32114 | | 29.86329 | | 103.535 | | Patch 91 | | 211.001 | | 2526.413 | | mixed patch w/ 2 8 in palet cinderblocks  2 10x10 box culverts  3 pieces concrete highway divider  low profile chunks of concrete | | | cinderblocks, culverts, highway divider, concrete | | | cinderblocks, culverts, highway divider, concrete | | CB, Culvert, HD, Concrete | | | mid-high | | 9 | 4 | | 8.974666 | | Other / Mixed | | RR Ties |
| 139 | 138 | | 7057.143 | | 4 | 21.8088 | | 832.7754 | | 18.09637 | | Concrete10 ton8 | | 85.45058 | | 470.6305 | | 10 ton concrete | | | concrete | | | 10 ton concrete | | 10T Concrete | | | low | | 8 | 1 | | 9.861796 | | Concrete | | RR Ties |
| 140 | 139 | | 786.6036 | | 2 | 21.86342 | | 827.6151 | | 24.27827 | | Concrete10 ton8 | | 85.45058 | | 470.6305 | | 10 ton concrete | | | concrete | | | 10 ton concrete | | 10T Concrete | | | low | | 8 | 1 | | 7.667724 | | Concrete | | RR Ties |
| 141 | 140 | | 4313.709 | | 4 | 21.85948 | | 727.26 | | 36.39143 | | Billy Kenon | | 134.7889 | | 934.4976 | | boat | | | boat | | | boat | | Boats | | | high | | 5 | 1 | | 9.369553 | | Boat | | RR Ties |
| 142 | 141 | | 46522.68 | | 143 | 21.80369 | | 682.631 | | 63.28104 | | Billy Kenon | | 134.7889 | | 934.4976 | | boat | | | boat | | | boat | | Boats | | | high | | 5 | 1 | | 11.7477 | | Boat | | RR Ties |
| 143 | 142 | | 28735.3 | | 15 | 21.69472 | | 824.4614 | | 21.63689 | | Concrete10 ton10 | | 83.76552 | | 459.9229 | | 10 ton concrete | | | concrete | | | 10 ton concrete | | 10T Concrete | | | low | | 8 | 1 | | 11.26588 | | Concrete | | RR Ties |
| 144 | 143 | | 223638.7 | | 163 | 21.57223 | | 545.8807 | | 14.17096 | | 100tonmixed18 | | 310.5831 | | 5094.639 | | 75 ton ties, 25 tons broken concrete | | | rr ties, concrete | | | RR, Concrete | | RR, Concrete | | | mid | | 6 | 2 | | 13.31779 | | RR Ties | | RR Ties |
| 145 | 144 | | 1810.409 | | 2 | 20.86228 | | 813.8068 | | 36.8305 | | Patch 69 | | 458.0803 | | 11033.35 | | mixed patch w/ 1 6 in palet cinderblocks  2 8 in palet cinderblocks  2 10x10 box culverts  3 pieces concrete highway divider  low profile chunks of concrete | | | cinderblocks, culverts, highway divider, concrete | | | cinderblocks, culverts, highway divider, concrete | | CB, Culvert, HD, Concrete | | | mid-high | | 9 | 4 | | 8.501308 | | Other / Mixed | | RR Ties |
| 146 | 145 | | 8192.462 | | 2 | 22.57499 | | 276.3703 | | 18.43453 | | 100tonmixed23 | | 381.0174 | | 8729.635 | | 75 ton ties, 25 tons broken concrete | | | rr ties, concrete | | | RR, Concrete | | RR, Concrete | | | mid | | 6 | 2 | | 10.01097 | | RR Ties | | RR Ties |
| 147 | 146 | | 24865.91 | | 151 | 23.08107 | | 141.6561 | | 31.03278 | | Mix_40 | | 353.9422 | | 5332.888 | | 25 ton RR Ties, 25 ton Concrete, 6 Pallets CinderBlock | | | rr ties, concrete, cinderblocks | | | RR, Concrete, CB | | RR, Concrete, CB | | | mid | | 6 | 3 | | 11.12125 | | RR Ties | | RR Ties |
| 148 | 147 | | 1485.837 | | 3 | 23.14486 | | 161.6971 | | 37.7183 | | Patch 106 | | 177.4416 | | 1670.948 | | mixed patch w/ 2 8 in palet cinderblocks  2 8 in palet cinderblocks  low profile chunks of concrete  concrete watermill structure | | | cinderblocks, water mill, highway divider, concrete | | | cinderblocks, water mill, highway divider, concrete | | CB, WM, HD, Concrete | | | mid-high | | 9 | 4 | | 8.303733 | | Other / Mixed | | RR Ties |
| 149 | 148 | | 6720.729 | | 4 | 23.11826 | | 101.1559 | | 49.0968 | | Patch 106 | | 177.4416 | | 1670.948 | | mixed patch w/ 2 8 in palet cinderblocks  2 8 in palet cinderblocks  low profile chunks of concrete  concrete watermill structure | | | cinderblocks, water mill, highway divider, concrete | | | cinderblocks, water mill, highway divider, concrete | | CB, WM, HD, Concrete | | | mid-high | | 9 | 4 | | 9.812952 | | Other / Mixed | | RR Ties |
| 150 | 149 | | 41396.3 | | 131 | 23.08824 | | 181.9226 | | 22.91431 | | Mix_16 | | 338.3012 | | 3776.111 | | 25 ton RR Ties, 25 ton Concrete, 6 Pallets CinderBlock | | | rr ties, concrete, cinderblocks | | | RR, Concrete, CB | | RR, Concrete, CB | | | mid | | 6 | 3 | | 11.63095 | | RR Ties | | RR Ties |
| 151 | 150 | | 3454.783 | | 4 | 21.60342 | | 122.7611 | | 23.7365 | | 250-10 | | 478.9589 | | 10907.67 | | 250 ton pile rr ties | | | rr ties | | | 250T RR | | 250T RR | | | mid-high | | 6 | 1 | | 9.147515 | | RR Ties | | RR Ties |
| 152 | 151 | | 119912.2 | | 59 | 23.28833 | | 57.97038 | | 72.98208 | | 250-1 | | 403.1612 | | 12376.16 | | 250 ton pile rr ties | | | rr ties | | | 250T RR | | 250T RR | | | mid-high | | 6 | 1 | | 12.69452 | | RR Ties | | RR Ties |
| 153 | 152 | | 33199.4 | | 22 | 23.30464 | | 46.18629 | | 97.09172 | | 250-1 | | 403.1612 | | 12376.16 | | 250 ton pile rr ties | | | rr ties | | | 250T RR | | 250T RR | | | mid-high | | 6 | 1 | | 11.41029 | | RR Ties | | RR Ties |
| 154 | 153 | | 18302.15 | | 6 | 23.09816 | | 112.0163 | | 50.26007 | | Patch 106 | | 177.4416 | | 1670.948 | | mixed patch w/ 2 8 in palet cinderblocks  2 8 in palet cinderblocks  low profile chunks of concrete  concrete watermill structure | | | cinderblocks, water mill, highway divider, concrete | | | cinderblocks, water mill, highway divider, concrete | | CB, WM, HD, Concrete | | | mid-high | | 9 | 4 | | 10.81477 | | Other / Mixed | | RR Ties |
| 155 | 154 | | 11454.09 | | 4 | 23.09318 | | 115.3361 | | 42.2687 | | 250-1 | | 403.1612 | | 12376.16 | | 250 ton pile rr ties | | | rr ties | | | 250T RR | | 250T RR | | | mid-high | | 6 | 1 | | 10.3461 | | RR Ties | | RR Ties |
| 156 | 155 | | 184447.3 | | 14 | 22.95213 | | 173.0394 | | 27.24778 | | 250-2 | | 524.274 | | 14719.58 | | 250 ton pile rr ties | | | rr ties | | | 250T RR | | 250T RR | | | mid-high | | 6 | 1 | | 13.12512 | | RR Ties | | RR Ties |
| 157 | 156 | | 4652.906 | | 2 | 22.88293 | | 246.7083 | | 20.42648 | | Mix_39 | | 191.8251 | | 1821.718 | | 25 ton RR Ties, 25 ton Concrete, 6 Pallets CinderBlock | | | rr ties, concrete, cinderblocks | | | RR, Concrete, CB | | RR, Concrete, CB | | | mid | | 6 | 3 | | 9.445247 | | RR Ties | | RR Ties |
| 158 | 157 | | 92713.52 | | 842 | 20.7573 | | 781.6825 | | 20.72693 | | Andy Faskin | | 87.86762 | | 431.0793 | | boat | | | boat | | | boat | | Boats | | | high | | 5 | 1 | | 12.43727 | | Boat | | RR Ties |
| 159 | 158 | | 2378.077 | | 2 | 19.30039 | | 427.2631 | | 54.26527 | | Patch 27 | | 713.1051 | | 24736.61 | | pyramids | | | pyramids | | | 16PY | | 16PY | | | mid | | 8 | 1 | | 8.774048 | | Pyramids | | RR Ties |
| 160 | 159 | | 48269.24 | | 43 | 20.90012 | | 1022.773 | | 21.7814 | | Patch 58 | | 357.3882 | | 8363.916 | | mixed patch w/ 1 8 in palet cinderblocks  1 8 in palet cinderblocks  4 pyramids | | | cinderblocks, pyramids | | | cinderblocks, pyramids | | CB, PY | | | mid | | 9 | 2 | | 11.78455 | | Pyramids | | RR Ties |
| 161 | 160 | | 70850.42 | | 2 | 18.41261 | | 212.4721 | | 57.79421 | | Patch 6 | | 163.488 | | 1152.274 | | mixed; pyramids, lpm | | | pyramids, low profile | | | 1MX | | 1MX | | | mid | | 8 | 2 | | 12.16833 | | Pyramids | | RR Ties |
| 162 | 161 | | 2193.035 | | 3 | 20.73973 | | 214.1451 | | 40.85432 | | Patch 53 | | 329.6576 | | 6980.813 | | pyramids | | | pyramids | | | 4PY | | 4PY | | | mid | | 8 | 1 | | 8.693042 | | Pyramids | | RR Ties |
| 163 | 162 | | 66.23933 | | 2 | 22.63691 | | 604.5848 | | 26.71117 | | d-26 big spool | | 860.7689 | | 30331.27 | | Biggest spool with bouy and broken concrete | | | spools, concrete | | | rr ties, spools, concrete | | RR, Spools, Concrete | | | mid-high | | 5 | 3 | | 5.193274 | | Other / Mixed | | RR Ties |
| 164 | 163 | | 219487.9 | | 411 | 22.73045 | | 563.6271 | | 36.6129 | | d-26 big spool | | 860.7689 | | 30331.27 | | Biggest spool with bouy and broken concrete | | | spools, concrete | | | rr ties, spools, concrete | | RR, Spools, Concrete | | | mid-high | | 5 | 3 | | 13.29905 | | Other / Mixed | | RR Ties |
| 165 | 164 | | 45707.53 | | 8 | 23.02034 | | 201.528 | | 34.03737 | | D-8 | | 779.046 | | 37066.62 | | railroadties, Largest Spool Medium Spool | | | rr ties, spools | | | RR, Spools | | RR, Spools | | | mid-high | | 5 | 2 | | 11.73002 | | Other / Mixed | | RR Ties |
| 166 | 165 | | 1459.946 | | 7 | 23.00085 | | 181.1785 | | 52.47847 | | D-8 | | 779.046 | | 37066.62 | | railroadties, Largest Spool Medium Spool | | | rr ties, spools | | | RR, Spools | | RR, Spools | | | mid-high | | 5 | 2 | | 8.286155 | | Other / Mixed | | RR Ties |
| 167 | 166 | | 1287.231 | | 2 | 22.16194 | | 60.04794 | | 77.01119 | | 250-8 | | 435.2568 | | 12347.32 | | 250 ton pile rr ties | | | rr ties | | | 250T RR | | 250T RR | | | mid-high | | 6 | 1 | | 8.160249 | | RR Ties | | RR Ties |
| 168 | 167 | | 3545.199 | | 2 | 20.84772 | | 691.3744 | | 26.97354 | | Octoreef2 | | 27.38307 | | 47.99176 | | octoreef | | | octoreef | | | octoreef | | Octoreef | | | mid | | 9 | 1 | | 9.17335 | | Pyramids | | RR Ties |
| 169 | 168 | | 37768.15 | | 86 | 20.74204 | | 763.6277 | | 27.33704 | | Andy Faskin | | 87.86762 | | 431.0793 | | boat | | | boat | | | boat | | Boats | | | high | | 5 | 1 | | 11.53922 | | Boat | | RR Ties |
| 170 | 169 | | 501.3155 | | 3 | 21.11876 | | 748.9786 | | 13.91537 | | 100tonmixed4 | | 316.8873 | | 4826.869 | | 75 ton ties, 25 tons broken concrete | | | rr ties, concrete | | | RR, Concrete | | RR, Concrete | | | mid | | 6 | 2 | | 7.217236 | | RR Ties | | RR Ties |
| 171 | 170 | | 137.1343 | | 2 | 21.99815 | | 496.8355 | | 45.77096 | | 3-4 Block10 | | 55.32938 | | 227.4086 | | 3 4 in palet cinderblocks | | | cinderblocks | | | 3 pallets cinderblocks | | 3CB | | | low | | 8 | 1 | | 5.920961 | | Cinderblocks | | RR Ties |
| 172 | 171 | | 600.2314 | | 2 | 22.6091 | | 596.6185 | | 56.99909 | | D-1 | | 857.3153 | | 41943.89 | | Large Spool, broken ties, 15ft Spools | | | rr ties, spools, concrete | | | RR, Spools, Concrete | | RR, Spools, Concrete | | | mid-high | | 5 | 3 | | 7.397315 | | Other / Mixed | | RR Ties |
| 173 | 172 | | 465.9785 | | 2 | 21.68196 | | 556.5726 | | 24.99731 | | LS11 | | 29.21247 | | 63.39908 | | One Gulf LP | | | limestone | | | limestone | | Limestone | | | low | | 8 | 1 | | 7.144139 | | Concrete | | RR Ties |
| 174 | 173 | | 6143.731 | | 2 | 22.29005 | | 247.8613 | | 49.76874 | | Patch 82 | | 281.5516 | | 4100.548 | | mixed patch w/ 1 6 in palet cinderblocks  2 8 in palet cinderblocks  2 10x10 box culverts  3 pieces concrete highway divider  3 pieces concrete highway divider  low profile chunks of concrete | | | cinderblocks, culverts, highway divider, concrete | | | cinderblocks, culverts, highway divider, concrete | | CB, Culvert, HD, Concrete | | | mid-high | | 9 | 4 | | 9.723187 | | Other / Mixed | | RR Ties |
| 175 | 174 | | 6527.948 | | 3 | 21.38538 | | 315.5371 | | 56.45948 | | Patch 63 | | 37.19301 | | 83.95348 | | single pyramid | | | pyramid | | | 1PY | | 1PY | | | mid | | 8 | 1 | | 9.783848 | | Pyramids | | Boat |
| 176 | 175 | | 256.4811 | | 2 | 20.65069 | | 717.3814 | | 94.91273 | | Patch 51 | | 107.5291 | | 585.2189 | | mixed; pyramids, lpm | | | pyramids, low profile | | | 1MX | | 1MX | | | mid | | 8 | 2 | | 6.547055 | | Pyramids | | Boat |
| 177 | 176 | | 2976.431 | | 2 | 22.81222 | | 432.4327 | | 94.70254 | | D-24 | | 838.8928 | | 44936.25 | | 18 ft spool,12-15 ft spools, concrete rubble, culverts | | | rr ties, spools, concrete, culverts | | | RR, Spools, Concrete, Culvert | | RR, Spools, Concrete, Culvert | | | mid-high | | 5 | 4 | | 8.99848 | | Other / Mixed | | Boat |
| 178 | 177 | | 39575.23 | | 21 | 22.3118 | | 362.37 | | 9.69386 | | Patch 104 | | 79.63223 | | 329.7727 | | mixed patch w/ 2 8 in palet cinderblocks   low profile chunks of concrete | | | cinderblocks, concrete | | | cinderblocks, concrete | | CB, Concrete | | | low | | 9 | 2 | | 11.58596 | | Other / Mixed | | Boat |
| 179 | 178 | | 1141.181 | | 18 | 22.01924 | | 309.3127 | | 35.63466 | | 100tonmixed22 | | 328.8479 | | 5628.861 | | 75 ton ties, 25 tons broken concrete | | | rr ties, concrete | | | RR, Concrete | | RR, Concrete | | | mid | | 6 | 2 | | 8.039819 | | RR Ties | | Boat |
| 180 | 179 | | 2660.932 | | 3 | 21.22028 | | 309.866 | | 46.93695 | | Extra rr ties 1 | | 135.3473 | | 811.1455 | | RR ties | | | rr ties | | | RR | | Extra RR | | | mid | | 6 | 1 | | 8.886432 | | RR Ties | | Boat |
| 181 | 180 | | 1348.274 | | 9 | 21.08823 | | 319.6037 | | 46.2545 | | Mix_3 | | 138.3346 | | 1324.907 | | 25 ton RR Ties, 25 ton Concrete, 6 Pallets CinderBlock | | | rr ties, concrete, cinderblocks | | | RR, Concrete, CB | | RR, Concrete, CB | | | mid | | 6 | 3 | | 8.20658 | | RR Ties | | RR Ties |
| 182 | 181 | | 1503.919 | | 74 | 18.78608 | | 84.28666 | | 143.7774 | | Patch 6 | | 163.488 | | 1152.274 | | mixed; pyramids, lpm | | | pyramids, low profile | | | 1MX | | 1MX | | | mid | | 8 | 2 | | 8.31583 | | Pyramids | | RR Ties |
| 183 | 182 | | 285451.5 | | 2235 | 21.67068 | | 126.0782 | | 22.34933 | | 500T_1 | | 411.7361 | | 10206.17 | | 500 rr ties | | | rr ties | | | 500T RR | | 500T RR | | | high | | 6 | 1 | | 13.56183 | | RR Ties | | RR Ties |
| 184 | 183 | | 210773.2 | | 31 | 22.67122 | | 139.9592 | | 2.93608 | | 250-5 | | 449.1978 | | 13227.3 | | 250 ton pile rr ties | | | rr ties | | | 250T RR | | 250T RR | | | mid-high | | 6 | 1 | | 13.25854 | | RR Ties | | RR Ties |
| 185 | 184 | | 1084.114 | | 54 | 21.07874 | | 753.9202 | | 34.30869 | | Andy Faskin | | 87.86762 | | 431.0793 | | boat | | | boat | | | boat | | Boats | | | high | | 5 | 1 | | 7.988519 | | Boat | | RR Ties |
| 186 | 185 | | 3388.385 | | 170 | 22.93899 | | 395.3016 | | 59.31282 | | D-15 | | 570.4288 | | 18953.98 | | Biggest spoked spool, 12ft spools, concrete railroad ties | | | rr ties, spools | | | RR, Spools | | RR, Spools | | | mid-high | | 5 | 2 | | 9.128109 | | Other / Mixed | | RR Ties |
| 187 | 186 | | 24262.22 | | 163 | 19.58459 | | 205.7323 | | 19.06645 | | Patch 3 | | 324.2377 | | 5122.985 | | pyramids | | | pyramids | | | 4PY | | 4PY | | | mid | | 8 | 1 | | 11.09668 | | Pyramids | | RR Ties |
| 188 | 187 | | 11508.67 | | 575 | 23.07728 | | 307.7836 | | 53.61523 | | D-23 | | 821.8087 | | 31780.67 | | Spools, broken concrete | | | rr ties, spools, concrete | | | RR, Spools, Concrete | | RR, Spools, Concrete | | | mid-high | | 5 | 3 | | 10.35086 | | Other / Mixed | | RR Ties |
| 189 | 188 | | 4556.109 | | 2 | 21.5448 | | 251.7839 | | 53.74284 | | Patch 78 | | 340.1461 | | 6208.392 | | mixed patch w/ 1 6 in palet cinderblocks  2 8 in palet cinderblocks  2 10x10 box culverts  3 pieces concrete highway divider  low profile chunks of concrete | | | cinderblocks, culverts, highway divider, concrete | | | cinderblocks, culverts, highway divider, concrete | | CB, Culvert, HD, Concrete | | | mid-high | | 9 | 4 | | 9.424224 | | Other / Mixed | | RR Ties |
| 190 | 189 | | 8085.926 | | 2 | 21.55264 | | 239.001 | | 34.59599 | | 1-8 Block37 | | 40.67442 | | 102.3391 | | 1 8 in palet cinderblocks | | | cinderblocks | | | 1 pallet cinderblocks | | 1CB | | | low | | 8 | 1 | | 9.99788 | | Cinderblocks | | RR Ties |
| 191 | 190 | | 1910.252 | | 2 | 22.11999 | | 227.2168 | | 58.85125 | | Patch 80 | | 292.6903 | | 5145.313 | | mixed patch w/ 1 6 in palet cinderblocks  2 8 in palet cinderblocks  2 10x10 box culverts  3 pieces concrete highway divider  low profile chunks of concrete | | | cinderblocks, culverts, highway divider, concrete | | | cinderblocks, culverts, highway divider, concrete | | CB, Culvert, HD, Concrete | | | mid-high | | 9 | 4 | | 8.55499 | | Other / Mixed | | RR Ties |
| 192 | 191 | | 13182.96 | | 7 | 22.07498 | | 847.4287 | | 12.85846 | | Concrete10 ton6 | | 44.18505 | | 117.3124 | | 10 ton concrete | | | concrete | | | 10 ton concrete | | 10T Concrete | | | low | | 8 | 1 | | 10.48668 | | Concrete | | RR Ties |
| 193 | 192 | | 36183.53 | | 9 | 21.97752 | | 838.8123 | | 8.349939 | | Concrete10 ton7 | | 58.80284 | | 213.2196 | | 10 ton concrete | | | concrete | | | 10 ton concrete | | 10T Concrete | | | low | | 8 | 1 | | 11.49636 | | Concrete | | RR Ties |
| 194 | 193 | | 5475.042 | | 4 | 21.8508 | | 870.0239 | | 20.02569 | | Concrete10 ton8 | | 85.45058 | | 470.6305 | | 10 ton concrete | | | concrete | | | 10 ton concrete | | 10T Concrete | | | low | | 8 | 1 | | 9.607955 | | Concrete | | RR Ties |
| 195 | 194 | | 60512.32 | | 247 | 18.88598 | | 210.9148 | | 25.30512 | | Patch 11 | | 608.4765 | | 23407.14 | | mixed; pyramids, lpm | | | pyramids, low profile | | | 16MX | | 16MX | | | mid | | 8 | 2 | | 12.0106 | | Pyramids | | RR Ties |
| 196 | 195 | | 19820.99 | | 9 | 18.70017 | | 250.9362 | | 55.78115 | | Patch 11 | | 608.4765 | | 23407.14 | | mixed; pyramids, lpm | | | pyramids, low profile | | | 16MX | | 16MX | | | mid | | 8 | 2 | | 10.8945 | | Pyramids | | RR Ties |
| 197 | 196 | | 1910.1 | | 2 | 19.87058 | | 524.8856 | | 33.30073 | | Patch 18 | | 658.7559 | | 29101.67 | | mixed; pyramids, lpm | | | pyramids, low profile | | | 16MX | | 16MX | | | mid | | 8 | 2 | | 8.554911 | | Pyramids | | RR Ties |
| 198 | 197 | | 3964.107 | | 3 | 20.16667 | | 582.2144 | | 26.61414 | | Patch 18 | | 658.7559 | | 29101.67 | | mixed; pyramids, lpm | | | pyramids, low profile | | | 16MX | | 16MX | | | mid | | 8 | 2 | | 9.285036 | | Pyramids | | RR Ties |
| 199 | 198 | | 20803.73 | | 15 | 20.13307 | | 559.1563 | | 5.668634 | | Patch 18 | | 658.7559 | | 29101.67 | | mixed; pyramids, lpm | | | pyramids, low profile | | | 16MX | | 16MX | | | mid | | 8 | 2 | | 10.94289 | | Pyramids | | RR Ties |
| 200 | 199 | | 1660.348 | | 3 | 19.54933 | | 581.2181 | | 35.43257 | | Patch 18 | | 658.7559 | | 29101.67 | | mixed; pyramids, lpm | | | pyramids, low profile | | | 16MX | | 16MX | | | mid | | 8 | 2 | | 8.414783 | | Pyramids | | RR Ties |
| 201 | 200 | | 1025.944 | | 14 | 19.80846 | | 569.6484 | | 16.04817 | | Patch 18 | | 658.7559 | | 29101.67 | | mixed; pyramids, lpm | | | pyramids, low profile | | | 16MX | | 16MX | | | mid | | 8 | 2 | | 7.933368 | | Pyramids | | RR Ties |
| 202 | 201 | | 6348.612 | | 2 | 19.89317 | | 538.9703 | | 28.59657 | | Patch 18 | | 658.7559 | | 29101.67 | | mixed; pyramids, lpm | | | pyramids, low profile | | | 16MX | | 16MX | | | mid | | 8 | 2 | | 9.755992 | | Pyramids | | RR Ties |
| 203 | 202 | | 16745.48 | | 2 | 19.43661 | | 230.1022 | | 21.3628 | | Patch 21 | | 721.9896 | | 30699.53 | | mixed; pyramids, lpm | | | pyramids, low profile | | | 16MX | | 16MX | | | mid | | 8 | 2 | | 10.72588 | | Pyramids | | RR Ties |
| 204 | 203 | | 762.6259 | | 2 | 19.56525 | | 175.4477 | | 39.30498 | | Patch 21 | | 721.9896 | | 30699.53 | | mixed; pyramids, lpm | | | pyramids, low profile | | | 16MX | | 16MX | | | mid | | 8 | 2 | | 7.636768 | | Pyramids | | RR Ties |
| 205 | 204 | | 21751.58 | | 5 | 18.92188 | | 197.9048 | | 45.5661 | | Patch 21 | | 721.9896 | | 30699.53 | | mixed; pyramids, lpm | | | pyramids, low profile | | | 16MX | | 16MX | | | mid | | 8 | 2 | | 10.98744 | | Pyramids | | RR Ties |
| 206 | 205 | | 11122.99 | | 3 | 20.26701 | | 288.1256 | | 25.91676 | | Patch 48 | | 637.7184 | | 23935.57 | | mixed; pyramids, lpm | | | pyramids, low profile | | | 16MX | | 16MX | | | mid | | 8 | 2 | | 10.31677 | | Pyramids | | RR Ties |
| 207 | 206 | | 2702.993 | | 3 | 19.91514 | | 277.9938 | | 43.54772 | | Patch 48 | | 637.7184 | | 23935.57 | | mixed; pyramids, lpm | | | pyramids, low profile | | | 16MX | | 16MX | | | mid | | 8 | 2 | | 8.902115 | | Pyramids | | RR Ties |
| 208 | 207 | | 20543.2 | | 20 | 20.09696 | | 245.0975 | | 40.60394 | | Patch 48 | | 637.7184 | | 23935.57 | | mixed; pyramids, lpm | | | pyramids, low profile | | | 16MX | | 16MX | | | mid | | 8 | 2 | | 10.93029 | | Pyramids | | RR Ties |
| 209 | 208 | | 10052.48 | | 8 | 20.52655 | | 220.5336 | | 50.48353 | | Patch 5 | | 581.2411 | | 24281.88 | | mixed; pyramids, lpm | | | pyramids, low profile | | | 16MX | | 16MX | | | mid | | 8 | 2 | | 10.21557 | | Pyramids | | RR Ties |
| 210 | 209 | | 2810.911 | | 2 | 20.88081 | | 198.4082 | | 10.19056 | | Patch 5 | | 581.2411 | | 24281.88 | | mixed; pyramids, lpm | | | pyramids, low profile | | | 16MX | | 16MX | | | mid | | 8 | 2 | | 8.941264 | | Pyramids | | RR Ties |
| 211 | 210 | | 7246.22 | | 4 | 20.79432 | | 212.0189 | | 31.29545 | | Patch 5 | | 581.2411 | | 24281.88 | | mixed; pyramids, lpm | | | pyramids, low profile | | | 16MX | | 16MX | | | mid | | 8 | 2 | | 9.888235 | | Pyramids | | RR Ties |
| 212 | 211 | | 1270.46 | | 2 | 21.12715 | | 623.3404 | | 15.61826 | | Patch 51 | | 107.5291 | | 585.2189 | | mixed; pyramids, lpm | | | pyramids, low profile | | | 1MX | | 1MX | | | mid | | 8 | 2 | | 8.147134 | | Pyramids | | RR Ties |
| 213 | 212 | | 3309.208 | | 3 | 20.9858 | | 641.3203 | | 9.191207 | | Patch 51 | | 107.5291 | | 585.2189 | | mixed; pyramids, lpm | | | pyramids, low profile | | | 1MX | | 1MX | | | mid | | 8 | 2 | | 9.104464 | | Pyramids | | RR Ties |
| 214 | 213 | | 12030.53 | | 6 | 20.58227 | | 743.4483 | | 28.53108 | | Patch 20 | | 804.1207 | | 27295.57 | | pyramids | | | pyramids | | | 16PY | | 16PY | | | mid | | 8 | 1 | | 10.3952 | | Pyramids | | RR Ties |
| 215 | 214 | | 18319.07 | | 6 | 19.7501 | | 389.4104 | | 16.91332 | | Patch 27 | | 713.1051 | | 24736.61 | | pyramids | | | pyramids | | | 16PY | | 16PY | | | mid | | 8 | 1 | | 10.8157 | | Pyramids | | RR Ties |
| 216 | 215 | | 15635.55 | | 3 | 20.05146 | | 772.6909 | | 35.45972 | | Patch 29 | | 581.9387 | | 17854.57 | | pyramids | | | pyramids | | | 16PY | | 16PY | | | mid | | 8 | 1 | | 10.6573 | | Pyramids | | RR Ties |
| 217 | 216 | | 16223.5 | | 14 | 18.91974 | | 212.1281 | | 45.87644 | | Patch 41 | | 628.1935 | | 23819.9 | | pyramids | | | pyramids | | | 16PY | | 16PY | | | mid | | 8 | 1 | | 10.69422 | | Pyramids | | RR Ties |
| 218 | 217 | | 39574.54 | | 25 | 19.76391 | | 392.9029 | | 49.98594 | | Patch 8 | | 617.3887 | | 25733.57 | | pyramids | | | pyramids | | | 16PY | | 16PY | | | mid | | 8 | 1 | | 11.58594 | | Pyramids | | RR Ties |
| 219 | 218 | | 28445.75 | | 62 | 20.08064 | | 380.1202 | | 13.35958 | | Patch 8 | | 617.3887 | | 25733.57 | | pyramids | | | pyramids | | | 16PY | | 16PY | | | mid | | 8 | 1 | | 11.25575 | | Pyramids | | RR Ties |
| 220 | 219 | | 6284.105 | | 4 | 20.02347 | | 729.7345 | | 9.530778 | | Patch 19 | | 42.212 | | 84.86567 | | mixed; pyramids, lpm | | | pyramids, low profile | | | 1MX | | 1MX | | | mid | | 8 | 2 | | 9.745779 | | Pyramids | | RR Ties |
| 221 | 220 | | 6269.445 | | 3 | 19.37517 | | 207.8105 | | 8.287314 | | Patch 31 | | 69.6398 | | 302.1493 | | mixed; pyramids, lpm | | | pyramids, low profile | | | 1MX | | 1MX | | | mid | | 8 | 2 | | 9.743443 | | Pyramids | | RR Ties |
| 222 | 221 | | 19443.49 | | 13 | 18.91784 | | 184.0138 | | 28.47432 | | Patch 6 | | 163.488 | | 1152.274 | | mixed; pyramids, lpm | | | pyramids, low profile | | | 1MX | | 1MX | | | mid | | 8 | 2 | | 10.87527 | | Pyramids | | RR Ties |
| 223 | 222 | | 11669.65 | | 4 | 20.19976 | | 570.3249 | | 26.41732 | | Patch 14 | | 44.59184 | | 105.6696 | | single pyramid | | | pyramid | | | 1PY | | 1PY | | | mid | | 8 | 1 | | 10.36475 | | Pyramids | | RR Ties |
| 224 | 223 | | 5233.234 | | 83 | 19.30984 | | 201.1655 | | 2.411915 | | Patch 46 | | 48.74995 | | 136.2459 | | single pyramid | | | pyramid | | | 1PY | | 1PY | | | mid | | 8 | 1 | | 9.562785 | | Pyramids | | RR Ties |
| 225 | 224 | | 4127.463 | | 5 | 22.59431 | | 574.5072 | | 23.36392 | | Concrete2 ton12 | | 41.67629 | | 94.5566 | | 2 ton concrete | | | concrete | | | 2 ton concrete | | 2T Concrete | | | low | | 8 | 1 | | 9.325418 | | Concrete | | RR Ties |
| 226 | 225 | | 5275.303 | | 2 | 23.22489 | | 187.7079 | | 12.82536 | | Concrete2 ton3 | | 177.9373 | | 1772.472 | | 2 ton concrete | | | concrete | | | 2 ton concrete | | 2T Concrete | | | low | | 8 | 1 | | 9.570791 | | Concrete | | RR Ties |
| 227 | 226 | | 1827.183 | | 2 | 21.98352 | | 362.5061 | | 6.42388 | | Concrete2 ton7 | | 180.12 | | 825.461 | | 2 ton concrete | | | concrete | | | 2 ton concrete | | 2T Concrete | | | low | | 8 | 1 | | 8.510531 | | Concrete | | RR Ties |
| 228 | 227 | | 181790.6 | | 7 | 23.21015 | | 133.7235 | | 11.02597 | | 250-1 | | 403.1612 | | 12376.16 | | 250 ton pile rr ties | | | rr ties | | | 250T RR | | 250T RR | | | mid-high | | 6 | 1 | | 13.11061 | | RR Ties | | RR Ties |
| 229 | 228 | | 28770.44 | | 12 | 21.939 | | 129.1025 | | 13.15456 | | 250-10 | | 428.8781 | | 12050.11 | | 250 ton pile rr ties | | | rr ties | | | 250T RR | | 250T RR | | | mid-high | | 6 | 1 | | 11.2671 | | RR Ties | | RR Ties |
| 230 | 229 | | 5787.633 | | 3 | 21.84701 | | 129.8592 | | 22.38812 | | 250-11 | | 453.4038 | | 12760.36 | | 250 ton pile rr ties | | | rr ties | | | 250T RR | | 250T RR | | | mid-high | | 6 | 1 | | 9.663479 | | RR Ties | | RR Ties |
| 231 | 230 | | 16014.04 | | 3 | 21.97753 | | 125.7084 | | 3.107278 | | 250-11 | | 453.4038 | | 12760.36 | | 250 ton pile rr ties | | | rr ties | | | 250T RR | | 250T RR | | | mid-high | | 6 | 1 | | 10.68122 | | RR Ties | | RR Ties |
| 232 | 231 | | 26064.26 | | 4 | 22.92092 | | 218.5685 | | 6.517301 | | 250-18 | | 502.4975 | | 14896.36 | | 250 ton pile rr ties | | | rr ties | | | 250T RR | | 250T RR | | | mid-high | | 6 | 1 | | 11.16832 | | RR Ties | | RR Ties |
| 233 | 232 | | 4828.51 | | 3 | 23.01563 | | 250.4439 | | 15.13617 | | 250-19 | | 398.0899 | | 9756.074 | | 250 ton pile rr ties | | | rr ties | | | 250T RR | | 250T RR | | | mid-high | | 6 | 1 | | 9.482293 | | RR Ties | | RR Ties |
| 234 | 233 | | 1258.24 | | 37 | 22.78247 | | 224.0706 | | 9.808669 | | 250-20 | | 547.0743 | | 17094.68 | | 250 ton pile rr ties | | | rr ties | | | 250T RR | | 250T RR | | | mid-high | | 6 | 1 | | 8.137469 | | RR Ties | | RR Ties |
| 235 | 234 | | 11172.32 | | 3 | 23.32007 | | 153.8036 | | 22.84941 | | 250-21 | | 495.6666 | | 13837.4 | | 250 ton pile rr ties | | | rr ties | | | 250T RR | | 250T RR | | | mid-high | | 6 | 1 | | 10.32119 | | RR Ties | | RR Ties |
| 236 | 235 | | 6302.186 | | 3 | 23.31234 | | 140.9575 | | 35.96378 | | 250-21 | | 495.6666 | | 13837.4 | | 250 ton pile rr ties | | | rr ties | | | 250T RR | | 250T RR | | | mid-high | | 6 | 1 | | 9.748652 | | RR Ties | | RR Ties |
| 237 | 236 | | 6767.332 | | 2 | 23.15244 | | 183.297 | | 1.101365 | | 250-22 | | 439.6424 | | 9330.136 | | 250 ton pile rr ties | | | rr ties | | | 250T RR | | 250T RR | | | mid-high | | 6 | 1 | | 9.819862 | | RR Ties | | RR Ties |
| 238 | 237 | | 58447.84 | | 8 | 22.94398 | | 130.5352 | | 10.29117 | | 250-3 | | 533.8506 | | 12928.94 | | 250 ton pile rr ties | | | rr ties | | | 250T RR | | 250T RR | | | mid-high | | 6 | 1 | | 11.97589 | | RR Ties | | RR Ties |
| 239 | 238 | | 10783.89 | | 3 | 22.35392 | | 110.6031 | | 6.277164 | | 250-7 | | 493.0979 | | 12838.84 | | 250 ton pile rr ties | | | rr ties | | | 250T RR | | 250T RR | | | mid-high | | 6 | 1 | | 10.28581 | | RR Ties | | RR Ties |
| 240 | 239 | | 31100.93 | | 7 | 22.51374 | | 120.5221 | | 4.295281 | | 250-8 | | 435.2568 | | 12347.32 | | 250 ton pile rr ties | | | rr ties | | | 250T RR | | 250T RR | | | mid-high | | 6 | 1 | | 11.34499 | | RR Ties | | RR Ties |
| 241 | 240 | | 686.8596 | | 2 | 20.03449 | | 99.34195 | | 9.149767 | | 3-4 Block2 | | 81.21312 | | 364.3771 | | 3 4 in palet cinderblocks | | | cinderblocks | | | 3 pallets cinderblocks | | 3CB | | | low | | 8 | 1 | | 7.53213 | | Cinderblocks | | RR Ties |
| 242 | 241 | | 7767.483 | | 3 | 22.87533 | | 325.6882 | | 17.17128 | | 3-4 Block6 | | 95.47537 | | 564.5025 | | 3 4 in palet cinderblocks | | | cinderblocks | | | 3 pallets cinderblocks | | 3CB | | | low | | 8 | 1 | | 9.957701 | | Cinderblocks | | RR Ties |
| 243 | 242 | | 6789.283 | | 5 | 22.04528 | | 1032.589 | | 28.22099 | | 3-8 Block14 | | 199.1356 | | 2396.159 | | 3 8 in pallets cinderblocks | | | cinderblocks | | | 3 pallets cinderblocks | | 3CB | | | low | | 8 | 1 | | 9.823101 | | Cinderblocks | | RR Ties |
| 244 | 243 | | 10477.28 | | 3 | 21.49671 | | 1039.479 | | 18.36318 | | 3-mixed Block21 | | 121.5736 | | 1063.589 | | 3 pallets mixed size ciderblocks | | | cinderblocks | | | 3 pallets cinderblocks | | 3CB | | | low | | 8 | 1 | | 10.25696 | | Cinderblocks | | RR Ties |
| 245 | 244 | | 18896.28 | | 3 | 21.29401 | | 802.5316 | | 14.70761 | | 3-mixed Block22 | | 109.9789 | | 849.4539 | | 3 pallets mixed size ciderblocks | | | cinderblocks | | | 3 pallets cinderblocks | | 3CB | | | low | | 8 | 1 | | 10.84672 | | Cinderblocks | | RR Ties |
| 246 | 245 | | 19842.91 | | 2 | 22.98312 | | 280.2725 | | 34.80954 | | CCA Corner | | 1377.797 | | 60636 | | 100 tons rr ties in line | | | CCA rr tie ridge | | | CCA rr tie ridge | | CCA Ridge | | | mid-high | | 6 | 1 | | 10.8956 | | RR Ties | | RR Ties |
| 247 | 246 | | 4979.582 | | 2 | 21.70286 | | 817.5909 | | 3.277784 | | 3-mixed Block39 | | 96.6829 | | 513.8501 | | 3 pallets mixed size ciderblocks | | | cinderblocks | | | 3 pallets cinderblocks | | 3CB | | | low | | 8 | 1 | | 9.513101 | | Cinderblocks | | RR Ties |
| 248 | 247 | | 16125.62 | | 2 | 21.50017 | | 978.9406 | | 4.824634 | | 30-mixed Block3 | | 223.7235 | | 3484.575 | | 30 pallets mixed size cinderblocks | | | cinderblocks | | | 30 pallets cinderblocks | | 30CB | | | low-mid | | 8 | 1 | | 10.68816 | | Cinderblocks | | RR Ties |
| 249 | 248 | | 1685.752 | | 3 | 20.18907 | | 535.7435 | | 61.69083 | | Patch 15 | | 300.8062 | | 5493.472 | | low profile | | | low profile | | | 4LP | | 4LP | | | low | | 8 | 1 | | 8.429967 | | Low Profile | | RR Ties |
| 250 | 249 | | 2764.814 | | 2 | 19.21339 | | 250.4736 | | 64.65612 | | Patch 47 | | 289.7673 | | 3959.842 | | low profile | | | low profile | | | 4LP | | 4LP | | | low | | 8 | 1 | | 8.924729 | | Low Profile | | RR Ties |
| 251 | 250 | | 2622.611 | | 2 | 20.48765 | | 374.8077 | | 12.55306 | | Patch 10 | | 307.7802 | | 5655.625 | | mixed; pyramids, lpm | | | pyramids, low profile | | | 4MX | | 4MX | | | mid | | 8 | 2 | | 8.871926 | | Pyramids | | RR Ties |
| 252 | 251 | | 7152.92 | | 2 | 20.4596 | | 378.2051 | | 3.85782 | | Patch 10 | | 307.7802 | | 5655.625 | | mixed; pyramids, lpm | | | pyramids, low profile | | | 4MX | | 4MX | | | mid | | 8 | 2 | | 9.875276 | | Pyramids | | RR Ties |
| 253 | 252 | | 3281.256 | | 13 | 19.57223 | | 195.7254 | | 18.58388 | | Patch 16 | | 409.546 | | 6681.443 | | mixed; pyramids, lpm | | | pyramids, low profile | | | 4MX | | 4MX | | | mid | | 8 | 2 | | 9.095981 | | Pyramids | | RR Ties |
| 254 | 253 | | 24912.04 | | 21 | 19.28091 | | 219.9643 | | 8.504002 | | Patch 16 | | 409.546 | | 6681.443 | | mixed; pyramids, lpm | | | pyramids, low profile | | | 4MX | | 4MX | | | mid | | 8 | 2 | | 11.12311 | | Pyramids | | RR Ties |
| 255 | 254 | | 2709.914 | | 3 | 19.48361 | | 172.7987 | | 41.33037 | | Patch 16 | | 409.546 | | 6681.443 | | mixed; pyramids, lpm | | | pyramids, low profile | | | 4MX | | 4MX | | | mid | | 8 | 2 | | 8.904672 | | Pyramids | | RR Ties |
| 256 | 255 | | 33956.77 | | 11 | 20.50417 | | 942.1482 | | 21.51155 | | Patch 25 | | 346.4492 | | 8016.418 | | mixed; pyramids, lpm | | | pyramids, low profile | | | 4MX | | 4MX | | | mid | | 8 | 2 | | 11.43284 | | Pyramids | | RR Ties |
| 257 | 256 | | 1083.834 | | 6 | 20.78847 | | 910.0886 | | 18.12163 | | Patch 25 | | 346.4492 | | 8016.418 | | mixed; pyramids, lpm | | | pyramids, low profile | | | 4MX | | 4MX | | | mid | | 8 | 2 | | 7.98826 | | Pyramids | | RR Ties |
| 258 | 257 | | 16938.54 | | 8 | 19.69993 | | 371.1442 | | 11.34652 | | Patch 32 | | 360.0463 | | 8019.002 | | mixed; pyramids, lpm | | | pyramids, low profile | | | 4MX | | 4MX | | | mid | | 8 | 2 | | 10.73735 | | Pyramids | | RR Ties |
| 259 | 258 | | 1154.742 | | 2 | 19.60149 | | 360.8198 | | 23.11139 | | Patch 32 | | 360.0463 | | 8019.002 | | mixed; pyramids, lpm | | | pyramids, low profile | | | 4MX | | 4MX | | | mid | | 8 | 2 | | 8.051632 | | Pyramids | | RR Ties |
| 260 | 259 | | 6898.834 | | 2 | 19.40795 | | 397.0573 | | 16.11082 | | Patch 32 | | 360.0463 | | 8019.002 | | mixed; pyramids, lpm | | | pyramids, low profile | | | 4MX | | 4MX | | | mid | | 8 | 2 | | 9.839108 | | Pyramids | | RR Ties |
| 261 | 260 | | 66625.47 | | 618 | 19.12519 | | 367.1964 | | 17.33992 | | Patch 7 | | 362.0707 | | 7757.877 | | mixed; pyramids, lpm | | | pyramids, low profile | | | 4MX | | 4MX | | | mid | | 8 | 2 | | 12.10684 | | Pyramids | | RR Ties |
| 262 | 261 | | 50255.74 | | 14 | 19.61365 | | 381.1091 | | 1.614254 | | Patch 17 | | 258.5143 | | 4003.081 | | pyramids | | | pyramids | | | 4PY | | 4PY | | | mid | | 8 | 1 | | 11.82488 | | Pyramids | | RR Ties |
| 263 | 262 | | 28073.41 | | 12 | 20.15241 | | 747.6596 | | 14.83461 | | Patch 24 | | 248.2686 | | 3252.476 | | pyramids | | | pyramids | | | 4PY | | 4PY | | | mid | | 8 | 1 | | 11.24258 | | Pyramids | | RR Ties |
| 264 | 263 | | 62888.25 | | 4 | 20.0597 | | 572.6132 | | 7.723299 | | Patch 33 | | 188.9853 | | 1770.835 | | pyramids | | | pyramids | | | 4PY | | 4PY | | | mid | | 8 | 1 | | 12.04911 | | Pyramids | | RR Ties |
| 265 | 264 | | 12465.51 | | 2 | 19.39764 | | 234.0094 | | 34.21301 | | Patch 36 | | 312.6145 | | 5909.587 | | pyramids | | | pyramids | | | 4PY | | 4PY | | | mid | | 8 | 1 | | 10.43072 | | Pyramids | | Concrete |
| 266 | 265 | | 1088.507 | | 2 | 19.78415 | | 197.3189 | | 11.1131 | | Patch 36 | | 312.6145 | | 5909.587 | | pyramids | | | pyramids | | | 4PY | | 4PY | | | mid | | 8 | 1 | | 7.992562 | | Pyramids | | Concrete |
| 267 | 266 | | 6453.721 | | 3 | 20.17655 | | 465.1187 | | 44.13377 | | Patch 45 | | 249.6864 | | 3879.805 | | pyramids | | | pyramids | | | 4PY | | 4PY | | | mid | | 8 | 1 | | 9.772412 | | Pyramids | | Concrete |
| 268 | 267 | | 2920.247 | | 2 | 20.94468 | | 264.8232 | | 35.81454 | | Patch 53 | | 329.6576 | | 6980.813 | | pyramids | | | pyramids | | | 4PY | | 4PY | | | mid | | 8 | 1 | | 8.979424 | | Pyramids | | Concrete |
| 269 | 268 | | 623.5867 | | 2 | 20.50788 | | 459.8128 | | 25.16643 | | Patch 54 | | 416.7709 | | 9568.277 | | pyramids | | | pyramids | | | 4PY | | 4PY | | | mid | | 8 | 1 | | 7.435488 | | Pyramids | | Concrete |
| 270 | 269 | | 19416.71 | | 56 | 20.93938 | | 814.2101 | | 28.63164 | | Patch 57 | | 242.1795 | | 3523.531 | | cinderblocks, pyramids | | | cinderblocks, pyramids | | | cinderblocks, pyramids | | CB, PY | | | mid | | 9 | 2 | | 10.87389 | | Pyramids | | Concrete |
| 271 | 270 | | 78881.83 | | 12 | 20.65208 | | 822.987 | | 30.11457 | | Patch 57 | | 242.1795 | | 3523.531 | | cinderblocks, pyramids | | | cinderblocks, pyramids | | | cinderblocks, pyramids | | CB, PY | | | mid | | 9 | 2 | | 12.27571 | | Pyramids | | Concrete |
| 272 | 271 | | 21886.26 | | 8 | 20.67785 | | 942.8613 | | 24.911 | | Patch 59 | | 447.1259 | | 11696.09 | | cinderblocks, pyramids | | | cinderblocks, pyramids | | | cinderblocks, pyramids | | CB, PY | | | mid | | 9 | 2 | | 10.99361 | | Pyramids | | Concrete |
| 273 | 272 | | 2145.579 | | 2 | 22.18251 | | 902.1543 | | 22.52228 | | 50.1 | | 345.7408 | | 6620.539 | | 50 rr tie | | | rr ties | | | 50 rr tie | | 50T RR | | | low-mid | | 8 | 1 | | 8.671165 | | RR Ties | | Concrete |
| 274 | 273 | | 19841.12 | | 683 | 21.09664 | | 324.672 | | 21.44337 | | 500T_2 | | 431.6438 | | 12308.43 | | 500 rr ties | | | rr ties | | | 500T RR | | 500T RR | | | high | | 6 | 1 | | 10.89551 | | RR Ties | | Concrete |
| 275 | 274 | | 27765.6 | | 16 | 21.03711 | | 357.8143 | | 14.54103 | | 500T_2 | | 431.6438 | | 12308.43 | | 500 rr ties | | | rr ties | | | 500T RR | | 500T RR | | | high | | 6 | 1 | | 11.23155 | | RR Ties | | Concrete |
| 276 | 275 | | 6713.721 | | 4 | 21.07002 | | 341.0857 | | 16.43982 | | 500T_2 | | 431.6438 | | 12308.43 | | 500 rr ties | | | rr ties | | | 500T RR | | 500T RR | | | high | | 6 | 1 | | 9.811909 | | RR Ties | | Concrete |
| 277 | 276 | | 31230.79 | | 165 | 22.53987 | | 313.6131 | | 20.1563 | | Big Pile | | 771.4804 | | 34964.52 | | big pile | | | big pile | | | big pile | | Big Pile | | | high | | 8 | 1 | | 11.34916 | | Big Pile | | Concrete |
| 278 | 277 | | 807317 | | 33919 | 21.89843 | | 737.1365 | | 10.72534 | | Billy Kenon | | 134.7889 | | 934.4976 | | boat | | | boat | | | boat | | Boats | | | high | | 5 | 1 | | 14.60147 | | Boat | | Concrete |
| 279 | 278 | | 346756.1 | | 17297 | 21.89091 | | 725.5797 | | 22.33003 | | Billy Kenon | | 134.7889 | | 934.4976 | | boat | | | boat | | | boat | | Boats | | | high | | 5 | 1 | | 13.75638 | | Boat | | Low Profile |
| 280 | 279 | | 17847.29 | | 8 | 22.38785 | | 747.6611 | | 16.50875 | | EMR Capt Berry | | 205.433 | | 2309.324 | | boat | | | boat | | | boat | | Boats | | | high | | 6 | 1 | | 10.78961 | | Boat | | Low Profile |
| 281 | 280 | | 124234.4 | | 4677 | 22.36063 | | 777.4906 | | 17.84322 | | 400 | | 428.1912 | | 10399.7 | | 400 rr tie | | | rr ties | | | 400 rr tie | | 400T RR | | | high | | 8 | 1 | | 12.72993 | | RR Ties | | Low Profile |
| 282 | 281 | | 48060.26 | | 2403 | 22.26641 | | 791.4871 | | 20.53974 | | 400 | | 428.1912 | | 10399.7 | | 400 rr tie | | | rr ties | | | 400 rr tie | | 400T RR | | | high | | 8 | 1 | | 11.78021 | | RR Ties | | Low Profile |
| 283 | 282 | | 147364.2 | | 6 | 23.3183 | | 151.4181 | | 13.12767 | | Patch 99 | | 275.534 | | 4359.957 | | mixed patch w/ 1 6 in palet cinderblocks  2 8 in palet cinderblocks  3 pieces concrete highway divider  5 round culverts  low profile chunks of concrete | | | cinderblocks, culverts, highway divider, concrete | | | cinderblocks, culverts, highway divider, concrete | | CB, Culvert, HD, Concrete | | | mid-high | | 9 | 4 | | 12.90066 | | Other / Mixed | | Low Profile |
| 284 | 283 | | 3426824 | | 163032 | 21.30918 | | 380.7305 | | 13.13238 | | RGV Tug | | 153.2792 | | 1220.279 | | boat | | | boat | | | boat | | Boats | | | high | | 10 | 1 | | 16.04714 | | Boat | | Low Profile |
| 285 | 284 | | 6110.945 | | 3 | 20.77509 | | 385.8388 | | 31.09262 | | RGV Tug | | 153.2792 | | 1220.279 | | boat | | | boat | | | boat | | Boats | | | high | | 10 | 1 | | 9.717837 | | Boat | | Low Profile |
| 286 | 285 | | 13589.87 | | 600 | 20.77146 | | 407.1009 | | 31.63719 | | RGV Tug | | 153.2792 | | 1220.279 | | boat | | | boat | | | boat | | Boats | | | high | | 10 | 1 | | 10.51708 | | Boat | | Low Profile |
| 287 | 286 | | 2914.591 | | 146 | 20.98351 | | 363.5635 | | 24.24224 | | RGV Tug | | 153.2792 | | 1220.279 | | boat | | | boat | | | boat | | Boats | | | high | | 10 | 1 | | 8.977485 | | Boat | | Low Profile |
| 288 | 287 | | 2785.22 | | 6 | 21.61177 | | 802.6521 | | 10.33565 | | D-27 | | 1459.357 | | 75504.47 | | Spools, culverts and railroad ties | | | rr ties, spools, culverts | | | RR, Spools, Culvert | | RR, Spools, Culvert | | | mid-high | | 5 | 3 | | 8.932082 | | Other / Mixed | | Low Profile |
| 289 | 288 | | 1548.57 | | 3 | 21.54255 | | 783.896 | | 31.45315 | | D-27 | | 1459.357 | | 75504.47 | | Spools, culverts and railroad ties | | | rr ties, spools, culverts | | | RR, Spools, Culvert | | RR, Spools, Culvert | | | mid-high | | 5 | 3 | | 8.345087 | | Other / Mixed | | Low Profile |
| 290 | 289 | | 50953.79 | | 110 | 21.91304 | | 685.9167 | | 11.05264 | | 3-4 Block7 | | 73.60568 | | 275.3223 | | 3 4 in palet cinderblocks | | | cinderblocks | | | 3 pallets cinderblocks | | 3CB | | | low | | 8 | 1 | | 11.83867 | | Cinderblocks | | Low Profile |
| 291 | 290 | | 3375.075 | | 4 | 21.60652 | | 750.8618 | | 59.43717 | | D-27 | | 1459.357 | | 75504.47 | | Spools, culverts and railroad ties | | | rr ties, spools, culverts | | | RR, Spools, Culvert | | RR, Spools, Culvert | | | mid-high | | 5 | 3 | | 9.124173 | | Other / Mixed | | Low Profile |
| 292 | 291 | | 1063.55 | | 2 | 21.94016 | | 790.6325 | | 71.09982 | | EMR Capt Berry | | 205.433 | | 2309.324 | | boat | | | boat | | | boat | | Boats | | | high | | 6 | 1 | | 7.969368 | | Boat | | Low Profile |
| 293 | 292 | | 67773.2 | | 3389 | 21.59077 | | 825.0619 | | 11.34623 | | SPI Ceviche | | 171.3348 | | 1110.387 | | boat | | | boat | | | boat | | Boats | | | high | | 5 | 1 | | 12.12392 | | Boat | | Low Profile |
| 294 | 293 | | 19424.94 | | 6 | 23.12734 | | 219.0513 | | 27.14785 | | 250-19 | | 398.0899 | | 9756.074 | | 250 ton pile rr ties | | | rr ties | | | 250T RR | | 250T RR | | | mid-high | | 6 | 1 | | 10.87431 | | RR Ties | | Pyramids |
| 295 | 294 | | 14364.25 | | 4 | 23.35354 | | 148.4691 | | 30.94083 | | 250-21 | | 495.6666 | | 13837.4 | | 250 ton pile rr ties | | | rr ties | | | 250T RR | | 250T RR | | | mid-high | | 6 | 1 | | 10.5725 | | RR Ties | | Pyramids |
| 296 | 295 | | 489.5679 | | 12 | 23.32252 | | 171.242 | | 6.913575 | | 250-21 | | 495.6666 | | 13837.4 | | 250 ton pile rr ties | | | rr ties | | | 250T RR | | 250T RR | | | mid-high | | 6 | 1 | | 7.193523 | | RR Ties | | Pyramids |
| 297 | 296 | | 29696.11 | | 26 | 22.50327 | | 541.0446 | | 63.95483 | | 3-6 Block6 | | 274.3334 | | 3209.422 | | 3 6 in pallets cinderblocks | | | cinderblocks | | | 3 pallets cinderblocks | | 3CB | | | low | | 8 | 1 | | 11.29877 | | Cinderblocks | | Pyramids |
| 298 | 297 | | 6069.035 | | 4 | 22.32813 | | 536.4231 | | 41.03135 | | 3-8 Block4 | | 138.789 | | 1294.941 | | 3 8 in pallets cinderblocks | | | cinderblocks | | | 3 pallets cinderblocks | | 3CB | | | low | | 8 | 1 | | 9.710955 | | Cinderblocks | | Pyramids |
| 299 | 298 | | 2537.948 | | 2 | 22.21857 | | 529.7966 | | 94.64346 | | 3-8 Block4 | | 138.789 | | 1294.941 | | 3 8 in pallets cinderblocks | | | cinderblocks | | | 3 pallets cinderblocks | | 3CB | | | low | | 8 | 1 | | 8.839111 | | Cinderblocks | | Pyramids |
| 300 | 299 | | 1807.292 | | 2 | 22.47253 | | 531.5972 | | 43.1678 | | 3mb ridge | | 2104.209 | | 64058.34 | | 3 pallets mixed size cinderblocks in line | | | cinderblocks | | | cinderblock ridge | | CB Ridge | | | low | | 8 | 1 | | 8.499585 | | Cinderblocks | | Pyramids |
| 301 | 300 | | 11834.57 | | 7 | 22.53675 | | 535.8602 | | 90.72564 | | 3-8 Block9 | | 219.28 | | 3020.48 | | 3 8 in pallets cinderblocks | | | cinderblocks | | | 3 pallets cinderblocks | | 3CB | | | low | | 8 | 1 | | 10.37878 | | Cinderblocks | | Pyramids |
| 302 | 301 | | 4104.1 | | 8 | 22.4957 | | 522.0947 | | 89.73976 | | 3-8 Block9 | | 219.28 | | 3020.48 | | 3 8 in pallets cinderblocks | | | cinderblocks | | | 3 pallets cinderblocks | | 3CB | | | low | | 8 | 1 | | 9.319742 | | Cinderblocks | | Pyramids |
| 303 | 302 | | 2327.916 | | 2 | 22.38196 | | 532.6798 | | 34.32121 | | 3mb ridge | | 2104.209 | | 64058.34 | | 3 pallets mixed size cinderblocks in line | | | cinderblocks | | | cinderblock ridge | | CB Ridge | | | low | | 8 | 1 | | 8.752729 | | Cinderblocks | | Pyramids |
| 304 | 303 | | 1980.778 | | 2 | 22.28384 | | 535.9144 | | 33.16585 | | 3-8 Block4 | | 138.789 | | 1294.941 | | 3 8 in pallets cinderblocks | | | cinderblocks | | | 3 pallets cinderblocks | | 3CB | | | low | | 8 | 1 | | 8.591245 | | Cinderblocks | | Pyramids |
| 305 | 304 | | 2871.749 | | 3 | 22.46781 | | 533.5125 | | 24.77176 | | 3mb ridge | | 2104.209 | | 64058.34 | | 3 pallets mixed size cinderblocks in line | | | cinderblocks | | | cinderblock ridge | | CB Ridge | | | low | | 8 | 1 | | 8.962676 | | Cinderblocks | | Pyramids |
| 306 | 305 | | 2248.724 | | 2 | 23.39028 | | 96.00932 | | 28.67343 | | Patch 87 | | 282.7408 | | 4011.823 | | mixed patch w/ 2 8 in palet cinderblocks  low profile chunks of concrete  low profile chunks of concrete | | | cinderblocks, concrete | | | cinderblocks, concrete | | CB, Concrete | | | low | | 9 | 2 | | 8.718118 | | Other / Mixed | | Pyramids |
| 307 | 306 | | 2826.772 | | 2 | 23.44825 | | 120.2889 | | 19.66881 | | Patch 87 | | 282.7408 | | 4011.823 | | mixed patch w/ 2 8 in palet cinderblocks  low profile chunks of concrete  low profile chunks of concrete | | | cinderblocks, concrete | | | cinderblocks, concrete | | CB, Concrete | | | low | | 9 | 2 | | 8.946891 | | Other / Mixed | | Pyramids |
| 308 | 307 | | 16908.3 | | 7 | 23.37787 | | 63.26686 | | 62.71452 | | Patch 87 | | 282.7408 | | 4011.823 | | mixed patch w/ 2 8 in palet cinderblocks  low profile chunks of concrete  low profile chunks of concrete | | | cinderblocks, concrete | | | cinderblocks, concrete | | CB, Concrete | | | low | | 9 | 2 | | 10.73556 | | Other / Mixed | | Pyramids |
| 309 | 308 | | 22907.83 | | 4 | 23.48682 | | 91.49779 | | 24.82428 | | Patch 87 | | 282.7408 | | 4011.823 | | mixed patch w/ 2 8 in palet cinderblocks  low profile chunks of concrete  low profile chunks of concrete | | | cinderblocks, concrete | | | cinderblocks, concrete | | CB, Concrete | | | low | | 9 | 2 | | 11.03923 | | Other / Mixed | | Pyramids |
| 310 | 309 | | 14224.13 | | 5 | 23.33302 | | 74.18584 | | 48.24324 | | Patch 87 | | 282.7408 | | 4011.823 | | mixed patch w/ 2 8 in palet cinderblocks  low profile chunks of concrete  low profile chunks of concrete | | | cinderblocks, concrete | | | cinderblocks, concrete | | CB, Concrete | | | low | | 9 | 2 | | 10.5627 | | Other / Mixed | | Pyramids |
| 311 | 310 | | 11029.23 | | 7 | 22.62274 | | 288.1559 | | 9.299261 | | Patch 83 | | 261.7858 | | 4026.786 | | mixed patch w/ 1 6 in palet cinderblocks  2 8 in palet cinderblocks  2 10x10 box culverts  3 pieces concrete highway divider | | | cinderblocks, culverts, highway divider | | | cinderblocks, culverts, highway divider | | CB, Culvert, HD | | | mid-high | | 9 | 3 | | 10.3083 | | Other / Mixed | | Pyramids |
| 312 | 311 | | 67964.11 | | 4 | 22.98248 | | 137.0918 | | 5.80018 | | Patch 100 | | 235.7576 | | 3644.533 | | mixed patch w/ 1 6 in palet cinderblocks, 2 8 in palet cinderblocks , 2 10x10 box culverts, 3 pieces concrete highway divider, low profile chunks of concrete | | | cinderblocks, culverts, highway divider, concrete | | | cinderblocks, culverts, highway divider, concrete | | CB, Culvert, HD, Concrete | | | mid-high | | 9 | 4 | | 12.12674 | | Other / Mixed | | Pyramids |
| 313 | 312 | | 9303.93 | | 2 | 22.95559 | | 128.9934 | | 25.58023 | | Patch 100 | | 235.7576 | | 3644.533 | | mixed patch w/ 1 6 in palet cinderblocks, 2 8 in palet cinderblocks , 2 10x10 box culverts, 3 pieces concrete highway divider, low profile chunks of concrete | | | cinderblocks, culverts, highway divider, concrete | | | cinderblocks, culverts, highway divider, concrete | | CB, Culvert, HD, Concrete | | | mid-high | | 9 | 4 | | 10.13819 | | Other / Mixed | | Pyramids |
| 314 | 313 | | 13988.95 | | 2 | 20.98819 | | 619.5575 | | 14.29 | | Patch 72 | | 222.1526 | | 2646.466 | | mixed patch w/ 1 6 in palet cinderblocks  2 8 in palet cinderblocks  2 10x10 box culverts  3 pieces concrete highway divider  low profile chunks of concrete | | | cinderblocks, culverts, highway divider, concrete | | | cinderblocks, culverts, highway divider, concrete | | CB, Culvert, HD, Concrete | | | mid-high | | 9 | 4 | | 10.54602 | | Other / Mixed | | Pyramids |
| 315 | 314 | | 12253.12 | | 2 | 21.25929 | | 402.2882 | | 38.66937 | | Patch 74 | | 229.2741 | | 3617.252 | | mixed patch w/ 2 8 in palet cinderblocks  2 10x10 box culverts  3 pieces concrete highway divider  low profile chunks of concrete  low profile chunks of concrete | | | cinderblocks, culverts, highway divider, concrete | | | cinderblocks, culverts, highway divider, concrete | | CB, Culvert, HD, Concrete | | | mid-high | | 9 | 4 | | 10.41354 | | Other / Mixed | | Pyramids |
| 316 | 315 | | 10851.01 | | 2 | 21.55938 | | 414.9924 | | 8.320821 | | Patch 74 | | 229.2741 | | 3617.252 | | mixed patch w/ 2 8 in palet cinderblocks  2 10x10 box culverts  3 pieces concrete highway divider  low profile chunks of concrete  low profile chunks of concrete | | | cinderblocks, culverts, highway divider, concrete | | | cinderblocks, culverts, highway divider, concrete | | CB, Culvert, HD, Concrete | | | mid-high | | 9 | 4 | | 10.29201 | | Other / Mixed | | Pyramids |
| 317 | 316 | | 32710.96 | | 7 | 21.74982 | | 281.6994 | | 12.42653 | | Patch 77 | | 311.3754 | | 5600.178 | | mixed patch w/ 1 6 in palet cinderblocks  2 8 in palet cinderblocks  2 10x10 box culverts  3 pieces concrete highway divider  low profile chunks of concrete | | | cinderblocks, culverts, highway divider, concrete | | | cinderblocks, culverts, highway divider, concrete | | CB, Culvert, HD, Concrete | | | mid-high | | 9 | 4 | | 11.39547 | | Other / Mixed | | Pyramids |
| 318 | 317 | | 956.9973 | | 6 | 21.76919 | | 279.0206 | | 16.0382 | | Patch 78 | | 340.1461 | | 6208.392 | | mixed patch w/ 1 6 in palet cinderblocks  2 8 in palet cinderblocks  2 10x10 box culverts  3 pieces concrete highway divider  low profile chunks of concrete | | | cinderblocks, culverts, highway divider, concrete | | | cinderblocks, culverts, highway divider, concrete | | CB, Culvert, HD, Concrete | | | mid-high | | 9 | 4 | | 7.863801 | | Other / Mixed | | Pyramids |
| 319 | 318 | | 4083.683 | | 2 | 22.796 | | 289.7826 | | 19.014 | | Patch 85 | | 137.7693 | | 1059.623 | | mixed patch w/ 1 6 in palet cinderblocks  2 8 in palet cinderblocks  2 10x10 box culverts  low profile chunks of concrete  low profile chunks of concrete | | | cinderblocks, culverts, highway divider, concrete | | | cinderblocks, culverts, highway divider, concrete | | CB, Culvert, HD, Concrete | | | mid-high | | 9 | 4 | | 9.314755 | | Other / Mixed | | Pyramids |
| 320 | 319 | | 22148.67 | | 5 | 23.15577 | | 163.9655 | | 18.98356 | | Patch 89 | | 246.0308 | | 3705.774 | | mixed patch w/ 2 8 in palet cinderblocks  3 pieces concrete highway divider  5 round culverts  low profile chunks of concrete | | | cinderblocks, culverts, highway divider, concrete | | | cinderblocks, culverts, highway divider, concrete | | CB, Culvert, HD, Concrete | | | mid-high | | 9 | 4 | | 11.00553 | | Other / Mixed | | Pyramids |
| 321 | 320 | | 5355.658 | | 2 | 23.15775 | | 170.5158 | | 21.76171 | | Patch 90 | | 112.6175 | | 860.7581 | | mixed patch w/ 2 8 in palet cinderblocks   low profile chunks of concrete   concrete watermill structure | | | cinderblocks, water mill, concrete | | | cinderblocks, water mill, concrete | | CB, WM, Concrete | | | mid-high | | 9 | 3 | | 9.585909 | | Other / Mixed | | Pyramids |
| 322 | 321 | | 18173.37 | | 4 | 20.83758 | | 255.0946 | | 12.18033 | | Patch 52 | | 561.5502 | | 18916.29 | | mixed patch w/ 1 8 in palet cinderblocks  1 8 in palet cinderblocks  4 pyramids | | | cinderblocks, pyramids | | | cinderblocks, pyramids | | CB, PY | | | mid | | 9 | 2 | | 10.80771 | | Pyramids | | Pyramids |
| 323 | 322 | | 1465 | | 4 | 20.91657 | | 249.761 | | 16.48016 | | Patch 52 | | 561.5502 | | 18916.29 | | mixed patch w/ 1 8 in palet cinderblocks  1 8 in palet cinderblocks  4 pyramids | | | cinderblocks, pyramids | | | cinderblocks, pyramids | | CB, PY | | | mid | | 9 | 2 | | 8.28961 | | Pyramids | | Pyramids |
| 324 | 323 | | 7205.235 | | 9 | 20.67612 | | 223.8532 | | 40.68488 | | Patch 52 | | 561.5502 | | 18916.29 | | mixed patch w/ 1 8 in palet cinderblocks  1 8 in palet cinderblocks  4 pyramids | | | cinderblocks, pyramids | | | cinderblocks, pyramids | | CB, PY | | | mid | | 9 | 2 | | 9.882563 | | Pyramids | | Pyramids |
| 325 | 324 | | 10670.22 | | 3 | 21.17388 | | 437.4353 | | 26.82792 | | Patch 55 | | 516.0461 | | 18359.74 | | mixed patch w/ 1 8 in palet cinderblocks  1 8 in palet cinderblocks  4 pyramids | | | cinderblocks, pyramids | | | cinderblocks, pyramids | | CB, PY | | | mid | | 9 | 2 | | 10.27521 | | Pyramids | | Pyramids |
| 326 | 325 | | 3134.872 | | 2 | 21.10765 | | 622.7036 | | 8.10423 | | Patch 56 | | 310.139 | | 5748.809 | | mixed patch w/ 1 8 in palet cinderblocks  1 8 in palet cinderblocks  4 pyramids | | | cinderblocks, pyramids | | | cinderblocks, pyramids | | CB, PY | | | mid | | 9 | 2 | | 9.050344 | | Pyramids | | Pyramids |
| 327 | 326 | | 2945.312 | | 2 | 21.09039 | | 909.9143 | | 10.66916 | | Patch 60 | | 584.4798 | | 20123.45 | | mixed patch w/ 1 8 in palet cinderblocks  1 8 in palet cinderblocks  4 pyramids | | | cinderblocks, pyramids | | | cinderblocks, pyramids | | CB, PY | | | mid | | 9 | 2 | | 8.98797 | | Pyramids | | Pyramids |
| 328 | 327 | | 5047.016 | | 2 | 21.20092 | | 929.9215 | | 17.7798 | | Patch 60 | | 584.4798 | | 20123.45 | | mixed patch w/ 1 8 in palet cinderblocks  1 8 in palet cinderblocks  4 pyramids | | | cinderblocks, pyramids | | | cinderblocks, pyramids | | CB, PY | | | mid | | 9 | 2 | | 9.526553 | | Pyramids | | Pyramids |
| 329 | 328 | | 1137.493 | | 2 | 23.48107 | | 125.2894 | | 22.62728 | | Patch 88 | | 84.63844 | | 476.493 | | mixed patch w/ 2 8 in palet cinderblocks  low profile chunks of concrete  concrete watermill structure | | | cinderblocks, water mill, concrete | | | cinderblocks, water mill, concrete | | CB, WM, Concrete | | | mid-high | | 9 | 3 | | 8.036582 | | Other / Mixed | | Pyramids |
| 330 | 329 | | 12552.94 | | 9 | 20.96092 | | 105.6271 | | 5.99165 | | Hwy Dividers1 | | 120.2711 | | 586.2387 | | 3 pieces concrete highway divider, 60 tons rr ties | | | highway divider, rr ties | | | highway divider, rr ties | | HD, RR | | | mid | | 9 | 2 | | 10.43771 | | RR Ties | | Pyramids |
| 331 | 330 | | 5354.25 | | 2 | 22.37201 | | 106.4096 | | 11.47556 | | Hwy Dividers33 | | 148.5237 | | 726.2203 | | 3 pieces concrete highway divider, 60 tons rr ties | | | highway divider, rr ties | | | highway divider, rr ties | | HD, RR | | | mid | | 9 | 2 | | 9.585646 | | RR Ties | | Pyramids |
| 332 | 331 | | 4776.368 | | 2 | 22.33947 | | 104.2276 | | 22.72815 | | Hwy Dividers33 | | 148.5237 | | 726.2203 | | 3 pieces concrete highway divider, 60 tons rr ties | | | highway divider, rr ties | | | highway divider, rr ties | | HD, RR | | | mid | | 9 | 2 | | 9.471436 | | RR Ties | | Pyramids |
| 333 | 332 | | 3646.884 | | 2 | 22.49562 | | 118.9109 | | 26.76039 | | Hwy Dividers 34 and 35 | | 232.4753 | | 2215.741 | | 6 pieces concrete highway divider, 60 tons rr ties | | | highway divider, rr ties | | | highway divider, rr ties | | HD, RR | | | mid | | 9 | 2 | | 9.201628 | | RR Ties | | Pyramids |
| 334 | 333 | | 1663.532 | | 2 | 21.05696 | | 101.5167 | | 5.933571 | | Hwy Dividers5 | | 167.3844 | | 1641.49 | | 3 pieces concrete highway divider, 60 tons rr ties | | | highway divider, rr ties | | | highway divider, rr ties | | HD, RR | | | mid | | 9 | 2 | | 8.416698 | | RR Ties | | Pyramids |
| 335 | 334 | | 11978.98 | | 4 | 22.66594 | | 109.4837 | | 6.096111 | | Hwy Dividers11 | | 121.8719 | | 955.7722 | | 3 pieces concrete highway divider, 60 tons rr ties, 10 tons concrete | | | highway divider, rr ties, concrete | | | highway divider, rr ties, concrete | | HD, RR, Concrete | | | mid | | 9 | 3 | | 10.39091 | | RR Ties | | Pyramids |
| 336 | 335 | | 659.984 | | 2 | 21.59699 | | 597.6025 | | 4.410977 | | LS15 | | 29.67278 | | 59.58879 | | One Gulf LP | | | limestone | | | limestone | | Limestone | | | low | | 8 | 1 | | 7.492216 | | Concrete | | Pyramids |
| 337 | 336 | | 4641.395 | | 2 | 21.51233 | | 785.0527 | | 22.5861 | | Octoreef4 | | 38.33831 | | 88.26445 | | octoreef | | | octoreef | | | octoreef | | Octoreef | | | mid | | 9 | 1 | | 9.44277 | | Pyramids | | Pyramids |
| 338 | 337 | | 1138.785 | | 3 | 22.64351 | | 600.2401 | | 86.32101 | | 3-6 Block5 | | 42.86508 | | 123.3765 | | 3 6 in palet cinderblocks | | | cinderblocks | | | 3 pallets cinderblocks | | 3CB | | | low | | 8 | 1 | | 8.037717 | | Cinderblocks | | Pyramids |
| 339 | 338 | | 67330.97 | | 30 | 22.47039 | | 735.5898 | | 39.06325 | | rr tie ridge | | 2402.195 | | 49406.01 | | rr tie ridge | | | rr tie ridge | | | rr tie ridge | | RR Ridge | | | mid | | 8 | 1 | | 12.11738 | | RR Ties | | Pyramids |
| 340 | 339 | | 37936.39 | | 24 | 22.7271 | | 641.1412 | | 81.26042 | | 3-6 Block5 | | 42.86508 | | 123.3765 | | 3 6 in palet cinderblocks | | | cinderblocks | | | 3 pallets cinderblocks | | 3CB | | | low | | 8 | 1 | | 11.54367 | | Cinderblocks | | Pyramids |
| 341 | 340 | | 110388.2 | | 406 | 22.12668 | | 750.069 | | 83.36627 | | 3-mixed Block17 | | 145.0921 | | 1384.376 | | 3 pallets mixed size cinderblocks | | | cinderblocks | | | 3 pallets cinderblocks | | 3CB | | | low | | 8 | 1 | | 12.61176 | | Cinderblocks | | Pyramids |
| 342 | 341 | | 3882.622 | | 2 | 22.37615 | | 760.2034 | | 17.15174 | | rr tie ridge | | 2402.195 | | 49406.01 | | rr tie ridge | | | rr tie ridge | | | rr tie ridge | | RR Ridge | | | mid | | 8 | 1 | | 9.264266 | | RR Ties | | Pyramids |
| 343 | 342 | | 39138.46 | | 11 | 22.59997 | | 701.9136 | | 71.77034 | | rr tie ridge | | 2402.195 | | 49406.01 | | rr tie ridge | | | rr tie ridge | | | rr tie ridge | | RR Ridge | | | mid | | 8 | 1 | | 11.57486 | | RR Ties | | Pyramids |
| 344 | 343 | | 1968.805 | | 2 | 22.19851 | | 746.9513 | | 80.8617 | | 3-8 Block5 | | 181.4628 | | 1507.007 | | 3 8 in palet cinderblocks | | | cinderblocks | | | 3 pallets cinderblocks | | 3CB | | | low | | 8 | 1 | | 8.585182 | | Cinderblocks | | Pyramids |
| 345 | 344 | | 7078.94 | | 2 | 22.49778 | | 725.9309 | | 47.14043 | | rr tie ridge | | 2402.195 | | 49406.01 | | rr tie ridge | | | rr tie ridge | | | rr tie ridge | | RR Ridge | | | mid | | 8 | 1 | | 9.864879 | | RR Ties | | Pyramids |
| 346 | 345 | | 26164.47 | | 8 | 22.42343 | | 749.9895 | | 3.239309 | | rr tie ridge | | 2402.195 | | 49406.01 | | rr tie ridge | | | rr tie ridge | | | rr tie ridge | | RR Ridge | | | mid | | 8 | 1 | | 11.17216 | | RR Ties | | Pyramids |
| 347 | 346 | | 2114.738 | | 5 | 23.01328 | | 231.2261 | | 22.2638 | | D-16 | | 527.3876 | | 16841.85 | | Concrete Railroad Ties | | | rr ties | | | RR Ties | | RR Ties | | | mid | | 5 | 1 | | 8.656686 | | RR Ties | | Pyramids |
| 348 | 347 | | 4812.063 | | 2 | 21.62862 | | 609.9863 | | 8.821921 | | 100tonmixed13 | | 325.5087 | | 4821.023 | | 75 ton ties, 25 tons broken concrete | | | rr ties, concrete | | | RR, Concrete | | RR, Concrete | | | mid | | 6 | 2 | | 9.478881 | | RR Ties | | Pyramids |
| 349 | 348 | | 23097.23 | | 48 | 21.61459 | | 604.0036 | | 7.669202 | | 100tonmixed14 | | 411.9476 | | 6941.372 | | 75 ton ties, 25 tons broken concrete | | | rr ties, concrete | | | RR, Concrete | | RR, Concrete | | | mid | | 6 | 2 | | 11.04747 | | RR Ties | | Pyramids |
| 350 | 349 | | 7919.958 | | 4 | 21.68665 | | 568.2499 | | 2.61996 | | 100tonmixed15 | | 332.3616 | | 3493.598 | | 75 ton ties, 25 tons broken concrete | | | rr ties, concrete | | | RR, Concrete | | RR, Concrete | | | mid | | 6 | 2 | | 9.977141 | | RR Ties | | Pyramids |
| 351 | 350 | | 6296.307 | | 2 | 21.68946 | | 553.2343 | | 11.59753 | | 100tonmixed17 | | 317.462 | | 4789.935 | | 75 ton ties, 25 tons broken concrete | | | rr ties, concrete | | | RR, Concrete | | RR, Concrete | | | mid | | 6 | 2 | | 9.747719 | | RR Ties | | Pyramids |
| 352 | 351 | | 32508.72 | | 12 | 21.44508 | | 527.7536 | | 17.04859 | | 100tonmixed17 | | 317.462 | | 4789.935 | | 75 ton ties, 25 tons broken concrete | | | rr ties, concrete | | | RR, Concrete | | RR, Concrete | | | mid | | 6 | 2 | | 11.38926 | | RR Ties | | Pyramids |
| 353 | 352 | | 6596.446 | | 4 | 21.52578 | | 524.4203 | | 11.17369 | | 100tonmixed18 | | 310.5831 | | 5094.639 | | 75 ton ties, 25 tons broken concrete | | | rr ties, concrete | | | RR, Concrete | | RR, Concrete | | | mid | | 6 | 2 | | 9.794286 | | RR Ties | | Pyramids |
| 354 | 353 | | 4533.372 | | 3 | 22.02869 | | 272.3282 | | 4.612376 | | 100tonmixed20 | | 234.7018 | | 2328.458 | | 75 ton ties, 25 tons broken concrete | | | rr ties, concrete | | | RR, Concrete | | RR, Concrete | | | mid | | 6 | 2 | | 9.419221 | | RR Ties | | Cinderblocks |
| 355 | 354 | | 13925.38 | | 3 | 23.09474 | | 171.498 | | 19.34938 | | Mix_40 | | 353.9422 | | 5332.888 | | 25 ton RR Ties, 25 ton Concrete, 6 Pallets CinderBlock | | | rr ties, concrete, cinderblocks | | | RR, Concrete, CB | | RR, Concrete, CB | | | mid | | 6 | 3 | | 10.54147 | | RR Ties | | Cinderblocks |
| 356 | 355 | | 19711.26 | | 33 | 21.10933 | | 761.9293 | | 17.65931 | | 100tonmixed5 | | 293.9717 | | 4327.636 | | 75 ton ties, 25 tons broken concrete | | | rr ties, concrete | | | RR, Concrete | | RR, Concrete | | | mid | | 6 | 2 | | 10.88895 | | RR Ties | | Cinderblocks |
| 357 | 356 | | 13430.28 | | 10 | 21.20124 | | 758.2351 | | 19.88699 | | 100tonmixed6 | | 287.7827 | | 4824.275 | | 75 ton ties, 25 tons broken concrete | | | rr ties, concrete | | | RR, Concrete | | RR, Concrete | | | mid | | 6 | 2 | | 10.50527 | | RR Ties | | Cinderblocks |
| 358 | 357 | | 22559.69 | | 261 | 20.90285 | | 253.4362 | | 5.081272 | | Mix_1 | | 177.5809 | | 1787.628 | | 25 ton RR Ties, 25 ton Concrete, 6 Pallets CinderBlock | | | rr ties, concrete, cinderblocks | | | RR, Concrete, CB | | RR, Concrete, CB | | | mid | | 6 | 3 | | 11.02392 | | RR Ties | | Cinderblocks |
| 359 | 358 | | 4311.209 | | 4 | 22.32819 | | 216.7143 | | 26.18001 | | Mix_11 | | 328.3018 | | 5522.462 | | 25 ton RR Ties, 25 ton Concrete, 6 Pallets CinderBlock | | | rr ties, concrete, cinderblocks | | | RR, Concrete, CB | | RR, Concrete, CB | | | mid | | 6 | 3 | | 9.368974 | | RR Ties | | Pyramids |
| 360 | 359 | | 8473.773 | | 4 | 22.59768 | | 199.8503 | | 2.624564 | | Mix_13 | | 215.4591 | | 1983.282 | | 25 ton RR Ties, 25 ton Concrete, 6 Pallets CinderBlock | | | rr ties, concrete, cinderblocks | | | RR, Concrete, CB | | RR, Concrete, CB | | | mid | | 6 | 3 | | 10.04473 | | RR Ties | | Pyramids |
| 361 | 360 | | 7899.597 | | 6 | 22.75607 | | 203.3164 | | 1.489596 | | Mix_14 | | 323.4378 | | 4433.64 | | 25 ton RR Ties, 25 ton Concrete, 6 Pallets CinderBlock | | | rr ties, concrete, cinderblocks | | | RR, Concrete, CB | | RR, Concrete, CB | | | mid | | 6 | 3 | | 9.974567 | | RR Ties | | Pyramids |
| 362 | 361 | | 2086.339 | | 2 | 22.74004 | | 202.0397 | | 22.32298 | | Mix_14 | | 323.4378 | | 4433.64 | | 25 ton RR Ties, 25 ton Concrete, 6 Pallets CinderBlock | | | rr ties, concrete, cinderblocks | | | RR, Concrete, CB | | RR, Concrete, CB | | | mid | | 6 | 3 | | 8.643166 | | RR Ties | | Pyramids |
| 363 | 362 | | 31626.93 | | 9 | 23.1205 | | 201.1314 | | 9.925475 | | Mix_16 | | 338.3012 | | 3776.111 | | 25 ton RR Ties, 25 ton Concrete, 6 Pallets CinderBlock | | | rr ties, concrete, cinderblocks | | | RR, Concrete, CB | | RR, Concrete, CB | | | mid | | 6 | 3 | | 11.36176 | | RR Ties | | Pyramids |
| 364 | 363 | | 12102.91 | | 185 | 21.15412 | | 269.5541 | | 6.600957 | | Mix_2 | | 207.8502 | | 2713.89 | | 25 ton RR Ties, 25 ton Concrete, 6 Pallets CinderBlock | | | rr ties, concrete, cinderblocks | | | RR, Concrete, CB | | RR, Concrete, CB | | | mid | | 6 | 3 | | 10.4012 | | RR Ties | | Pyramids |
| 365 | 364 | | 35679.65 | | 1540 | 21.17201 | | 259.3551 | | 4.888341 | | Mix_2 | | 207.8502 | | 2713.89 | | 25 ton RR Ties, 25 ton Concrete, 6 Pallets CinderBlock | | | rr ties, concrete, cinderblocks | | | RR, Concrete, CB | | RR, Concrete, CB | | | mid | | 6 | 3 | | 11.48234 | | RR Ties | | Pyramids |
| 366 | 365 | | 9358.121 | | 3 | 22.81305 | | 382.1696 | | 2.70986 | | Mix_24 | | 358.614 | | 6890.864 | | 25 ton RR Ties, 25 ton Concrete, 6 Pallets CinderBlock | | | rr ties, concrete, cinderblocks | | | RR, Concrete, CB | | RR, Concrete, CB | | | mid | | 6 | 3 | | 10.144 | | RR Ties | | Pyramids |
| 367 | 366 | | 9469.379 | | 4 | 22.57276 | | 653.4264 | | 7.422404 | | Mix_25 | | 280.2439 | | 4038.682 | | 25 ton RR Ties, 25 ton Concrete, 6 Pallets CinderBlock | | | rr ties, concrete, cinderblocks | | | RR, Concrete, CB | | RR, Concrete, CB | | | mid | | 6 | 3 | | 10.15582 | | RR Ties | | Pyramids |
| 368 | 367 | | 18894.04 | | 10 | 22.26853 | | 571.09 | | 2.683134 | | Mix_27 | | 280.7085 | | 3356.455 | | 25 ton RR Ties, 25 ton Concrete, 6 Pallets CinderBlock | | | rr ties, concrete, cinderblocks | | | RR, Concrete, CB | | RR, Concrete, CB | | | mid | | 6 | 3 | | 10.8466 | | RR Ties | | Pyramids |
| 369 | 368 | | 4020.891 | | 2 | 21.88666 | | 661.2387 | | 5.917958 | | Mix_28 | | 343.8062 | | 3553.502 | | 25 ton RR Ties, 25 ton Concrete, 6 Pallets CinderBlock | | | rr ties, concrete, cinderblocks | | | RR, Concrete, CB | | RR, Concrete, CB | | | mid | | 6 | 3 | | 9.299259 | | RR Ties | | Pyramids |
| 370 | 369 | | 18446.09 | | 4 | 21.02435 | | 175.4499 | | 21.96806 | | Mix_29 | | 322.2633 | | 5654.458 | | 25 ton RR Ties, 25 ton Concrete, 6 Pallets CinderBlock | | | rr ties, concrete, cinderblocks | | | RR, Concrete, CB | | RR, Concrete, CB | | | mid | | 6 | 3 | | 10.82261 | | RR Ties | | Pyramids |
| 371 | 370 | | 14506.21 | | 669 | 21.3049 | | 196.3962 | | 8.485554 | | Mix_29 | | 322.2633 | | 5654.458 | | 25 ton RR Ties, 25 ton Concrete, 6 Pallets CinderBlock | | | rr ties, concrete, cinderblocks | | | RR, Concrete, CB | | RR, Concrete, CB | | | mid | | 6 | 3 | | 10.58233 | | RR Ties | | Pyramids |
| 372 | 371 | | 12536.85 | | 3 | 21.22549 | | 274.3234 | | 15.34899 | | Extra rr ties 1 | | 135.3473 | | 811.1455 | | RR ties | | | rr ties | | | RR | | Extra RR | | | mid | | 6 | 1 | | 10.43643 | | RR Ties | | Pyramids |
| 373 | 372 | | 39652.92 | | 9 | 21.31565 | | 274.8885 | | 2.041799 | | Mix_3 | | 138.3346 | | 1324.907 | | 25 ton RR Ties, 25 ton Concrete, 6 Pallets CinderBlock | | | rr ties, concrete, cinderblocks | | | RR, Concrete, CB | | RR, Concrete, CB | | | mid | | 6 | 3 | | 11.58792 | | RR Ties | | Pyramids |
| 374 | 373 | | 89001.46 | | 10 | 21.21416 | | 272.3918 | | 17.29396 | | Mix_3 | | 138.3346 | | 1324.907 | | 25 ton RR Ties, 25 ton Concrete, 6 Pallets CinderBlock | | | rr ties, concrete, cinderblocks | | | RR, Concrete, CB | | RR, Concrete, CB | | | mid | | 6 | 3 | | 12.39641 | | RR Ties | | Pyramids |
| 375 | 374 | | 4638.421 | | 2 | 21.08952 | | 184.428 | | 14.74264 | | Mix_30 | | 177.1633 | | 1493.936 | | 25 ton RR Ties, 25 ton Concrete, 6 Pallets CinderBlock | | | rr ties, concrete, cinderblocks | | | RR, Concrete, CB | | RR, Concrete, CB | | | mid | | 6 | 3 | | 9.442129 | | RR Ties | | Pyramids |
| 376 | 375 | | 11727.44 | | 263 | 21.37117 | | 191.6629 | | 10.1487 | | Mix_31 | | 226.9695 | | 1748.217 | | 25 ton RR Ties, 25 ton Concrete, 6 Pallets CinderBlock | | | rr ties, concrete, cinderblocks | | | RR, Concrete, CB | | RR, Concrete, CB | | | mid | | 6 | 3 | | 10.36969 | | RR Ties | | Pyramids |
| 377 | 376 | | 912.2959 | | 2 | 21.66019 | | 190.4293 | | 22.92179 | | Mix_34 | | 245.0884 | | 2582.158 | | 25 ton RR Ties, 25 ton Concrete, 6 Pallets CinderBlock | | | rr ties, concrete, cinderblocks | | | RR, Concrete, CB | | RR, Concrete, CB | | | mid | | 6 | 3 | | 7.815964 | | RR Ties | | Pyramids |
| 378 | 377 | | 635.0437 | | 2 | 22.04487 | | 186.2839 | | 6.871061 | | Mix_38 | | 264.2196 | | 3112.755 | | 25 ton RR Ties, 25 ton Concrete, 6 Pallets CinderBlock | | | rr ties, concrete, cinderblocks | | | RR, Concrete, CB | | RR, Concrete, CB | | | mid | | 6 | 3 | | 7.453694 | | RR Ties | | Pyramids |
| 379 | 378 | | 17326.17 | | 13 | 22.97652 | | 242.5356 | | 2.170632 | | Mix_39 | | 191.8251 | | 1821.718 | | 25 ton RR Ties, 25 ton Concrete, 6 Pallets CinderBlock | | | rr ties, concrete, cinderblocks | | | RR, Concrete, CB | | RR, Concrete, CB | | | mid | | 6 | 3 | | 10.75997 | | RR Ties | | Pyramids |
| 380 | 379 | | 15314.8 | | 13 | 21.28581 | | 234.3441 | | 6.116024 | | Mix_4 | | 171.9907 | | 2088.396 | | 25 ton RR Ties, 25 ton Concrete, 6 Pallets CinderBlock | | | rr ties, concrete, cinderblocks | | | RR, Concrete, CB | | RR, Concrete, CB | | | mid | | 6 | 3 | | 10.63658 | | RR Ties | | Pyramids |
| 381 | 380 | | 108196.8 | | 16 | 23.12176 | | 153.1841 | | 7.83989 | | Mix_40 | | 353.9422 | | 5332.888 | | 25 ton RR Ties, 25 ton Concrete, 6 Pallets CinderBlock | | | rr ties, concrete, cinderblocks | | | RR, Concrete, CB | | RR, Concrete, CB | | | mid | | 6 | 3 | | 12.59171 | | RR Ties | | Pyramids |
| 382 | 381 | | 2001.143 | | 3 | 22.50602 | | 286.3572 | | 21.12411 | | Mix_41 | | 239.3908 | | 2932.138 | | 25 ton RR Ties, 25 ton Concrete, 6 Pallets CinderBlock | | | rr ties, concrete, cinderblocks | | | RR, Concrete, CB | | RR, Concrete, CB | | | mid | | 6 | 3 | | 8.601474 | | RR Ties | | Pyramids |
| 383 | 382 | | 17527.46 | | 8 | 22.7754 | | 285.1299 | | 4.322062 | | Mix_42 | | 221.5067 | | 2829.814 | | 25 ton RR Ties, 25 ton Concrete, 6 Pallets CinderBlock | | | rr ties, concrete, cinderblocks | | | RR, Concrete, CB | | RR, Concrete, CB | | | mid | | 6 | 3 | | 10.77152 | | RR Ties | | Pyramids |
| 384 | 383 | | 5444.936 | | 2 | 23.14959 | | 199.7843 | | 6.084308 | | Mix_44 | | 330.685 | | 5537.713 | | 25 ton RR Ties, 25 ton Concrete, 6 Pallets CinderBlock | | | rr ties, concrete, cinderblocks | | | RR, Concrete, CB | | RR, Concrete, CB | | | mid | | 6 | 3 | | 9.602441 | | RR Ties | | Pyramids |
| 385 | 384 | | 29499.13 | | 12 | 22.96104 | | 254.3363 | | 9.145721 | | Mix_45 | | 281.2536 | | 4210.47 | | 25 ton RR Ties, 25 ton Concrete, 6 Pallets CinderBlock | | | rr ties, concrete, cinderblocks | | | RR, Concrete, CB | | RR, Concrete, CB | | | mid | | 6 | 3 | | 11.29212 | | RR Ties | | Pyramids |
| 386 | 385 | | 18280.13 | | 333 | 21.63622 | | 232.9968 | | 7.436568 | | Mix_5 | | 216.2756 | | 2299.653 | | 25 ton RR Ties, 25 ton Concrete, 6 Pallets CinderBlock | | | rr ties, concrete, cinderblocks | | | RR, Concrete, CB | | RR, Concrete, CB | | | mid | | 6 | 3 | | 10.81357 | | RR Ties | | Pyramids |
| 387 | 386 | | 28583.98 | | 8 | 21.69022 | | 273.3839 | | 9.910655 | | Mix_6 | | 200.4879 | | 2070.031 | | 25 ton RR Ties, 25 ton Concrete, 6 Pallets CinderBlock | | | rr ties, concrete, cinderblocks | | | RR, Concrete, CB | | RR, Concrete, CB | | | mid | | 6 | 3 | | 11.2606 | | RR Ties | | Pyramids |
| 388 | 387 | | 102902.3 | | 3 | 22.83786 | | 401.9798 | | 8.188541 | | D-10 | | 898.0723 | | 48165.23 | | 1 large spool 300 ties | | | rr ties, spools | | | RR, Spools | | RR, Spools | | | mid-high | | 5 | 2 | | 12.54154 | | Other / Mixed | | Pyramids |
| 389 | 388 | | 143850.4 | | 7193 | 22.60737 | | 478.1586 | | 12.22276 | | D-11 | | 810.1454 | | 38470.51 | | 1 large spool 300 ties | | | rr ties, spools | | | RR, Spools | | RR, Spools | | | mid-high | | 5 | 2 | | 12.87653 | | Other / Mixed | | Pyramids |
| 390 | 389 | | 43096.03 | | 8 | 22.6115 | | 495.0398 | | 9.340111 | | D-2 | | 699.6385 | | 27098.22 | | 1 large spool 400 ties | | | rr ties, spools | | | RR, Spools | | RR, Spools | | | mid-high | | 5 | 2 | | 11.67119 | | Other / Mixed | | Pyramids |
| 391 | 390 | | 21195.17 | | 5 | 23.29484 | | 128.248 | | 2.565507 | | D-20 | | 455.1934 | | 10831.77 | | Spools, concrete railroad ties | | | rr ties, spools, concrete | | | RR, Spools, Concrete | | RR, Spools, Concrete | | | mid-high | | 5 | 3 | | 10.96153 | | Other / Mixed | | Pyramids |
| 392 | 391 | | 69573.91 | | 9 | 23.18198 | | 210.6305 | | 11.20238 | | D-22 | | 446.7276 | | 12921.52 | | Spools, concrete railroad ties | | | rr ties, spools | | | RR, Spools | | RR, Spools | | | mid-high | | 5 | 2 | | 12.15014 | | Other / Mixed | | Pyramids |
| 393 | 392 | | 8836.447 | | 4 | 22.95683 | | 393.3551 | | 30.93344 | | D-3 | | 882.0725 | | 34901.06 | | 3 medium spools, 30 small spools. railroad ties | | | rr ties, spools | | | RR, Spools | | RR, Spools | | | mid-high | | 5 | 2 | | 10.08664 | | Other / Mixed | | Pyramids |
| 394 | 393 | | 4519.431 | | 3 | 22.91621 | | 378.6715 | | 39.61569 | | D-3 | | 882.0725 | | 34901.06 | | 3 medium spools, 30 small spools. railroad ties | | | rr ties, spools | | | RR, Spools | | RR, Spools | | | mid-high | | 5 | 2 | | 9.416141 | | Other / Mixed | | Pyramids |
| 395 | 394 | | 68004.66 | | 2 | 22.56912 | | 571 | | 13.98309 | | D-1 | | 857.3153 | | 41943.89 | | Large Spool, broken ties, 15ft Spools | | | rr ties, spools, concrete | | | RR, Spools, Concrete | | RR, Spools, Concrete | | | mid-high | | 5 | 3 | | 12.12733 | | Other / Mixed | | Pyramids |
| 396 | 395 | | 13175.01 | | 2 | 22.61471 | | 587.9898 | | 8.079965 | | D-1 | | 857.3153 | | 41943.89 | | Large Spool, broken ties, 15ft Spools | | | rr ties, spools, concrete | | | RR, Spools, Concrete | | RR, Spools, Concrete | | | mid-high | | 5 | 3 | | 10.48608 | | Other / Mixed | | Pyramids |
| 397 | 396 | | 34581.36 | | 167 | 23.30604 | | 124.4149 | | 1.095587 | | D-21 | | 566.7995 | | 13511.86 | | Spools, concrete railroad ties | | | rr ties, spools, concrete | | | RR, Spools, Concrete | | RR, Spools, Concrete | | | mid-high | | 5 | 3 | | 11.45107 | | Other / Mixed | | Pyramids |
| 398 | 397 | | 45799.88 | | 8 | 23.24887 | | 142.1649 | | 20.6188 | | D-21 | | 566.7995 | | 13511.86 | | Spools, concrete railroad ties | | | rr ties, spools, concrete | | | RR, Spools, Concrete | | RR, Spools, Concrete | | | mid-high | | 5 | 3 | | 11.73204 | | Other / Mixed | | Pyramids |
| 399 | 398 | | 1322.644 | | 2 | 23.31037 | | 97.94444 | | 28.832 | | D-21 | | 566.7995 | | 13511.86 | | Spools, concrete railroad ties | | | rr ties, spools, concrete | | | RR, Spools, Concrete | | RR, Spools, Concrete | | | mid-high | | 5 | 3 | | 8.187388 | | Other / Mixed | | Pyramids |
| 400 | 399 | | 7604.901 | | 3 | 23.26286 | | 143.8313 | | 23.28814 | | D-21 | | 566.7995 | | 13511.86 | | Spools, concrete railroad ties | | | rr ties, spools, concrete | | | RR, Spools, Concrete | | RR, Spools, Concrete | | | mid-high | | 5 | 3 | | 9.936548 | | Other / Mixed | | Pyramids |
| 401 | 400 | | 144069.4 | | 196 | 22.99584 | | 261.3708 | | 34.25408 | | D-23 | | 821.8087 | | 31780.67 | | Spools, broken concrete | | | rr ties, spools, concrete | | | RR, Spools, Concrete | | RR, Spools, Concrete | | | mid-high | | 5 | 3 | | 12.87805 | | Other / Mixed | | Pyramids |
| 402 | 401 | | 5177.654 | | 2 | 23.04404 | | 262.5605 | | 23.37291 | | D-23 | | 821.8087 | | 31780.67 | | Spools, broken concrete | | | rr ties, spools, concrete | | | RR, Spools, Concrete | | RR, Spools, Concrete | | | mid-high | | 5 | 3 | | 9.552107 | | Other / Mixed | | Pyramids |
| 403 | 402 | | 9523.53 | | 3 | 22.9577 | | 273.8912 | | 59.11017 | | D-23 | | 821.8087 | | 31780.67 | | Spools, broken concrete | | | rr ties, spools, concrete | | | RR, Spools, Concrete | | RR, Spools, Concrete | | | mid-high | | 5 | 3 | | 10.16152 | | Other / Mixed | | Pyramids |
| 404 | 403 | | 183721.4 | | 4 | 22.72901 | | 519.126 | | 27.7325 | | D-25 | | 724.2878 | | 27444.08 | | Spools, broken concrete | | | rr ties, spools, concrete | | | RR, Spools, Concrete | | RR, Spools, Concrete | | | mid-high | | 5 | 3 | | 13.12118 | | Other / Mixed | | Pyramids |
| 405 | 404 | | 304538 | | 14 | 22.7343 | | 391.2028 | | 19.30366 | | D-24 | | 838.8928 | | 44936.25 | | 18 ft spool,12-15 ft spools, concrete rubble, culverts | | | rr ties, spools, concrete, culverts | | | RR, Spools, Concrete, Culvert | | RR, Spools, Concrete, Culvert | | | mid-high | | 5 | 4 | | 13.62655 | | Other / Mixed | | Pyramids |
| 406 | 405 | | 776617.2 | | 1178 | 22.72128 | | 505.793 | | 14.46085 | | D-19 | | 814.6802 | | 26650.23 | | 19 ft spool,12-15 ft spools, concrete rubble, culverts | | | rr ties, spools, concrete, culverts | | | RR, Spools, Concrete, Culvert | | RR, Spools, Concrete, Culvert | | | mid-high | | 5 | 4 | | 14.5627 | | Other / Mixed | | RR Ties |
| 407 | 406 | | 40392.35 | | 12 | 22.83759 | | 304.1569 | | 18.3711 | | D-4 | | 843.0299 | | 33942.2 | | Large Spool, ties, culverts | | | rr ties, spools, culverts | | | RR, Spools, Culvert | | RR, Spools, Culvert | | | mid-high | | 5 | 3 | | 11.6064 | | Other / Mixed | | RR Ties |
| 408 | 407 | | 6539.556 | | 3 | 22.81195 | | 271.9767 | | 39.90491 | | D-5 | | 945.796 | | 41554.73 | | Large Spool, ties, culverts | | | rr ties, spools, culverts | | | RR, Spools, Culvert | | RR, Spools, Culvert | | | mid-high | | 5 | 3 | | 9.785625 | | Other / Mixed | | RR Ties |
| 409 | -1 | | 194.0734 | | 1 | 21.63522 | | 797.9763 | | 51.70838 | | Concrete10 ton12 | | 68.64516 | | 305.03 | | 10 ton concrete | | | concrete | | | 10 ton concrete | | 10T Concrete | | | low | | 8 | 1 | | 6.268236 | | Concrete | | RR Ties |
| 410 | -1 | | 1335.641 | | 1 | 22.55878 | | 661.307 | | 15.27932 | | Concrete10 ton3 | | 51.48548 | | 188.1299 | | 10 ton concrete | | | concrete | | | 10 ton concrete | | 10T Concrete | | | low | | 8 | 1 | | 8.197167 | | Concrete | | RR Ties |
| 411 | -1 | | 4993.594 | | 1 | 21.56815 | | 846.5559 | | 17.22297 | | Concrete10 ton13 | | 99.51987 | | 478.2649 | | 10 ton concrete | | | concrete | | | 10 ton concrete | | 10T Concrete | | | low | | 8 | 1 | | 9.515911 | | Concrete | | Concrete |
| 412 | -1 | | 1658.355 | | 1 | 21.42475 | | 836.6996 | | 3.839867 | | Concrete10 ton13 | | 99.51987 | | 478.2649 | | 10 ton concrete | | | concrete | | | 10 ton concrete | | 10T Concrete | | | low | | 8 | 1 | | 8.413581 | | Concrete | | Concrete |
| 413 | -1 | | 1117.577 | | 1 | 22.62821 | | 745.7971 | | 7.489898 | | Concrete10 ton4 | | 49.14813 | | 170.839 | | 10 ton concrete | | | concrete | | | 10 ton concrete | | 10T Concrete | | | low | | 8 | 1 | | 8.018918 | | Concrete | | Concrete |
| 414 | -1 | | 1365.591 | | 1 | 22.12216 | | 833.2196 | | 10.89918 | | Concrete10 ton6 | | 44.18505 | | 117.3124 | | 10 ton concrete | | | concrete | | | 10 ton concrete | | 10T Concrete | | | low | | 8 | 1 | | 8.219343 | | Concrete | | Concrete |
| 415 | -1 | | 3375.83 | | 1 | 21.87133 | | 839.912 | | 14.46167 | | Concrete10 ton8 | | 85.45058 | | 470.6305 | | 10 ton concrete | | | concrete | | | 10 ton concrete | | 10T Concrete | | | low | | 8 | 1 | | 9.124396 | | Concrete | | Concrete |
| 416 | -1 | | 1985.057 | | 1 | 21.77775 | | 855.1317 | | 18.72949 | | Concrete10 ton9 | | 65.98344 | | 160.3781 | | 10 ton concrete | | | concrete | | | 10 ton concrete | | 10T Concrete | | | low | | 8 | 1 | | 8.593403 | | Concrete | | Concrete |
| 417 | -1 | | 1181.788 | | 1 | 18.99157 | | 215.6505 | | 34.37837 | | Patch 1 | | 636.7052 | | 22425.64 | | low profile | | | low profile | | | 16LP | | 16LP | | | low | | 8 | 1 | | 8.074783 | | Low Profile | | Concrete |
| 418 | -1 | | 27.30022 | | 1 | 19.92361 | | 689.9417 | | 36.58094 | | Patch 34 | | 332.9863 | | 5882.656 | | low profile | | | low profile | | | 16LP | | 16LP | | | low | | 8 | 1 | | 4.306895 | | Low Profile | | Concrete |
| 419 | -1 | | 93.31528 | | 5 | 19.78859 | | 287.2136 | | 106.4294 | | Patch 9 | | 454.9512 | | 13059.44 | | low profile | | | low profile | | | 16LP | | 16LP | | | low | | 8 | 1 | | 5.535984 | | Low Profile | | Concrete |
| 420 | -1 | | 95.85001 | | 5 | 19.28471 | | 171.607 | | 66.52647 | | Patch 1 | | 636.7052 | | 22425.64 | | low profile | | | low profile | | | 16LP | | 16LP | | | low | | 8 | 1 | | 5.562785 | | Low Profile | | Concrete |
| 421 | -1 | | 99.27403 | | 5 | 19.88154 | | 270.9311 | | 120.0895 | | Patch 9 | | 454.9512 | | 13059.44 | | low profile | | | low profile | | | 16LP | | 16LP | | | low | | 8 | 1 | | 5.597884 | | Low Profile | | Concrete |
| 422 | -1 | | 114.8205 | | 6 | 19.8895 | | 454.9823 | | 71.8622 | | Patch 9 | | 454.9512 | | 13059.44 | | low profile | | | low profile | | | 16LP | | 16LP | | | low | | 8 | 1 | | 5.74337 | | Low Profile | | Cinderblocks |
| 423 | -1 | | 122.3881 | | 6 | 19.28967 | | 68.13876 | | 125.0759 | | Patch 1 | | 636.7052 | | 22425.64 | | low profile | | | low profile | | | 16LP | | 16LP | | | low | | 8 | 1 | | 5.807197 | | Low Profile | | Cinderblocks |
| 424 | -1 | | 197.7667 | | 10 | 19.86106 | | 649.5484 | | 94.92753 | | Patch 34 | | 332.9863 | | 5882.656 | | low profile | | | low profile | | | 16LP | | 16LP | | | low | | 8 | 1 | | 6.287088 | | Low Profile | | Cinderblocks |
| 425 | -1 | | 199.1345 | | 10 | 19.80183 | | 280.2926 | | 111.0721 | | Patch 9 | | 454.9512 | | 13059.44 | | low profile | | | low profile | | | 16LP | | 16LP | | | low | | 8 | 1 | | 6.29398 | | Low Profile | | Cinderblocks |
| 426 | -1 | | 683.6568 | | 34 | 20.05832 | | 452.0848 | | 42.40381 | | Patch 44 | | 492.4562 | | 16049.46 | | low profile | | | low profile | | | 16LP | | 16LP | | | low | | 8 | 1 | | 7.527456 | | Low Profile | | Cinderblocks |
| 427 | -1 | | 2025.737 | | 101 | 19.85824 | | 252.446 | | 141.8032 | | Patch 9 | | 454.9512 | | 13059.44 | | low profile | | | low profile | | | 16LP | | 16LP | | | low | | 8 | 1 | | 8.613689 | | Low Profile | | Cinderblocks |
| 428 | -1 | | 1586.696 | | 1 | 19.1809 | | 194.9501 | | 43.98648 | | Patch 1 | | 636.7052 | | 22425.64 | | low profile | | | low profile | | | 16LP | | 16LP | | | low | | 8 | 1 | | 8.369409 | | Low Profile | | Cinderblocks |
| 429 | -1 | | 1071.578 | | 1 | 19.1404 | | 184.3942 | | 16.31613 | | Patch 1 | | 636.7052 | | 22425.64 | | low profile | | | low profile | | | 16LP | | 16LP | | | low | | 8 | 1 | | 7.976888 | | Low Profile | | Cinderblocks |
| 430 | -1 | | 485.2197 | | 24 | 19.23973 | | 393.2282 | | 16.8061 | | Patch 22 | | 514.5348 | | 13292.24 | | low profile | | | low profile | | | 16LP | | 16LP | | | low | | 8 | 1 | | 7.184602 | | Low Profile | | Cinderblocks |
| 431 | -1 | | 12782.93 | | 1 | 18.83574 | | 101.3036 | | 164.0245 | | Patch 21 | | 721.9896 | | 30699.53 | | mixed; pyramids, lpm | | | pyramids, low profile | | | 16MX | | 16MX | | | mid | | 8 | 2 | | 10.45587 | | Pyramids | | Cinderblocks |
| 432 | -1 | | 545.0925 | | 1 | 20.44991 | | 814.3281 | | 84.99767 | | Patch 35 | | 687.7667 | | 30005.6 | | mixed; pyramids, lpm | | | pyramids, low profile | | | 16MX | | 16MX | | | mid | | 8 | 2 | | 7.300955 | | Pyramids | | Cinderblocks |
| 433 | -1 | | 267.0073 | | 1 | 18.70608 | | 205.8014 | | 76.30132 | | Patch 21 | | 721.9896 | | 30699.53 | | mixed; pyramids, lpm | | | pyramids, low profile | | | 16MX | | 16MX | | | mid | | 8 | 2 | | 6.587276 | | Pyramids | | Cinderblocks |
| 434 | -1 | | 2369.248 | | 1 | 19.16589 | | 100.0765 | | 108.7111 | | Patch 21 | | 721.9896 | | 30699.53 | | mixed; pyramids, lpm | | | pyramids, low profile | | | 16MX | | 16MX | | | mid | | 8 | 2 | | 8.770328 | | Pyramids | | Cinderblocks |
| 435 | -1 | | 2350.286 | | 1 | 19.99942 | | 296.769 | | 87.48091 | | Patch 48 | | 637.7184 | | 23935.57 | | mixed; pyramids, lpm | | | pyramids, low profile | | | 16MX | | 16MX | | | mid | | 8 | 2 | | 8.762292 | | Pyramids | | Cinderblocks |
| 436 | -1 | | 28.26721 | | 1 | 20.47902 | | 227.0061 | | 68.64721 | | Patch 5 | | 581.2411 | | 24281.88 | | mixed; pyramids, lpm | | | pyramids, low profile | | | 16MX | | 16MX | | | mid | | 8 | 2 | | 4.341702 | | Pyramids | | Cinderblocks |
| 437 | -1 | | 34.05175 | | 2 | 20.10186 | | 223.1692 | | 67.47412 | | Patch 5 | | 581.2411 | | 24281.88 | | mixed; pyramids, lpm | | | pyramids, low profile | | | 16MX | | 16MX | | | mid | | 8 | 2 | | 4.527881 | | Pyramids | | Cinderblocks |
| 438 | -1 | | 1325.476 | | 66 | 18.77761 | | 215.1508 | | 111.1193 | | Patch 21 | | 721.9896 | | 30699.53 | | mixed; pyramids, lpm | | | pyramids, low profile | | | 16MX | | 16MX | | | mid | | 8 | 2 | | 8.189527 | | Pyramids | | Cinderblocks |
| 439 | -1 | | 4696.763 | | 1 | 19.00938 | | 177.8862 | | 51.53074 | | Patch 21 | | 721.9896 | | 30699.53 | | mixed; pyramids, lpm | | | pyramids, low profile | | | 16MX | | 16MX | | | mid | | 8 | 2 | | 9.454629 | | Pyramids | | Cinderblocks |
| 440 | -1 | | 3356.234 | | 1 | 19.16716 | | 150.1826 | | 98.1052 | | Patch 21 | | 721.9896 | | 30699.53 | | mixed; pyramids, lpm | | | pyramids, low profile | | | 16MX | | 16MX | | | mid | | 8 | 2 | | 9.118575 | | Pyramids | | Cinderblocks |
| 441 | -1 | | 8429.938 | | 1 | 20.89391 | | 834.4419 | | 29.80663 | | Patch 35 | | 687.7667 | | 30005.6 | | mixed; pyramids, lpm | | | pyramids, low profile | | | 16MX | | 16MX | | | mid | | 8 | 2 | | 10.03954 | | Pyramids | | Cinderblocks |
| 442 | -1 | | 60.59057 | | 3 | 20.81514 | | 815.0225 | | 30.78285 | | Patch 35 | | 687.7667 | | 30005.6 | | mixed; pyramids, lpm | | | pyramids, low profile | | | 16MX | | 16MX | | | mid | | 8 | 2 | | 5.104139 | | Pyramids | | Cinderblocks |
| 443 | -1 | | 253.125 | | 13 | 20.53976 | | 814.8723 | | 51.16519 | | Patch 35 | | 687.7667 | | 30005.6 | | mixed; pyramids, lpm | | | pyramids, low profile | | | 16MX | | 16MX | | | mid | | 8 | 2 | | 6.533883 | | Pyramids | | Cinderblocks |
| 444 | -1 | | 50993.37 | | 2550 | 20.76401 | | 815.7297 | | 11.47193 | | Patch 35 | | 687.7667 | | 30005.6 | | mixed; pyramids, lpm | | | pyramids, low profile | | | 16MX | | 16MX | | | mid | | 8 | 2 | | 11.83945 | | Pyramids | | Cinderblocks |
| 445 | -1 | | 802.8966 | | 1 | 19.861 | | 314.9035 | | 47.76054 | | Patch 48 | | 637.7184 | | 23935.57 | | mixed; pyramids, lpm | | | pyramids, low profile | | | 16MX | | 16MX | | | mid | | 8 | 2 | | 7.688226 | | Pyramids | | Cinderblocks |
| 446 | -1 | | 535.3859 | | 1 | 20.57064 | | 287.5805 | | 10.62322 | | Patch 48 | | 637.7184 | | 23935.57 | | mixed; pyramids, lpm | | | pyramids, low profile | | | 16MX | | 16MX | | | mid | | 8 | 2 | | 7.282988 | | Pyramids | | Cinderblocks |
| 447 | -1 | | 47.66885 | | 2 | 20.32873 | | 273.2137 | | 9.69684 | | Patch 48 | | 637.7184 | | 23935.57 | | mixed; pyramids, lpm | | | pyramids, low profile | | | 16MX | | 16MX | | | mid | | 8 | 2 | | 4.864278 | | Pyramids | | Cinderblocks |
| 448 | -1 | | 1188.952 | | 1 | 20.8627 | | 202.478 | | 8.979582 | | Patch 5 | | 581.2411 | | 24281.88 | | mixed; pyramids, lpm | | | pyramids, low profile | | | 16MX | | 16MX | | | mid | | 8 | 2 | | 8.080827 | | Pyramids | | Cinderblocks |
| 449 | -1 | | 4743.201 | | 1 | 20.28142 | | 805.1825 | | 69.82584 | | Patch 20 | | 804.1207 | | 27295.57 | | pyramids | | | pyramids | | | 16PY | | 16PY | | | mid | | 8 | 1 | | 9.464467 | | Pyramids | | Cinderblocks |
| 450 | -1 | | 1460.623 | | 1 | 20.38381 | | 832.5281 | | 101.2122 | | Patch 20 | | 804.1207 | | 27295.57 | | pyramids | | | pyramids | | | 16PY | | 16PY | | | mid | | 8 | 1 | | 8.286619 | | Pyramids | | Cinderblocks |
| 451 | -1 | | 68.41658 | | 1 | 19.19213 | | 390.887 | | 67.19131 | | Patch 27 | | 713.1051 | | 24736.61 | | pyramids | | | pyramids | | | 16PY | | 16PY | | | mid | | 8 | 1 | | 5.225615 | | Pyramids | | Cinderblocks |
| 452 | -1 | | 344.1024 | | 1 | 19.49567 | | 405.0934 | | 33.47846 | | Patch 27 | | 713.1051 | | 24736.61 | | pyramids | | | pyramids | | | 16PY | | 16PY | | | mid | | 8 | 1 | | 6.840939 | | Pyramids | | Cinderblocks |
| 453 | -1 | | 22.41657 | | 1 | 19.25272 | | 314.907 | | 78.14535 | | Patch 8 | | 617.3887 | | 25733.57 | | pyramids | | | pyramids | | | 16PY | | 16PY | | | mid | | 8 | 1 | | 4.1098 | | Pyramids | | Cinderblocks |
| 454 | -1 | | 39.23937 | | 2 | 20.03635 | | 687.175 | | 93.36705 | | Patch 20 | | 804.1207 | | 27295.57 | | pyramids | | | pyramids | | | 16PY | | 16PY | | | mid | | 8 | 1 | | 4.66968 | | Pyramids | | Cinderblocks |
| 455 | -1 | | 48.66507 | | 2 | 20.18186 | | 706.7661 | | 68.57991 | | Patch 20 | | 804.1207 | | 27295.57 | | pyramids | | | pyramids | | | 16PY | | 16PY | | | mid | | 8 | 1 | | 4.884961 | | Pyramids | | Cinderblocks |
| 456 | -1 | | 110.9859 | | 6 | 20.30856 | | 815.5747 | | 75.68116 | | Patch 20 | | 804.1207 | | 27295.57 | | pyramids | | | pyramids | | | 16PY | | 16PY | | | mid | | 8 | 1 | | 5.709403 | | Pyramids | | Cinderblocks |
| 457 | -1 | | 215.3476 | | 11 | 19.77135 | | 444.106 | | 112.1083 | | Patch 8 | | 617.3887 | | 25733.57 | | pyramids | | | pyramids | | | 16PY | | 16PY | | | mid | | 8 | 1 | | 6.372254 | | Pyramids | | Cinderblocks |
| 458 | -1 | | 917.6611 | | 1 | 20.67227 | | 743.0278 | | 15.25101 | | Patch 20 | | 804.1207 | | 27295.57 | | pyramids | | | pyramids | | | 16PY | | 16PY | | | mid | | 8 | 1 | | 7.821828 | | Pyramids | | Cinderblocks |
| 459 | -1 | | 4322.714 | | 1 | 20.04511 | | 219.9153 | | 19.37675 | | Patch 41 | | 628.1935 | | 23819.9 | | pyramids | | | pyramids | | | 16PY | | 16PY | | | mid | | 8 | 1 | | 9.371639 | | Pyramids | | Cinderblocks |
| 460 | -1 | | 61.32698 | | 3 | 19.30456 | | 241.4584 | | 44.43104 | | Patch 41 | | 628.1935 | | 23819.9 | | pyramids | | | pyramids | | | 16PY | | 16PY | | | mid | | 8 | 1 | | 5.11622 | | Pyramids | | Cinderblocks |
| 461 | -1 | | 835.7519 | | 1 | 22.10071 | | 304.1162 | | 35.10221 | | 1-6 Block16 | | 72.2536 | | 200.0671 | | 1 6 in palet cinderblocks | | | cinderblocks | | | 1 pallet cinderblocks | | 1CB | | | low | | 8 | 1 | | 7.728332 | | Cinderblocks | | Cinderblocks |
| 462 | -1 | | 3112.788 | | 1 | 22.47386 | | 522.1066 | | 29.42901 | | 1-6 Block19 | | 191.3992 | | 1271.376 | | 1 6 in palet cinderblocks | | | cinderblocks | | | 1 pallet cinderblocks | | 1CB | | | low | | 8 | 1 | | 9.043274 | | Cinderblocks | | Cinderblocks |
| 463 | -1 | | 911.4124 | | 1 | 21.72273 | | 234.1245 | | 5.100569 | | 1-8 Block37 | | 40.67442 | | 102.3391 | | 1 8 in palet cinderblocks | | | cinderblocks | | | 1 pallet cinderblocks | | 1CB | | | low | | 8 | 1 | | 7.814995 | | Cinderblocks | | Cinderblocks |
| 464 | -1 | | 2047.018 | | 1 | 21.69216 | | 209.6031 | | 42.084 | | 1-8 Block37 | | 40.67442 | | 102.3391 | | 1 8 in palet cinderblocks | | | cinderblocks | | | 1 pallet cinderblocks | | 1CB | | | low | | 8 | 1 | | 8.624139 | | Cinderblocks | | Cinderblocks |
| 465 | -1 | | 1306.964 | | 1 | 19.20058 | | 206.4393 | | 14.25305 | | Patch 31 | | 69.6398 | | 302.1493 | | mixed; pyramids, lpm | | | pyramids, low profile | | | 1MX | | 1MX | | | mid | | 8 | 2 | | 8.175462 | | Pyramids | | Cinderblocks |
| 466 | -1 | | 3316.34 | | 1 | 19.07345 | | 63.27058 | | 139.7653 | | Patch 6 | | 163.488 | | 1152.274 | | mixed; pyramids, lpm | | | pyramids, low profile | | | 1MX | | 1MX | | | mid | | 8 | 2 | | 9.106617 | | Pyramids | | Cinderblocks |
| 467 | -1 | | 1120.214 | | 1 | 19.33563 | | 156.8746 | | 51.54537 | | Patch 31 | | 69.6398 | | 302.1493 | | mixed; pyramids, lpm | | | pyramids, low profile | | | 1MX | | 1MX | | | mid | | 8 | 2 | | 8.021275 | | Pyramids | | Cinderblocks |
| 468 | -1 | | 3911.386 | | 1 | 19.7475 | | 634.1698 | | 84.13816 | | Patch 39 | | 72.89791 | | 311.5434 | | mixed; pyramids, lpm | | | pyramids, low profile | | | 1MX | | 1MX | | | mid | | 8 | 2 | | 9.271647 | | Pyramids | | Cinderblocks |
| 469 | -1 | | 2881.179 | | 1 | 20.71692 | | 705.5488 | | 103.3278 | | Patch 51 | | 107.5291 | | 585.2189 | | mixed; pyramids, lpm | | | pyramids, low profile | | | 1MX | | 1MX | | | mid | | 8 | 2 | | 8.965955 | | Pyramids | | Cinderblocks |
| 470 | -1 | | 30.80977 | | 2 | 19.95701 | | 649.7505 | | 91.38791 | | Patch 19 | | 42.212 | | 84.86567 | | mixed; pyramids, lpm | | | pyramids, low profile | | | 1MX | | 1MX | | | mid | | 8 | 2 | | 4.427832 | | Pyramids | | Cinderblocks |
| 471 | -1 | | 39.9339 | | 2 | 19.99945 | | 642.8092 | | 34.80913 | | Patch 39 | | 72.89791 | | 311.5434 | | mixed; pyramids, lpm | | | pyramids, low profile | | | 1MX | | 1MX | | | mid | | 8 | 2 | | 4.687226 | | Pyramids | | Cinderblocks |
| 472 | -1 | | 46.73662 | | 2 | 18.96405 | | 173.3524 | | 47.66076 | | Patch 31 | | 69.6398 | | 302.1493 | | mixed; pyramids, lpm | | | pyramids, low profile | | | 1MX | | 1MX | | | mid | | 8 | 2 | | 4.844528 | | Pyramids | | Cinderblocks |
| 473 | -1 | | 67.82277 | | 3 | 19.98692 | | 645.5899 | | 111.4897 | | Patch 19 | | 42.212 | | 84.86567 | | mixed; pyramids, lpm | | | pyramids, low profile | | | 1MX | | 1MX | | | mid | | 8 | 2 | | 5.216898 | | Pyramids | | Cinderblocks |
| 474 | -1 | | 9922.108 | | 496 | 19.15654 | | 269.9988 | | 71.63205 | | Patch 31 | | 69.6398 | | 302.1493 | | mixed; pyramids, lpm | | | pyramids, low profile | | | 1MX | | 1MX | | | mid | | 8 | 2 | | 10.20252 | | Pyramids | | Cinderblocks |
| 475 | -1 | | 26981.71 | | 1349 | 19.19378 | | 77.329 | | 118.1285 | | Patch 6 | | 163.488 | | 1152.274 | | mixed; pyramids, lpm | | | pyramids, low profile | | | 1MX | | 1MX | | | mid | | 8 | 2 | | 11.20291 | | Pyramids | | Cinderblocks |
| 476 | -1 | | 37800.74 | | 1890 | 19.15 | | 139.9181 | | 103.6546 | | Patch 31 | | 69.6398 | | 302.1493 | | mixed; pyramids, lpm | | | pyramids, low profile | | | 1MX | | 1MX | | | mid | | 8 | 2 | | 11.54008 | | Pyramids | | Cinderblocks |
| 477 | -1 | | 1914.145 | | 1 | 19.23574 | | 84.24436 | | 131.0207 | | Patch 46 | | 48.74995 | | 136.2459 | | single pyramid | | | pyramid | | | 1PY | | 1PY | | | mid | | 8 | 1 | | 8.557026 | | Pyramids | | Cinderblocks |
| 478 | -1 | | 773.598 | | 1 | 19.79813 | | 608.0877 | | 64.04936 | | Patch 14 | | 44.59184 | | 105.6696 | | single pyramid | | | pyramid | | | 1PY | | 1PY | | | mid | | 8 | 1 | | 7.651052 | | Pyramids | | Cinderblocks |
| 479 | -1 | | 241.7253 | | 1 | 20.42504 | | 629.6973 | | 48.73367 | | Patch 40 | | 54.91298 | | 211.3516 | | single pyramid | | | pyramids | | | 1PY | | 1PY | | | mid | | 8 | 1 | | 6.487802 | | Pyramids | | Cinderblocks |
| 480 | -1 | | 784.8236 | | 1 | 20.55847 | | 641.7105 | | 14.1973 | | Patch 40 | | 54.91298 | | 211.3516 | | single pyramid | | | pyramids | | | 1PY | | 1PY | | | mid | | 8 | 1 | | 7.665459 | | Pyramids | | Cinderblocks |
| 481 | -1 | | 21.24945 | | 1 | 19.00781 | | 164.5916 | | 35.35411 | | Patch 46 | | 48.74995 | | 136.2459 | | single pyramid | | | pyramid | | | 1PY | | 1PY | | | mid | | 8 | 1 | | 4.056331 | | Pyramids | | Cinderblocks |
| 482 | -1 | | 31.20082 | | 2 | 20.07341 | | 498.0477 | | 92.17307 | | Patch 14 | | 44.59184 | | 105.6696 | | single pyramid | | | pyramid | | | 1PY | | 1PY | | | mid | | 8 | 1 | | 4.440445 | | Pyramids | | Cinderblocks |
| 483 | -1 | | 39.75531 | | 2 | 19.9523 | | 600.2209 | | 68.08018 | | Patch 14 | | 44.59184 | | 105.6696 | | single pyramid | | | pyramid | | | 1PY | | 1PY | | | mid | | 8 | 1 | | 4.682743 | | Pyramids | | Cinderblocks |
| 484 | -1 | | 60.82621 | | 3 | 19.56211 | | 451.4929 | | 73.60229 | | Patch 42 | | 44.31758 | | 120.3585 | | single pyramid | | | pyramids | | | 1PY | | 1PY | | | mid | | 8 | 1 | | 5.108021 | | Pyramids | | Cinderblocks |
| 485 | -1 | | 69.3813 | | 3 | 20.29314 | | 637.0214 | | 29.24178 | | Patch 40 | | 54.91298 | | 211.3516 | | single pyramid | | | pyramids | | | 1PY | | 1PY | | | mid | | 8 | 1 | | 5.239617 | | Pyramids | | Cinderblocks |
| 486 | -1 | | 102.7869 | | 5 | 19.14659 | | 275.9667 | | 107.3548 | | Patch 2 | | 40.38134 | | 77.17003 | | single pyramid | | | pyramid | | | 1PY | | 1PY | | | mid | | 8 | 1 | | 5.632658 | | Pyramids | | Cinderblocks |
| 487 | -1 | | 173.1656 | | 9 | 20.03206 | | 480.1245 | | 95.35986 | | Patch 14 | | 44.59184 | | 105.6696 | | single pyramid | | | pyramid | | | 1PY | | 1PY | | | mid | | 8 | 1 | | 6.154248 | | Pyramids | | RR Ties |
| 488 | -1 | | 246.6854 | | 12 | 20.04661 | | 614.8285 | | 89.99475 | | Patch 14 | | 44.59184 | | 105.6696 | | single pyramid | | | pyramid | | | 1PY | | 1PY | | | mid | | 8 | 1 | | 6.508114 | | Pyramids | | RR Ties |
| 489 | -1 | | 281.1635 | | 14 | 19.0363 | | 253.1044 | | 73.82682 | | Patch 2 | | 40.38134 | | 77.17003 | | single pyramid | | | pyramid | | | 1PY | | 1PY | | | mid | | 8 | 1 | | 6.638936 | | Pyramids | | Low Profile |
| 490 | -1 | | 1046.683 | | 52 | 19.14711 | | 252.301 | | 93.513 | | Patch 46 | | 48.74995 | | 136.2459 | | single pyramid | | | pyramid | | | 1PY | | 1PY | | | mid | | 8 | 1 | | 7.953381 | | Pyramids | | Low Profile |
| 491 | -1 | | 1299.768 | | 65 | 19.07109 | | 100.0008 | | 118.194 | | Patch 2 | | 40.38134 | | 77.17003 | | single pyramid | | | pyramid | | | 1PY | | 1PY | | | mid | | 8 | 1 | | 8.169941 | | Pyramids | | Low Profile |
| 492 | -1 | | 5656.83 | | 283 | 19.05857 | | 99.45455 | | 104.417 | | Patch 2 | | 40.38134 | | 77.17003 | | single pyramid | | | pyramid | | | 1PY | | 1PY | | | mid | | 8 | 1 | | 9.640619 | | Pyramids | | Low Profile |
| 493 | -1 | | 35.38238 | | 2 | 19.90534 | | 545.4311 | | 1.393513 | | Patch 14 | | 44.59184 | | 105.6696 | | single pyramid | | | pyramid | | | 1PY | | 1PY | | | mid | | 8 | 1 | | 4.566214 | | Pyramids | | Low Profile |
| 494 | -1 | | 86.25275 | | 4 | 20.00007 | | 593.5906 | | 49.5611 | | Patch 14 | | 44.59184 | | 105.6696 | | single pyramid | | | pyramid | | | 1PY | | 1PY | | | mid | | 8 | 1 | | 5.457282 | | Pyramids | | Low Profile |
| 495 | -1 | | 300.0401 | | 1 | 19.08082 | | 256.813 | | 70.03961 | | Patch 46 | | 48.74995 | | 136.2459 | | single pyramid | | | pyramid | | | 1PY | | 1PY | | | mid | | 8 | 1 | | 6.703916 | | Pyramids | | Low Profile |
| 496 | -1 | | 13183.77 | | 659 | 22.43608 | | 703.7244 | | 5.082406 | | 200 | | 443.0979 | | 12805.92 | | 200 rr tie | | | rr ties | | | 200 rr tie | | 200T RR | | | mid-high | | 8 | 1 | | 10.48674 | | RR Ties | | Low Profile |
| 497 | -1 | | 444.9689 | | 1 | 22.61756 | | 223.3262 | | 71.70988 | | 250-20 | | 547.0743 | | 17094.68 | | 250 ton pile rr ties | | | rr ties | | | 250T RR | | 250T RR | | | mid-high | | 6 | 1 | | 7.098004 | | RR Ties | | Low Profile |
| 498 | -1 | | 181.5644 | | 1 | 22.9784 | | 265.0885 | | 50.64982 | | 250-17 | | 397.0561 | | 10737.79 | | 250 ton pile rr ties | | | rr ties | | | 250T RR | | 250T RR | | | mid-high | | 6 | 1 | | 6.20161 | | RR Ties | | Pyramids |
| 499 | -1 | | 838.1455 | | 1 | 22.98399 | | 239.6683 | | 25.95145 | | 250-18 | | 502.4975 | | 14896.36 | | 250 ton pile rr ties | | | rr ties | | | 250T RR | | 250T RR | | | mid-high | | 6 | 1 | | 7.731192 | | RR Ties | | Pyramids |
| 500 | -1 | | 9273.106 | | 1 | 22.98375 | | 256.5687 | | 37.99927 | | 250-17 | | 397.0561 | | 10737.79 | | 250 ton pile rr ties | | | rr ties | | | 250T RR | | 250T RR | | | mid-high | | 6 | 1 | | 10.13487 | | RR Ties | | Pyramids |
| 501 | -1 | | 2441.9 | | 1 | 23.12214 | | 180.1262 | | 19.56094 | | 250-22 | | 439.6424 | | 9330.136 | | 250 ton pile rr ties | | | rr ties | | | 250T RR | | 250T RR | | | mid-high | | 6 | 1 | | 8.800532 | | RR Ties | | Pyramids |
| 502 | -1 | | 247.2276 | | 1 | 22.94484 | | 111.6881 | | 27.13483 | | 250-3 | | 533.8506 | | 12928.94 | | 250 ton pile rr ties | | | rr ties | | | 250T RR | | 250T RR | | | mid-high | | 6 | 1 | | 6.510309 | | RR Ties | | Pyramids |
| 503 | -1 | | 1153.079 | | 1 | 23.20501 | | 108.8432 | | 25.89145 | | 250-1 | | 403.1612 | | 12376.16 | | 250 ton pile rr ties | | | rr ties | | | 250T RR | | 250T RR | | | mid-high | | 6 | 1 | | 8.050191 | | RR Ties | | Pyramids |
| 504 | -1 | | 1480.307 | | 1 | 22.50349 | | 145.2009 | | 35.75534 | | 250-5 | | 449.1978 | | 13227.3 | | 250 ton pile rr ties | | | rr ties | | | 250T RR | | 250T RR | | | mid-high | | 6 | 1 | | 8.300005 | | RR Ties | | Pyramids |
| 505 | -1 | | 2807.416 | | 1 | 22.56769 | | 105.6798 | | 24.12807 | | 250-6 | | 420.3532 | | 11466.76 | | 250 ton pile rr ties | | | rr ties | | | 250T RR | | 250T RR | | | mid-high | | 6 | 1 | | 8.94002 | | RR Ties | | Pyramids |
| 506 | -1 | | 24480.29 | | 1 | 22.50906 | | 102.0286 | | 32.98854 | | 250-6 | | 420.3532 | | 11466.76 | | 250 ton pile rr ties | | | rr ties | | | 250T RR | | 250T RR | | | mid-high | | 6 | 1 | | 11.10562 | | RR Ties | | Pyramids |
| 507 | -1 | | 465.4652 | | 1 | 22.58874 | | 124.8241 | | 38.45744 | | 250-5 | | 449.1978 | | 13227.3 | | 250 ton pile rr ties | | | rr ties | | | 250T RR | | 250T RR | | | mid-high | | 6 | 1 | | 7.143037 | | RR Ties | | Pyramids |
| 508 | -1 | | 3902.027 | | 1 | 22.66361 | | 138.6185 | | 23.21618 | | 250-5 | | 449.1978 | | 13227.3 | | 250 ton pile rr ties | | | rr ties | | | 250T RR | | 250T RR | | | mid-high | | 6 | 1 | | 9.269252 | | RR Ties | | Pyramids |
| 509 | -1 | | 1460.947 | | 1 | 22.57326 | | 128.7042 | | 18.73815 | | 250-5 | | 449.1978 | | 13227.3 | | 250 ton pile rr ties | | | rr ties | | | 250T RR | | 250T RR | | | mid-high | | 6 | 1 | | 8.28684 | | RR Ties | | Pyramids |
| 510 | -1 | | 778.1299 | | 1 | 22.97541 | | 245.4152 | | 23.12014 | | 250-18 | | 502.4975 | | 14896.36 | | 250 ton pile rr ties | | | rr ties | | | 250T RR | | 250T RR | | | mid-high | | 6 | 1 | | 7.656893 | | RR Ties | | Pyramids |
| 511 | -1 | | 7928.627 | | 1 | 22.77667 | | 202.7275 | | 33.59637 | | 250-20 | | 547.0743 | | 17094.68 | | 250 ton pile rr ties | | | rr ties | | | 250T RR | | 250T RR | | | mid-high | | 6 | 1 | | 9.978235 | | RR Ties | | Pyramids |
| 512 | -1 | | 2166.806 | | 1 | 23.2 | | 147.8686 | | 84.05834 | | 250-21 | | 495.6666 | | 13837.4 | | 250 ton pile rr ties | | | rr ties | | | 250T RR | | 250T RR | | | mid-high | | 6 | 1 | | 8.681009 | | RR Ties | | Pyramids |
| 513 | -1 | | 1273.187 | | 1 | 21.91693 | | 123.1293 | | 33.45727 | | 250-9 | | 498.9416 | | 14213.48 | | 250 ton pile rr ties | | | rr ties | | | 250T RR | | 250T RR | | | mid-high | | 6 | 1 | | 8.149278 | | RR Ties | | Pyramids |
| 514 | -1 | | 1198.372 | | 1 | 22.18955 | | 115.8716 | | 19.98978 | | 250-7 | | 493.0979 | | 12838.84 | | 250 ton pile rr ties | | | rr ties | | | 250T RR | | 250T RR | | | mid-high | | 6 | 1 | | 8.088719 | | RR Ties | | Pyramids |
| 515 | -1 | | 731.3095 | | 1 | 22.8893 | | 145.6176 | | 32.78727 | | 250-2 | | 524.274 | | 14719.58 | | 250 ton pile rr ties | | | rr ties | | | 250T RR | | 250T RR | | | mid-high | | 6 | 1 | | 7.594837 | | RR Ties | | Pyramids |
| 516 | -1 | | 1634.287 | | 1 | 23.00631 | | 144.9194 | | 41.03401 | | 250-1 | | 403.1612 | | 12376.16 | | 250 ton pile rr ties | | | rr ties | | | 250T RR | | 250T RR | | | mid-high | | 6 | 1 | | 8.398962 | | RR Ties | | Pyramids |
| 517 | -1 | | 4251.068 | | 1 | 22.60158 | | 212.6093 | | 72.95519 | | 250-20 | | 547.0743 | | 17094.68 | | 250 ton pile rr ties | | | rr ties | | | 250T RR | | 250T RR | | | mid-high | | 6 | 1 | | 9.354926 | | RR Ties | | Pyramids |
| 518 | -1 | | 352.8901 | | 1 | 21.75984 | | 122.8414 | | 10.04013 | | 250-13 | | 497.1636 | | 15316.15 | | 250 ton pile rr ties | | | rr ties | | | 250T RR | | 250T RR | | | mid-high | | 6 | 1 | | 6.866157 | | RR Ties | | Pyramids |
| 519 | -1 | | 1346.234 | | 1 | 23.09016 | | 240.1714 | | 4.131585 | | 250-19 | | 398.0899 | | 9756.074 | | 250 ton pile rr ties | | | rr ties | | | 250T RR | | 250T RR | | | mid-high | | 6 | 1 | | 8.205066 | | RR Ties | | Pyramids |
| 520 | -1 | | 995.1693 | | 1 | 23.36071 | | 177.6183 | | 9.202546 | | 250-21 | | 495.6666 | | 13837.4 | | 250 ton pile rr ties | | | rr ties | | | 250T RR | | 250T RR | | | mid-high | | 6 | 1 | | 7.902913 | | RR Ties | | Pyramids |
| 521 | -1 | | 229.8904 | | 1 | 21.75468 | | 925.2185 | | 24.97021 | | 25.2 | | 362.739 | | 8906.291 | | 25 rr tie | | | rr ties | | | 25 rr tie | | 25T RR | | | low-mid | | 8 | 1 | | 6.437603 | | RR Ties | | Pyramids |
| 522 | -1 | | 127.103 | | 1 | 22.42964 | | 828.1381 | | 6.525615 | | 25.1 | | 288.2691 | | 3637.474 | | 25 rr tie | | | rr ties | | | 25 rr tie | | 25T RR | | | low-mid | | 8 | 1 | | 5.844998 | | RR Ties | | Pyramids |
| 523 | -1 | | 3296.228 | | 165 | 22.05235 | | 946.5974 | | 17.17352 | | 25.2 | | 362.739 | | 8906.291 | | 25 rr tie | | | rr ties | | | 25 rr tie | | 25T RR | | | low-mid | | 8 | 1 | | 9.100534 | | RR Ties | | Pyramids |
| 524 | -1 | | 2664.906 | | 1 | 21.79252 | | 349.718 | | 45.22052 | | Concrete2 ton7 | | 180.12 | | 825.461 | | 2 ton concrete | | | concrete | | | 2 ton concrete | | 2T Concrete | | | low | | 8 | 1 | | 8.887924 | | Concrete | | Pyramids |
| 525 | -1 | | 47.20598 | | 2 | 20.8475 | | 811.9651 | | 54.30028 | | Concrete2 ton11 | | 58.0714 | | 211.5329 | | 2 ton concrete | | | concrete | | | 2 ton concrete | | 2T Concrete | | | low | | 8 | 1 | | 4.854521 | | Concrete | | Pyramids |
| 526 | -1 | | 2165.522 | | 1 | 20.66373 | | 477.2735 | | 16.34809 | | Concrete2 ton10 | | 67.74735 | | 154.19 | | 2 ton concrete | | | concrete | | | 2 ton concrete | | 2T Concrete | | | low | | 8 | 1 | | 8.680417 | | Concrete | | Pyramids |
| 527 | -1 | | 3964.3 | | 1 | 20.69682 | | 466.3907 | | 28.89583 | | Concrete2 ton10 | | 67.74735 | | 154.19 | | 2 ton concrete | | | concrete | | | 2 ton concrete | | 2T Concrete | | | low | | 8 | 1 | | 9.285085 | | Concrete | | Pyramids |
| 528 | -1 | | 23.9497 | | 1 | 20.88533 | | 786.5237 | | 9.982599 | | Concrete2 ton11 | | 58.0714 | | 211.5329 | | 2 ton concrete | | | concrete | | | 2 ton concrete | | 2T Concrete | | | low | | 8 | 1 | | 4.175956 | | Concrete | | Pyramids |
| 529 | -1 | | 1750.014 | | 1 | 22.28927 | | 447.097 | | 12.89565 | | Concrete2 ton6 | | 97.33692 | | 520.9346 | | 2 ton concrete | | | concrete | | | 2 ton concrete | | 2T Concrete | | | low | | 8 | 1 | | 8.467379 | | Concrete | | Pyramids |
| 530 | -1 | | 2000.963 | | 1 | 22.2999 | | 457.4123 | | 9.363694 | | Concrete2 ton6 | | 97.33692 | | 520.9346 | | 2 ton concrete | | | concrete | | | 2 ton concrete | | 2T Concrete | | | low | | 8 | 1 | | 8.601384 | | Concrete | | Pyramids |
| 531 | -1 | | 3194.055 | | 1 | 21.83604 | | 369.0995 | | 13.99558 | | Concrete2 ton7 | | 180.12 | | 825.461 | | 2 ton concrete | | | concrete | | | 2 ton concrete | | 2T Concrete | | | low | | 8 | 1 | | 9.069046 | | Concrete | | Pyramids |
| 532 | -1 | | 1324.913 | | 1 | 21.55875 | | 974.4589 | | 22.51373 | | 30-mixed Block3 | | 223.7235 | | 3484.575 | | 30 pallets mixed size cinderblocks | | | cinderblocks | | | 30 pallets cinderblocks | | 30CB | | | low-mid | | 8 | 1 | | 8.189102 | | Cinderblocks | | Pyramids |
| 533 | -1 | | 3727.932 | | 1 | 21.43313 | | 985.015 | | 16.17009 | | 30-mixed Block3 | | 223.7235 | | 3484.575 | | 30 pallets mixed size cinderblocks | | | cinderblocks | | | 30 pallets cinderblocks | | 30CB | | | low-mid | | 8 | 1 | | 9.223609 | | Cinderblocks | | Pyramids |
| 534 | -1 | | 1890.233 | | 1 | 21.8344 | | 902.4608 | | 27.73404 | | 30-mixed Block1 | | 298.2626 | | 6074.309 | | 30 pallets mixed sized cinderblocks | | | cinderblocks | | | 30 pallets cinderblocks | | 30CB | | | low-mid | | 8 | 1 | | 8.544455 | | Cinderblocks | | Pyramids |
| 535 | -1 | | 10680.1 | | 1 | 21.37684 | | 1003.83 | | 21.79767 | | 30-mixed Block3 | | 223.7235 | | 3484.575 | | 30 pallets mixed size cinderblocks | | | cinderblocks | | | 30 pallets cinderblocks | | 30CB | | | low-mid | | 8 | 1 | | 10.27614 | | Cinderblocks | | Pyramids |
| 536 | -1 | | 802.2704 | | 1 | 22.08456 | | 541.6642 | | 136.7003 | | 3-mixed Block17 | | 145.0921 | | 1384.376 | | 3 pallets mixed size cinderblocks | | | cinderblocks | | | 3 pallets cinderblocks | | 3CB | | | low | | 8 | 1 | | 7.687446 | | Cinderblocks | | Pyramids |
| 537 | -1 | | 1576.593 | | 1 | 22.14802 | | 577.3668 | | 90.88091 | | 3-mixed Block17 | | 145.0921 | | 1384.376 | | 3 pallets mixed size cinderblocks | | | cinderblocks | | | 3 pallets cinderblocks | | 3CB | | | low | | 8 | 1 | | 8.363021 | | Cinderblocks | | Pyramids |
| 538 | -1 | | 608.9512 | | 1 | 22.26402 | | 674.8246 | | 19.70113 | | 3-8 Block5 | | 181.4628 | | 1507.007 | | 3 8 in palet cinderblocks | | | cinderblocks | | | 3 pallets cinderblocks | | 3CB | | | low | | 8 | 1 | | 7.411738 | | Cinderblocks | | Pyramids |
| 539 | -1 | | 497.0394 | | 1 | 21.54278 | | 724.606 | | 77.46891 | | 3-4 Block11 | | 61.14735 | | 205.9266 | | 3 4 in palet cinderblocks | | | cinderblocks | | | 3 pallets cinderblocks | | 3CB | | | low | | 8 | 1 | | 7.208669 | | Cinderblocks | | Pyramids |
| 540 | -1 | | 293.2277 | | 1 | 22.49321 | | 560.9995 | | 57.30195 | | 3-6 Block6 | | 274.3334 | | 3209.422 | | 3 6 in pallets cinderblocks | | | cinderblocks | | | 3 pallets cinderblocks | | 3CB | | | low | | 8 | 1 | | 6.68095 | | Cinderblocks | | Pyramids |
| 541 | -1 | | 730.9902 | | 1 | 22.48692 | | 560.4114 | | 69.83906 | | 3-6 Block6 | | 274.3334 | | 3209.422 | | 3 6 in pallets cinderblocks | | | cinderblocks | | | 3 pallets cinderblocks | | 3CB | | | low | | 8 | 1 | | 7.5944 | | Cinderblocks | | Pyramids |
| 542 | -1 | | 1355.94 | | 1 | 21.47081 | | 1020.573 | | 63.86991 | | 3-mixed Block21 | | 121.5736 | | 1063.589 | | 3 pallets mixed size ciderblocks | | | cinderblocks | | | 3 pallets cinderblocks | | 3CB | | | low | | 8 | 1 | | 8.21225 | | Cinderblocks | | Pyramids |
| 543 | -1 | | 5496.715 | | 1 | 21.50374 | | 1032.702 | | 12.95695 | | 3-mixed Block21 | | 121.5736 | | 1063.589 | | 3 pallets mixed size ciderblocks | | | cinderblocks | | | 3 pallets cinderblocks | | 3CB | | | low | | 8 | 1 | | 9.611906 | | Cinderblocks | | Pyramids |
| 544 | -1 | | 458.4673 | | 1 | 21.67078 | | 964.4229 | | 43.6247 | | 3-mixed Block20 | | 137.4922 | | 983.9561 | | 3 pallets mixed size ciderblocks | | | cinderblocks | | | 3 pallets cinderblocks | | 3CB | | | low | | 8 | 1 | | 7.127889 | | Cinderblocks | | Pyramids |
| 545 | -1 | | 32.0308 | | 1 | 22.3446 | | 343.7431 | | 36.06282 | | 3-mixed Block27 | | 339.8356 | | 7239.474 | | 3 pallets mixed size cinderblocks | | | cinderblocks | | | 3 pallets cinderblocks | | 3CB | | | low | | 8 | 1 | | 4.466698 | | Cinderblocks | | Pyramids |
| 546 | -1 | | 67.14719 | | 3 | 22.92052 | | 289.5773 | | 39.93844 | | 3-8 Block13 | | 156.5938 | | 1242.676 | | 3 8 in pallets cinderblocks | | | cinderblocks | | | 3 pallets cinderblocks | | 3CB | | | low | | 8 | 1 | | 5.206887 | | Cinderblocks | | Pyramids |
| 547 | -1 | | 1635.62 | | 82 | 20.67154 | | 112.0886 | | 44.12731 | | 3-6 Block2 | | 291.9546 | | 3338.639 | | 3 6 in palet cinderblocks | | | cinderblocks | | | 3 pallets cinderblocks | | 3CB | | | low | | 8 | 1 | | 8.399777 | | Cinderblocks | | Pyramids |
| 548 | -1 | | 3789.344 | | 189 | 22.75218 | | 482.7797 | | 76.57922 | | 3-8 Block12 | | 219.5874 | | 2023.725 | | 3 8 in pallets cinderblocks | | | cinderblocks | | | 3 pallets cinderblocks | | 3CB | | | low | | 8 | 1 | | 9.239948 | | Cinderblocks | | Pyramids |
| 549 | -1 | | 3317.084 | | 1 | 21.99159 | | 445.3654 | | 16.88792 | | 3-4 Block10 | | 55.32938 | | 227.4086 | | 3 4 in palet cinderblocks | | | cinderblocks | | | 3 pallets cinderblocks | | 3CB | | | low | | 8 | 1 | | 9.106841 | | Cinderblocks | | Pyramids |
| 550 | -1 | | 1063.544 | | 1 | 22.12824 | | 595.7934 | | 56.07933 | | 3-8 Block4 | | 138.789 | | 1294.941 | | 3 8 in pallets cinderblocks | | | cinderblocks | | | 3 pallets cinderblocks | | 3CB | | | low | | 8 | 1 | | 7.969362 | | Cinderblocks | | RR Ties |
| 551 | -1 | | 1438.096 | | 1 | 22.99101 | | 373.0847 | | 6.344448 | | 3-4 Block5 | | 111.5065 | | 740.3079 | | 3 4 in palet cinderblocks | | | cinderblocks | | | 3 pallets cinderblocks | | 3CB | | | low | | 8 | 1 | | 8.271075 | | Cinderblocks | | RR Ties |
| 552 | -1 | | 1151.055 | | 1 | 22.86916 | | 354.6317 | | 23.56844 | | 3-4 Block8 | | 81.06252 | | 469.716 | | 3 4 in palet cinderblocks | | | cinderblocks | | | 3 pallets cinderblocks | | 3CB | | | low | | 8 | 1 | | 8.048434 | | Cinderblocks | | RR Ties |
| 553 | -1 | | 1275.922 | | 1 | 21.75564 | | 396.0064 | | 22.30349 | | 3-6 Block10 | | 213.7536 | | 2310.862 | | 3 6 in palet cinderblocks | | | cinderblocks | | | 3 pallets cinderblocks | | 3CB | | | low | | 8 | 1 | | 8.151424 | | Cinderblocks | | RR Ties |
| 554 | -1 | | 44.60028 | | 2 | 20.9258 | | 101.3308 | | 8.906478 | | 3-6 Block2 | | 291.9546 | | 3338.639 | | 3 6 in palet cinderblocks | | | cinderblocks | | | 3 pallets cinderblocks | | 3CB | | | low | | 8 | 1 | | 4.79774 | | Cinderblocks | | RR Ties |
| 555 | -1 | | 1032.793 | | 1 | 22.88637 | | 295.0365 | | 52.80055 | | 3-6 Block8 | | 233.9066 | | 2915.526 | | 3 6 in pallets cinderblocks | | | cinderblocks | | | 3 pallets cinderblocks | | 3CB | | | low | | 8 | 1 | | 7.940023 | | Cinderblocks | | RR Ties |
| 556 | -1 | | 2790.237 | | 1 | 21.45866 | | 913.6118 | | 37.23154 | | 3-6 Block9 | | 119.9936 | | 578.4422 | | 3 6 in palet cinderblocks | | | cinderblocks | | | 3 pallets cinderblocks | | 3CB | | | low | | 8 | 1 | | 8.933882 | | Cinderblocks | | RR Ties |
| 557 | -1 | | 1376.752 | | 1 | 22.7155 | | 398.5192 | | 17.60674 | | 3-8 Block10 | | 184.8973 | | 2135.207 | | 3 8 in pallets cinderblocks | | | cinderblocks | | | 3 pallets cinderblocks | | 3CB | | | low | | 8 | 1 | | 8.227483 | | Cinderblocks | | RR Ties |
| 558 | -1 | | 2155.51 | | 1 | 22.68734 | | 404.4364 | | 18.8001 | | 3-8 Block10 | | 184.8973 | | 2135.207 | | 3 8 in pallets cinderblocks | | | cinderblocks | | | 3 pallets cinderblocks | | 3CB | | | low | | 8 | 1 | | 8.675783 | | Cinderblocks | | Big Pile |
| 559 | -1 | | 5851.445 | | 1 | 22.75176 | | 443.3395 | | 23.99732 | | 3-8 Block11 | | 140.9602 | | 1105.631 | | 3 8 in pallets cinderblocks | | | cinderblocks | | | 3 pallets cinderblocks | | 3CB | | | low | | 8 | 1 | | 9.674444 | | Cinderblocks | | Big Pile |
| 560 | -1 | | 836.4511 | | 1 | 21.23988 | | 339.8528 | | 4.34901 | | 3-mixed Block24 | | 63.24544 | | 204.888 | | 3 pallets mixed size ciderblocks | | | cinderblocks | | | 3 pallets cinderblocks | | 3CB | | | low | | 8 | 1 | | 7.729168 | | Cinderblocks | | Big Pile |
| 561 | -1 | | 7601.332 | | 1 | 21.19791 | | 340.5204 | | 33.89589 | | 3-mixed Block24 | | 63.24544 | | 204.888 | | 3 pallets mixed size ciderblocks | | | cinderblocks | | | 3 pallets cinderblocks | | 3CB | | | low | | 8 | 1 | | 9.936079 | | Cinderblocks | | Big Pile |
| 562 | -1 | | 4263.663 | | 1 | 22.29338 | | 342.1496 | | 17.21378 | | 3-mixed Block27 | | 339.8356 | | 7239.474 | | 3 pallets mixed size cinderblocks | | | cinderblocks | | | 3 pallets cinderblocks | | 3CB | | | low | | 8 | 1 | | 9.357884 | | Cinderblocks | | Big Pile |
| 563 | -1 | | 79.92898 | | 1 | 22.71892 | | 336.6892 | | 3.776561 | | 3-mixed Block30 | | 241.2037 | | 3904.728 | | 3 pallets mixed size cinderblocks | | | cinderblocks | | | 3 pallets cinderblocks | | 3CB | | | low | | 8 | 1 | | 5.381138 | | Cinderblocks | | Big Pile |
| 564 | -1 | | 2282.33 | | 1 | 21.70769 | | 838.0264 | | 25.8106 | | 3-mixed Block39 | | 96.6829 | | 513.8501 | | 3 pallets mixed size ciderblocks | | | cinderblocks | | | 3 pallets cinderblocks | | 3CB | | | low | | 8 | 1 | | 8.732952 | | Cinderblocks | | Big Pile |
| 565 | -1 | | 81.22128 | | 1 | 21.20848 | | 277.9222 | | 87.01546 | | 3-mixed Block24 | | 63.24544 | | 204.888 | | 3 pallets mixed size ciderblocks | | | cinderblocks | | | 3 pallets cinderblocks | | 3CB | | | low | | 8 | 1 | | 5.397177 | | Cinderblocks | | Big Pile |
| 566 | -1 | | 1908.639 | | 1 | 22.25649 | | 522.883 | | 64.07548 | | 3-8 Block4 | | 138.789 | | 1294.941 | | 3 8 in pallets cinderblocks | | | cinderblocks | | | 3 pallets cinderblocks | | 3CB | | | low | | 8 | 1 | | 8.554146 | | Cinderblocks | | Big Pile |
| 567 | -1 | | 1987.455 | | 1 | 22.7777 | | 664.7755 | | 90.26353 | | 3-6 Block5 | | 42.86508 | | 123.3765 | | 3 6 in palet cinderblocks | | | cinderblocks | | | 3 pallets cinderblocks | | 3CB | | | low | | 8 | 1 | | 8.59461 | | Cinderblocks | | Cinderblocks |
| 568 | -1 | | 2780.259 | | 1 | 22.68965 | | 688.498 | | 78.56564 | | 3-8 Block6 | | 142.7701 | | 1038.886 | | 3 8 in pallets cinderblocks | | | cinderblocks | | | 3 pallets cinderblocks | | 3CB | | | low | | 8 | 1 | | 8.930299 | | Cinderblocks | | Cinderblocks |
| 569 | -1 | | 1703.964 | | 1 | 19.42478 | | 502.9995 | | 131.981 | | Patch 12 | | 262.2038 | | 3913.885 | | low profile | | | low profile | | | 4LP | | 4LP | | | low | | 8 | 1 | | 8.440713 | | Low Profile | | Cinderblocks |
| 570 | -1 | | 1569.413 | | 1 | 19.5982 | | 301.7161 | | 81.24162 | | Patch 47 | | 289.7673 | | 3959.842 | | low profile | | | low profile | | | 4LP | | 4LP | | | low | | 8 | 1 | | 8.358457 | | Low Profile | | Cinderblocks |
| 571 | -1 | | 20.19801 | | 1 | 20.22792 | | 583.2229 | | 39.76075 | | Patch 15 | | 300.8062 | | 5493.472 | | low profile | | | low profile | | | 4LP | | 4LP | | | low | | 8 | 1 | | 4.005584 | | Low Profile | | Cinderblocks |
| 572 | -1 | | 35.98706 | | 2 | 20.30906 | | 589.4804 | | 57.63015 | | Patch 15 | | 300.8062 | | 5493.472 | | low profile | | | low profile | | | 4LP | | 4LP | | | low | | 8 | 1 | | 4.583159 | | Low Profile | | Cinderblocks |
| 573 | -1 | | 83.95761 | | 4 | 20.31668 | | 520.242 | | 82.05868 | | Patch 15 | | 300.8062 | | 5493.472 | | low profile | | | low profile | | | 4LP | | 4LP | | | low | | 8 | 1 | | 5.430312 | | Low Profile | | Cinderblocks |
| 574 | -1 | | 238.879 | | 1 | 19.25494 | | 244.6953 | | 83.87591 | | Patch 47 | | 289.7673 | | 3959.842 | | low profile | | | low profile | | | 4LP | | 4LP | | | low | | 8 | 1 | | 6.475957 | | Low Profile | | Cinderblocks |
| 575 | -1 | | 549.6793 | | 1 | 19.29656 | | 299.0916 | | 15.76155 | | Patch 47 | | 289.7673 | | 3959.842 | | low profile | | | low profile | | | 4LP | | 4LP | | | low | | 8 | 1 | | 7.309335 | | Low Profile | | Cinderblocks |
| 576 | -1 | | 483.4837 | | 1 | 19.24926 | | 244.0121 | | 30.17851 | | Patch 16 | | 409.546 | | 6681.443 | | mixed; pyramids, lpm | | | pyramids, low profile | | | 4MX | | 4MX | | | mid | | 8 | 2 | | 7.181018 | | Pyramids | | Cinderblocks |
| 577 | -1 | | 252.5713 | | 1 | 20.27017 | | 864.7256 | | 75.06973 | | Patch 25 | | 346.4492 | | 8016.418 | | mixed; pyramids, lpm | | | pyramids, low profile | | | 4MX | | 4MX | | | mid | | 8 | 2 | | 6.531694 | | Pyramids | | Cinderblocks |
| 578 | -1 | | 10146.18 | | 1 | 19.1596 | | 441.6953 | | 93.59429 | | Patch 7 | | 362.0707 | | 7757.877 | | mixed; pyramids, lpm | | | pyramids, low profile | | | 4MX | | 4MX | | | mid | | 8 | 2 | | 10.22485 | | Pyramids | | Cinderblocks |
| 579 | -1 | | 21.01496 | | 1 | 20.332 | | 841.0158 | | 104.5221 | | Patch 25 | | 346.4492 | | 8016.418 | | mixed; pyramids, lpm | | | pyramids, low profile | | | 4MX | | 4MX | | | mid | | 8 | 2 | | 4.045235 | | Pyramids | | Cinderblocks |
| 580 | -1 | | 32.07656 | | 2 | 20.35563 | | 842.6477 | | 88.82502 | | Patch 25 | | 346.4492 | | 8016.418 | | mixed; pyramids, lpm | | | pyramids, low profile | | | 4MX | | 4MX | | | mid | | 8 | 2 | | 4.468126 | | Pyramids | | Cinderblocks |
| 581 | -1 | | 5827.75 | | 1 | 20.41423 | | 379.0783 | | 17.71106 | | Patch 10 | | 307.7802 | | 5655.625 | | mixed; pyramids, lpm | | | pyramids, low profile | | | 4MX | | 4MX | | | mid | | 8 | 2 | | 9.670386 | | Pyramids | | Cinderblocks |
| 582 | -1 | | 7089.066 | | 1 | 20.39695 | | 944.4401 | | 38.5784 | | Patch 25 | | 346.4492 | | 8016.418 | | mixed; pyramids, lpm | | | pyramids, low profile | | | 4MX | | 4MX | | | mid | | 8 | 2 | | 9.866309 | | Pyramids | | Concrete |
| 583 | -1 | | 3043.105 | | 1 | 19.13849 | | 184.6168 | | 82.78497 | | Patch 36 | | 312.6145 | | 5909.587 | | pyramids | | | pyramids | | | 4PY | | 4PY | | | mid | | 8 | 1 | | 9.020634 | | Pyramids | | Concrete |
| 584 | -1 | | 3829.069 | | 1 | 20.2866 | | 491.7819 | | 53.42975 | | Patch 45 | | 249.6864 | | 3879.805 | | pyramids | | | pyramids | | | 4PY | | 4PY | | | mid | | 8 | 1 | | 9.250377 | | Pyramids | | Concrete |
| 585 | -1 | | 81.80646 | | 1 | 20.08401 | | 785.0593 | | 104.6697 | | Patch 24 | | 248.2686 | | 3252.476 | | pyramids | | | pyramids | | | 4PY | | 4PY | | | mid | | 8 | 1 | | 5.404356 | | Pyramids | | Concrete |
| 586 | -1 | | 509.0244 | | 1 | 19.7084 | | 115.0112 | | 190.9389 | | Patch 3 | | 324.2377 | | 5122.985 | | pyramids | | | pyramids | | | 4PY | | 4PY | | | mid | | 8 | 1 | | 7.232496 | | Pyramids | | Low Profile |
| 587 | -1 | | 20.83341 | | 1 | 19.59352 | | 251.4292 | | 80.13525 | | Patch 3 | | 324.2377 | | 5122.985 | | pyramids | | | pyramids | | | 4PY | | 4PY | | | mid | | 8 | 1 | | 4.036558 | | Pyramids | | Low Profile |
| 588 | -1 | | 29.08896 | | 1 | 19.19352 | | 228.2919 | | 41.8137 | | Patch 36 | | 312.6145 | | 5909.587 | | pyramids | | | pyramids | | | 4PY | | 4PY | | | mid | | 8 | 1 | | 4.370359 | | Pyramids | | Low Profile |
| 589 | -1 | | 31.16959 | | 2 | 19.85494 | | 94.31022 | | 198.8068 | | Patch 3 | | 324.2377 | | 5122.985 | | pyramids | | | pyramids | | | 4PY | | 4PY | | | mid | | 8 | 1 | | 4.439443 | | Pyramids | | RR Ties |
| 590 | -1 | | 41.65367 | | 2 | 19.19605 | | 231.5434 | | 52.69394 | | Patch 3 | | 324.2377 | | 5122.985 | | pyramids | | | pyramids | | | 4PY | | 4PY | | | mid | | 8 | 1 | | 4.729389 | | Pyramids | | RR Ties |
| 591 | -1 | | 50.31047 | | 3 | 19.1089 | | 168.9831 | | 57.20329 | | Patch 3 | | 324.2377 | | 5122.985 | | pyramids | | | pyramids | | | 4PY | | 4PY | | | mid | | 8 | 1 | | 4.918213 | | Pyramids | | RR Ties |
| 592 | -1 | | 38132.73 | | 1907 | 19.93522 | | 27.61259 | | 235.4225 | | Patch 3 | | 324.2377 | | 5122.985 | | pyramids | | | pyramids | | | 4PY | | 4PY | | | mid | | 8 | 1 | | 11.54883 | | Pyramids | | RR Ties |
| 593 | -1 | | 200.4951 | | 1 | 20.11132 | | 561.2809 | | 5.930206 | | Patch 33 | | 188.9853 | | 1770.835 | | pyramids | | | pyramids | | | 4PY | | 4PY | | | mid | | 8 | 1 | | 6.30079 | | Pyramids | | RR Ties |
| 594 | -1 | | 2311.371 | | 1 | 19.95255 | | 585.5213 | | 20.04998 | | Patch 33 | | 188.9853 | | 1770.835 | | pyramids | | | pyramids | | | 4PY | | 4PY | | | mid | | 8 | 1 | | 8.745596 | | Pyramids | | RR Ties |
| 595 | -1 | | 450.1519 | | 1 | 19.42337 | | 201.932 | | 27.58057 | | Patch 36 | | 312.6145 | | 5909.587 | | pyramids | | | pyramids | | | 4PY | | 4PY | | | mid | | 8 | 1 | | 7.109585 | | Pyramids | | RR Ties |
| 596 | -1 | | 2708.621 | | 1 | 20.41792 | | 480.8313 | | 38.14282 | | Patch 45 | | 249.6864 | | 3879.805 | | pyramids | | | pyramids | | | 4PY | | 4PY | | | mid | | 8 | 1 | | 8.904195 | | Pyramids | | Other / Mixed |
| 597 | -1 | | 130.758 | | 7 | 20.54719 | | 435.2255 | | 28.01088 | | Patch 45 | | 249.6864 | | 3879.805 | | pyramids | | | pyramids | | | 4PY | | 4PY | | | mid | | 8 | 1 | | 5.873348 | | Pyramids | | Other / Mixed |
| 598 | -1 | | 2466.754 | | 1 | 20.88455 | | 255.0675 | | 23.62857 | | Patch 53 | | 329.6576 | | 6980.813 | | pyramids | | | pyramids | | | 4PY | | 4PY | | | mid | | 8 | 1 | | 8.810659 | | Pyramids | | Other / Mixed |
| 599 | -1 | | 3561.453 | | 1 | 20.99331 | | 257.7764 | | 9.499559 | | Patch 53 | | 329.6576 | | 6980.813 | | pyramids | | | pyramids | | | 4PY | | 4PY | | | mid | | 8 | 1 | | 9.177924 | | Pyramids | | Other / Mixed |
| 600 | -1 | | 152.3372 | | 8 | 20.82132 | | 440.8714 | | 32.93245 | | Patch 54 | | 416.7709 | | 9568.277 | | pyramids | | | pyramids | | | 4PY | | 4PY | | | mid | | 8 | 1 | | 6.026096 | | Pyramids | | Other / Mixed |
| 601 | -1 | | 78.57851 | | 4 | 20.69308 | | 277.9492 | | 66.53283 | | 500T_2 | | 431.6438 | | 12308.43 | | 500 rr ties | | | rr ties | | | 500T RR | | 500T RR | | | high | | 6 | 1 | | 5.364098 | | RR Ties | | Other / Mixed |
| 602 | -1 | | 959.9353 | | 1 | 21.21085 | | 342.2794 | | 2.243922 | | 500T_2 | | 431.6438 | | 12308.43 | | 500 rr ties | | | rr ties | | | 500T RR | | 500T RR | | | high | | 6 | 1 | | 7.866866 | | RR Ties | | Other / Mixed |
| 603 | -1 | | 1153.462 | | 1 | 22.16318 | | 926.0255 | | 2.519855 | | 50.1 | | 345.7408 | | 6620.539 | | 50 rr tie | | | rr ties | | | 50 rr tie | | 50T RR | | | low-mid | | 8 | 1 | | 8.050523 | | RR Ties | | Other / Mixed |
| 604 | -1 | | 11828.92 | | 1 | 22.76871 | | 682.4234 | | 99.17243 | | 67.2 | | 309.6253 | | 5393.584 | | 67 rr tie | | | rr ties | | | 67 rr tie | | 67T RR | | | low-mid | | 8 | 1 | | 10.3783 | | RR Ties | | Other / Mixed |
| 605 | -1 | | 1136.915 | | 1 | 21.70264 | | 893.8697 | | 53.40429 | | 67.1 | | 274.4974 | | 5166.366 | | 67 rr tie | | | rr ties | | | 67 rr tie | | 67T RR | | | low-mid | | 8 | 1 | | 8.036074 | | RR Ties | | Other / Mixed |
| 606 | -1 | | 1508.393 | | 1 | 23.0031 | | 256.3631 | | 58.78422 | | Big Pile | | 771.4804 | | 34964.52 | | big pile | | | big pile | | | big pile | | Big Pile | | | high | | 8 | 1 | | 8.3188 | | Big Pile | | Other / Mixed |
| 607 | -1 | | 2034.779 | | 1 | 23.04831 | | 231.1025 | | 67.90989 | | Big Pile | | 771.4804 | | 34964.52 | | big pile | | | big pile | | | big pile | | Big Pile | | | high | | 8 | 1 | | 8.618143 | | Big Pile | | Other / Mixed |
| 608 | -1 | | 6346.964 | | 1 | 23.21816 | | 245.3162 | | 56.99098 | | Big Pile | | 771.4804 | | 34964.52 | | big pile | | | big pile | | | big pile | | Big Pile | | | high | | 8 | 1 | | 9.755732 | | Big Pile | | Other / Mixed |
| 609 | -1 | | 2775722 | | 138786 | 23.17039 | | 298.6266 | | 20.26202 | | Big Pile | | 771.4804 | | 34964.52 | | big pile | | | big pile | | | big pile | | Big Pile | | | high | | 8 | 1 | | 15.83642 | | Big Pile | | Other / Mixed |
| 610 | -1 | | 177.0156 | | 1 | 23.12074 | | 116.0204 | | 21.25982 | | Murray Meg | | 206.0817 | | 2375.573 | | boat | | | boat | | | boat | | Boats | | | high | | 6 | 1 | | 6.176238 | | Boat | | Other / Mixed |
| 611 | -1 | | 9028.01 | | 1 | 21.52732 | | 150.482 | | 44.96735 | | RGV Shrimper | | 217.0793 | | 1568.787 | | boat | | | boat | | | boat | | Boats | | | high | | 10 | 1 | | 10.10809 | | Boat | | Other / Mixed |
| 612 | -1 | | 473.7953 | | 1 | 21.74946 | | 867.0853 | | 50.74365 | | SPI Ceviche | | 171.3348 | | 1110.387 | | boat | | | boat | | | boat | | Boats | | | high | | 5 | 1 | | 7.160775 | | Boat | | Other / Mixed |
| 613 | -1 | | 4474.222 | | 1 | 21.82915 | | 687.1144 | | 65.30378 | | Billy Kenon | | 134.7889 | | 934.4976 | | boat | | | boat | | | boat | | Boats | | | high | | 5 | 1 | | 9.406088 | | Boat | | Other / Mixed |
| 614 | -1 | | 775.9765 | | 1 | 20.78046 | | 753.0421 | | 13.15506 | | Andy Faskin | | 87.86762 | | 431.0793 | | boat | | | boat | | | boat | | Boats | | | high | | 5 | 1 | | 7.654122 | | Boat | | Other / Mixed |
| 615 | -1 | | 11019.6 | | 551 | 21.59402 | | 812.2422 | | 14.29769 | | SPI Ceviche | | 171.3348 | | 1110.387 | | boat | | | boat | | | boat | | Boats | | | high | | 5 | 1 | | 10.30743 | | Boat | | Other / Mixed |
| 616 | -1 | | 601.3224 | | 1 | 20.84254 | | 376.6716 | | 42.62537 | | RGV Tug | | 153.2792 | | 1220.279 | | boat | | | boat | | | boat | | Boats | | | high | | 10 | 1 | | 7.399131 | | Boat | | Other / Mixed |
| 617 | -1 | | 447.8105 | | 1 | 21.4926 | | 246.9078 | | 22.99426 | | Block UK4 | | 100.2363 | | 651.2407 | | unknown size pallet of cinderblocks | | | cinderblocks | | | UK pallets cinderblocks | | CB | | | low | | 8 | 1 | | 7.10437 | | Cinderblocks | | Other / Mixed |
| 618 | -1 | | 490.8505 | | 1 | 21.41939 | | 190.4295 | | 38.23932 | | Block UK1 | | 42.50869 | | 103.0169 | | unknown size pallet of cinderblocks | | | cinderblocks | | | uk pallets cinderblocks | | CB | | | low | | 8 | 1 | | 7.19614 | | Cinderblocks | | Other / Mixed |
| 619 | -1 | | 510.8842 | | 1 | 21.6222 | | 143.9664 | | 9.595277 | | Block UK1 | | 42.50869 | | 103.0169 | | unknown size pallet of cinderblocks | | | cinderblocks | | | uk pallets cinderblocks | | CB | | | low | | 8 | 1 | | 7.236143 | | Cinderblocks | | Other / Mixed |
| 620 | -1 | | 435.2558 | | 1 | 21.98475 | | 520.1711 | | 167.2053 | | Block UK6 | | 47.55979 | | 157.1565 | | unknown size pallet of cinderblocks | | | cinderblocks | | | uk pallets cinderblocks | | CB | | | low | | 8 | 1 | | 7.075934 | | Cinderblocks | | Other / Mixed |
| 621 | -1 | | 537.0403 | | 27 | 21.4919 | | 279.5234 | | 34.72252 | | Block UK4 | | 100.2363 | | 651.2407 | | unknown size pallet of cinderblocks | | | cinderblocks | | | UK pallets cinderblocks | | CB | | | low | | 8 | 1 | | 7.286073 | | Cinderblocks | | Other / Mixed |
| 622 | -1 | | 1664.859 | | 1 | 21.49033 | | 255.2103 | | 14.96662 | | Block UK4 | | 100.2363 | | 651.2407 | | unknown size pallet of cinderblocks | | | cinderblocks | | | UK pallets cinderblocks | | CB | | | low | | 8 | 1 | | 8.417495 | | Cinderblocks | | Other / Mixed |
| 623 | -1 | | 3754.062 | | 1 | 21.50226 | | 258.7323 | | 20.80145 | | Block UK4 | | 100.2363 | | 651.2407 | | unknown size pallet of cinderblocks | | | cinderblocks | | | UK pallets cinderblocks | | CB | | | low | | 8 | 1 | | 9.230594 | | Cinderblocks | | Other / Mixed |
| 624 | -1 | | 812.5641 | | 1 | 21.47116 | | 295.7819 | | 63.90464 | | Block UK4 | | 100.2363 | | 651.2407 | | unknown size pallet of cinderblocks | | | cinderblocks | | | UK pallets cinderblocks | | CB | | | low | | 8 | 1 | | 7.700195 | | Cinderblocks | | Other / Mixed |
| 625 | -1 | | 951.4133 | | 1 | 22.53394 | | 531.7843 | | 92.80628 | | 3mb ridge | | 2104.209 | | 64058.34 | | 3 pallets mixed size cinderblocks in line | | | cinderblocks | | | cinderblock ridge | | CB Ridge | | | low | | 8 | 1 | | 7.857949 | | Cinderblocks | | Other / Mixed |
| 626 | -1 | | 7601.777 | | 1 | 22.28628 | | 363.2837 | | 13.33667 | | Patch 104 | | 79.63223 | | 329.7727 | | mixed patch w/ 2 8 in palet cinderblocks   low profile chunks of concrete | | | cinderblocks, concrete | | | cinderblocks, concrete | | CB, Concrete | | | low | | 9 | 2 | | 9.936137 | | Other / Mixed | | Other / Mixed |
| 627 | -1 | | 1392.105 | | 1 | 23.31483 | | 117.069 | | 33.58831 | | Patch 87 | | 282.7408 | | 4011.823 | | mixed patch w/ 2 8 in palet cinderblocks  low profile chunks of concrete  low profile chunks of concrete | | | cinderblocks, concrete | | | cinderblocks, concrete | | CB, Concrete | | | low | | 9 | 2 | | 8.238572 | | Other / Mixed | | Other / Mixed |
| 628 | -1 | | 665.1988 | | 1 | 22.57585 | | 208.7831 | | 78.88066 | | Patch 83 | | 261.7858 | | 4026.786 | | mixed patch w/ 1 6 in palet cinderblocks  2 8 in palet cinderblocks  2 10x10 box culverts  3 pieces concrete highway divider | | | cinderblocks, culverts, highway divider | | | cinderblocks, culverts, highway divider | | CB, Culvert, HD | | | mid-high | | 9 | 3 | | 7.500086 | | Other / Mixed | | Other / Mixed |
| 629 | -1 | | 8594.852 | | 1 | 22.56188 | | 242.4003 | | 38.96972 | | Patch 83 | | 261.7858 | | 4026.786 | | mixed patch w/ 1 6 in palet cinderblocks  2 8 in palet cinderblocks  2 10x10 box culverts  3 pieces concrete highway divider | | | cinderblocks, culverts, highway divider | | | cinderblocks, culverts, highway divider | | CB, Culvert, HD | | | mid-high | | 9 | 3 | | 10.05892 | | Other / Mixed | | Other / Mixed |
| 630 | -1 | | 22.72513 | | 1 | 22.56307 | | 330.8819 | | 67.56284 | | Patch 83 | | 261.7858 | | 4026.786 | | mixed patch w/ 1 6 in palet cinderblocks  2 8 in palet cinderblocks  2 10x10 box culverts  3 pieces concrete highway divider | | | cinderblocks, culverts, highway divider | | | cinderblocks, culverts, highway divider | | CB, Culvert, HD | | | mid-high | | 9 | 3 | | 4.123471 | | Other / Mixed | | Other / Mixed |
| 631 | -1 | | 171.8311 | | 1 | 23.01909 | | 103.6472 | | 38.75888 | | Patch 98 | | 175.0049 | | 1736.466 | | mixed patch w/ 1 6 in palet cinderblocks   2 8 in palet cinderblocks   2 10x10 box culverts  3 pieces concrete highway divider  low profile chunks of concrete | | | cinderblocks, culverts, highway divider, concrete | | | cinderblocks, culverts, highway divider, concrete | | CB, Culvert, HD, Concrete | | | mid-high | | 9 | 4 | | 6.146512 | | Other / Mixed | | Other / Mixed |
| 632 | -1 | | 2350.119 | | 1 | 23.20623 | | 118.9079 | | 17.16662 | | Patch 91 | | 211.001 | | 2526.413 | | mixed patch w/ 2 8 in palet cinderblocks  2 10x10 box culverts  3 pieces concrete highway divider  low profile chunks of concrete | | | cinderblocks, culverts, highway divider, concrete | | | cinderblocks, culverts, highway divider, concrete | | CB, Culvert, HD, Concrete | | | mid-high | | 9 | 4 | | 8.762221 | | Other / Mixed | | Other / Mixed |
| 633 | -1 | | 719.0871 | | 1 | 23.15745 | | 181.8115 | | 9.446213 | | Patch 95 | | 194.3622 | | 2266.444 | | mixed patch w/ 1 6 in palet cinderblocks  2 8 in palet cinderblocks  2 10x10 box culverts  3 pieces concrete highway divider  low profile chunks of concrete | | | cinderblocks, culverts, highway divider, concrete | | | cinderblocks, culverts, highway divider, concrete | | CB, Culvert, HD, Concrete | | | mid-high | | 9 | 4 | | 7.577982 | | Other / Mixed | | Other / Mixed |
| 634 | -1 | | 372.4334 | | 1 | 23.02598 | | 126.2004 | | 17.25226 | | Patch 98 | | 175.0049 | | 1736.466 | | mixed patch w/ 1 6 in palet cinderblocks   2 8 in palet cinderblocks   2 10x10 box culverts  3 pieces concrete highway divider  low profile chunks of concrete | | | cinderblocks, culverts, highway divider, concrete | | | cinderblocks, culverts, highway divider, concrete | | CB, Culvert, HD, Concrete | | | mid-high | | 9 | 4 | | 6.920058 | | Other / Mixed | | Other / Mixed |
| 635 | -1 | | 265.4341 | | 1 | 23.19102 | | 110.815 | | 45.38917 | | Patch 99 | | 275.534 | | 4359.957 | | mixed patch w/ 1 6 in palet cinderblocks  2 8 in palet cinderblocks  3 pieces concrete highway divider  5 round culverts  low profile chunks of concrete | | | cinderblocks, culverts, highway divider, concrete | | | cinderblocks, culverts, highway divider, concrete | | CB, Culvert, HD, Concrete | | | mid-high | | 9 | 4 | | 6.581367 | | Other / Mixed | | Other / Mixed |
| 636 | -1 | | 640.817 | | 1 | 23.07124 | | 68.82894 | | 83.63362 | | Patch 99 | | 275.534 | | 4359.957 | | mixed patch w/ 1 6 in palet cinderblocks  2 8 in palet cinderblocks  3 pieces concrete highway divider  5 round culverts  low profile chunks of concrete | | | cinderblocks, culverts, highway divider, concrete | | | cinderblocks, culverts, highway divider, concrete | | CB, Culvert, HD, Concrete | | | mid-high | | 9 | 4 | | 7.462744 | | Other / Mixed | | Other / Mixed |
| 637 | -1 | | 5849.914 | | 1 | 23.175 | | 165.7368 | | 23.37454 | | Patch 98 | | 175.0049 | | 1736.466 | | mixed patch w/ 1 6 in palet cinderblocks   2 8 in palet cinderblocks   2 10x10 box culverts  3 pieces concrete highway divider  low profile chunks of concrete | | | cinderblocks, culverts, highway divider, concrete | | | cinderblocks, culverts, highway divider, concrete | | CB, Culvert, HD, Concrete | | | mid-high | | 9 | 4 | | 9.674182 | | Other / Mixed | | Other / Mixed |
| 638 | -1 | | 1429.095 | | 1 | 23.08529 | | 172.2537 | | 13.97787 | | Patch 97 | | 276.2059 | | 4294.15 | | mixed patch w/ 2 8 in palet cinderblocks  3 pieces concrete highway divider  5 round culverts  low profile chunks of concrete  low profile chunks of concrete | | | cinderblocks, culverts, highway divider, concrete | | | cinderblocks, culverts, highway divider, concrete | | CB, Culvert, HD, Concrete | | | mid-high | | 9 | 4 | | 8.264797 | | Other / Mixed | | Other / Mixed |
| 639 | -1 | | 1211.389 | | 1 | 22.80681 | | 165.8444 | | 32.54019 | | Patch 97 | | 276.2059 | | 4294.15 | | mixed patch w/ 2 8 in palet cinderblocks  3 pieces concrete highway divider  5 round culverts  low profile chunks of concrete  low profile chunks of concrete | | | cinderblocks, culverts, highway divider, concrete | | | cinderblocks, culverts, highway divider, concrete | | CB, Culvert, HD, Concrete | | | mid-high | | 9 | 4 | | 8.099523 | | Other / Mixed | | Other / Mixed |
| 640 | -1 | | 1606.365 | | 1 | 20.62572 | | 534.5181 | | 31.07839 | | Patch 66 | | 329.4691 | | 6463.169 | | mixed patch w/ 1 6 in palet cinderblocks  2 8 in palet cinderblocks  2 10x10 box culverts  3 pieces concrete highway divider  low profile chunks of concrete | | | cinderblocks, culverts, highway divider, concrete | | | cinderblocks, culverts, highway divider, concrete | | CB, Culvert, HD, Concrete | | | mid-high | | 9 | 4 | | 8.381729 | | Other / Mixed | | Other / Mixed |
| 641 | -1 | | 825.6243 | | 1 | 20.92155 | | 422.8403 | | 12.71862 | | Patch 65 | | 192.3443 | | 2532.184 | | mixed patch w/ 1 6 in palet cinderblocks   2 8 in palet cinderblocks   2 10x10 box culverts  3 pieces concrete highway divider  low profile chunks of concrete | | | cinderblocks, culverts, highway divider, concrete | | | cinderblocks, culverts, highway divider, concrete | | CB, Culvert, HD, Concrete | | | mid-high | | 9 | 4 | | 7.71614 | | Other / Mixed | | Other / Mixed |
| 642 | -1 | | 1841.73 | | 1 | 22.79215 | | 250.6812 | | 22.0063 | | Patch 85 | | 137.7693 | | 1059.623 | | mixed patch w/ 1 6 in palet cinderblocks  2 8 in palet cinderblocks  2 10x10 box culverts  low profile chunks of concrete  low profile chunks of concrete | | | cinderblocks, culverts, highway divider, concrete | | | cinderblocks, culverts, highway divider, concrete | | CB, Culvert, HD, Concrete | | | mid-high | | 9 | 4 | | 8.51846 | | Other / Mixed | | Other / Mixed |
| 643 | -1 | | 1594.375 | | 1 | 22.96646 | | 295.0374 | | 39.1498 | | Patch 86 | | 317.8525 | | 4563.282 | | mixed patch w/ 1 6 in palet cinderblocks  2 8 in palet cinderblocks  2 10x10 box culverts  3 pieces concrete highway divider  low profile chunks of concrete  low profile chunks of concrete | | | cinderblocks, culverts, highway divider, concrete | | | cinderblocks, culverts, highway divider, concrete | | CB, Culvert, HD, Concrete | | | mid-high | | 9 | 4 | | 8.374237 | | Other / Mixed | | Other / Mixed |
| 644 | -1 | | 757.1674 | | 1 | 23.00174 | | 134.5857 | | 32.24932 | | Patch 97 | | 276.2059 | | 4294.15 | | mixed patch w/ 2 8 in palet cinderblocks  3 pieces concrete highway divider  5 round culverts  low profile chunks of concrete  low profile chunks of concrete | | | cinderblocks, culverts, highway divider, concrete | | | cinderblocks, culverts, highway divider, concrete | | CB, Culvert, HD, Concrete | | | mid-high | | 9 | 4 | | 7.629584 | | Other / Mixed | | Other / Mixed |
| 645 | -1 | | 611.8512 | | 1 | 21.17813 | | 463.6196 | | 58.74269 | | Patch 73 | | 139.6919 | | 1347.989 | | mixed patch w/ 1 6 in palet cinderblocks  2 8 in palet cinderblocks  2 10x10 box culverts  3 pieces concrete highway divider  low profile chunks of concrete | | | cinderblocks, culverts, highway divider, concrete | | | cinderblocks, culverts, highway divider, concrete | | CB, Culvert, HD, Concrete | | | mid-high | | 9 | 4 | | 7.416489 | | Other / Mixed | | Other / Mixed |
| 646 | -1 | | 626.3858 | | 1 | 20.91797 | | 580.4752 | | 31.40937 | | Patch 72 | | 222.1526 | | 2646.466 | | mixed patch w/ 1 6 in palet cinderblocks  2 8 in palet cinderblocks  2 10x10 box culverts  3 pieces concrete highway divider  low profile chunks of concrete | | | cinderblocks, culverts, highway divider, concrete | | | cinderblocks, culverts, highway divider, concrete | | CB, Culvert, HD, Concrete | | | mid-high | | 9 | 4 | | 7.439966 | | Other / Mixed | | Other / Mixed |
| 647 | -1 | | 3891.965 | | 1 | 20.8211 | | 686.9249 | | 107.0427 | | Patch 72 | | 222.1526 | | 2646.466 | | mixed patch w/ 1 6 in palet cinderblocks  2 8 in palet cinderblocks  2 10x10 box culverts  3 pieces concrete highway divider  low profile chunks of concrete | | | cinderblocks, culverts, highway divider, concrete | | | cinderblocks, culverts, highway divider, concrete | | CB, Culvert, HD, Concrete | | | mid-high | | 9 | 4 | | 9.26667 | | Other / Mixed | | Other / Mixed |
| 648 | -1 | | 1821.999 | | 1 | 22.212 | | 251.296 | | 41.05634 | | Patch 81 | | 286.0177 | | 4739.138 | | mixed patch w/ 1 6 in palet cinderblocks  2 8 in palet cinderblocks  2 10x10 box culverts  3 pieces concrete highway divider  low profile chunks of concrete | | | cinderblocks, culverts, highway divider, concrete | | | cinderblocks, culverts, highway divider, concrete | | CB, Culvert, HD, Concrete | | | mid-high | | 9 | 4 | | 8.50769 | | Other / Mixed | | Other / Mixed |
| 649 | -1 | | 1970.468 | | 1 | 23.17565 | | 175.5409 | | 26.90986 | | Patch 95 | | 194.3622 | | 2266.444 | | mixed patch w/ 1 6 in palet cinderblocks  2 8 in palet cinderblocks  2 10x10 box culverts  3 pieces concrete highway divider  low profile chunks of concrete | | | cinderblocks, culverts, highway divider, concrete | | | cinderblocks, culverts, highway divider, concrete | | CB, Culvert, HD, Concrete | | | mid-high | | 9 | 4 | | 8.586026 | | Other / Mixed | | Other / Mixed |
| 650 | -1 | | 678.4623 | | 1 | 20.50994 | | 506.8979 | | 46.21634 | | Patch 66 | | 329.4691 | | 6463.169 | | mixed patch w/ 1 6 in palet cinderblocks  2 8 in palet cinderblocks  2 10x10 box culverts  3 pieces concrete highway divider  low profile chunks of concrete | | | cinderblocks, culverts, highway divider, concrete | | | cinderblocks, culverts, highway divider, concrete | | CB, Culvert, HD, Concrete | | | mid-high | | 9 | 4 | | 7.519829 | | Other / Mixed | | Other / Mixed |
| 651 | -1 | | 127.3353 | | 1 | 21.48594 | | 270.9802 | | 30.87009 | | Patch 75 | | 203.2362 | | 1807.639 | | mixed patch w/ 1 6 in palet cinderblocks   2 8 in palet cinderblocks   2 10x10 box culverts  3 pieces concrete highway divider  low profile chunks of concrete | | | cinderblocks, culverts, highway divider, concrete | | | cinderblocks, culverts, highway divider, concrete | | CB, Culvert, HD, Concrete | | | mid-high | | 9 | 4 | | 5.846824 | | Other / Mixed | | Other / Mixed |
| 652 | -1 | | 1025.296 | | 1 | 21.44018 | | 241.3268 | | 44.25897 | | Patch 76 | | 275.5071 | | 4518.707 | | mixed patch w/ 1 6 in palet cinderblocks  2 8 in palet cinderblocks  2 10x10 box culverts  3 pieces concrete highway divider  low profile chunks of concrete | | | cinderblocks, culverts, highway divider, concrete | | | cinderblocks, culverts, highway divider, concrete | | CB, Culvert, HD, Concrete | | | mid-high | | 9 | 4 | | 7.932736 | | Other / Mixed | | Other / Mixed |
| 653 | -1 | | 42.54532 | | 2 | 20.30924 | | 619.9691 | | 107.9261 | | Patch 66 | | 329.4691 | | 6463.169 | | mixed patch w/ 1 6 in palet cinderblocks  2 8 in palet cinderblocks  2 10x10 box culverts  3 pieces concrete highway divider  low profile chunks of concrete | | | cinderblocks, culverts, highway divider, concrete | | | cinderblocks, culverts, highway divider, concrete | | CB, Culvert, HD, Concrete | | | mid-high | | 9 | 4 | | 4.75057 | | Other / Mixed | | Other / Mixed |
| 654 | -1 | | 70.76305 | | 4 | 20.43785 | | 629.2107 | | 109.9003 | | Patch 67 | | 339.7311 | | 8294.548 | | mixed patch w/ 1 6 in palet cinderblocks  2 8 in palet cinderblocks  2 10x10 box culverts  3 pieces concrete highway divider  low profile chunks of concrete | | | cinderblocks, culverts, highway divider, concrete | | | cinderblocks, culverts, highway divider, concrete | | CB, Culvert, HD, Concrete | | | mid-high | | 9 | 4 | | 5.259337 | | Other / Mixed | | Other / Mixed |
| 655 | -1 | | 99.61042 | | 5 | 20.54218 | | 681.7677 | | 80.17551 | | Patch 67 | | 339.7311 | | 8294.548 | | mixed patch w/ 1 6 in palet cinderblocks  2 8 in palet cinderblocks  2 10x10 box culverts  3 pieces concrete highway divider  low profile chunks of concrete | | | cinderblocks, culverts, highway divider, concrete | | | cinderblocks, culverts, highway divider, concrete | | CB, Culvert, HD, Concrete | | | mid-high | | 9 | 4 | | 5.601267 | | Other / Mixed | | Other / Mixed |
| 656 | -1 | | 136.4779 | | 7 | 21.08063 | | 1027.947 | | 96.37668 | | Patch 71 | | 285.8251 | | 5126.988 | | mixed patch w/ 2 8 in palet cinderblocks  2 10x10 box culverts  3 pieces concrete highway divider  3 pieces concrete highway divider  low profile chunks of concrete | | | cinderblocks, culverts, highway divider, concrete | | | cinderblocks, culverts, highway divider, concrete | | CB, Culvert, HD, Concrete | | | mid-high | | 9 | 4 | | 5.916163 | | Other / Mixed | | Other / Mixed |
| 657 | -1 | | 893.973 | | 45 | 20.40476 | | 605.0685 | | 75.39884 | | Patch 66 | | 329.4691 | | 6463.169 | | mixed patch w/ 1 6 in palet cinderblocks  2 8 in palet cinderblocks  2 10x10 box culverts  3 pieces concrete highway divider  low profile chunks of concrete | | | cinderblocks, culverts, highway divider, concrete | | | cinderblocks, culverts, highway divider, concrete | | CB, Culvert, HD, Concrete | | | mid-high | | 9 | 4 | | 7.795676 | | Other / Mixed | | Other / Mixed |
| 658 | -1 | | 1819.251 | | 1 | 21.598 | | 231.1546 | | 41.68251 | | Patch 77 | | 311.3754 | | 5600.178 | | mixed patch w/ 1 6 in palet cinderblocks  2 8 in palet cinderblocks  2 10x10 box culverts  3 pieces concrete highway divider  low profile chunks of concrete | | | cinderblocks, culverts, highway divider, concrete | | | cinderblocks, culverts, highway divider, concrete | | CB, Culvert, HD, Concrete | | | mid-high | | 9 | 4 | | 8.50618 | | Other / Mixed | | Other / Mixed |
| 659 | -1 | | 5733.981 | | 1 | 21.58765 | | 235.7903 | | 40.03439 | | Patch 78 | | 340.1461 | | 6208.392 | | mixed patch w/ 1 6 in palet cinderblocks  2 8 in palet cinderblocks  2 10x10 box culverts  3 pieces concrete highway divider  low profile chunks of concrete | | | cinderblocks, culverts, highway divider, concrete | | | cinderblocks, culverts, highway divider, concrete | | CB, Culvert, HD, Concrete | | | mid-high | | 9 | 4 | | 9.654165 | | Other / Mixed | | Other / Mixed |
| 660 | -1 | | 12282.87 | | 1 | 21.88788 | | 245.9476 | | 39.14325 | | Patch 79 | | 344.2203 | | 8327.054 | | mixed patch w/ 1 6 in palet cinderblocks  2 8 in palet cinderblocks  2 10x10 box culverts  3 pieces concrete highway divider  low profile chunks of concrete | | | cinderblocks, culverts, highway divider, concrete | | | cinderblocks, culverts, highway divider, concrete | | CB, Culvert, HD, Concrete | | | mid-high | | 9 | 4 | | 10.41596 | | Other / Mixed | | Other / Mixed |
| 661 | -1 | | 21168.45 | | 1 | 21.94457 | | 252.1414 | | 45.95539 | | Patch 79 | | 344.2203 | | 8327.054 | | mixed patch w/ 1 6 in palet cinderblocks  2 8 in palet cinderblocks  2 10x10 box culverts  3 pieces concrete highway divider  low profile chunks of concrete | | | cinderblocks, culverts, highway divider, concrete | | | cinderblocks, culverts, highway divider, concrete | | CB, Culvert, HD, Concrete | | | mid-high | | 9 | 4 | | 10.96027 | | Other / Mixed | | Other / Mixed |
| 662 | -1 | | 772.9607 | | 1 | 21.98919 | | 246.4399 | | 66.53142 | | Patch 79 | | 344.2203 | | 8327.054 | | mixed patch w/ 1 6 in palet cinderblocks  2 8 in palet cinderblocks  2 10x10 box culverts  3 pieces concrete highway divider  low profile chunks of concrete | | | cinderblocks, culverts, highway divider, concrete | | | cinderblocks, culverts, highway divider, concrete | | CB, Culvert, HD, Concrete | | | mid-high | | 9 | 4 | | 7.650228 | | Other / Mixed | | Other / Mixed |
| 663 | -1 | | 1178.327 | | 1 | 22.8086 | | 278.9605 | | 23.39042 | | Patch 85 | | 137.7693 | | 1059.623 | | mixed patch w/ 1 6 in palet cinderblocks  2 8 in palet cinderblocks  2 10x10 box culverts  low profile chunks of concrete  low profile chunks of concrete | | | cinderblocks, culverts, highway divider, concrete | | | cinderblocks, culverts, highway divider, concrete | | CB, Culvert, HD, Concrete | | | mid-high | | 9 | 4 | | 8.071851 | | Other / Mixed | | Other / Mixed |
| 664 | -1 | | 1147.384 | | 1 | 22.86992 | | 152.5454 | | 35.66297 | | Patch 100 | | 235.7576 | | 3644.533 | | mixed patch w/ 1 6 in palet cinderblocks, 2 8 in palet cinderblocks , 2 10x10 box culverts, 3 pieces concrete highway divider, low profile chunks of concrete | | | cinderblocks, culverts, highway divider, concrete | | | cinderblocks, culverts, highway divider, concrete | | CB, Culvert, HD, Concrete | | | mid-high | | 9 | 4 | | 8.04524 | | Other / Mixed | | Other / Mixed |
| 665 | -1 | | 2747.255 | | 1 | 23.04774 | | 135.1062 | | 2.360838 | | Patch 101 | | 299.1166 | | 4868.212 | | mixed patch w/ 1 6 in palet cinderblocks  2 8 in palet cinderblocks  3 pieces concrete highway divider  5 round culverts  low profile chunks of concrete | | | cinderblocks, culverts, highway divider, concrete | | | cinderblocks, culverts, highway divider, concrete | | CB, Culvert, HD, Concrete | | | mid-high | | 9 | 4 | | 8.918358 | | Other / Mixed | | Other / Mixed |
| 666 | -1 | | 725.6373 | | 36 | 20.95258 | | 744.8901 | | 14.00277 | | Patch 68 | | 311.2744 | | 4672.48 | | mixed patch w/ low profile chunks of concrete  1 6 in palet cinderblocks  2 8 in palet cinderblocks  2 10x10 box culverts  3 pieces concrete highway divider | | | cinderblocks, culverts, highway divider, concrete | | | cinderblocks, culverts, highway divider, concrete | | CB, Culvert, HD, Concrete | | | mid-high | | 9 | 4 | | 7.58705 | | Other / Mixed | | Other / Mixed |
| 667 | -1 | | 6039.16 | | 302 | 21.07491 | | 844.0295 | | 15.79128 | | Patch 69 | | 458.0803 | | 11033.35 | | mixed patch w/ 1 6 in palet cinderblocks  2 8 in palet cinderblocks  2 10x10 box culverts  3 pieces concrete highway divider  low profile chunks of concrete | | | cinderblocks, culverts, highway divider, concrete | | | cinderblocks, culverts, highway divider, concrete | | CB, Culvert, HD, Concrete | | | mid-high | | 9 | 4 | | 9.70602 | | Other / Mixed | | Other / Mixed |
| 668 | -1 | | 1844.922 | | 1 | 21.2263 | | 929.2702 | | 2.867856 | | Patch 70 | | 387.2696 | | 8213.416 | | mixed patch w/ 1 6 in palet cinderblocks  2 8 in palet cinderblocks  2 10x10 box culverts  3 pieces concrete highway divider  low profile chunks of concrete | | | cinderblocks, culverts, highway divider, concrete | | | cinderblocks, culverts, highway divider, concrete | | CB, Culvert, HD, Concrete | | | mid-high | | 9 | 4 | | 8.520192 | | Other / Mixed | | Other / Mixed |
| 669 | -1 | | 3233.576 | | 1 | 21.11264 | | 950.6193 | | 26.32044 | | Patch 70 | | 387.2696 | | 8213.416 | | mixed patch w/ 1 6 in palet cinderblocks  2 8 in palet cinderblocks  2 10x10 box culverts  3 pieces concrete highway divider  low profile chunks of concrete | | | cinderblocks, culverts, highway divider, concrete | | | cinderblocks, culverts, highway divider, concrete | | CB, Culvert, HD, Concrete | | | mid-high | | 9 | 4 | | 9.081344 | | Other / Mixed | | Other / Mixed |
| 670 | -1 | | 13248.07 | | 662 | 21.16244 | | 1042.919 | | 12.37649 | | Patch 71 | | 285.8251 | | 5126.988 | | mixed patch w/ 2 8 in palet cinderblocks  2 10x10 box culverts  3 pieces concrete highway divider  3 pieces concrete highway divider  low profile chunks of concrete | | | cinderblocks, culverts, highway divider, concrete | | | cinderblocks, culverts, highway divider, concrete | | CB, Culvert, HD, Concrete | | | mid-high | | 9 | 4 | | 10.49161 | | Other / Mixed | | Other / Mixed |
| 671 | -1 | | 159.7236 | | 8 | 21.84063 | | 257.3908 | | 13.36793 | | Patch 77 | | 311.3754 | | 5600.178 | | mixed patch w/ 1 6 in palet cinderblocks  2 8 in palet cinderblocks  2 10x10 box culverts  3 pieces concrete highway divider  low profile chunks of concrete | | | cinderblocks, culverts, highway divider, concrete | | | cinderblocks, culverts, highway divider, concrete | | CB, Culvert, HD, Concrete | | | mid-high | | 9 | 4 | | 6.073445 | | Other / Mixed | | Other / Mixed |
| 672 | -1 | | 843.3826 | | 1 | 21.7368 | | 278.2294 | | 43.00179 | | Patch 78 | | 340.1461 | | 6208.392 | | mixed patch w/ 1 6 in palet cinderblocks  2 8 in palet cinderblocks  2 10x10 box culverts  3 pieces concrete highway divider  low profile chunks of concrete | | | cinderblocks, culverts, highway divider, concrete | | | cinderblocks, culverts, highway divider, concrete | | CB, Culvert, HD, Concrete | | | mid-high | | 9 | 4 | | 7.737421 | | Other / Mixed | | Other / Mixed |
| 673 | -1 | | 1717.532 | | 1 | 21.59909 | | 285.5101 | | 29.78617 | | Patch 78 | | 340.1461 | | 6208.392 | | mixed patch w/ 1 6 in palet cinderblocks  2 8 in palet cinderblocks  2 10x10 box culverts  3 pieces concrete highway divider  low profile chunks of concrete | | | cinderblocks, culverts, highway divider, concrete | | | cinderblocks, culverts, highway divider, concrete | | CB, Culvert, HD, Concrete | | | mid-high | | 9 | 4 | | 8.448644 | | Other / Mixed | | Other / Mixed |
| 674 | -1 | | 1821.325 | | 1 | 22.42944 | | 286.7401 | | 33.18715 | | Patch 82 | | 281.5516 | | 4100.548 | | mixed patch w/ 1 6 in palet cinderblocks  2 8 in palet cinderblocks  2 10x10 box culverts  3 pieces concrete highway divider  3 pieces concrete highway divider  low profile chunks of concrete | | | cinderblocks, culverts, highway divider, concrete | | | cinderblocks, culverts, highway divider, concrete | | CB, Culvert, HD, Concrete | | | mid-high | | 9 | 4 | | 8.50732 | | Other / Mixed | | Other / Mixed |
| 675 | -1 | | 1751.814 | | 1 | 22.58875 | | 258.0652 | | 36.01939 | | Patch 84 | | 302.2478 | | 5295.404 | | mixed patch w/ 1 6 in palet cinderblocks  2 8 in palet cinderblocks  2 10x10 box culverts  3 pieces concrete highway divider  low profile chunks of concrete | | | cinderblocks, culverts, highway divider, concrete | | | cinderblocks, culverts, highway divider, concrete | | CB, Culvert, HD, Concrete | | | mid-high | | 9 | 4 | | 8.468407 | | Other / Mixed | | Other / Mixed |
| 676 | -1 | | 3639.405 | | 1 | 22.69993 | | 260.8672 | | 51.55003 | | Patch 84 | | 302.2478 | | 5295.404 | | mixed patch w/ 1 6 in palet cinderblocks  2 8 in palet cinderblocks  2 10x10 box culverts  3 pieces concrete highway divider  low profile chunks of concrete | | | cinderblocks, culverts, highway divider, concrete | | | cinderblocks, culverts, highway divider, concrete | | CB, Culvert, HD, Concrete | | | mid-high | | 9 | 4 | | 9.199575 | | Other / Mixed | | Other / Mixed |
| 677 | -1 | | 6481.578 | | 1 | 22.50145 | | 252.739 | | 37.45691 | | Patch 84 | | 302.2478 | | 5295.404 | | mixed patch w/ 1 6 in palet cinderblocks  2 8 in palet cinderblocks  2 10x10 box culverts  3 pieces concrete highway divider  low profile chunks of concrete | | | cinderblocks, culverts, highway divider, concrete | | | cinderblocks, culverts, highway divider, concrete | | CB, Culvert, HD, Concrete | | | mid-high | | 9 | 4 | | 9.776719 | | Other / Mixed | | Other / Mixed |
| 678 | -1 | | 3895.066 | | 1 | 22.91829 | | 262.7158 | | 8.128097 | | Patch 85 | | 137.7693 | | 1059.623 | | mixed patch w/ 1 6 in palet cinderblocks  2 8 in palet cinderblocks  2 10x10 box culverts  low profile chunks of concrete  low profile chunks of concrete | | | cinderblocks, culverts, highway divider, concrete | | | cinderblocks, culverts, highway divider, concrete | | CB, Culvert, HD, Concrete | | | mid-high | | 9 | 4 | | 9.267466 | | Other / Mixed | | Other / Mixed |
| 679 | -1 | | 1441.555 | | 1 | 23.00038 | | 282.3896 | | 3.658197 | | Patch 86 | | 317.8525 | | 4563.282 | | mixed patch w/ 1 6 in palet cinderblocks  2 8 in palet cinderblocks  2 10x10 box culverts  3 pieces concrete highway divider  low profile chunks of concrete  low profile chunks of concrete | | | cinderblocks, culverts, highway divider, concrete | | | cinderblocks, culverts, highway divider, concrete | | CB, Culvert, HD, Concrete | | | mid-high | | 9 | 4 | | 8.273478 | | Other / Mixed | | Other / Mixed |
| 680 | -1 | | 129.2517 | | 6 | 23.30393 | | 192.4586 | | 7.945759 | | Patch 95 | | 194.3622 | | 2266.444 | | mixed patch w/ 1 6 in palet cinderblocks  2 8 in palet cinderblocks  2 10x10 box culverts  3 pieces concrete highway divider  low profile chunks of concrete | | | cinderblocks, culverts, highway divider, concrete | | | cinderblocks, culverts, highway divider, concrete | | CB, Culvert, HD, Concrete | | | mid-high | | 9 | 4 | | 5.861762 | | Other / Mixed | | Other / Mixed |
| 681 | -1 | | 6208.564 | | 1 | 23.19066 | | 151.7828 | | 11.86483 | | Patch 98 | | 175.0049 | | 1736.466 | | mixed patch w/ 1 6 in palet cinderblocks   2 8 in palet cinderblocks   2 10x10 box culverts  3 pieces concrete highway divider  low profile chunks of concrete | | | cinderblocks, culverts, highway divider, concrete | | | cinderblocks, culverts, highway divider, concrete | | CB, Culvert, HD, Concrete | | | mid-high | | 9 | 4 | | 9.733685 | | Other / Mixed | | Other / Mixed |
| 682 | -1 | | 5003.355 | | 1 | 21.55929 | | 281.2417 | | 39.64203 | | Patch 77 | | 311.3754 | | 5600.178 | | mixed patch w/ 1 6 in palet cinderblocks  2 8 in palet cinderblocks  2 10x10 box culverts  3 pieces concrete highway divider  low profile chunks of concrete | | | cinderblocks, culverts, highway divider, concrete | | | cinderblocks, culverts, highway divider, concrete | | CB, Culvert, HD, Concrete | | | mid-high | | 9 | 4 | | 9.517864 | | Other / Mixed | | Other / Mixed |
| 683 | -1 | | 1401.188 | | 1 | 20.53563 | | 1026.151 | | 98.72318 | | Patch 58 | | 357.3882 | | 8363.916 | | mixed patch w/ 1 8 in palet cinderblocks  1 8 in palet cinderblocks  4 pyramids | | | cinderblocks, pyramids | | | cinderblocks, pyramids | | CB, PY | | | mid | | 9 | 2 | | 8.245076 | | Pyramids | | Other / Mixed |
| 684 | -1 | | 152.5882 | | 1 | 21.00561 | | 995.5168 | | 83.6386 | | Patch 60 | | 584.4798 | | 20123.45 | | mixed patch w/ 1 8 in palet cinderblocks  1 8 in palet cinderblocks  4 pyramids | | | cinderblocks, pyramids | | | cinderblocks, pyramids | | CB, PY | | | mid | | 9 | 2 | | 6.027743 | | Pyramids | | Other / Mixed |
| 685 | -1 | | 573.2563 | | 1 | 21.18816 | | 919.9506 | | 23.69886 | | Patch 60 | | 584.4798 | | 20123.45 | | mixed patch w/ 1 8 in palet cinderblocks  1 8 in palet cinderblocks  4 pyramids | | | cinderblocks, pyramids | | | cinderblocks, pyramids | | CB, PY | | | mid | | 9 | 2 | | 7.351333 | | Pyramids | | Other / Mixed |
| 686 | -1 | | 2025.091 | | 1 | 20.62782 | | 209.8518 | | 53.56811 | | Patch 52 | | 561.5502 | | 18916.29 | | mixed patch w/ 1 8 in palet cinderblocks  1 8 in palet cinderblocks  4 pyramids | | | cinderblocks, pyramids | | | cinderblocks, pyramids | | CB, PY | | | mid | | 9 | 2 | | 8.61337 | | Pyramids | | Other / Mixed |
| 687 | -1 | | 360.9231 | | 1 | 20.51242 | | 338.7475 | | 116.732 | | Patch 52 | | 561.5502 | | 18916.29 | | mixed patch w/ 1 8 in palet cinderblocks  1 8 in palet cinderblocks  4 pyramids | | | cinderblocks, pyramids | | | cinderblocks, pyramids | | CB, PY | | | mid | | 9 | 2 | | 6.888665 | | Pyramids | | Other / Mixed |
| 688 | -1 | | 534.5908 | | 27 | 20.64081 | | 908.4778 | | 111.2439 | | Patch 58 | | 357.3882 | | 8363.916 | | mixed patch w/ 1 8 in palet cinderblocks  1 8 in palet cinderblocks  4 pyramids | | | cinderblocks, pyramids | | | cinderblocks, pyramids | | CB, PY | | | mid | | 9 | 2 | | 7.281502 | | Pyramids | | Other / Mixed |
| 689 | -1 | | 57858.09 | | 2893 | 20.51341 | | 819.9605 | | 64.74595 | | Patch 57 | | 242.1795 | | 3523.531 | | cinderblocks, pyramids | | | cinderblocks, pyramids | | | cinderblocks, pyramids | | CB, PY | | | mid | | 9 | 2 | | 11.96575 | | Pyramids | | Other / Mixed |
| 690 | -1 | | 880.4844 | | 1 | 20.75063 | | 802.9155 | | 11.46672 | | Patch 57 | | 242.1795 | | 3523.531 | | cinderblocks, pyramids | | | cinderblocks, pyramids | | | cinderblocks, pyramids | | CB, PY | | | mid | | 9 | 2 | | 7.780472 | | Pyramids | | Other / Mixed |
| 691 | -1 | | 1197.801 | | 1 | 20.54983 | | 903.0565 | | 50.6123 | | Patch 59 | | 447.1259 | | 11696.09 | | cinderblocks, pyramids | | | cinderblocks, pyramids | | | cinderblocks, pyramids | | CB, PY | | | mid | | 9 | 2 | | 8.088243 | | Pyramids | | Other / Mixed |
| 692 | -1 | | 1451.608 | | 1 | 20.59144 | | 949.7072 | | 37.39967 | | Patch 59 | | 447.1259 | | 11696.09 | | cinderblocks, pyramids | | | cinderblocks, pyramids | | | cinderblocks, pyramids | | CB, PY | | | mid | | 9 | 2 | | 8.280427 | | Pyramids | | Other / Mixed |
| 693 | -1 | | 20.18186 | | 1 | 20.48423 | | 890.6203 | | 75.0641 | | Patch 59 | | 447.1259 | | 11696.09 | | cinderblocks, pyramids | | | cinderblocks, pyramids | | | cinderblocks, pyramids | | CB, PY | | | mid | | 9 | 2 | | 4.004784 | | Pyramids | | Other / Mixed |
| 694 | -1 | | 264.071 | | 1 | 20.67431 | | 269.5763 | | 37.94786 | | Patch 52 | | 561.5502 | | 18916.29 | | mixed patch w/ 1 8 in palet cinderblocks  1 8 in palet cinderblocks  4 pyramids | | | cinderblocks, pyramids | | | cinderblocks, pyramids | | CB, PY | | | mid | | 9 | 2 | | 6.576218 | | Pyramids | | Pyramids |
| 695 | -1 | | 342.9071 | | 1 | 20.51342 | | 293.8935 | | 31.15145 | | Patch 52 | | 561.5502 | | 18916.29 | | mixed patch w/ 1 8 in palet cinderblocks  1 8 in palet cinderblocks  4 pyramids | | | cinderblocks, pyramids | | | cinderblocks, pyramids | | CB, PY | | | mid | | 9 | 2 | | 6.837459 | | Pyramids | | Pyramids |
| 696 | -1 | | 173.3835 | | 1 | 20.62389 | | 272.8569 | | 18.75251 | | Patch 52 | | 561.5502 | | 18916.29 | | mixed patch w/ 1 8 in palet cinderblocks  1 8 in palet cinderblocks  4 pyramids | | | cinderblocks, pyramids | | | cinderblocks, pyramids | | CB, PY | | | mid | | 9 | 2 | | 6.155506 | | Pyramids | | Pyramids |
| 697 | -1 | | 5108.252 | | 1 | 20.70253 | | 231.0452 | | 44.94545 | | Patch 52 | | 561.5502 | | 18916.29 | | mixed patch w/ 1 8 in palet cinderblocks  1 8 in palet cinderblocks  4 pyramids | | | cinderblocks, pyramids | | | cinderblocks, pyramids | | CB, PY | | | mid | | 9 | 2 | | 9.538613 | | Pyramids | | Pyramids |
| 698 | -1 | | 5876.302 | | 1 | 20.73048 | | 241.5047 | | 43.43183 | | Patch 52 | | 561.5502 | | 18916.29 | | mixed patch w/ 1 8 in palet cinderblocks  1 8 in palet cinderblocks  4 pyramids | | | cinderblocks, pyramids | | | cinderblocks, pyramids | | CB, PY | | | mid | | 9 | 2 | | 9.678683 | | Pyramids | | Pyramids |
| 699 | -1 | | 594.4951 | | 1 | 20.67905 | | 276.2433 | | 16.42548 | | Patch 52 | | 561.5502 | | 18916.29 | | mixed patch w/ 1 8 in palet cinderblocks  1 8 in palet cinderblocks  4 pyramids | | | cinderblocks, pyramids | | | cinderblocks, pyramids | | CB, PY | | | mid | | 9 | 2 | | 7.387713 | | Pyramids | | Pyramids |
| 700 | -1 | | 1406.013 | | 1 | 20.70091 | | 272.4896 | | 63.94539 | | Patch 52 | | 561.5502 | | 18916.29 | | mixed patch w/ 1 8 in palet cinderblocks  1 8 in palet cinderblocks  4 pyramids | | | cinderblocks, pyramids | | | cinderblocks, pyramids | | CB, PY | | | mid | | 9 | 2 | | 8.248513 | | Pyramids | | Pyramids |
| 701 | -1 | | 2229.991 | | 1 | 20.6324 | | 276.4736 | | 27.97293 | | Patch 52 | | 561.5502 | | 18916.29 | | mixed patch w/ 1 8 in palet cinderblocks  1 8 in palet cinderblocks  4 pyramids | | | cinderblocks, pyramids | | | cinderblocks, pyramids | | CB, PY | | | mid | | 9 | 2 | | 8.709753 | | Pyramids | | Pyramids |
| 702 | -1 | | 579.2797 | | 1 | 21.10083 | | 594.8597 | | 20.51412 | | Patch 56 | | 310.139 | | 5748.809 | | mixed patch w/ 1 8 in palet cinderblocks  1 8 in palet cinderblocks  4 pyramids | | | cinderblocks, pyramids | | | cinderblocks, pyramids | | CB, PY | | | mid | | 9 | 2 | | 7.361786 | | Pyramids | | Pyramids |
| 703 | -1 | | 1292.479 | | 1 | 21.169 | | 608.419 | | 6.358658 | | Patch 56 | | 310.139 | | 5748.809 | | mixed patch w/ 1 8 in palet cinderblocks  1 8 in palet cinderblocks  4 pyramids | | | cinderblocks, pyramids | | | cinderblocks, pyramids | | CB, PY | | | mid | | 9 | 2 | | 8.164317 | | Pyramids | | Pyramids |
| 704 | -1 | | 1053.437 | | 1 | 21.02291 | | 1001.104 | | 12.45603 | | Patch 58 | | 357.3882 | | 8363.916 | | mixed patch w/ 1 8 in palet cinderblocks  1 8 in palet cinderblocks  4 pyramids | | | cinderblocks, pyramids | | | cinderblocks, pyramids | | CB, PY | | | mid | | 9 | 2 | | 7.959813 | | Pyramids | | Pyramids |
| 705 | -1 | | 135.4459 | | 7 | 20.93554 | | 868.2508 | | 49.55817 | | Patch 60 | | 584.4798 | | 20123.45 | | mixed patch w/ 1 8 in palet cinderblocks  1 8 in palet cinderblocks  4 pyramids | | | cinderblocks, pyramids | | | cinderblocks, pyramids | | CB, PY | | | mid | | 9 | 2 | | 5.908573 | | Pyramids | | Pyramids |
| 706 | -1 | | 4386.079 | | 1 | 23.19716 | | 124.3588 | | 18.98845 | | Patch 88 | | 84.63844 | | 476.493 | | mixed patch w/ 2 8 in palet cinderblocks  low profile chunks of concrete  concrete watermill structure | | | cinderblocks, water mill, concrete | | | cinderblocks, water mill, concrete | | CB, WM, Concrete | | | mid-high | | 9 | 3 | | 9.386191 | | Other / Mixed | | Pyramids |
| 707 | -1 | | 1341.229 | | 1 | 23.32982 | | 129.5565 | | 3.958257 | | Patch 88 | | 84.63844 | | 476.493 | | mixed patch w/ 2 8 in palet cinderblocks  low profile chunks of concrete  concrete watermill structure | | | cinderblocks, water mill, concrete | | | cinderblocks, water mill, concrete | | CB, WM, Concrete | | | mid-high | | 9 | 3 | | 8.201342 | | Other / Mixed | | Pyramids |
| 708 | -1 | | 1839.909 | | 1 | 23.18733 | | 82.34703 | | 69.83077 | | Patch 106 | | 177.4416 | | 1670.948 | | mixed patch w/ 2 8 in palet cinderblocks  2 8 in palet cinderblocks  low profile chunks of concrete  concrete watermill structure | | | cinderblocks, water mill, highway divider, concrete | | | cinderblocks, water mill, highway divider, concrete | | CB, WM, HD, Concrete | | | mid-high | | 9 | 4 | | 8.517471 | | Other / Mixed | | Pyramids |
| 709 | -1 | | 26.70913 | | 1 | 20.67851 | | 183.1084 | | 30.9425 | | Patch 64 | | 284.3085 | | 4381.324 | | mixed patch w/ 2 8 in palet cinderblocks  3 pieces concrete highway divider  low profile chunks of concrete  concrete watermill structure  concrete watermill structure | | | cinderblocks, water mill, highway divider, concrete | | | cinderblocks, water mill, highway divider, concrete | | CB, WM, HD, Concrete | | | mid-high | | 9 | 4 | | 4.285005 | | Other / Mixed | | Pyramids |
| 710 | -1 | | 34.83888 | | 2 | 20.6707 | | 107.8483 | | 91.42116 | | Patch 64 | | 284.3085 | | 4381.324 | | mixed patch w/ 2 8 in palet cinderblocks  3 pieces concrete highway divider  low profile chunks of concrete  concrete watermill structure  concrete watermill structure | | | cinderblocks, water mill, highway divider, concrete | | | cinderblocks, water mill, highway divider, concrete | | CB, WM, HD, Concrete | | | mid-high | | 9 | 4 | | 4.550734 | | Other / Mixed | | Pyramids |
| 711 | -1 | | 83.29957 | | 4 | 20.59369 | | 235.3769 | | 97.83144 | | Patch 64 | | 284.3085 | | 4381.324 | | mixed patch w/ 2 8 in palet cinderblocks  3 pieces concrete highway divider  low profile chunks of concrete  concrete watermill structure  concrete watermill structure | | | cinderblocks, water mill, highway divider, concrete | | | cinderblocks, water mill, highway divider, concrete | | CB, WM, HD, Concrete | | | mid-high | | 9 | 4 | | 5.422443 | | Other / Mixed | | Pyramids |
| 712 | -1 | | 9768.565 | | 488 | 22.97458 | | 142.1279 | | 38.20035 | | Patch 106 | | 177.4416 | | 1670.948 | | mixed patch w/ 2 8 in palet cinderblocks  2 8 in palet cinderblocks  low profile chunks of concrete  concrete watermill structure | | | cinderblocks, water mill, highway divider, concrete | | | cinderblocks, water mill, highway divider, concrete | | CB, WM, HD, Concrete | | | mid-high | | 9 | 4 | | 10.18692 | | Other / Mixed | | Pyramids |
| 713 | -1 | | 788.3736 | | 39 | 20.64495 | | 170.0962 | | 29.18865 | | Patch 64 | | 284.3085 | | 4381.324 | | mixed patch w/ 2 8 in palet cinderblocks  3 pieces concrete highway divider  low profile chunks of concrete  concrete watermill structure  concrete watermill structure | | | cinderblocks, water mill, highway divider, concrete | | | cinderblocks, water mill, highway divider, concrete | | CB, WM, HD, Concrete | | | mid-high | | 9 | 4 | | 7.669972 | | Other / Mixed | | Pyramids |
| 714 | -1 | | 3517.315 | | 176 | 20.66216 | | 182.9508 | | 16.65691 | | Patch 64 | | 284.3085 | | 4381.324 | | mixed patch w/ 2 8 in palet cinderblocks  3 pieces concrete highway divider  low profile chunks of concrete  concrete watermill structure  concrete watermill structure | | | cinderblocks, water mill, highway divider, concrete | | | cinderblocks, water mill, highway divider, concrete | | CB, WM, HD, Concrete | | | mid-high | | 9 | 4 | | 9.165453 | | Other / Mixed | | Pyramids |
| 715 | -1 | | 1679.617 | | 1 | 22.97884 | | 249.3217 | | 24.74077 | | CCA Corner | | 1377.797 | | 60636 | | 100 tons rr ties in line | | | CCA rr tie ridge | | | CCA rr tie ridge | | CCA Ridge | | | mid-high | | 6 | 1 | | 8.426321 | | RR Ties | | Pyramids |
| 716 | -1 | | 5584.391 | | 1 | 22.90131 | | 273.3181 | | 19.23125 | | CCA Corner | | 1377.797 | | 60636 | | 100 tons rr ties in line | | | CCA rr tie ridge | | | CCA rr tie ridge | | CCA Ridge | | | mid-high | | 6 | 1 | | 9.627731 | | RR Ties | | Pyramids |
| 717 | -1 | | 136.3775 | | 1 | 22.55706 | | 636.0535 | | 43.37583 | | tile 200 ton1 | | 298.0293 | | 5012.627 | | concrete tiles | | | concrete | | | concrete | | Concrete | | | low | | 9 | 1 | | 5.915427 | | Concrete | | Pyramids |
| 718 | -1 | | 2471.173 | | 1 | 22.55356 | | 668.2273 | | 18.79581 | | tile 200 ton1 | | 298.0293 | | 5012.627 | | concrete tiles | | | concrete | | | concrete | | Concrete | | | low | | 9 | 1 | | 8.812448 | | Concrete | | Pyramids |
| 719 | -1 | | 497.703 | | 1 | 21.1754 | | 1020.43 | | 29.24664 | | Extra rr ties 2 | | 381.6534 | | 5889.56 | | RR ties | | | rr ties | | | RR | | Extra RR | | | mid | | 6 | 1 | | 7.210004 | | RR Ties | | Pyramids |
| 720 | -1 | | 1323.202 | | 1 | 21.28253 | | 310.016 | | 40.64092 | | Extra rr ties 1 | | 135.3473 | | 811.1455 | | RR ties | | | rr ties | | | RR | | Extra RR | | | mid | | 6 | 1 | | 8.187809 | | RR Ties | | Pyramids |
| 721 | -1 | | 1288.349 | | 1 | 21.15919 | | 261.3502 | | 15.97445 | | Extra rr ties 1 | | 135.3473 | | 811.1455 | | RR ties | | | rr ties | | | RR | | Extra RR | | | mid | | 6 | 1 | | 8.161117 | | RR Ties | | Pyramids |
| 722 | -1 | | 13303.1 | | 1 | 21.59805 | | 79.73997 | | 46.65301 | | Hwy Dividers7 | | 94.88432 | | 318.1473 | | 3 pieces concrete highway divider, 60 tons rr ties | | | highway divider, rr ties | | | highway divider, rr ties | | HD, RR | | | mid | | 9 | 2 | | 10.49575 | | RR Ties | | Pyramids |
| 723 | -1 | | 1793.459 | | 1 | 21.84174 | | 103.7124 | | 21.03115 | | Hwy Dividers30 | | 187.8667 | | 1699.327 | | 3 pieces concrete highway divider, 60 tons rr ties | | | highway divider, rr ties | | | highway divider, rr ties | | HD, RR | | | mid | | 9 | 2 | | 8.491901 | | RR Ties | | Pyramids |
| 724 | -1 | | 203.5617 | | 1 | 21.78244 | | 111.6068 | | 36.14513 | | Hwy Dividers30 | | 187.8667 | | 1699.327 | | 3 pieces concrete highway divider, 60 tons rr ties | | | highway divider, rr ties | | | highway divider, rr ties | | HD, RR | | | mid | | 9 | 2 | | 6.315969 | | RR Ties | | Pyramids |
| 725 | -1 | | 3451.655 | | 1 | 21.69428 | | 120.0285 | | 19.63028 | | Hwy Dividers31 | | 115.1263 | | 771.9108 | | 3 pieces concrete highway divider, 60 tons rr ties | | | highway divider, rr ties | | | highway divider, rr ties | | HD, RR | | | mid | | 9 | 2 | | 9.146609 | | RR Ties | | Pyramids |
| 726 | -1 | | 20675.73 | | 1 | 22.13685 | | 166.1885 | | 71.8182 | | Hwy Dividers33 | | 148.5237 | | 726.2203 | | 3 pieces concrete highway divider, 60 tons rr ties | | | highway divider, rr ties | | | highway divider, rr ties | | HD, RR | | | mid | | 9 | 2 | | 10.93672 | | RR Ties | | Pyramids |
| 727 | -1 | | 409.4351 | | 1 | 22.28638 | | 111.7845 | | 41.79673 | | Hwy Dividers33 | | 148.5237 | | 726.2203 | | 3 pieces concrete highway divider, 60 tons rr ties | | | highway divider, rr ties | | | highway divider, rr ties | | HD, RR | | | mid | | 9 | 2 | | 7.014778 | | RR Ties | | Pyramids |
| 728 | -1 | | 331.371 | | 1 | 22.17856 | | 101.9568 | | 56.97553 | | Hwy Dividers33 | | 148.5237 | | 726.2203 | | 3 pieces concrete highway divider, 60 tons rr ties | | | highway divider, rr ties | | | highway divider, rr ties | | HD, RR | | | mid | | 9 | 2 | | 6.803239 | | RR Ties | | Pyramids |
| 729 | -1 | | 946.2371 | | 1 | 21.8043 | | 80.40285 | | 43.32514 | | Hwy Dividers31 | | 115.1263 | | 771.9108 | | 3 pieces concrete highway divider, 60 tons rr ties | | | highway divider, rr ties | | | highway divider, rr ties | | HD, RR | | | mid | | 9 | 2 | | 7.852493 | | RR Ties | | Pyramids |
| 730 | -1 | | 758.2199 | | 1 | 21.60895 | | 77.82127 | | 31.98233 | | Hwy Dividers7 | | 94.88432 | | 318.1473 | | 3 pieces concrete highway divider, 60 tons rr ties | | | highway divider, rr ties | | | highway divider, rr ties | | HD, RR | | | mid | | 9 | 2 | | 7.630973 | | RR Ties | | Other / Mixed |
| 731 | -1 | | 778.8349 | | 1 | 21.58521 | | 85.87217 | | 45.68221 | | Hwy Dividers 4 and 8 | | 265.8981 | | 3820.08 | | 6 pieces concrete highway divider, 60 tons rr ties | | | highway divider, rr ties | | | highway divider, rr ties | | HD, RR | | | mid | | 9 | 2 | | 7.657799 | | RR Ties | | Other / Mixed |
| 732 | -1 | | 8447.661 | | 1 | 21.58785 | | 93.32896 | | 25.33237 | | Hwy Dividers9 | | 83.73836 | | 296.5562 | | 3 pieces concrete highway divider, 60 tons rr ties | | | highway divider, rr ties | | | highway divider, rr ties | | HD, RR | | | mid | | 9 | 2 | | 10.04164 | | RR Ties | | Other / Mixed |
| 733 | -1 | | 487.0342 | | 1 | 22.48833 | | 118.6948 | | 48.93641 | | Hwy Dividers 34 and 35 | | 232.4753 | | 2215.741 | | 6 pieces concrete highway divider, 60 tons rr ties | | | highway divider, rr ties | | | highway divider, rr ties | | HD, RR | | | mid | | 9 | 2 | | 7.188334 | | RR Ties | | Other / Mixed |
| 734 | -1 | | 2871.458 | | 1 | 21.67168 | | 112.987 | | 6.395394 | | Hwy Dividers 4 and 8 | | 265.8981 | | 3820.08 | | 6 pieces concrete highway divider, 60 tons rr ties | | | highway divider, rr ties | | | highway divider, rr ties | | HD, RR | | | mid | | 9 | 2 | | 8.962575 | | RR Ties | | Other / Mixed |
| 735 | -1 | | 912.8642 | | 1 | 20.85618 | | 96.70916 | | 17.4946 | | Hwy Dividers6 | | 111.8173 | | 439.9159 | | 3 pieces concrete highway divider, 60 tons rr ties | | | highway divider, rr ties | | | highway divider, rr ties | | HD, RR | | | mid | | 9 | 2 | | 7.816587 | | RR Ties | | Other / Mixed |
| 736 | -1 | | 1608.262 | | 1 | 22.73782 | | 145.1542 | | 34.59908 | | Hwy Dividers12 | | 135.0356 | | 980.845 | | 3 pieces concrete highway divider, 60 tons rr ties, 10 tons concrete | | | highway divider, rr ties, concrete | | | highway divider, rr ties, concrete | | HD, RR, Concrete | | | mid | | 9 | 3 | | 8.38291 | | RR Ties | | Other / Mixed |
| 737 | -1 | | 343.1634 | | 1 | 21.59835 | | 588.8728 | | 19.84576 | | LS15 | | 29.67278 | | 59.58879 | | One Gulf LP | | | limestone | | | limestone | | Limestone | | | low | | 8 | 1 | | 6.838207 | | Concrete | | Other / Mixed |
| 738 | -1 | | 899.3828 | | 1 | 21.33055 | | 451.457 | | 8.158099 | | LS2 | | 21.2308 | | 30.91988 | | One Gulf LP | | | limestone | | | limestone | | Limestone | | | low | | 8 | 1 | | 7.801709 | | Concrete | | Other / Mixed |
| 739 | -1 | | 30.85158 | | 1 | 21.29607 | | 602.5153 | | 47.62562 | | LPM2 | | 29.8093 | | 55.19877 | | One Gulf LP | | | low profile | | | low profile | | Low Profile | | | low | | 8 | 1 | | 4.429188 | | Low Profile | | Other / Mixed |
| 740 | -1 | | 64.38273 | | 1 | 21.28193 | | 598.8201 | | 70.48353 | | LPM2 | | 29.8093 | | 55.19877 | | One Gulf LP | | | low profile | | | low profile | | Low Profile | | | low | | 8 | 1 | | 5.164845 | | Low Profile | | Other / Mixed |
| 741 | -1 | | 198.3266 | | 1 | 21.27729 | | 580.6095 | | 22.58472 | | LPM2 | | 29.8093 | | 55.19877 | | One Gulf LP | | | low profile | | | low profile | | Low Profile | | | low | | 8 | 1 | | 6.289915 | | Low Profile | | Other / Mixed |
| 742 | -1 | | 505.9585 | | 1 | 21.98851 | | 508.0737 | | 160.6158 | | Octoreef5 | | 53.09517 | | 159.8517 | | octoreef | | | octoreef | | | octoreef | | Octoreef | | | mid | | 9 | 1 | | 7.226455 | | Pyramids | | Other / Mixed |
| 743 | -1 | | 2007.563 | | 1 | 21.52553 | | 266.3835 | | 21.9134 | | Octoreef1 | | 41.39858 | | 98.39998 | | octoreef | | | octoreef | | | octoreef | | Octoreef | | | mid | | 9 | 1 | | 8.604677 | | Pyramids | | Other / Mixed |
| 744 | -1 | | 3596.618 | | 1 | 20.98253 | | 703.4837 | | 6.994543 | | Octoreef2 | | 27.38307 | | 47.99176 | | octoreef | | | octoreef | | | octoreef | | Octoreef | | | mid | | 9 | 1 | | 9.187749 | | Pyramids | | Other / Mixed |
| 745 | -1 | | 1138.747 | | 1 | 22.30993 | | 746.9371 | | 63.67357 | | rr tie ridge | | 2402.195 | | 49406.01 | | rr tie ridge | | | rr tie ridge | | | rr tie ridge | | RR Ridge | | | mid | | 8 | 1 | | 8.037684 | | RR Ties | | Other / Mixed |
| 746 | -1 | | 4169.226 | | 1 | 22.56816 | | 707.8141 | | 64.67481 | | rr tie ridge | | 2402.195 | | 49406.01 | | rr tie ridge | | | rr tie ridge | | | rr tie ridge | | RR Ridge | | | mid | | 8 | 1 | | 9.335486 | | RR Ties | | Other / Mixed |
| 747 | -1 | | 1359.012 | | 1 | 23.09453 | | 169.9363 | | 53.38785 | | D-17 | | 522.3085 | | 14796.04 | | Concrete Railroad Ties | | | rr ties | | | RR Ties | | RR Ties | | | mid | | 5 | 1 | | 8.214513 | | RR Ties | | Other / Mixed |
| 748 | -1 | | 2932.429 | | 1 | 20.89019 | | 831.6492 | | 87.9733 | | 100tonmixed5 | | 293.9717 | | 4327.636 | | 75 ton ties, 25 tons broken concrete | | | rr ties, concrete | | | RR, Concrete | | RR, Concrete | | | mid | | 6 | 2 | | 8.983586 | | RR Ties | | Other / Mixed |
| 749 | -1 | | 279.0213 | | 1 | 21.3541 | | 728.4178 | | 39.4676 | | 100tonmixed7 | | 242.3415 | | 3558.944 | | 75 ton ties, 25 tons broken concrete | | | rr ties, concrete | | | RR, Concrete | | RR, Concrete | | | mid | | 6 | 2 | | 6.631288 | | RR Ties | | Other / Mixed |
| 750 | -1 | | 7403.625 | | 370 | 21.61917 | | 812.3124 | | 30.55388 | | 100tonmixed12 | | 286.2058 | | 4736.871 | | 75 ton ties, 25 tons broken concrete | | | rr ties, concrete | | | RR, Concrete | | RR, Concrete | | | mid | | 6 | 2 | | 9.909725 | | RR Ties | | Other / Mixed |
| 751 | -1 | | 2857.363 | | 143 | 21.6306 | | 535.1956 | | 7.163609 | | 100tonmixed17 | | 317.462 | | 4789.935 | | 75 ton ties, 25 tons broken concrete | | | rr ties, concrete | | | RR, Concrete | | RR, Concrete | | | mid | | 6 | 2 | | 8.957654 | | RR Ties | | Other / Mixed |
| 752 | -1 | | 908.1098 | | 1 | 20.73302 | | 257.1218 | | 31.82362 | | Mix_1 | | 177.5809 | | 1787.628 | | 25 ton RR Ties, 25 ton Concrete, 6 Pallets CinderBlock | | | rr ties, concrete, cinderblocks | | | RR, Concrete, CB | | RR, Concrete, CB | | | mid | | 6 | 3 | | 7.811365 | | RR Ties | | Other / Mixed |
| 753 | -1 | | 112.4578 | | 1 | 21.79752 | | 272.6759 | | 30.46012 | | Mix_7 | | 352.5258 | | 4514.797 | | 25 ton RR Ties, 25 ton Concrete, 6 Pallets CinderBlock | | | rr ties, concrete, cinderblocks | | | RR, Concrete, CB | | RR, Concrete, CB | | | mid | | 6 | 3 | | 5.722578 | | RR Ties | | Concrete |
| 754 | -1 | | 2801.918 | | 1 | 23.02975 | | 216.2857 | | 12.22058 | | Mix_44 | | 330.685 | | 5537.713 | | 25 ton RR Ties, 25 ton Concrete, 6 Pallets CinderBlock | | | rr ties, concrete, cinderblocks | | | RR, Concrete, CB | | RR, Concrete, CB | | | mid | | 6 | 3 | | 8.938059 | | RR Ties | | Concrete |
| 755 | -1 | | 942.5589 | | 1 | 22.41888 | | 217.8208 | | 60.67482 | | Mix_12 | | 218.8478 | | 2640.978 | | 25 ton RR Ties, 25 ton Concrete, 6 Pallets CinderBlock | | | rr ties, concrete, cinderblocks | | | RR, Concrete, CB | | RR, Concrete, CB | | | mid | | 6 | 3 | | 7.848598 | | RR Ties | | RR Ties |
| 756 | -1 | | 327.8892 | | 1 | 21.94251 | | 206.7121 | | 19.13934 | | Mix_37 | | 284.0474 | | 4531.315 | | 25 ton RR Ties, 25 ton Concrete, 6 Pallets CinderBlock | | | rr ties, concrete, cinderblocks | | | RR, Concrete, CB | | RR, Concrete, CB | | | mid | | 6 | 3 | | 6.792676 | | RR Ties | | RR Ties |
| 757 | -1 | | 2102.997 | | 1 | 21.13536 | | 186.0668 | | 30.22459 | | Mix_31 | | 226.9695 | | 1748.217 | | 25 ton RR Ties, 25 ton Concrete, 6 Pallets CinderBlock | | | rr ties, concrete, cinderblocks | | | RR, Concrete, CB | | RR, Concrete, CB | | | mid | | 6 | 3 | | 8.651119 | | RR Ties | | RR Ties |
| 758 | -1 | | 34.55705 | | 1 | 21.52254 | | 223.7878 | | 34.57674 | | Mix_34 | | 245.0884 | | 2582.158 | | 25 ton RR Ties, 25 ton Concrete, 6 Pallets CinderBlock | | | rr ties, concrete, cinderblocks | | | RR, Concrete, CB | | RR, Concrete, CB | | | mid | | 6 | 3 | | 4.542611 | | RR Ties | | RR Ties |
| 759 | -1 | | 692.542 | | 1 | 21.64019 | | 255.287 | | 26.02027 | | Mix_5 | | 216.2756 | | 2299.653 | | 25 ton RR Ties, 25 ton Concrete, 6 Pallets CinderBlock | | | rr ties, concrete, cinderblocks | | | RR, Concrete, CB | | RR, Concrete, CB | | | mid | | 6 | 3 | | 7.540369 | | RR Ties | | RR Ties |
| 760 | -1 | | 5592.453 | | 1 | 21.67016 | | 227.0472 | | 24.06895 | | Mix_5 | | 216.2756 | | 2299.653 | | 25 ton RR Ties, 25 ton Concrete, 6 Pallets CinderBlock | | | rr ties, concrete, cinderblocks | | | RR, Concrete, CB | | RR, Concrete, CB | | | mid | | 6 | 3 | | 9.629173 | | RR Ties | | RR Ties |
| 761 | -1 | | 2845.809 | | 1 | 22.64312 | | 395.3061 | | 39.21448 | | Mix_43 | | 323.2332 | | 5469.278 | | 25 ton RR Ties, 25 ton Concrete, 6 Pallets CinderBlock | | | rr ties, concrete, cinderblocks | | | RR, Concrete, CB | | RR, Concrete, CB | | | mid | | 6 | 3 | | 8.953603 | | RR Ties | | RR Ties |
| 762 | -1 | | 46.46175 | | 1 | 22.57966 | | 613.3228 | | 54.97228 | | Mix_25 | | 280.2439 | | 4038.682 | | 25 ton RR Ties, 25 ton Concrete, 6 Pallets CinderBlock | | | rr ties, concrete, cinderblocks | | | RR, Concrete, CB | | RR, Concrete, CB | | | mid | | 6 | 3 | | 4.838629 | | RR Ties | | RR Ties |
| 763 | -1 | | 607.2436 | | 1 | 22.56004 | | 625.284 | | 44.38921 | | Mix_25 | | 280.2439 | | 4038.682 | | 25 ton RR Ties, 25 ton Concrete, 6 Pallets CinderBlock | | | rr ties, concrete, cinderblocks | | | RR, Concrete, CB | | RR, Concrete, CB | | | mid | | 6 | 3 | | 7.40893 | | RR Ties | | RR Ties |
| 764 | -1 | | 43.53598 | | 1 | 22.63893 | | 609.6303 | | 63.61884 | | Mix_25 | | 280.2439 | | 4038.682 | | 25 ton RR Ties, 25 ton Concrete, 6 Pallets CinderBlock | | | rr ties, concrete, cinderblocks | | | RR, Concrete, CB | | RR, Concrete, CB | | | mid | | 6 | 3 | | 4.773588 | | RR Ties | | RR Ties |
| 765 | -1 | | 2605.997 | | 1 | 22.41214 | | 552.5625 | | 33.0001 | | Mix_26 | | 209.4082 | | 2473.816 | | 25 ton RR Ties, 25 ton Concrete, 6 Pallets CinderBlock | | | rr ties, concrete, cinderblocks | | | RR, Concrete, CB | | RR, Concrete, CB | | | mid | | 6 | 3 | | 8.865571 | | RR Ties | | RR Ties |
| 766 | -1 | | 942.0655 | | 47 | 21.50042 | | 243.8403 | | 38.06578 | | Mix_5 | | 216.2756 | | 2299.653 | | 25 ton RR Ties, 25 ton Concrete, 6 Pallets CinderBlock | | | rr ties, concrete, cinderblocks | | | RR, Concrete, CB | | RR, Concrete, CB | | | mid | | 6 | 3 | | 7.848075 | | RR Ties | | RR Ties |
| 767 | -1 | | 1303.572 | | 1 | 22.48554 | | 208.005 | | 14.79747 | | Mix_12 | | 218.8478 | | 2640.978 | | 25 ton RR Ties, 25 ton Concrete, 6 Pallets CinderBlock | | | rr ties, concrete, cinderblocks | | | RR, Concrete, CB | | RR, Concrete, CB | | | mid | | 6 | 3 | | 8.172864 | | RR Ties | | RR Ties |
| 768 | -1 | | 86.7102 | | 1 | 21.88644 | | 670.1267 | | 17.61436 | | Mix_28 | | 343.8062 | | 3553.502 | | 25 ton RR Ties, 25 ton Concrete, 6 Pallets CinderBlock | | | rr ties, concrete, cinderblocks | | | RR, Concrete, CB | | RR, Concrete, CB | | | mid | | 6 | 3 | | 5.462572 | | RR Ties | | RR Ties |
| 769 | -1 | | 348.5601 | | 1 | 21.27811 | | 198.0183 | | 21.71764 | | Mix_29 | | 322.2633 | | 5654.458 | | 25 ton RR Ties, 25 ton Concrete, 6 Pallets CinderBlock | | | rr ties, concrete, cinderblocks | | | RR, Concrete, CB | | RR, Concrete, CB | | | mid | | 6 | 3 | | 6.853811 | | RR Ties | | RR Ties |
| 770 | -1 | | 859.2773 | | 1 | 21.85933 | | 207.7234 | | 6.040595 | | Mix_35 | | 233.2249 | | 2659.636 | | 25 ton RR Ties, 25 ton Concrete, 6 Pallets CinderBlock | | | rr ties, concrete, cinderblocks | | | RR, Concrete, CB | | RR, Concrete, CB | | | mid | | 6 | 3 | | 7.756092 | | RR Ties | | RR Ties |
| 771 | -1 | | 836.2088 | | 1 | 21.86667 | | 189.9249 | | 16.72658 | | Mix_36 | | 231.0741 | | 2931.041 | | 25 ton RR Ties, 25 ton Concrete, 6 Pallets CinderBlock | | | rr ties, concrete, cinderblocks | | | RR, Concrete, CB | | RR, Concrete, CB | | | mid | | 6 | 3 | | 7.728878 | | RR Ties | | RR Ties |
| 772 | -1 | | 218.6577 | | 1 | 21.97068 | | 202.6513 | | 3.076164 | | Mix_37 | | 284.0474 | | 4531.315 | | 25 ton RR Ties, 25 ton Concrete, 6 Pallets CinderBlock | | | rr ties, concrete, cinderblocks | | | RR, Concrete, CB | | RR, Concrete, CB | | | mid | | 6 | 3 | | 6.387508 | | RR Ties | | RR Ties |
| 773 | -1 | | 725.4587 | | 1 | 22.71553 | | 291.9039 | | 24.9702 | | Mix_42 | | 221.5067 | | 2829.814 | | 25 ton RR Ties, 25 ton Concrete, 6 Pallets CinderBlock | | | rr ties, concrete, cinderblocks | | | RR, Concrete, CB | | RR, Concrete, CB | | | mid | | 6 | 3 | | 7.586804 | | RR Ties | | RR Ties |
| 774 | -1 | | 496.3907 | | 1 | 21.61471 | | 267.9071 | | 12.52677 | | Mix_6 | | 200.4879 | | 2070.031 | | 25 ton RR Ties, 25 ton Concrete, 6 Pallets CinderBlock | | | rr ties, concrete, cinderblocks | | | RR, Concrete, CB | | RR, Concrete, CB | | | mid | | 6 | 3 | | 7.207363 | | RR Ties | | RR Ties |
| 775 | -1 | | 408.3771 | | 20 | 21.8052 | | 263.6286 | | 7.122224 | | Mix_7 | | 352.5258 | | 4514.797 | | 25 ton RR Ties, 25 ton Concrete, 6 Pallets CinderBlock | | | rr ties, concrete, cinderblocks | | | RR, Concrete, CB | | RR, Concrete, CB | | | mid | | 6 | 3 | | 7.012191 | | RR Ties | | RR Ties |
| 776 | -1 | | 4907.148 | | 1 | 22.94979 | | 355.4357 | | 42.92172 | | D-15 | | 570.4288 | | 18953.98 | | Biggest spoked spool, 12ft spools, concrete railroad ties | | | rr ties, spools | | | RR, Spools | | RR, Spools | | | mid-high | | 5 | 2 | | 9.498448 | | Other / Mixed | | RR Ties |
| 777 | -1 | | 20141.91 | | 1 | 22.9983 | | 354.8851 | | 52.62029 | | D-15 | | 570.4288 | | 18953.98 | | Biggest spoked spool, 12ft spools, concrete railroad ties | | | rr ties, spools | | | RR, Spools | | RR, Spools | | | mid-high | | 5 | 2 | | 10.91056 | | Other / Mixed | | RR Ties |
| 778 | -1 | | 133.2225 | | 1 | 22.88604 | | 534.8803 | | 59.59395 | | D-14 | | 833.029 | | 42829.89 | | Biggest spoked spool, 12ft spools, 5ft spools, concrete railroad ties | | | rr ties, spools | | | RR, Spools | | RR, Spools | | | mid-high | | 5 | 2 | | 5.892021 | | Other / Mixed | | RR Ties |
| 779 | -1 | | 377.5675 | | 1 | 23.26319 | | 246.0834 | | 14.81193 | | D-8 | | 779.046 | | 37066.62 | | railroadties, Largest Spool Medium Spool | | | rr ties, spools | | | RR, Spools | | RR, Spools | | | mid-high | | 5 | 2 | | 6.933749 | | Other / Mixed | | RR Ties |
| 780 | -1 | | 161.8998 | | 1 | 22.68296 | | 545.9077 | | 38.76801 | | D-12 | | 917.8977 | | 40499.67 | | 18' Drum 300 ties 4 medium spools | | | rr ties, spools, drum | | | rr ties, spools, drum | | RR, Spools | | | mid-high | | 5 | 2 | | 6.086978 | | Other / Mixed | | RR Ties |
| 781 | -1 | | 3960.034 | | 1 | 22.63722 | | 600.0974 | | 36.58818 | | D-12 | | 917.8977 | | 40499.67 | | 18' Drum 300 ties 4 medium spools | | | rr ties, spools, drum | | | rr ties, spools, drum | | RR, Spools | | | mid-high | | 5 | 2 | | 9.284008 | | Other / Mixed | | RR Ties |
| 782 | -1 | | 3312.406 | | 1 | 22.64613 | | 514.8869 | | 35.67088 | | D-14 | | 833.029 | | 42829.89 | | Biggest spoked spool, 12ft spools, 5ft spools, concrete railroad ties | | | rr ties, spools | | | RR, Spools | | RR, Spools | | | mid-high | | 5 | 2 | | 9.10543 | | Other / Mixed | | RR Ties |
| 783 | -1 | | 48.2624 | | 2 | 22.88402 | | 389.3199 | | 7.561146 | | D-15 | | 570.4288 | | 18953.98 | | Biggest spoked spool, 12ft spools, concrete railroad ties | | | rr ties, spools | | | RR, Spools | | RR, Spools | | | mid-high | | 5 | 2 | | 4.876653 | | Other / Mixed | | RR Ties |
| 784 | -1 | | 41.42202 | | 1 | 22.78886 | | 467.0301 | | 28.35351 | | D-2 | | 699.6385 | | 27098.22 | | 1 large spool 400 ties | | | rr ties, spools | | | RR, Spools | | RR, Spools | | | mid-high | | 5 | 2 | | 4.723813 | | Other / Mixed | | RR Ties |
| 785 | -1 | | 9457.06 | | 1 | 22.91692 | | 395.4893 | | 23.28816 | | D-3 | | 882.0725 | | 34901.06 | | 3 medium spools, 30 small spools. railroad ties | | | rr ties, spools | | | RR, Spools | | RR, Spools | | | mid-high | | 5 | 2 | | 10.15452 | | Other / Mixed | | RR Ties |
| 786 | -1 | | 611.4184 | | 1 | 22.8084 | | 431.3273 | | 13.26462 | | D-3 | | 882.0725 | | 34901.06 | | 3 medium spools, 30 small spools. railroad ties | | | rr ties, spools | | | RR, Spools | | RR, Spools | | | mid-high | | 5 | 2 | | 7.415782 | | Other / Mixed | | RR Ties |
| 787 | -1 | | 21598.16 | | 1 | 22.92209 | | 411.7541 | | 15.76039 | | D-3 | | 882.0725 | | 34901.06 | | 3 medium spools, 30 small spools. railroad ties | | | rr ties, spools | | | RR, Spools | | RR, Spools | | | mid-high | | 5 | 2 | | 10.98036 | | Other / Mixed | | RR Ties |
| 788 | -1 | | 642.3136 | | 1 | 23.08248 | | 244.9776 | | 32.99038 | | D-22 | | 446.7276 | | 12921.52 | | Spools, concrete railroad ties | | | rr ties, spools | | | RR, Spools | | RR, Spools | | | mid-high | | 5 | 2 | | 7.465077 | | Other / Mixed | | RR Ties |
| 789 | -1 | | 471.4745 | | 1 | 23.08431 | | 265.5328 | | 23.75955 | | D-23 | | 821.8087 | | 31780.67 | | Spools, broken concrete | | | rr ties, spools, concrete | | | RR, Spools, Concrete | | RR, Spools, Concrete | | | mid-high | | 5 | 3 | | 7.155865 | | Other / Mixed | | RR Ties |
| 790 | -1 | | 4301.992 | | 1 | 23.34468 | | 86.17786 | | 44.76548 | | D-20 | | 455.1934 | | 10831.77 | | Spools, concrete railroad ties | | | rr ties, spools, concrete | | | RR, Spools, Concrete | | RR, Spools, Concrete | | | mid-high | | 5 | 3 | | 9.366834 | | Other / Mixed | | RR Ties |
| 791 | -1 | | 576.8209 | | 1 | 23.07759 | | 286.7624 | | 17.39202 | | D-23 | | 821.8087 | | 31780.67 | | Spools, broken concrete | | | rr ties, spools, concrete | | | RR, Spools, Concrete | | RR, Spools, Concrete | | | mid-high | | 5 | 3 | | 7.357532 | | Other / Mixed | | RR Ties |
| 792 | -1 | | 121.8326 | | 1 | 22.9238 | | 308.5894 | | 23.3635 | | D-23 | | 821.8087 | | 31780.67 | | Spools, broken concrete | | | rr ties, spools, concrete | | | RR, Spools, Concrete | | RR, Spools, Concrete | | | mid-high | | 5 | 3 | | 5.802648 | | Other / Mixed | | RR Ties |
| 793 | -1 | | 141.3196 | | 1 | 22.6584 | | 528.2085 | | 54.3789 | | d-26 big spool | | 860.7689 | | 30331.27 | | Biggest spool with bouy and broken concrete | | | spools, concrete | | | rr ties, spools, concrete | | RR, Spools, Concrete | | | mid-high | | 5 | 3 | | 5.951024 | | Other / Mixed | | RR Ties |
| 794 | -1 | | 277.3657 | | 14 | 23.25336 | | 138.5313 | | 38.24493 | | D-20 | | 455.1934 | | 10831.77 | | Spools, concrete railroad ties | | | rr ties, spools, concrete | | | RR, Spools, Concrete | | RR, Spools, Concrete | | | mid-high | | 5 | 3 | | 6.625337 | | Other / Mixed | | RR Ties |
| 795 | -1 | | 1471.866 | | 74 | 22.68694 | | 555.5987 | | 31.43537 | | d-26 big spool | | 860.7689 | | 30331.27 | | Biggest spool with bouy and broken concrete | | | spools, concrete | | | rr ties, spools, concrete | | RR, Spools, Concrete | | | mid-high | | 5 | 3 | | 8.294286 | | Other / Mixed | | RR Ties |
| 796 | -1 | | 4601.3 | | 1 | 23.21698 | | 148.0925 | | 17.34442 | | D-20 | | 455.1934 | | 10831.77 | | Spools, concrete railroad ties | | | rr ties, spools, concrete | | | RR, Spools, Concrete | | RR, Spools, Concrete | | | mid-high | | 5 | 3 | | 9.434094 | | Other / Mixed | | RR Ties |
| 797 | -1 | | 7548.062 | | 1 | 22.59202 | | 553.0525 | | 29.7301 | | D-1 | | 857.3153 | | 41943.89 | | Large Spool, broken ties, 15ft Spools | | | rr ties, spools, concrete | | | RR, Spools, Concrete | | RR, Spools, Concrete | | | mid-high | | 5 | 3 | | 9.929046 | | Other / Mixed | | Pyramids |
| 798 | -1 | | 9998.864 | | 1 | 22.52082 | | 571.7994 | | 8.141447 | | D-1 | | 857.3153 | | 41943.89 | | Large Spool, broken ties, 15ft Spools | | | rr ties, spools, concrete | | | RR, Spools, Concrete | | RR, Spools, Concrete | | | mid-high | | 5 | 3 | | 10.21023 | | Other / Mixed | | Pyramids |
| 799 | -1 | | 2023.941 | | 1 | 23.22989 | | 134.15 | | 21.22489 | | D-21 | | 566.7995 | | 13511.86 | | Spools, concrete railroad ties | | | rr ties, spools, concrete | | | RR, Spools, Concrete | | RR, Spools, Concrete | | | mid-high | | 5 | 3 | | 8.612802 | | Other / Mixed | | Pyramids |
| 800 | -1 | | 238.7371 | | 1 | 22.97593 | | 511.5395 | | 38.43731 | | D-25 | | 724.2878 | | 27444.08 | | Spools, broken concrete | | | rr ties, spools, concrete | | | RR, Spools, Concrete | | RR, Spools, Concrete | | | mid-high | | 5 | 3 | | 6.475363 | | Other / Mixed | | Pyramids |
| 801 | -1 | | 745.8772 | | 1 | 22.68789 | | 494.2614 | | 3.079969 | | D-25 | | 724.2878 | | 27444.08 | | Spools, broken concrete | | | rr ties, spools, concrete | | | RR, Spools, Concrete | | RR, Spools, Concrete | | | mid-high | | 5 | 3 | | 7.614561 | | Other / Mixed | | Pyramids |
| 802 | -1 | | 2932.068 | | 1 | 22.7873 | | 348.4391 | | 46.07053 | | D-4 | | 843.0299 | | 33942.2 | | Large Spool, ties, culverts | | | rr ties, spools, culverts | | | RR, Spools, Culvert | | RR, Spools, Culvert | | | mid-high | | 5 | 3 | | 8.983463 | | Other / Mixed | | Boat |
| 803 | -1 | | 1545.355 | | 1 | 22.87031 | | 350.0652 | | 38.51041 | | D-4 | | 843.0299 | | 33942.2 | | Large Spool, ties, culverts | | | rr ties, spools, culverts | | | RR, Spools, Culvert | | RR, Spools, Culvert | | | mid-high | | 5 | 3 | | 8.343009 | | Other / Mixed | | Boat |
| 804 | -1 | | 3356.088 | | 1 | 21.58744 | | 817.4786 | | 47.64918 | | D-27 | | 1459.357 | | 75504.47 | | Spools, culverts and railroad ties | | | rr ties, spools, culverts | | | RR, Spools, Culvert | | RR, Spools, Culvert | | | mid-high | | 5 | 3 | | 9.118531 | | Other / Mixed | | Boat |
| 805 | -1 | | 60.18487 | | 1 | 21.62123 | | 799.0341 | | 11.75325 | | D-27 | | 1459.357 | | 75504.47 | | Spools, culverts and railroad ties | | | rr ties, spools, culverts | | | RR, Spools, Culvert | | RR, Spools, Culvert | | | mid-high | | 5 | 3 | | 5.097421 | | Other / Mixed | | Boat |
| 806 | -1 | | 9256.618 | | 1 | 22.92746 | | 193.6463 | | 46.94331 | | D-5 | | 945.796 | | 41554.73 | | Large Spool, ties, culverts | | | rr ties, spools, culverts | | | RR, Spools, Culvert | | RR, Spools, Culvert | | | mid-high | | 5 | 3 | | 10.13309 | | Other / Mixed | | Boat |
| 807 | -1 | | 361.7348 | | 18 | 23.10555 | | 227.0114 | | 12.84489 | | D-5 | | 945.796 | | 41554.73 | | Large Spool, ties, culverts | | | rr ties, spools, culverts | | | RR, Spools, Culvert | | RR, Spools, Culvert | | | mid-high | | 5 | 3 | | 6.890911 | | Other / Mixed | | Boat |
| OBJECTID | | CLUSTER_ID | | Weight_g | | | Count_fish | | Avg_depth_m | | Dist_boundary_m | | Dist_near_site_m | | Near_site | | structure_length_m | | structure_area_m | material | | structure | structure_category_number | | structure_category | | relief_category | site_age | | number_materials | | | log_weight | | Weight_kg | | structure2 | |
| 59 | | 59 | | 92713.52 | | | 842.9898 | | 20.7573 | | 781.6825 | | 20.70077 | | Andy Faskin | | 26.78074 | | 40.04824 | boat | | boat | Boats | | Boats | | high | 4 | | 1 | | | 12.43727 | | 92.71352 | | Boat | |
| 62 | | 62 | | 807317 | | | 33920.08 | | 21.89843 | | 737.1365 | | 10.71175 | | Billy Kenon | | 41.08165 | | 86.81695 | boat | | boat | Boats | | Boats | | high | 4 | | 1 | | | 14.60147 | | 807.317 | | Boat | |
| 63 | | 63 | | 346756.1 | | | 17296.81 | | 21.89091 | | 725.5797 | | 22.30174 | | Billy Kenon | | 41.08165 | | 86.81695 | boat | | boat | Boats | | Boats | | high | 4 | | 1 | | | 13.75638 | | 346.7561 | | Boat | |
| 64 | | 64 | | 46522.68 | | | 143.3748 | | 21.80369 | | 682.631 | | 63.20084 | | Billy Kenon | | 41.08165 | | 86.81695 | boat | | boat | Boats | | Boats | | high | 4 | | 1 | | | 11.7477 | | 46.52268 | | Boat | |
| 210 | | 210 | | 1084.114 | | | 53.78725 | | 21.07874 | | 753.9202 | | 34.26538 | | Andy Faskin | | 26.78074 | | 40.04824 | boat | | boat | Boats | | Boats | | high | 4 | | 1 | | | 7.988519 | | 1.084114 | | Boat | |
| 245 | | 245 | | 40098.22 | | | 26 | | 21.64663 | | 840.9918 | | 46.30599 | | SPI Ceviche | | 52.22029 | | 103.1575 | boat | | boat | Boats | | Boats | | high | 4 | | 1 | | | 11.59909 | | 40.09822 | | Boat | |
| 249 | | 249 | | 20181.06 | | | 9 | | 21.66571 | | 868.9391 | | 63.42377 | | SPI Ceviche | | 52.22029 | | 103.1575 | boat | | boat | Boats | | Boats | | high | 4 | | 1 | | | 10.9125 | | 20.18106 | | Boat | |
| 250 | | 250 | | 67773.2 | | | 3388.66 | | 21.59077 | | 825.0619 | | 11.3319 | | SPI Ceviche | | 52.22029 | | 103.1575 | boat | | boat | Boats | | Boats | | high | 4 | | 1 | | | 12.12392 | | 67.7732 | | Boat | |
| 368 | | 368 | | 10857.5 | | | 16 | | 21.79712 | | 880.3271 | | 65.74299 | | SPI Ceviche | | 52.22029 | | 103.1575 | boat | | boat | Boats | | Boats | | high | 4 | | 1 | | | 10.29261 | | 10.8575 | | Boat | |
| 369 | | 369 | | 20138.36 | | | 15 | | 21.62304 | | 812.8725 | | 31.34575 | | SPI Ceviche | | 52.22029 | | 103.1575 | boat | | boat | Boats | | Boats | | high | 4 | | 1 | | | 10.91038 | | 20.13836 | | Boat | |
| 374 | | 374 | | 4313.709 | | | 4 | | 21.85948 | | 727.26 | | 36.34531 | | Billy Kenon | | 41.08165 | | 86.81695 | boat | | boat | Boats | | Boats | | high | 4 | | 1 | | | 9.369553 | | 4.313709 | | Boat | |
| 395 | | 395 | | 37768.15 | | | 86 | | 20.74204 | | 763.6277 | | 27.30253 | | Andy Faskin | | 26.78074 | | 40.04824 | boat | | boat | Boats | | Boats | | high | 4 | | 1 | | | 11.53922 | | 37.76815 | | Boat | |
| 581 | | -1 | | 11019.6 | | | 550.9799 | | 21.59402 | | 812.2422 | | 14.29769 | | SPI Ceviche | | 52.22029 | | 103.1575 | boat | | boat | Boats | | Boats | | high | 4 | | 1 | | | 10.30743 | | 11.0196 | | Boat | |
| 677 | | -1 | | 473.7953 | | | 1 | | 21.74946 | | 867.0853 | | 50.74365 | | SPI Ceviche | | 52.22029 | | 103.1575 | boat | | boat | Boats | | Boats | | high | 4 | | 1 | | | 7.160775 | | 0.473795 | | Boat | |
| 688 | | -1 | | 4474.222 | | | 1 | | 21.82915 | | 687.1144 | | 65.30378 | | Billy Kenon | | 41.08165 | | 86.81695 | boat | | boat | Boats | | Boats | | high | 4 | | 1 | | | 9.406088 | | 4.474222 | | Boat | |
| 700 | | -1 | | 775.9765 | | | 1 | | 20.78046 | | 753.0421 | | 13.15506 | | Andy Faskin | | 26.78074 | | 40.04824 | boat | | boat | Boats | | Boats | | high | 4 | | 1 | | | 7.654122 | | 0.775977 | | Boat | |
| 78 | | 78 | | 2114.738 | | | 5 | | 23.01328 | | 231.2261 | | 22.23566 | | D-16 | | 160.7399 | | 1564.646 | Concrete Railroad Ties | | rr ties | RR Ties | | RR | | mid | 4 | | 1 | | | 8.656686 | | 2.114738 | | RR Ties | |
| 301 | | 301 | | 20561.46 | | | 75 | | 22.95845 | | 238.1145 | | 62.20016 | | D-16 | | 160.7399 | | 1564.646 | Concrete Railroad Ties | | rr ties | RR Ties | | RR | | mid | 4 | | 1 | | | 10.93117 | | 20.56146 | | RR Ties | |
| 627 | | -1 | | 1359.012 | | | 1 | | 23.09453 | | 169.9363 | | 53.38785 | | D-17 | | 159.1919 | | 1374.586 | Concrete Railroad Ties | | rr ties | RR Ties | | RR | | mid | 4 | | 1 | | | 8.214513 | | 1.359012 | | RR Ties | |
| 76 | | 76 | | 102902.3 | | | 3 | | 22.83786 | | 401.9798 | | 8.178199 | | D-10 | | 273.7191 | | 4474.659 | 1 large spool 300 ties | | rr ties, spools | RR, Spools | | RR, Spools | | mid-high | 4 | | 2 | | | 12.54154 | | 102.9023 | | Other / Mixed | |
| 77 | | 77 | | 143850.4 | | | 7192.519 | | 22.60737 | | 478.1586 | | 12.20732 | | D-11 | | 246.9203 | | 3573.997 | 1 large spool 300 ties | | rr ties, spools | RR, Spools | | RR, Spools | | mid-high | 4 | | 2 | | | 12.87653 | | 143.8504 | | Other / Mixed | |
| 80 | | 80 | | 43096.03 | | | 7.954752 | | 22.6115 | | 495.0398 | | 9.328321 | | D-2 | | 213.2394 | | 2517.486 | 1 large spool 400 ties | | rr ties, spools | RR, Spools | | RR, Spools | | mid-high | 4 | | 2 | | | 11.67119 | | 43.09603 | | Other / Mixed | |
| 87 | | 87 | | 69573.91 | | | 9 | | 23.18198 | | 210.6305 | | 11.1882 | | D-22 | | 136.1559 | | 1200.439 | Spools, concrete railroad ties | | rr ties, spools | RR, Spools | | RR, Spools | | mid-high | 4 | | 2 | | | 12.15014 | | 69.57391 | | Other / Mixed | |
| 94 | | 94 | | 8836.447 | | | 4 | | 22.95683 | | 393.3551 | | 30.89439 | | D-3 | | 268.8426 | | 3242.388 | 3 medium spools, 30 small spools. railroad ties | | rr ties, spools | RR, Spools | | RR, Spools | | mid-high | 4 | | 2 | | | 10.08664 | | 8.836447 | | Other / Mixed | |
| 95 | | 95 | | 4519.431 | | | 3 | | 22.91621 | | 378.6715 | | 39.56568 | | D-3 | | 268.8426 | | 3242.388 | 3 medium spools, 30 small spools. railroad ties | | rr ties, spools | RR, Spools | | RR, Spools | | mid-high | 4 | | 2 | | | 9.416141 | | 4.519431 | | Other / Mixed | |
| 98 | | 98 | | 45707.53 | | | 8 | | 23.02034 | | 201.528 | | 33.99438 | | D-8 | | 237.4416 | | 3443.573 | railroadties, Largest Spool Medium Spool | | rr ties, spools | RR, Spools | | RR, Spools | | mid-high | 4 | | 2 | | | 11.73002 | | 45.70753 | | Other / Mixed | |
| 302 | | 302 | | 48801.56 | | | 41 | | 23.09358 | | 201.2303 | | 60.56645 | | D-22 | | 136.1559 | | 1200.439 | Spools, concrete railroad ties | | rr ties, spools | RR, Spools | | RR, Spools | | mid-high | 4 | | 2 | | | 11.79552 | | 48.80156 | | Other / Mixed | |
| 345 | | 345 | | 164101.8 | | | 2 | | 22.68858 | | 420.3397 | | 31.04581 | | D-10 | | 273.7191 | | 4474.659 | 1 large spool 300 ties | | rr ties, spools | RR, Spools | | RR, Spools | | mid-high | 4 | | 2 | | | 13.00824 | | 164.1018 | | Other / Mixed | |
| 346 | | 346 | | 76794.28 | | | 563 | | 23.06091 | | 257.7498 | | 57.60065 | | D-22 | | 136.1559 | | 1200.439 | Spools, concrete railroad ties | | rr ties, spools | RR, Spools | | RR, Spools | | mid-high | 4 | | 2 | | | 12.24889 | | 76.79428 | | Other / Mixed | |
| 349 | | 349 | | 3976.118 | | | 2 | | 22.73012 | | 400.5242 | | 29.48943 | | D-10 | | 273.7191 | | 4474.659 | 1 large spool 300 ties | | rr ties, spools | RR, Spools | | RR, Spools | | mid-high | 4 | | 2 | | | 9.288061 | | 3.976118 | | Other / Mixed | |
| 350 | | 350 | | 4172.59 | | | 2 | | 22.84347 | | 368.9451 | | 38.02096 | | D-10 | | 273.7191 | | 4474.659 | 1 large spool 300 ties | | rr ties, spools | RR, Spools | | RR, Spools | | mid-high | 4 | | 2 | | | 9.336292 | | 4.17259 | | Other / Mixed | |
| 351 | | 351 | | 293.7421 | | | 2 | | 22.54459 | | 529.8249 | | 54.26203 | | D-11 | | 246.9203 | | 3573.997 | 1 large spool 300 ties | | rr ties, spools | RR, Spools | | RR, Spools | | mid-high | 4 | | 2 | | | 6.682702 | | 0.293742 | | Other / Mixed | |
| 352 | | 352 | | 44497.72 | | | 2 | | 22.69814 | | 424.8896 | | 19.97596 | | D-10 | | 273.7191 | | 4474.659 | 1 large spool 300 ties | | rr ties, spools | RR, Spools | | RR, Spools | | mid-high | 4 | | 2 | | | 11.70319 | | 44.49772 | | Other / Mixed | |
| 353 | | 353 | | 7455.672 | | | 2 | | 23.00261 | | 244.939 | | 12.20067 | | D-8 | | 237.4416 | | 3443.573 | railroadties, Largest Spool Medium Spool | | rr ties, spools | RR, Spools | | RR, Spools | | mid-high | 4 | | 2 | | | 9.91673 | | 7.455672 | | Other / Mixed | |
| 392 | | 392 | | 1459.946 | | | 7 | | 23.00085 | | 181.1785 | | 52.41219 | | D-8 | | 237.4416 | | 3443.573 | railroadties, Largest Spool Medium Spool | | rr ties, spools | RR, Spools | | RR, Spools | | mid-high | 4 | | 2 | | | 8.286155 | | 1.459946 | | Other / Mixed | |
| 407 | | 407 | | 3388.385 | | | 170 | | 22.93899 | | 395.3016 | | 59.23784 | | D-15 | | 173.8582 | | 1760.868 | Biggest spoked spool, 12ft spools, concrete railroad ties | | rr ties, spools | RR, Spools | | RR, Spools | | mid-high | 4 | | 2 | | | 9.128109 | | 3.388385 | | Other / Mixed | |
| 475 | | -1 | | 3312.406 | | | 1 | | 22.64613 | | 514.8869 | | 35.67088 | | D-14 | | 253.8949 | | 3978.994 | Biggest spoked spool, 12ft spools, 5ft spools, concrete railroad ties | | rr ties, spools | RR, Spools | | RR, Spools | | mid-high | 4 | | 2 | | | 9.10543 | | 3.312406 | | Other / Mixed | |
| 476 | | -1 | | 48.2624 | | | 2.41312 | | 22.88402 | | 389.3199 | | 7.561146 | | D-15 | | 173.8582 | | 1760.868 | Biggest spoked spool, 12ft spools, concrete railroad ties | | rr ties, spools | RR, Spools | | RR, Spools | | mid-high | 4 | | 2 | | | 4.876653 | | 0.048262 | | Other / Mixed | |
| 477 | | -1 | | 41.42202 | | | 1 | | 22.78886 | | 467.0301 | | 28.35351 | | D-2 | | 213.2394 | | 2517.486 | 1 large spool 400 ties | | rr ties, spools | RR, Spools | | RR, Spools | | mid-high | 4 | | 2 | | | 4.723813 | | 0.041422 | | Other / Mixed | |
| 480 | | -1 | | 642.3136 | | | 1 | | 23.08248 | | 244.9776 | | 32.99038 | | D-22 | | 136.1559 | | 1200.439 | Spools, concrete railroad ties | | rr ties, spools | RR, Spools | | RR, Spools | | mid-high | 4 | | 2 | | | 7.465077 | | 0.642314 | | Other / Mixed | |
| 483 | | -1 | | 9457.06 | | | 1 | | 22.91692 | | 395.4893 | | 23.28816 | | D-3 | | 268.8426 | | 3242.388 | 3 medium spools, 30 small spools. railroad ties | | rr ties, spools | RR, Spools | | RR, Spools | | mid-high | 4 | | 2 | | | 10.15452 | | 9.45706 | | Other / Mixed | |
| 484 | | -1 | | 611.4184 | | | 1 | | 22.8084 | | 431.3273 | | 13.26462 | | D-3 | | 268.8426 | | 3242.388 | 3 medium spools, 30 small spools. railroad ties | | rr ties, spools | RR, Spools | | RR, Spools | | mid-high | 4 | | 2 | | | 7.415782 | | 0.611418 | | Other / Mixed | |
| 485 | | -1 | | 21598.16 | | | 1 | | 22.92209 | | 411.7541 | | 15.76039 | | D-3 | | 268.8426 | | 3242.388 | 3 medium spools, 30 small spools. railroad ties | | rr ties, spools | RR, Spools | | RR, Spools | | mid-high | 4 | | 2 | | | 10.98036 | | 21.59816 | | Other / Mixed | |
| 624 | | -1 | | 4907.148 | | | 1 | | 22.94979 | | 355.4357 | | 42.92172 | | D-15 | | 173.8582 | | 1760.868 | Biggest spoked spool, 12ft spools, concrete railroad ties | | rr ties, spools | RR, Spools | | RR, Spools | | mid-high | 4 | | 2 | | | 9.498448 | | 4.907148 | | Other / Mixed | |
| 665 | | -1 | | 20141.91 | | | 1 | | 22.9983 | | 354.8851 | | 52.62029 | | D-15 | | 173.8582 | | 1760.868 | Biggest spoked spool, 12ft spools, concrete railroad ties | | rr ties, spools | RR, Spools | | RR, Spools | | mid-high | 4 | | 2 | | | 10.91056 | | 20.14191 | | Other / Mixed | |
| 668 | | -1 | | 133.2225 | | | 1 | | 22.88604 | | 534.8803 | | 59.59395 | | D-14 | | 253.8949 | | 3978.994 | Biggest spoked spool, 12ft spools, 5ft spools, concrete railroad ties | | rr ties, spools | RR, Spools | | RR, Spools | | mid-high | 4 | | 2 | | | 5.892021 | | 0.133223 | | Other / Mixed | |
| 670 | | -1 | | 377.5675 | | | 1 | | 23.26319 | | 246.0834 | | 14.81193 | | D-8 | | 237.4416 | | 3443.573 | railroadties, Largest Spool Medium Spool | | rr ties, spools | RR, Spools | | RR, Spools | | mid-high | 4 | | 2 | | | 6.933749 | | 0.377568 | | Other / Mixed | |
| 672 | | -1 | | 161.8998 | | | 1 | | 22.68296 | | 545.9077 | | 38.76801 | | D-12 | | 279.7616 | | 3762.511 | 18' Drum 300 ties 4 medium spools | | rr ties, spools, drum | RR, Spools | | RR, Spools | | mid-high | 4 | | 2 | | | 6.086978 | | 0.1619 | | Other / Mixed | |
| 731 | | -1 | | 3960.034 | | | 1 | | 22.63722 | | 600.0974 | | 36.58818 | | D-12 | | 279.7616 | | 3762.511 | 18' Drum 300 ties 4 medium spools | | rr ties, spools, drum | RR, Spools | | RR, Spools | | mid-high | 4 | | 2 | | | 9.284008 | | 3.960034 | | Other / Mixed | |
| 74 | | 74 | | 68004.66 | | | 2 | | 22.56912 | | 571 | | 13.96544 | | D-1 | | 261.2969 | | 3896.682 | Large Spool, broken ties, 15ft Spools | | rr ties, spools, concrete | RR, Spools, Concrete | | RR, Spools, Concrete | | mid-high | 4 | | 3 | | | 12.12733 | | 68.00466 | | Other / Mixed | |
| 75 | | 75 | | 13175.01 | | | 2 | | 22.61471 | | 587.9898 | | 8.069768 | | D-1 | | 261.2969 | | 3896.682 | Large Spool, broken ties, 15ft Spools | | rr ties, spools, concrete | RR, Spools, Concrete | | RR, Spools, Concrete | | mid-high | 4 | | 3 | | | 10.48608 | | 13.17501 | | Other / Mixed | |
| 81 | | 81 | | 21195.17 | | | 5 | | 23.29484 | | 128.248 | | 2.562263 | | D-20 | | 138.7362 | | 1006.296 | Spools, concrete railroad ties | | rr ties, spools, concrete | RR, Spools, Concrete | | RR, Spools, Concrete | | mid-high | 4 | | 3 | | | 10.96153 | | 21.19517 | | Other / Mixed | |
| 82 | | 82 | | 34581.36 | | | 167.0502 | | 23.30604 | | 124.4149 | | 1.0942 | | D-21 | | 172.7521 | | 1255.282 | Spools, concrete railroad ties | | rr ties, spools, concrete | RR, Spools, Concrete | | RR, Spools, Concrete | | mid-high | 4 | | 3 | | | 11.45107 | | 34.58136 | | Other / Mixed | |
| 83 | | 83 | | 174711.7 | | | 34 | | 23.24285 | | 112.9677 | | 30.51856 | | D-21 | | 172.7521 | | 1255.282 | Spools, concrete railroad ties | | rr ties, spools, concrete | RR, Spools, Concrete | | RR, Spools, Concrete | | mid-high | 4 | | 3 | | | 13.07089 | | 174.7117 | | Other / Mixed | |
| 84 | | 84 | | 45799.88 | | | 8 | | 23.24887 | | 142.1649 | | 20.59271 | | D-21 | | 172.7521 | | 1255.282 | Spools, concrete railroad ties | | rr ties, spools, concrete | RR, Spools, Concrete | | RR, Spools, Concrete | | mid-high | 4 | | 3 | | | 11.73204 | | 45.79988 | | Other / Mixed | |
| 85 | | 85 | | 1322.644 | | | 2 | | 23.31037 | | 97.94444 | | 28.79552 | | D-21 | | 172.7521 | | 1255.282 | Spools, concrete railroad ties | | rr ties, spools, concrete | RR, Spools, Concrete | | RR, Spools, Concrete | | mid-high | 4 | | 3 | | | 8.187388 | | 1.322644 | | Other / Mixed | |
| 86 | | 86 | | 7604.901 | | | 3 | | 23.26286 | | 143.8313 | | 23.25868 | | D-21 | | 172.7521 | | 1255.282 | Spools, concrete railroad ties | | rr ties, spools, concrete | RR, Spools, Concrete | | RR, Spools, Concrete | | mid-high | 4 | | 3 | | | 9.936548 | | 7.604901 | | Other / Mixed | |
| 88 | | 88 | | 144069.4 | | | 196.236 | | 22.99584 | | 261.3708 | | 34.21073 | | D-23 | | 250.4751 | | 2952.496 | Spools, broken concrete | | rr ties, spools, concrete | RR, Spools, Concrete | | RR, Spools, Concrete | | mid-high | 4 | | 3 | | | 12.87805 | | 144.0694 | | Other / Mixed | |
| 89 | | 89 | | 5177.654 | | | 2 | | 23.04404 | | 262.5605 | | 23.34333 | | D-23 | | 250.4751 | | 2952.496 | Spools, broken concrete | | rr ties, spools, concrete | RR, Spools, Concrete | | RR, Spools, Concrete | | mid-high | 4 | | 3 | | | 9.552107 | | 5.177654 | | Other / Mixed | |
| 90 | | 90 | | 9523.53 | | | 3 | | 22.9577 | | 273.8912 | | 59.03535 | | D-23 | | 250.4751 | | 2952.496 | Spools, broken concrete | | rr ties, spools, concrete | RR, Spools, Concrete | | RR, Spools, Concrete | | mid-high | 4 | | 3 | | | 10.16152 | | 9.52353 | | Other / Mixed | |
| 92 | | 92 | | 183721.4 | | | 4 | | 22.72901 | | 519.126 | | 27.6974 | | D-25 | | 220.7521 | | 2549.617 | Spools, broken concrete | | rr ties, spools, concrete | RR, Spools, Concrete | | RR, Spools, Concrete | | mid-high | 4 | | 3 | | | 13.12118 | | 183.7214 | | Other / Mixed | |
| 93 | | 93 | | 219487.9 | | | 410.7964 | | 22.73045 | | 563.6271 | | 36.56656 | | d-26 big spool | | 262.3496 | | 2817.844 | Biggest spool with bouy and broken concrete | | spools, concrete | RR, Spools, Concrete | | RR, Spools, Concrete | | mid-high | 4 | | 3 | | | 13.29905 | | 219.4879 | | Other / Mixed | |
| 344 | | 344 | | 30254.61 | | | 7 | | 23.18702 | | 155.6589 | | 29.53746 | | D-20 | | 138.7362 | | 1006.296 | Spools, concrete railroad ties | | rr ties, spools, concrete | RR, Spools, Concrete | | RR, Spools, Concrete | | mid-high | 4 | | 3 | | | 11.3174 | | 30.25461 | | Other / Mixed | |
| 347 | | 347 | | 704.3783 | | | 21 | | 23.09736 | | 267.916 | | 46.93907 | | D-23 | | 250.4751 | | 2952.496 | Spools, broken concrete | | rr ties, spools, concrete | RR, Spools, Concrete | | RR, Spools, Concrete | | mid-high | 4 | | 3 | | | 7.557316 | | 0.704378 | | Other / Mixed | |
| 348 | | 348 | | 4343.889 | | | 2 | | 23.11756 | | 278.8044 | | 46.92964 | | D-23 | | 250.4751 | | 2952.496 | Spools, broken concrete | | rr ties, spools, concrete | RR, Spools, Concrete | | RR, Spools, Concrete | | mid-high | 4 | | 3 | | | 9.376525 | | 4.343889 | | Other / Mixed | |
| 391 | | 391 | | 66.23933 | | | 2 | | 22.63691 | | 604.5848 | | 26.67736 | | d-26 big spool | | 262.3496 | | 2817.844 | Biggest spool with bouy and broken concrete | | spools, concrete | RR, Spools, Concrete | | RR, Spools, Concrete | | mid-high | 4 | | 3 | | | 5.193274 | | 0.066239 | | Other / Mixed | |
| 398 | | 398 | | 600.2314 | | | 2 | | 22.6091 | | 596.6185 | | 56.92717 | | D-1 | | 261.2969 | | 3896.682 | Large Spool, broken ties, 15ft Spools | | rr ties, spools, concrete | RR, Spools, Concrete | | RR, Spools, Concrete | | mid-high | 4 | | 3 | | | 7.397315 | | 0.600231 | | Other / Mixed | |
| 408 | | 408 | | 11508.67 | | | 575 | | 23.07728 | | 307.7836 | | 53.54739 | | D-23 | | 250.4751 | | 2952.496 | Spools, broken concrete | | rr ties, spools, concrete | RR, Spools, Concrete | | RR, Spools, Concrete | | mid-high | 4 | | 3 | | | 10.35086 | | 11.50867 | | Other / Mixed | |
| 473 | | -1 | | 7548.062 | | | 1 | | 22.59202 | | 553.0525 | | 29.7301 | | D-1 | | 261.2969 | | 3896.682 | Large Spool, broken ties, 15ft Spools | | rr ties, spools, concrete | RR, Spools, Concrete | | RR, Spools, Concrete | | mid-high | 4 | | 3 | | | 9.929046 | | 7.548062 | | Other / Mixed | |
| 474 | | -1 | | 9998.864 | | | 1 | | 22.52082 | | 571.7994 | | 8.141447 | | D-1 | | 261.2969 | | 3896.682 | Large Spool, broken ties, 15ft Spools | | rr ties, spools, concrete | RR, Spools, Concrete | | RR, Spools, Concrete | | mid-high | 4 | | 3 | | | 10.21023 | | 9.998864 | | Other / Mixed | |
| 478 | | -1 | | 4601.3 | | | 1 | | 23.21698 | | 148.0925 | | 17.34442 | | D-20 | | 138.7362 | | 1006.296 | Spools, concrete railroad ties | | rr ties, spools, concrete | RR, Spools, Concrete | | RR, Spools, Concrete | | mid-high | 4 | | 3 | | | 9.434094 | | 4.6013 | | Other / Mixed | |
| 479 | | -1 | | 2023.941 | | | 1 | | 23.22989 | | 134.15 | | 21.22489 | | D-21 | | 172.7521 | | 1255.282 | Spools, concrete railroad ties | | rr ties, spools, concrete | RR, Spools, Concrete | | RR, Spools, Concrete | | mid-high | 4 | | 3 | | | 8.612802 | | 2.023941 | | Other / Mixed | |
| 481 | | -1 | | 238.7371 | | | 1 | | 22.97593 | | 511.5395 | | 38.43731 | | D-25 | | 220.7521 | | 2549.617 | Spools, broken concrete | | rr ties, spools, concrete | RR, Spools, Concrete | | RR, Spools, Concrete | | mid-high | 4 | | 3 | | | 6.475363 | | 0.238737 | | Other / Mixed | |
| 482 | | -1 | | 745.8772 | | | 1 | | 22.68789 | | 494.2614 | | 3.079969 | | D-25 | | 220.7521 | | 2549.617 | Spools, broken concrete | | rr ties, spools, concrete | RR, Spools, Concrete | | RR, Spools, Concrete | | mid-high | 4 | | 3 | | | 7.614561 | | 0.745877 | | Other / Mixed | |
| 625 | | -1 | | 471.4745 | | | 1 | | 23.08431 | | 265.5328 | | 23.75955 | | D-23 | | 250.4751 | | 2952.496 | Spools, broken concrete | | rr ties, spools, concrete | RR, Spools, Concrete | | RR, Spools, Concrete | | mid-high | 4 | | 3 | | | 7.155865 | | 0.471474 | | Other / Mixed | |
| 626 | | -1 | | 4301.992 | | | 1 | | 23.34468 | | 86.17786 | | 44.76548 | | D-20 | | 138.7362 | | 1006.296 | Spools, concrete railroad ties | | rr ties, spools, concrete | RR, Spools, Concrete | | RR, Spools, Concrete | | mid-high | 4 | | 3 | | | 9.366834 | | 4.301992 | | Other / Mixed | |
| 666 | | -1 | | 576.8209 | | | 1 | | 23.07759 | | 286.7624 | | 17.39202 | | D-23 | | 250.4751 | | 2952.496 | Spools, broken concrete | | rr ties, spools, concrete | RR, Spools, Concrete | | RR, Spools, Concrete | | mid-high | 4 | | 3 | | | 7.357532 | | 0.576821 | | Other / Mixed | |
| 667 | | -1 | | 121.8326 | | | 1 | | 22.9238 | | 308.5894 | | 23.3635 | | D-23 | | 250.4751 | | 2952.496 | Spools, broken concrete | | rr ties, spools, concrete | RR, Spools, Concrete | | RR, Spools, Concrete | | mid-high | 4 | | 3 | | | 5.802648 | | 0.121833 | | Other / Mixed | |
| 674 | | -1 | | 141.3196 | | | 1 | | 22.6584 | | 528.2085 | | 54.3789 | | d-26 big spool | | 262.3496 | | 2817.844 | Biggest spool with bouy and broken concrete | | spools, concrete | RR, Spools, Concrete | | RR, Spools, Concrete | | mid-high | 4 | | 3 | | | 5.951024 | | 0.14132 | | Other / Mixed | |
| 787 | | -1 | | 277.3657 | | | 14 | | 23.25336 | | 138.5313 | | 38.24493 | | D-20 | | 138.7362 | | 1006.296 | Spools, concrete railroad ties | | rr ties, spools, concrete | RR, Spools, Concrete | | RR, Spools, Concrete | | mid-high | 4 | | 3 | | | 6.625337 | | 0.277366 | | Other / Mixed | |
| 797 | | -1 | | 1471.866 | | | 74 | | 22.68694 | | 555.5987 | | 31.43537 | | d-26 big spool | | 262.3496 | | 2817.844 | Biggest spool with bouy and broken concrete | | spools, concrete | RR, Spools, Concrete | | RR, Spools, Concrete | | mid-high | 4 | | 3 | | | 8.294286 | | 1.471866 | | Other / Mixed | |
| 79 | | 79 | | 304538 | | | 14.16196 | | 22.7343 | | 391.2028 | | 19.27923 | | D-24 | | 255.682 | | 4174.679 | 18 ft spool,12-15 ft spools, concrete rubble, culverts | | rr ties, spools, concrete, culverts | RR, Spools, Concrete, Culvert | | RR, Spools, Culvert | | mid-high | 4 | | 4 | | | 13.62655 | | 304.538 | | Other / Mixed | |
| 91 | | 91 | | 776617.2 | | | 1178.175 | | 22.72128 | | 505.793 | | 14.44256 | | D-19 | | 248.3024 | | 2475.867 | 19 ft spool,12-15 ft spools, concrete rubble, culverts | | rr ties, spools, concrete, culverts | RR, Spools, Concrete, Culvert | | RR, Spools, Culvert | | mid-high | 4 | | 4 | | | 14.5627 | | 776.6172 | | Other / Mixed | |
| 402 | | 402 | | 2976.431 | | | 2 | | 22.81222 | | 432.4327 | | 94.58268 | | D-24 | | 255.682 | | 4174.679 | 18 ft spool,12-15 ft spools, concrete rubble, culverts | | rr ties, spools, concrete, culverts | RR, Spools, Concrete, Culvert | | RR, Spools, Culvert | | mid-high | 4 | | 4 | | | 8.99848 | | 2.976431 | | Other / Mixed | |
| 96 | | 96 | | 40392.35 | | | 12 | | 22.83759 | | 304.1569 | | 18.34791 | | D-4 | | 256.943 | | 3153.308 | Large Spool, ties, culverts | | rr ties, spools, culverts | RR, Spools, Culvert | | RR, Spools, Culvert | | mid-high | 4 | | 3 | | | 11.6064 | | 40.39235 | | Other / Mixed | |
| 97 | | 97 | | 6539.556 | | | 3 | | 22.81195 | | 271.9767 | | 39.85453 | | D-5 | | 288.2646 | | 3860.529 | Large Spool, ties, culverts | | rr ties, spools, culverts | RR, Spools, Culvert | | RR, Spools, Culvert | | mid-high | 4 | | 3 | | | 9.785625 | | 6.539556 | | Other / Mixed | |
| 243 | | 243 | | 2785.22 | | | 6 | | 21.61177 | | 802.6521 | | 10.3226 | | D-27 | | 444.7902 | | 7014.537 | Spools, culverts and railroad ties | | rr ties, spools, culverts | RR, Spools, Culvert | | RR, Spools, Culvert | | mid-high | 4 | | 3 | | | 8.932082 | | 2.78522 | | Other / Mixed | |
| 244 | | 244 | | 1548.57 | | | 3 | | 21.54255 | | 783.896 | | 31.41344 | | D-27 | | 444.7902 | | 7014.537 | Spools, culverts and railroad ties | | rr ties, spools, culverts | RR, Spools, Culvert | | RR, Spools, Culvert | | mid-high | 4 | | 3 | | | 8.345087 | | 1.54857 | | Other / Mixed | |
| 247 | | 247 | | 3375.075 | | | 4 | | 21.60652 | | 750.8618 | | 59.36213 | | D-27 | | 444.7902 | | 7014.537 | Spools, culverts and railroad ties | | rr ties, spools, culverts | RR, Spools, Culvert | | RR, Spools, Culvert | | mid-high | 4 | | 3 | | | 9.124173 | | 3.375075 | | Other / Mixed | |
| 354 | | 354 | | 10766.34 | | | 3 | | 22.86668 | | 312.1642 | | 18.82358 | | D-4 | | 256.943 | | 3153.308 | Large Spool, ties, culverts | | rr ties, spools, culverts | RR, Spools, Culvert | | RR, Spools, Culvert | | mid-high | 4 | | 3 | | | 10.28418 | | 10.76634 | | Other / Mixed | |
| 355 | | 355 | | 26999.84 | | | 3 | | 22.82067 | | 283.0495 | | 37.70788 | | D-4 | | 256.943 | | 3153.308 | Large Spool, ties, culverts | | rr ties, spools, culverts | RR, Spools, Culvert | | RR, Spools, Culvert | | mid-high | 4 | | 3 | | | 11.20359 | | 26.99984 | | Other / Mixed | |
| 356 | | 356 | | 309675 | | | 6 | | 22.91376 | | 250.7882 | | 33.48832 | | D-5 | | 288.2646 | | 3860.529 | Large Spool, ties, culverts | | rr ties, spools, culverts | RR, Spools, Culvert | | RR, Spools, Culvert | | mid-high | 4 | | 3 | | | 13.64328 | | 309.675 | | Other / Mixed | |
| 365 | | 365 | | 7289.725 | | | 2 | | 21.65602 | | 795.0786 | | 39.92881 | | D-27 | | 444.7902 | | 7014.537 | Spools, culverts and railroad ties | | rr ties, spools, culverts | RR, Spools, Culvert | | RR, Spools, Culvert | | mid-high | 4 | | 3 | | | 9.894221 | | 7.289725 | | Other / Mixed | |
| 486 | | -1 | | 9256.618 | | | 1 | | 22.92746 | | 193.6463 | | 46.94331 | | D-5 | | 288.2646 | | 3860.529 | Large Spool, ties, culverts | | rr ties, spools, culverts | RR, Spools, Culvert | | RR, Spools, Culvert | | mid-high | 4 | | 3 | | | 10.13309 | | 9.256618 | | Other / Mixed | |
| 487 | | -1 | | 361.7348 | | | 18.08674 | | 23.10555 | | 227.0114 | | 12.84489 | | D-5 | | 288.2646 | | 3860.529 | Large Spool, ties, culverts | | rr ties, spools, culverts | RR, Spools, Culvert | | RR, Spools, Culvert | | mid-high | 4 | | 3 | | | 6.890911 | | 0.361735 | | Other / Mixed | |
| 580 | | -1 | | 60.18487 | | | 1 | | 21.62123 | | 799.0341 | | 11.75325 | | D-27 | | 444.7902 | | 7014.537 | Spools, culverts and railroad ties | | rr ties, spools, culverts | RR, Spools, Culvert | | RR, Spools, Culvert | | mid-high | 4 | | 3 | | | 5.097421 | | 0.060185 | | Other / Mixed | |
| 669 | | -1 | | 2932.068 | | | 1 | | 22.7873 | | 348.4391 | | 46.07053 | | D-4 | | 256.943 | | 3153.308 | Large Spool, ties, culverts | | rr ties, spools, culverts | RR, Spools, Culvert | | RR, Spools, Culvert | | mid-high | 4 | | 3 | | | 8.983463 | | 2.932068 | | Other / Mixed | |
| 671 | | -1 | | 1545.355 | | | 1 | | 22.87031 | | 350.0652 | | 38.51041 | | D-4 | | 256.943 | | 3153.308 | Large Spool, ties, culverts | | rr ties, spools, culverts | RR, Spools, Culvert | | RR, Spools, Culvert | | mid-high | 4 | | 3 | | | 8.343009 | | 1.545355 | | Other / Mixed | |
| 717 | | -1 | | 3356.088 | | | 1 | | 21.58744 | | 817.4786 | | 47.64918 | | D-27 | | 444.7902 | | 7014.537 | Spools, culverts and railroad ties | | rr ties, spools, culverts | RR, Spools, Culvert | | RR, Spools, Culvert | | mid-high | 4 | | 3 | | | 9.118531 | | 3.356088 | | Other / Mixed | |
| 20 | | 20 | | 181790.6 | | | 7 | | 23.21015 | | 133.7235 | | 11.01211 | | 250-1 | | 122.8775 | | 1149.773 | 250 ton pile rr ties | | rr ties | 250T RR | | RR | | mid-high | 5 | | 1 | | | 13.11061 | | 181.7906 | | RR Ties | |
| 21 | | 21 | | 28770.44 | | | 12 | | 21.939 | | 129.1025 | | 13.13802 | | 250-10 | | 130.7157 | | 1119.483 | 250 ton pile rr ties | | rr ties | 250T RR | | RR | | mid-high | 5 | | 1 | | | 11.2671 | | 28.77044 | | RR Ties | |
| 22 | | 22 | | 5787.633 | | | 3 | | 21.84701 | | 129.8592 | | 22.35998 | | 250-11 | | 138.1907 | | 1185.466 | 250 ton pile rr ties | | rr ties | 250T RR | | RR | | mid-high | 5 | | 1 | | | 9.663479 | | 5.787633 | | RR Ties | |
| 23 | | 23 | | 16014.04 | | | 3 | | 21.97753 | | 125.7084 | | 3.103372 | | 250-11 | | 138.1907 | | 1185.466 | 250 ton pile rr ties | | rr ties | 250T RR | | RR | | mid-high | 5 | | 1 | | | 10.68122 | | 16.01404 | | RR Ties | |
| 24 | | 24 | | 26064.26 | | | 4 | | 22.92092 | | 218.5685 | | 6.509014 | | 250-18 | | 153.1538 | | 1383.906 | 250 ton pile rr ties | | rr ties | 250T RR | | RR | | mid-high | 5 | | 1 | | | 11.16832 | | 26.06426 | | RR Ties | |
| 25 | | 25 | | 4828.51 | | | 3 | | 23.01563 | | 250.4439 | | 15.11694 | | 250-19 | | 121.3319 | | 906.3614 | 250 ton pile rr ties | | rr ties | 250T RR | | RR | | mid-high | 5 | | 1 | | | 9.482293 | | 4.82851 | | RR Ties | |
| 26 | | 26 | | 71286.1 | | | 52.89061 | | 22.90683 | | 141.9355 | | 15.15357 | | 250-2 | | 159.7909 | | 1367.482 | 250 ton pile rr ties | | rr ties | 250T RR | | RR | | mid-high | 5 | | 1 | | | 12.17446 | | 71.2861 | | RR Ties | |
| 27 | | 27 | | 60133.39 | | | 8 | | 22.97002 | | 134.0655 | | 17.59871 | | 250-2 | | 159.7909 | | 1367.482 | 250 ton pile rr ties | | rr ties | 250T RR | | RR | | mid-high | 5 | | 1 | | | 12.00432 | | 60.13339 | | RR Ties | |
| 28 | | 28 | | 1258.24 | | | 36.67699 | | 22.78247 | | 224.0706 | | 9.796197 | | 250-20 | | 166.7401 | | 1588.134 | 250 ton pile rr ties | | rr ties | 250T RR | | RR | | mid-high | 5 | | 1 | | | 8.137469 | | 1.25824 | | RR Ties | |
| 29 | | 29 | | 11172.32 | | | 3 | | 23.32007 | | 153.8036 | | 22.82039 | | 250-21 | | 151.0718 | | 1285.526 | 250 ton pile rr ties | | rr ties | 250T RR | | RR | | mid-high | 5 | | 1 | | | 10.32119 | | 11.17232 | | RR Ties | |
| 30 | | 30 | | 6302.186 | | | 3 | | 23.31234 | | 140.9575 | | 35.9181 | | 250-21 | | 151.0718 | | 1285.526 | 250 ton pile rr ties | | rr ties | 250T RR | | RR | | mid-high | 5 | | 1 | | | 9.748652 | | 6.302186 | | RR Ties | |
| 31 | | 31 | | 6767.332 | | | 2 | | 23.15244 | | 183.297 | | 1.099965 | | 250-22 | | 133.9965 | | 866.7908 | 250 ton pile rr ties | | rr ties | 250T RR | | RR | | mid-high | 5 | | 1 | | | 9.819862 | | 6.767332 | | RR Ties | |
| 32 | | 32 | | 44985.29 | | | 14 | | 22.93328 | | 131.8941 | | 13.01067 | | 250-3 | | 162.7097 | | 1201.128 | 250 ton pile rr ties | | rr ties | 250T RR | | RR | | mid-high | 5 | | 1 | | | 11.71409 | | 44.98529 | | RR Ties | |
| 33 | | 33 | | 58447.84 | | | 8 | | 22.94398 | | 130.5352 | | 10.27823 | | 250-3 | | 162.7097 | | 1201.128 | 250 ton pile rr ties | | rr ties | 250T RR | | RR | | mid-high | 5 | | 1 | | | 11.97589 | | 58.44784 | | RR Ties | |
| 34 | | 34 | | 55941.77 | | | 6 | | 22.50413 | | 105.4182 | | 34.6032 | | 250-5 | | 136.9088 | | 1228.846 | 250 ton pile rr ties | | rr ties | 250T RR | | RR | | mid-high | 5 | | 1 | | | 11.93207 | | 55.94177 | | RR Ties | |
| 35 | | 35 | | 210773.2 | | | 31 | | 22.67122 | | 139.9592 | | 2.932388 | | 250-5 | | 136.9088 | | 1228.846 | 250 ton pile rr ties | | rr ties | 250T RR | | RR | | mid-high | 5 | | 1 | | | 13.25854 | | 210.7732 | | RR Ties | |
| 36 | | 36 | | 60745.73 | | | 17 | | 22.56907 | | 127.6193 | | 6.695613 | | 250-6 | | 128.1174 | | 1065.288 | 250 ton pile rr ties | | rr ties | 250T RR | | RR | | mid-high | 5 | | 1 | | | 12.01445 | | 60.74573 | | RR Ties | |
| 37 | | 37 | | 10783.89 | | | 3 | | 22.35392 | | 110.6031 | | 6.269274 | | 250-7 | | 150.2889 | | 1192.758 | 250 ton pile rr ties | | rr ties | 250T RR | | RR | | mid-high | 5 | | 1 | | | 10.28581 | | 10.78389 | | RR Ties | |
| 38 | | 38 | | 31100.93 | | | 7 | | 22.51374 | | 120.5221 | | 4.289882 | | 250-8 | | 132.6598 | | 1147.094 | 250 ton pile rr ties | | rr ties | 250T RR | | RR | | mid-high | 5 | | 1 | | | 11.34499 | | 31.10093 | | RR Ties | |
| 65 | | 65 | | 19424.94 | | | 6 | | 23.12734 | | 219.0513 | | 27.11336 | | 250-19 | | 121.3319 | | 906.3614 | 250 ton pile rr ties | | rr ties | 250T RR | | RR | | mid-high | 5 | | 1 | | | 10.87431 | | 19.42494 | | RR Ties | |
| 66 | | 66 | | 14364.25 | | | 4 | | 23.35354 | | 148.4691 | | 30.90153 | | 250-21 | | 151.0718 | | 1285.526 | 250 ton pile rr ties | | rr ties | 250T RR | | RR | | mid-high | 5 | | 1 | | | 10.5725 | | 14.36425 | | RR Ties | |
| 67 | | 67 | | 489.5679 | | | 12.25273 | | 23.32252 | | 171.242 | | 6.904794 | | 250-21 | | 151.0718 | | 1285.526 | 250 ton pile rr ties | | rr ties | 250T RR | | RR | | mid-high | 5 | | 1 | | | 7.193523 | | 0.489568 | | RR Ties | |
| 264 | | 264 | | 253178.9 | | | 28 | | 22.81022 | | 232.9726 | | 21.82994 | | 250-20 | | 166.7401 | | 1588.134 | 250 ton pile rr ties | | rr ties | 250T RR | | RR | | mid-high | 5 | | 1 | | | 13.44185 | | 253.1789 | | RR Ties | |
| 265 | | 265 | | 20537.41 | | | 6 | | 22.79143 | | 237.3534 | | 13.19528 | | 250-20 | | 166.7401 | | 1588.134 | 250 ton pile rr ties | | rr ties | 250T RR | | RR | | mid-high | 5 | | 1 | | | 10.93 | | 20.53741 | | RR Ties | |
| 266 | | 266 | | 3496.812 | | | 2 | | 22.80235 | | 243.9664 | | 41.476 | | 250-20 | | 166.7401 | | 1588.134 | 250 ton pile rr ties | | rr ties | 250T RR | | RR | | mid-high | 5 | | 1 | | | 9.159607 | | 3.496812 | | RR Ties | |
| 268 | | 268 | | 13469.05 | | | 10 | | 23.0109 | | 262.7005 | | 36.18055 | | 250-17 | | 121.0168 | | 997.5653 | 250 ton pile rr ties | | rr ties | 250T RR | | RR | | mid-high | 5 | | 1 | | | 10.50815 | | 13.46905 | | RR Ties | |
| 303 | | 303 | | 15932.34 | | | 2 | | 23.15488 | | 106.5856 | | 37.3821 | | 250-1 | | 122.8775 | | 1149.773 | 250 ton pile rr ties | | rr ties | 250T RR | | RR | | mid-high | 5 | | 1 | | | 10.67611 | | 15.93234 | | RR Ties | |
| 304 | | 304 | | 29646 | | | 5 | | 23.03497 | | 115.8143 | | 31.84418 | | 250-2 | | 159.7909 | | 1367.482 | 250 ton pile rr ties | | rr ties | 250T RR | | RR | | mid-high | 5 | | 1 | | | 11.29708 | | 29.646 | | RR Ties | |
| 305 | | 305 | | 20392.41 | | | 2 | | 23.04475 | | 101.8777 | | 47.60974 | | 250-2 | | 159.7909 | | 1367.482 | 250 ton pile rr ties | | rr ties | 250T RR | | RR | | mid-high | 5 | | 1 | | | 10.92292 | | 20.39241 | | RR Ties | |
| 307 | | 307 | | 3501.825 | | | 2 | | 23.07611 | | 137.6863 | | 32.86575 | | 250-1 | | 122.8775 | | 1149.773 | 250 ton pile rr ties | | rr ties | 250T RR | | RR | | mid-high | 5 | | 1 | | | 9.16104 | | 3.501825 | | RR Ties | |
| 308 | | 308 | | 32906.64 | | | 13 | | 23.25526 | | 103.8307 | | 26.62662 | | 250-1 | | 122.8775 | | 1149.773 | 250 ton pile rr ties | | rr ties | 250T RR | | RR | | mid-high | 5 | | 1 | | | 11.40143 | | 32.90664 | | RR Ties | |
| 309 | | 309 | | 18079.51 | | | 8 | | 23.1377 | | 126.0964 | | 30.06709 | | 250-1 | | 122.8775 | | 1149.773 | 250 ton pile rr ties | | rr ties | 250T RR | | RR | | mid-high | 5 | | 1 | | | 10.80253 | | 18.07951 | | RR Ties | |
| 310 | | 310 | | 3917.706 | | | 3 | | 22.73685 | | 117.6711 | | 39.0826 | | 250-3 | | 162.7097 | | 1201.128 | 250 ton pile rr ties | | rr ties | 250T RR | | RR | | mid-high | 5 | | 1 | | | 9.273262 | | 3.917706 | | RR Ties | |
| 311 | | 311 | | 16074.02 | | | 5 | | 22.90861 | | 137.261 | | 21.09284 | | 250-3 | | 162.7097 | | 1201.128 | 250 ton pile rr ties | | rr ties | 250T RR | | RR | | mid-high | 5 | | 1 | | | 10.68496 | | 16.07402 | | RR Ties | |
| 312 | | 312 | | 7907.146 | | | 4 | | 22.56973 | | 133.2138 | | 34.60809 | | 250-4 | | 125.7751 | | 1127.217 | 250 ton pile rr ties | | rr ties | 250T RR | | RR | | mid-high | 5 | | 1 | | | 9.975522 | | 7.907146 | | RR Ties | |
| 313 | | 313 | | 17212.33 | | | 3 | | 22.5329 | | 98.3824 | | 28.02988 | | 250-6 | | 128.1174 | | 1065.288 | 250 ton pile rr ties | | rr ties | 250T RR | | RR | | mid-high | 5 | | 1 | | | 10.75338 | | 17.21233 | | RR Ties | |
| 314 | | 314 | | 23616.09 | | | 3 | | 22.39396 | | 132.5915 | | 33.73149 | | 250-6 | | 128.1174 | | 1065.288 | 250 ton pile rr ties | | rr ties | 250T RR | | RR | | mid-high | 5 | | 1 | | | 11.06968 | | 23.61609 | | RR Ties | |
| 381 | | 381 | | 3454.783 | | | 4 | | 21.60342 | | 122.7611 | | 23.70666 | | 250-10 | | 145.9795 | | 1013.348 | 250 ton pile rr ties | | rr ties | 250T RR | | RR | | mid-high | 5 | | 1 | | | 9.147515 | | 3.454783 | | RR Ties | |
| 382 | | 382 | | 119912.2 | | | 59 | | 23.28833 | | 57.97038 | | 72.89031 | | 250-1 | | 122.8775 | | 1149.773 | 250 ton pile rr ties | | rr ties | 250T RR | | RR | | mid-high | 5 | | 1 | | | 12.69452 | | 119.9122 | | RR Ties | |
| 383 | | 383 | | 33199.4 | | | 22 | | 23.30464 | | 46.18629 | | 96.96962 | | 250-1 | | 122.8775 | | 1149.773 | 250 ton pile rr ties | | rr ties | 250T RR | | RR | | mid-high | 5 | | 1 | | | 11.41029 | | 33.1994 | | RR Ties | |
| 385 | | 385 | | 11454.09 | | | 4 | | 23.09318 | | 115.3361 | | 42.21555 | | 250-1 | | 122.8775 | | 1149.773 | 250 ton pile rr ties | | rr ties | 250T RR | | RR | | mid-high | 5 | | 1 | | | 10.3461 | | 11.45409 | | RR Ties | |
| 386 | | 386 | | 184447.3 | | | 14 | | 22.95213 | | 173.0394 | | 27.21352 | | 250-2 | | 159.7909 | | 1367.482 | 250 ton pile rr ties | | rr ties | 250T RR | | RR | | mid-high | 5 | | 1 | | | 13.12512 | | 184.4473 | | RR Ties | |
| 393 | | 393 | | 1287.231 | | | 2 | | 22.16194 | | 60.04794 | | 76.9144 | | 250-8 | | 132.6598 | | 1147.094 | 250 ton pile rr ties | | rr ties | 250T RR | | RR | | mid-high | 5 | | 1 | | | 8.160249 | | 1.287231 | | RR Ties | |
| 424 | | -1 | | 4251.068 | | | 1 | | 22.60158 | | 212.6093 | | 72.95519 | | 250-20 | | 166.7401 | | 1588.134 | 250 ton pile rr ties | | rr ties | 250T RR | | RR | | mid-high | 5 | | 1 | | | 9.354926 | | 4.251068 | | RR Ties | |
| 427 | | -1 | | 352.8901 | | | 1 | | 21.75984 | | 122.8414 | | 10.04013 | | 250-13 | | 151.5281 | | 1422.905 | 250 ton pile rr ties | | rr ties | 250T RR | | RR | | mid-high | 5 | | 1 | | | 6.866157 | | 0.35289 | | RR Ties | |
| 428 | | -1 | | 1346.234 | | | 1 | | 23.09016 | | 240.1714 | | 4.131585 | | 250-19 | | 121.3319 | | 906.3614 | 250 ton pile rr ties | | rr ties | 250T RR | | RR | | mid-high | 5 | | 1 | | | 8.205066 | | 1.346234 | | RR Ties | |
| 458 | | -1 | | 995.1693 | | | 1 | | 23.36071 | | 177.6183 | | 9.202546 | | 250-21 | | 151.0718 | | 1285.526 | 250 ton pile rr ties | | rr ties | 250T RR | | RR | | mid-high | 5 | | 1 | | | 7.902913 | | 0.995169 | | RR Ties | |
| 590 | | -1 | | 444.9689 | | | 1 | | 22.61756 | | 223.3262 | | 71.70988 | | 250-20 | | 166.7401 | | 1588.134 | 250 ton pile rr ties | | rr ties | 250T RR | | RR | | mid-high | 5 | | 1 | | | 7.098004 | | 0.444969 | | RR Ties | |
| 591 | | -1 | | 181.5644 | | | 1 | | 22.9784 | | 265.0885 | | 50.64982 | | 250-17 | | 121.0168 | | 997.5653 | 250 ton pile rr ties | | rr ties | 250T RR | | RR | | mid-high | 5 | | 1 | | | 6.20161 | | 0.181564 | | RR Ties | |
| 596 | | -1 | | 838.1455 | | | 1 | | 22.98399 | | 239.6683 | | 25.95145 | | 250-18 | | 153.1538 | | 1383.906 | 250 ton pile rr ties | | rr ties | 250T RR | | RR | | mid-high | 5 | | 1 | | | 7.731192 | | 0.838146 | | RR Ties | |
| 597 | | -1 | | 9273.106 | | | 1 | | 22.98375 | | 256.5687 | | 37.99927 | | 250-17 | | 121.0168 | | 997.5653 | 250 ton pile rr ties | | rr ties | 250T RR | | RR | | mid-high | 5 | | 1 | | | 10.13487 | | 9.273106 | | RR Ties | |
| 603 | | -1 | | 2441.9 | | | 1 | | 23.12214 | | 180.1262 | | 19.56094 | | 250-22 | | 133.9965 | | 866.7908 | 250 ton pile rr ties | | rr ties | 250T RR | | RR | | mid-high | 5 | | 1 | | | 8.800532 | | 2.4419 | | RR Ties | |
| 628 | | -1 | | 247.2276 | | | 1 | | 22.94484 | | 111.6881 | | 27.13483 | | 250-3 | | 162.7097 | | 1201.128 | 250 ton pile rr ties | | rr ties | 250T RR | | RR | | mid-high | 5 | | 1 | | | 6.510309 | | 0.247228 | | RR Ties | |
| 629 | | -1 | | 1153.079 | | | 1 | | 23.20501 | | 108.8432 | | 25.89145 | | 250-1 | | 122.8775 | | 1149.773 | 250 ton pile rr ties | | rr ties | 250T RR | | RR | | mid-high | 5 | | 1 | | | 8.050191 | | 1.153079 | | RR Ties | |
| 630 | | -1 | | 1480.307 | | | 1 | | 22.50349 | | 145.2009 | | 35.75534 | | 250-5 | | 136.9088 | | 1228.846 | 250 ton pile rr ties | | rr ties | 250T RR | | RR | | mid-high | 5 | | 1 | | | 8.300005 | | 1.480307 | | RR Ties | |
| 631 | | -1 | | 2807.416 | | | 1 | | 22.56769 | | 105.6798 | | 24.12807 | | 250-6 | | 128.1174 | | 1065.288 | 250 ton pile rr ties | | rr ties | 250T RR | | RR | | mid-high | 5 | | 1 | | | 8.94002 | | 2.807416 | | RR Ties | |
| 632 | | -1 | | 24480.29 | | | 1 | | 22.50906 | | 102.0286 | | 32.98854 | | 250-6 | | 128.1174 | | 1065.288 | 250 ton pile rr ties | | rr ties | 250T RR | | RR | | mid-high | 5 | | 1 | | | 11.10562 | | 24.48029 | | RR Ties | |
| 633 | | -1 | | 465.4652 | | | 1 | | 22.58874 | | 124.8241 | | 38.45744 | | 250-5 | | 136.9088 | | 1228.846 | 250 ton pile rr ties | | rr ties | 250T RR | | RR | | mid-high | 5 | | 1 | | | 7.143037 | | 0.465465 | | RR Ties | |
| 634 | | -1 | | 3902.027 | | | 1 | | 22.66361 | | 138.6185 | | 23.21618 | | 250-5 | | 136.9088 | | 1228.846 | 250 ton pile rr ties | | rr ties | 250T RR | | RR | | mid-high | 5 | | 1 | | | 9.269252 | | 3.902027 | | RR Ties | |
| 635 | | -1 | | 1460.947 | | | 1 | | 22.57326 | | 128.7042 | | 18.73815 | | 250-5 | | 136.9088 | | 1228.846 | 250 ton pile rr ties | | rr ties | 250T RR | | RR | | mid-high | 5 | | 1 | | | 8.28684 | | 1.460947 | | RR Ties | |
| 684 | | -1 | | 778.1299 | | | 1 | | 22.97541 | | 245.4152 | | 23.12014 | | 250-18 | | 153.1538 | | 1383.906 | 250 ton pile rr ties | | rr ties | 250T RR | | RR | | mid-high | 5 | | 1 | | | 7.656893 | | 0.77813 | | RR Ties | |
| 685 | | -1 | | 7928.627 | | | 1 | | 22.77667 | | 202.7275 | | 33.59637 | | 250-20 | | 166.7401 | | 1588.134 | 250 ton pile rr ties | | rr ties | 250T RR | | RR | | mid-high | 5 | | 1 | | | 9.978235 | | 7.928627 | | RR Ties | |
| 686 | | -1 | | 2166.806 | | | 1 | | 23.2 | | 147.8686 | | 84.05834 | | 250-21 | | 151.0718 | | 1285.526 | 250 ton pile rr ties | | rr ties | 250T RR | | RR | | mid-high | 5 | | 1 | | | 8.681009 | | 2.166806 | | RR Ties | |
| 696 | | -1 | | 1273.187 | | | 1 | | 21.91693 | | 123.1293 | | 33.45727 | | 250-9 | | 152.07 | | 1320.464 | 250 ton pile rr ties | | rr ties | 250T RR | | RR | | mid-high | 5 | | 1 | | | 8.149278 | | 1.273187 | | RR Ties | |
| 697 | | -1 | | 1198.372 | | | 1 | | 22.18955 | | 115.8716 | | 19.98978 | | 250-7 | | 150.2889 | | 1192.758 | 250 ton pile rr ties | | rr ties | 250T RR | | RR | | mid-high | 5 | | 1 | | | 8.088719 | | 1.198372 | | RR Ties | |
| 698 | | -1 | | 731.3095 | | | 1 | | 22.8893 | | 145.6176 | | 32.78727 | | 250-2 | | 159.7909 | | 1367.482 | 250 ton pile rr ties | | rr ties | 250T RR | | RR | | mid-high | 5 | | 1 | | | 7.594837 | | 0.731309 | | RR Ties | |
| 699 | | -1 | | 1634.287 | | | 1 | | 23.00631 | | 144.9194 | | 41.03401 | | 250-1 | | 122.8775 | | 1149.773 | 250 ton pile rr ties | | rr ties | 250T RR | | RR | | mid-high | 5 | | 1 | | | 8.398962 | | 1.634287 | | RR Ties | |
| 13 | | 13 | | 285451.5 | | | 2236.029 | | 21.67068 | | 126.0782 | | 22.3209 | | 500T_1 | | 125.491 | | 948.1763 | 500 rr ties | | rr ties | 500T RR | | RR | | high | 5 | | 1 | | | 13.56183 | | 285.4515 | | RR Ties | |
| 56 | | 56 | | 19841.12 | | | 682.7297 | | 21.09664 | | 324.672 | | 21.41638 | | 500T_2 | | 131.5586 | | 1143.481 | 500 rr ties | | rr ties | 500T RR | | RR | | high | 5 | | 1 | | | 10.89551 | | 19.84112 | | RR Ties | |
| 57 | | 57 | | 27765.6 | | | 16 | | 21.03711 | | 357.8143 | | 14.52273 | | 500T_2 | | 131.5586 | | 1143.481 | 500 rr ties | | rr ties | 500T RR | | RR | | high | 5 | | 1 | | | 11.23155 | | 27.7656 | | RR Ties | |
| 58 | | 58 | | 6713.721 | | | 4 | | 21.07002 | | 341.0857 | | 16.41913 | | 500T_2 | | 131.5586 | | 1143.481 | 500 rr ties | | rr ties | 500T RR | | RR | | high | 5 | | 1 | | | 9.811909 | | 6.713721 | | RR Ties | |
| 295 | | 295 | | 15557.68 | | | 2 | | 21.39906 | | 10.56114 | | 100.0338 | | 500T_1 | | 125.491 | | 948.1763 | 500 rr ties | | rr ties | 500T RR | | RR | | high | 5 | | 1 | | | 10.65231 | | 15.55768 | | RR Ties | |
| 452 | | -1 | | 959.9353 | | | 1 | | 21.21085 | | 342.2794 | | 2.243922 | | 500T_2 | | 131.5586 | | 1143.481 | 500 rr ties | | rr ties | 500T RR | | RR | | high | 5 | | 1 | | | 7.866866 | | 0.959935 | | RR Ties | |
| 770 | | -1 | | 78.57851 | | | 4 | | 20.69308 | | 277.9492 | | 66.53283 | | 500T_2 | | 131.5586 | | 1143.481 | 500 rr ties | | rr ties | 500T RR | | RR | | high | 5 | | 1 | | | 5.364098 | | 0.078579 | | RR Ties | |
| 99 | | 99 | | 17847.29 | | | 8 | | 22.38785 | | 747.6611 | | 16.48791 | | EMR Capt Berry | | 62.61292 | | 214.5414 | boat | | boat | Boats | | Boats | | high | 5 | | 1 | | | 10.78961 | | 17.84729 | | Boat | |
| 143 | | 143 | | 222395.5 | | | 634.2925 | | 23.23702 | | 116.3917 | | 27.63655 | | Murray Meg | | 62.81063 | | 220.6961 | boat | | boat | Boats | | Boats | | high | 5 | | 1 | | | 13.31221 | | 222.3955 | | Boat | |
| 248 | | 248 | | 1063.55 | | | 2 | | 21.94016 | | 790.6325 | | 71.01006 | | EMR Capt Berry | | 62.61292 | | 214.5414 | boat | | boat | Boats | | Boats | | high | 5 | | 1 | | | 7.969368 | | 1.06355 | | Boat | |
| 272 | | 272 | | 74096.56 | | | 2 | | 23.2949 | | 26.85115 | | 110.5476 | | Murray Meg | | 62.81063 | | 220.6961 | boat | | boat | Boats | | Boats | | high | 5 | | 1 | | | 12.21312 | | 74.09656 | | Boat | |
| 361 | | 361 | | 1223.643 | | | 5 | | 21.99238 | | 729.2064 | | 64.26487 | | EMR Capt Berry | | 62.61292 | | 214.5414 | boat | | boat | Boats | | Boats | | high | 5 | | 1 | | | 8.109588 | | 1.223643 | | Boat | |
| 598 | | -1 | | 177.0156 | | | 1 | | 23.12074 | | 116.0204 | | 21.25982 | | Murray Meg | | 62.81063 | | 220.6961 | boat | | boat | Boats | | Boats | | high | 5 | | 1 | | | 6.176238 | | 0.177016 | | Boat | |
| 54 | | 54 | | 19842.91 | | | 2 | | 22.98312 | | 280.2725 | | 34.76531 | | CCA Corner | | 419.932 | | 5633.222 | 100 tons rr ties in line | | CCA rr tie ridge | CCA Ridge | | RR | | mid-high | 5 | | 1 | | | 10.8956 | | 19.84291 | | RR Ties | |
| 459 | | -1 | | 1679.617 | | | 1 | | 22.97884 | | 249.3217 | | 24.74077 | | CCA Corner | | 419.932 | | 5633.222 | 100 tons rr ties in line | | CCA rr tie ridge | CCA Ridge | | RR | | mid-high | 5 | | 1 | | | 8.426321 | | 1.679617 | | RR Ties | |
| 460 | | -1 | | 5584.391 | | | 1 | | 22.90131 | | 273.3181 | | 19.23125 | | CCA Corner | | 419.932 | | 5633.222 | 100 tons rr ties in line | | CCA rr tie ridge | CCA Ridge | | RR | | mid-high | 5 | | 1 | | | 9.627731 | | 5.584391 | | RR Ties | |
| 125 | | 125 | | 12536.85 | | | 3 | | 21.22549 | | 274.3234 | | 15.32968 | | Extra rr ties 1 | | 41.25185 | | 75.35726 | rr ties | | rr ties | RR | | RR | | mid | 5 | | 1 | | | 10.43643 | | 12.53685 | | RR Ties | |
| 404 | | 404 | | 2660.932 | | | 3 | | 21.22028 | | 309.866 | | 46.87788 | | Extra rr ties 1 | | 41.25185 | | 75.35726 | rr ties | | rr ties | RR | | RR | | mid | 5 | | 1 | | | 8.886432 | | 2.660932 | | RR Ties | |
| 497 | | -1 | | 1288.349 | | | 1 | | 21.15919 | | 261.3502 | | 15.97445 | | Extra rr ties 1 | | 41.25185 | | 75.35726 | rr ties | | rr ties | RR | | RR | | mid | 5 | | 1 | | | 8.161117 | | 1.288349 | | RR Ties | |
| 719 | | -1 | | 497.703 | | | 1 | | 21.1754 | | 1020.43 | | 29.24664 | | Extra rr ties 2 | | 116.3223 | | 547.1535 | rr ties | | rr ties | RR | | RR | | mid | 5 | | 1 | | | 7.210004 | | 0.497703 | | RR Ties | |
| 734 | | -1 | | 1323.202 | | | 1 | | 21.28253 | | 310.016 | | 40.64092 | | Extra rr ties 1 | | 41.25185 | | 75.35726 | rr ties | | rr ties | RR | | RR | | mid | 5 | | 1 | | | 8.187809 | | 1.323202 | | RR Ties | |
| 2 | | 2 | | 4812.063 | | | 2 | | 21.62862 | | 609.9863 | | 8.810733 | | 100tonmixed13 | | 99.21022 | | 447.8839 | 75 ton ties, 25 tons broken concrete | | rr ties, concrete | RR, Concrete | | RR, Concrete | | mid | 5 | | 2 | | | 9.478881 | | 4.812063 | | RR Ties | |
| 3 | | 3 | | 23097.23 | | | 48.20445 | | 21.61459 | | 604.0036 | | 7.659475 | | 100tonmixed14 | | 125.5555 | | 644.8692 | 75 ton ties, 25 tons broken concrete | | rr ties, concrete | RR, Concrete | | RR, Concrete | | mid | 5 | | 2 | | | 11.04747 | | 23.09723 | | RR Ties | |
| 4 | | 4 | | 7919.958 | | | 4 | | 21.68665 | | 568.2499 | | 2.616636 | | 100tonmixed15 | | 101.2989 | | 324.5632 | 75 ton ties, 25 tons broken concrete | | rr ties, concrete | RR, Concrete | | RR, Concrete | | mid | 5 | | 2 | | | 9.977141 | | 7.919958 | | RR Ties | |
| 5 | | 5 | | 6296.307 | | | 2 | | 21.68946 | | 553.2343 | | 11.58282 | | 100tonmixed17 | | 96.75769 | | 444.9959 | 75 ton ties, 25 tons broken concrete | | rr ties, concrete | RR, Concrete | | RR, Concrete | | mid | 5 | | 2 | | | 9.747719 | | 6.296307 | | RR Ties | |
| 6 | | 6 | | 223638.7 | | | 163.4141 | | 21.57223 | | 545.8807 | | 14.15298 | | 100tonmixed18 | | 94.66111 | | 473.3035 | 75 ton ties, 25 tons broken concrete | | rr ties, concrete | RR, Concrete | | RR, Concrete | | mid | 5 | | 2 | | | 13.31779 | | 223.6387 | | RR Ties | |
| 7 | | 7 | | 32508.72 | | | 12 | | 21.44508 | | 527.7536 | | 17.02696 | | 100tonmixed17 | | 96.75769 | | 444.9959 | 75 ton ties, 25 tons broken concrete | | rr ties, concrete | RR, Concrete | | RR, Concrete | | mid | 5 | | 2 | | | 11.38926 | | 32.50872 | | RR Ties | |
| 8 | | 8 | | 6596.446 | | | 4 | | 21.52578 | | 524.4203 | | 11.15952 | | 100tonmixed18 | | 94.66111 | | 473.3035 | 75 ton ties, 25 tons broken concrete | | rr ties, concrete | RR, Concrete | | RR, Concrete | | mid | 5 | | 2 | | | 9.794286 | | 6.596446 | | RR Ties | |
| 9 | | 9 | | 4533.372 | | | 3 | | 22.02869 | | 272.3282 | | 4.606572 | | 100tonmixed20 | | 71.53361 | | 216.3191 | 75 ton ties, 25 tons broken concrete | | rr ties, concrete | RR, Concrete | | RR, Concrete | | mid | 5 | | 2 | | | 9.419221 | | 4.533372 | | RR Ties | |
| 14 | | 14 | | 19711.26 | | | 33.22878 | | 21.10933 | | 761.9293 | | 17.63702 | | 100tonmixed5 | | 89.59821 | | 402.0472 | 75 ton ties, 25 tons broken concrete | | rr ties, concrete | RR, Concrete | | RR, Concrete | | mid | 5 | | 2 | | | 10.88895 | | 19.71126 | | RR Ties | |
| 15 | | 15 | | 13430.28 | | | 10 | | 21.20124 | | 758.2351 | | 19.86189 | | 100tonmixed6 | | 87.71188 | | 448.186 | 75 ton ties, 25 tons broken concrete | | rr ties, concrete | RR, Concrete | | RR, Concrete | | mid | 5 | | 2 | | | 10.50527 | | 13.43028 | | RR Ties | |
| 377 | | 377 | | 8192.462 | | | 2 | | 22.57499 | | 276.3703 | | 18.41133 | | 100tonmixed23 | | 116.1284 | | 811.0029 | 75 ton ties, 25 tons broken concrete | | rr ties, concrete | RR, Concrete | | RR, Concrete | | mid | 5 | | 2 | | | 10.01097 | | 8.192462 | | RR Ties | |
| 396 | | 396 | | 501.3155 | | | 3 | | 21.11876 | | 748.9786 | | 13.89781 | | 100tonmixed4 | | 96.58254 | | 448.4271 | 75 ton ties, 25 tons broken concrete | | rr ties, concrete | RR, Concrete | | RR, Concrete | | mid | 5 | | 2 | | | 7.217236 | | 0.501315 | | RR Ties | |
| 403 | | 403 | | 1141.181 | | | 18 | | 22.01924 | | 309.3127 | | 35.58981 | | 100tonmixed22 | | 100.228 | | 522.934 | 75 ton ties, 25 tons broken concrete | | rr ties, concrete | RR, Concrete | | RR, Concrete | | mid | 5 | | 2 | | | 8.039819 | | 1.141181 | | RR Ties | |
| 412 | | -1 | | 7403.625 | | | 370.1813 | | 21.61917 | | 812.3124 | | 30.55388 | | 100tonmixed12 | | 87.23127 | | 440.0661 | 75 ton ties, 25 tons broken concrete | | rr ties, concrete | RR, Concrete | | RR, Concrete | | mid | 5 | | 2 | | | 9.909725 | | 7.403625 | | RR Ties | |
| 413 | | -1 | | 2857.363 | | | 142.8681 | | 21.6306 | | 535.1956 | | 7.163609 | | 100tonmixed17 | | 96.75769 | | 444.9959 | 75 ton ties, 25 tons broken concrete | | rr ties, concrete | RR, Concrete | | RR, Concrete | | mid | 5 | | 2 | | | 8.957654 | | 2.857363 | | RR Ties | |
| 652 | | -1 | | 2932.429 | | | 1 | | 20.89019 | | 831.6492 | | 87.9733 | | 100tonmixed5 | | 89.59821 | | 402.0472 | 75 ton ties, 25 tons broken concrete | | rr ties, concrete | RR, Concrete | | RR, Concrete | | mid | 5 | | 2 | | | 8.983586 | | 2.932429 | | RR Ties | |
| 710 | | -1 | | 279.0213 | | | 1 | | 21.3541 | | 728.4178 | | 39.4676 | | 100tonmixed7 | | 73.86209 | | 330.634 | 75 ton ties, 25 tons broken concrete | | rr ties, concrete | RR, Concrete | | RR, Concrete | | mid | 5 | | 2 | | | 6.631288 | | 0.279021 | | RR Ties | |
| 10 | | 10 | | 13925.38 | | | 3 | | 23.09474 | | 171.498 | | 19.32503 | | Mix_40 | | 107.8763 | | 495.4374 | 25 ton RR Ties, 25 ton Concrete, 6 Pallets CinderBlock | | rr ties, concrete, cinderblocks | RR, Concrete, CB | | RR, Concrete, CB | | mid | 5 | | 3 | | | 10.54147 | | 13.92538 | | RR Ties | |
| 109 | | 109 | | 22559.69 | | | 261.394 | | 20.90285 | | 253.4362 | | 5.074879 | | Mix_1 | | 54.124 | | 166.0747 | 25 ton RR Ties, 25 ton Concrete, 6 Pallets CinderBlock | | rr ties, concrete, cinderblocks | RR, Concrete, CB | | RR, Concrete, CB | | mid | 5 | | 3 | | | 11.02392 | | 22.55969 | | RR Ties | |
| 110 | | 110 | | 4311.209 | | | 4 | | 22.32819 | | 216.7143 | | 26.14707 | | Mix_11 | | 100.0615 | | 513.0493 | 25 ton RR Ties, 25 ton Concrete, 6 Pallets CinderBlock | | rr ties, concrete, cinderblocks | RR, Concrete, CB | | RR, Concrete, CB | | mid | 5 | | 3 | | | 9.368974 | | 4.311209 | | RR Ties | |
| 111 | | 111 | | 8473.773 | | | 4 | | 22.59768 | | 199.8503 | | 2.621263 | | Mix_13 | | 65.66873 | | 184.2514 | 25 ton RR Ties, 25 ton Concrete, 6 Pallets CinderBlock | | rr ties, concrete, cinderblocks | RR, Concrete, CB | | RR, Concrete, CB | | mid | 5 | | 3 | | | 10.04473 | | 8.473773 | | RR Ties | |
| 112 | | 112 | | 7899.597 | | | 6 | | 22.75607 | | 203.3164 | | 1.487723 | | Mix_14 | | 98.57902 | | 411.8953 | 25 ton RR Ties, 25 ton Concrete, 6 Pallets CinderBlock | | rr ties, concrete, cinderblocks | RR, Concrete, CB | | RR, Concrete, CB | | mid | 5 | | 3 | | | 9.974567 | | 7.899597 | | RR Ties | |
| 113 | | 113 | | 2086.339 | | | 2 | | 22.74004 | | 202.0397 | | 22.29491 | | Mix_14 | | 98.57902 | | 411.8953 | 25 ton RR Ties, 25 ton Concrete, 6 Pallets CinderBlock | | rr ties, concrete, cinderblocks | RR, Concrete, CB | | RR, Concrete, CB | | mid | 5 | | 3 | | | 8.643166 | | 2.086339 | | RR Ties | |
| 114 | | 114 | | 41396.3 | | | 130.5866 | | 23.08824 | | 181.9226 | | 22.88549 | | Mix_16 | | 103.1092 | | 350.8092 | 25 ton RR Ties, 25 ton Concrete, 6 Pallets CinderBlock | | rr ties, concrete, cinderblocks | RR, Concrete, CB | | RR, Concrete, CB | | mid | 5 | | 3 | | | 11.63095 | | 41.3963 | | RR Ties | |
| 115 | | 115 | | 31626.93 | | | 9 | | 23.1205 | | 201.1314 | | 9.91299 | | Mix_16 | | 103.1092 | | 350.8092 | 25 ton RR Ties, 25 ton Concrete, 6 Pallets CinderBlock | | rr ties, concrete, cinderblocks | RR, Concrete, CB | | RR, Concrete, CB | | mid | 5 | | 3 | | | 11.36176 | | 31.62693 | | RR Ties | |
| 116 | | 116 | | 18800.96 | | | 9 | | 23.07101 | | 212.9817 | | 20.67948 | | Mix_16 | | 103.1092 | | 350.8092 | 25 ton RR Ties, 25 ton Concrete, 6 Pallets CinderBlock | | rr ties, concrete, cinderblocks | RR, Concrete, CB | | RR, Concrete, CB | | mid | 5 | | 3 | | | 10.84166 | | 18.80096 | | RR Ties | |
| 117 | | 117 | | 12102.91 | | | 184.7 | | 21.15412 | | 269.5541 | | 6.592652 | | Mix_2 | | 63.34965 | | 252.1265 | 25 ton RR Ties, 25 ton Concrete, 6 Pallets CinderBlock | | rr ties, concrete, cinderblocks | RR, Concrete, CB | | RR, Concrete, CB | | mid | 5 | | 3 | | | 10.4012 | | 12.10291 | | RR Ties | |
| 118 | | 118 | | 35679.65 | | | 1539.543 | | 21.17201 | | 259.3551 | | 4.882191 | | Mix_2 | | 63.34965 | | 252.1265 | 25 ton RR Ties, 25 ton Concrete, 6 Pallets CinderBlock | | rr ties, concrete, cinderblocks | RR, Concrete, CB | | RR, Concrete, CB | | mid | 5 | | 3 | | | 11.48234 | | 35.67965 | | RR Ties | |
| 119 | | 119 | | 9358.121 | | | 3 | | 22.81305 | | 382.1696 | | 2.706441 | | Mix_24 | | 109.3002 | | 640.1769 | 25 ton RR Ties, 25 ton Concrete, 6 Pallets CinderBlock | | rr ties, concrete, cinderblocks | RR, Concrete, CB | | RR, Concrete, CB | | mid | 5 | | 3 | | | 10.144 | | 9.358121 | | RR Ties | |
| 120 | | 120 | | 9469.379 | | | 4 | | 22.57276 | | 653.4264 | | 7.41304 | | Mix_25 | | 85.41418 | | 375.2027 | 25 ton RR Ties, 25 ton Concrete, 6 Pallets CinderBlock | | rr ties, concrete, cinderblocks | RR, Concrete, CB | | RR, Concrete, CB | | mid | 5 | | 3 | | | 10.15582 | | 9.469379 | | RR Ties | |
| 121 | | 121 | | 18894.04 | | | 10 | | 22.26853 | | 571.09 | | 2.679752 | | Mix_27 | | 85.55578 | | 311.8222 | 25 ton RR Ties, 25 ton Concrete, 6 Pallets CinderBlock | | rr ties, concrete, cinderblocks | RR, Concrete, CB | | RR, Concrete, CB | | mid | 5 | | 3 | | | 10.8466 | | 18.89404 | | RR Ties | |
| 122 | | 122 | | 4020.891 | | | 2 | | 21.88666 | | 661.2387 | | 5.910493 | | Mix_28 | | 104.787 | | 330.1284 | 25 ton RR Ties, 25 ton Concrete, 6 Pallets CinderBlock | | rr ties, concrete, cinderblocks | RR, Concrete, CB | | RR, Concrete, CB | | mid | 5 | | 3 | | | 9.299259 | | 4.020891 | | RR Ties | |
| 123 | | 123 | | 18446.09 | | | 4 | | 21.02435 | | 175.4499 | | 21.94044 | | Mix_29 | | 98.22105 | | 525.312 | 25 ton RR Ties, 25 ton Concrete, 6 Pallets CinderBlock | | rr ties, concrete, cinderblocks | RR, Concrete, CB | | RR, Concrete, CB | | mid | 5 | | 3 | | | 10.82261 | | 18.44609 | | RR Ties | |
| 124 | | 124 | | 14506.21 | | | 669.3056 | | 21.3049 | | 196.3962 | | 8.474883 | | Mix_29 | | 98.22105 | | 525.312 | 25 ton RR Ties, 25 ton Concrete, 6 Pallets CinderBlock | | rr ties, concrete, cinderblocks | RR, Concrete, CB | | RR, Concrete, CB | | mid | 5 | | 3 | | | 10.58233 | | 14.50621 | | RR Ties | |
| 126 | | 126 | | 39652.92 | | | 9 | | 21.31565 | | 274.8885 | | 2.03923 | | Mix_3 | | 42.16232 | | 123.0869 | 25 ton RR Ties, 25 ton Concrete, 6 Pallets CinderBlock | | rr ties, concrete, cinderblocks | RR, Concrete, CB | | RR, Concrete, CB | | mid | 5 | | 3 | | | 11.58792 | | 39.65292 | | RR Ties | |
| 127 | | 127 | | 89001.46 | | | 10 | | 21.21416 | | 272.3918 | | 17.2722 | | Mix_3 | | 42.16232 | | 123.0869 | 25 ton RR Ties, 25 ton Concrete, 6 Pallets CinderBlock | | rr ties, concrete, cinderblocks | RR, Concrete, CB | | RR, Concrete, CB | | mid | 5 | | 3 | | | 12.39641 | | 89.00146 | | RR Ties | |
| 128 | | 128 | | 4638.421 | | | 2 | | 21.08952 | | 184.428 | | 14.7241 | | Mix_30 | | 53.99674 | | 138.79 | 25 ton RR Ties, 25 ton Concrete, 6 Pallets CinderBlock | | rr ties, concrete, cinderblocks | RR, Concrete, CB | | RR, Concrete, CB | | mid | 5 | | 3 | | | 9.442129 | | 4.638421 | | RR Ties | |
| 129 | | 129 | | 11727.44 | | | 263.4776 | | 21.37117 | | 191.6629 | | 10.13594 | | Mix_31 | | 69.17692 | | 162.4133 | 25 ton RR Ties, 25 ton Concrete, 6 Pallets CinderBlock | | rr ties, concrete, cinderblocks | RR, Concrete, CB | | RR, Concrete, CB | | mid | 5 | | 3 | | | 10.36969 | | 11.72744 | | RR Ties | |
| 130 | | 130 | | 912.2959 | | | 2 | | 21.66019 | | 190.4293 | | 22.89296 | | Mix_34 | | 74.69929 | | 239.8884 | 25 ton RR Ties, 25 ton Concrete, 6 Pallets CinderBlock | | rr ties, concrete, cinderblocks | RR, Concrete, CB | | RR, Concrete, CB | | mid | 5 | | 3 | | | 7.815964 | | 0.912296 | | RR Ties | |
| 131 | | 131 | | 635.0437 | | | 2 | | 22.04487 | | 186.2839 | | 6.86242 | | Mix_38 | | 80.53022 | | 289.182 | 25 ton RR Ties, 25 ton Concrete, 6 Pallets CinderBlock | | rr ties, concrete, cinderblocks | RR, Concrete, CB | | RR, Concrete, CB | | mid | 5 | | 3 | | | 7.453694 | | 0.635044 | | RR Ties | |
| 132 | | 132 | | 17326.17 | | | 13 | | 22.97652 | | 242.5356 | | 2.1679 | | Mix_39 | | 58.46545 | | 169.2418 | 25 ton RR Ties, 25 ton Concrete, 6 Pallets CinderBlock | | rr ties, concrete, cinderblocks | RR, Concrete, CB | | RR, Concrete, CB | | mid | 5 | | 3 | | | 10.75997 | | 17.32617 | | RR Ties | |
| 133 | | 133 | | 15314.8 | | | 13.4845 | | 21.28581 | | 234.3441 | | 6.10833 | | Mix_4 | | 52.4202 | | 194.0167 | 25 ton RR Ties, 25 ton Concrete, 6 Pallets CinderBlock | | rr ties, concrete, cinderblocks | RR, Concrete, CB | | RR, Concrete, CB | | mid | 5 | | 3 | | | 10.63658 | | 15.3148 | | RR Ties | |
| 134 | | 134 | | 108196.8 | | | 16 | | 23.12176 | | 153.1841 | | 7.830021 | | Mix_40 | | 107.8763 | | 495.4374 | 25 ton RR Ties, 25 ton Concrete, 6 Pallets CinderBlock | | rr ties, concrete, cinderblocks | RR, Concrete, CB | | RR, Concrete, CB | | mid | 5 | | 3 | | | 12.59171 | | 108.1968 | | RR Ties | |
| 135 | | 135 | | 2001.143 | | | 3 | | 22.50602 | | 286.3572 | | 21.09752 | | Mix_41 | | 72.96277 | | 272.4023 | 25 ton RR Ties, 25 ton Concrete, 6 Pallets CinderBlock | | rr ties, concrete, cinderblocks | RR, Concrete, CB | | RR, Concrete, CB | | mid | 5 | | 3 | | | 8.601474 | | 2.001143 | | RR Ties | |
| 136 | | 136 | | 17527.46 | | | 8 | | 22.7754 | | 285.1299 | | 4.316622 | | Mix_42 | | 67.51195 | | 262.8961 | 25 ton RR Ties, 25 ton Concrete, 6 Pallets CinderBlock | | rr ties, concrete, cinderblocks | RR, Concrete, CB | | RR, Concrete, CB | | mid | 5 | | 3 | | | 10.77152 | | 17.52746 | | RR Ties | |
| 137 | | 137 | | 5444.936 | | | 2 | | 23.14959 | | 199.7843 | | 6.076646 | | Mix_44 | | 100.7879 | | 514.4661 | 25 ton RR Ties, 25 ton Concrete, 6 Pallets CinderBlock | | rr ties, concrete, cinderblocks | RR, Concrete, CB | | RR, Concrete, CB | | mid | 5 | | 3 | | | 9.602441 | | 5.444936 | | RR Ties | |
| 138 | | 138 | | 29499.13 | | | 12 | | 22.96104 | | 254.3363 | | 9.134197 | | Mix_45 | | 85.72191 | | 391.1622 | 25 ton RR Ties, 25 ton Concrete, 6 Pallets CinderBlock | | rr ties, concrete, cinderblocks | RR, Concrete, CB | | RR, Concrete, CB | | mid | 5 | | 3 | | | 11.29212 | | 29.49913 | | RR Ties | |
| 139 | | 139 | | 18280.13 | | | 333.0445 | | 21.63622 | | 232.9968 | | 7.427213 | | Mix_5 | | 65.91758 | | 213.643 | 25 ton RR Ties, 25 ton Concrete, 6 Pallets CinderBlock | | rr ties, concrete, cinderblocks | RR, Concrete, CB | | RR, Concrete, CB | | mid | 5 | | 3 | | | 10.81357 | | 18.28013 | | RR Ties | |
| 140 | | 140 | | 28583.98 | | | 8 | | 21.69022 | | 273.3839 | | 9.898185 | | Mix_6 | | 61.10573 | | 192.3106 | 25 ton RR Ties, 25 ton Concrete, 6 Pallets CinderBlock | | rr ties, concrete, cinderblocks | RR, Concrete, CB | | RR, Concrete, CB | | mid | 5 | | 3 | | | 11.2606 | | 28.58398 | | RR Ties | |
| 378 | | 378 | | 24865.91 | | | 151 | | 23.08107 | | 141.6561 | | 30.99372 | | Mix_40 | | 107.8763 | | 495.4374 | 25 ton RR Ties, 25 ton Concrete, 6 Pallets CinderBlock | | rr ties, concrete, cinderblocks | RR, Concrete, CB | | RR, Concrete, CB | | mid | 5 | | 3 | | | 11.12125 | | 24.86591 | | RR Ties | |
| 387 | | 387 | | 4652.906 | | | 2 | | 22.88293 | | 246.7083 | | 20.40077 | | Mix_39 | | 58.46545 | | 169.2418 | 25 ton RR Ties, 25 ton Concrete, 6 Pallets CinderBlock | | rr ties, concrete, cinderblocks | RR, Concrete, CB | | RR, Concrete, CB | | mid | 5 | | 3 | | | 9.445247 | | 4.652906 | | RR Ties | |
| 405 | | 405 | | 1348.274 | | | 9 | | 21.08823 | | 319.6037 | | 46.19629 | | Mix_3 | | 42.16232 | | 123.0869 | 25 ton RR Ties, 25 ton Concrete, 6 Pallets CinderBlock | | rr ties, concrete, cinderblocks | RR, Concrete, CB | | RR, Concrete, CB | | mid | 5 | | 3 | | | 8.20658 | | 1.348274 | | RR Ties | |
| 494 | | -1 | | 1303.572 | | | 1 | | 22.48554 | | 208.005 | | 14.79747 | | Mix_12 | | 66.70157 | | 245.3528 | 25 ton RR Ties, 25 ton Concrete, 6 Pallets CinderBlock | | rr ties, concrete, cinderblocks | RR, Concrete, CB | | RR, Concrete, CB | | mid | 5 | | 3 | | | 8.172864 | | 1.303572 | | RR Ties | |
| 495 | | -1 | | 86.7102 | | | 1 | | 21.88644 | | 670.1267 | | 17.61436 | | Mix_28 | | 104.787 | | 330.1284 | 25 ton RR Ties, 25 ton Concrete, 6 Pallets CinderBlock | | rr ties, concrete, cinderblocks | RR, Concrete, CB | | RR, Concrete, CB | | mid | 5 | | 3 | | | 5.462572 | | 0.08671 | | RR Ties | |
| 496 | | -1 | | 348.5601 | | | 1 | | 21.27811 | | 198.0183 | | 21.71764 | | Mix_29 | | 98.22105 | | 525.312 | 25 ton RR Ties, 25 ton Concrete, 6 Pallets CinderBlock | | rr ties, concrete, cinderblocks | RR, Concrete, CB | | RR, Concrete, CB | | mid | 5 | | 3 | | | 6.853811 | | 0.34856 | | RR Ties | |
| 498 | | -1 | | 859.2773 | | | 1 | | 21.85933 | | 207.7234 | | 6.040595 | | Mix_35 | | 71.08347 | | 247.0862 | 25 ton RR Ties, 25 ton Concrete, 6 Pallets CinderBlock | | rr ties, concrete, cinderblocks | RR, Concrete, CB | | RR, Concrete, CB | | mid | 5 | | 3 | | | 7.756092 | | 0.859277 | | RR Ties | |
| 499 | | -1 | | 836.2088 | | | 1 | | 21.86667 | | 189.9249 | | 16.72658 | | Mix_36 | | 70.42796 | | 272.3004 | 25 ton RR Ties, 25 ton Concrete, 6 Pallets CinderBlock | | rr ties, concrete, cinderblocks | RR, Concrete, CB | | RR, Concrete, CB | | mid | 5 | | 3 | | | 7.728878 | | 0.836209 | | RR Ties | |
| 500 | | -1 | | 218.6577 | | | 1 | | 21.97068 | | 202.6513 | | 3.076164 | | Mix_37 | | 86.57344 | | 420.9694 | 25 ton RR Ties, 25 ton Concrete, 6 Pallets CinderBlock | | rr ties, concrete, cinderblocks | RR, Concrete, CB | | RR, Concrete, CB | | mid | 5 | | 3 | | | 6.387508 | | 0.218658 | | RR Ties | |
| 501 | | -1 | | 725.4587 | | | 1 | | 22.71553 | | 291.9039 | | 24.9702 | | Mix_42 | | 67.51195 | | 262.8961 | 25 ton RR Ties, 25 ton Concrete, 6 Pallets CinderBlock | | rr ties, concrete, cinderblocks | RR, Concrete, CB | | RR, Concrete, CB | | mid | 5 | | 3 | | | 7.586804 | | 0.725459 | | RR Ties | |
| 502 | | -1 | | 496.3907 | | | 1 | | 21.61471 | | 267.9071 | | 12.52677 | | Mix_6 | | 61.10573 | | 192.3106 | 25 ton RR Ties, 25 ton Concrete, 6 Pallets CinderBlock | | rr ties, concrete, cinderblocks | RR, Concrete, CB | | RR, Concrete, CB | | mid | 5 | | 3 | | | 7.207363 | | 0.496391 | | RR Ties | |
| 503 | | -1 | | 408.3771 | | | 20.41885 | | 21.8052 | | 263.6286 | | 7.122224 | | Mix_7 | | 107.4446 | | 419.4349 | 25 ton RR Ties, 25 ton Concrete, 6 Pallets CinderBlock | | rr ties, concrete, cinderblocks | RR, Concrete, CB | | RR, Concrete, CB | | mid | 5 | | 3 | | | 7.012191 | | 0.408377 | | RR Ties | |
| 689 | | -1 | | 908.1098 | | | 1 | | 20.73302 | | 257.1218 | | 31.82362 | | Mix_1 | | 54.124 | | 166.0747 | 25 ton RR Ties, 25 ton Concrete, 6 Pallets CinderBlock | | rr ties, concrete, cinderblocks | RR, Concrete, CB | | RR, Concrete, CB | | mid | 5 | | 3 | | | 7.811365 | | 0.90811 | | RR Ties | |
| 690 | | -1 | | 112.4578 | | | 1 | | 21.79752 | | 272.6759 | | 30.46012 | | Mix_7 | | 107.4446 | | 419.4349 | 25 ton RR Ties, 25 ton Concrete, 6 Pallets CinderBlock | | rr ties, concrete, cinderblocks | RR, Concrete, CB | | RR, Concrete, CB | | mid | 5 | | 3 | | | 5.722578 | | 0.112458 | | RR Ties | |
| 691 | | -1 | | 2801.918 | | | 1 | | 23.02975 | | 216.2857 | | 12.22058 | | Mix_44 | | 100.7879 | | 514.4661 | 25 ton RR Ties, 25 ton Concrete, 6 Pallets CinderBlock | | rr ties, concrete, cinderblocks | RR, Concrete, CB | | RR, Concrete, CB | | mid | 5 | | 3 | | | 8.938059 | | 2.801918 | | RR Ties | |
| 693 | | -1 | | 942.5589 | | | 1 | | 22.41888 | | 217.8208 | | 60.67482 | | Mix_12 | | 66.70157 | | 245.3528 | 25 ton RR Ties, 25 ton Concrete, 6 Pallets CinderBlock | | rr ties, concrete, cinderblocks | RR, Concrete, CB | | RR, Concrete, CB | | mid | 5 | | 3 | | | 7.848598 | | 0.942559 | | RR Ties | |
| 694 | | -1 | | 327.8892 | | | 1 | | 21.94251 | | 206.7121 | | 19.13934 | | Mix_37 | | 86.57344 | | 420.9694 | 25 ton RR Ties, 25 ton Concrete, 6 Pallets CinderBlock | | rr ties, concrete, cinderblocks | RR, Concrete, CB | | RR, Concrete, CB | | mid | 5 | | 3 | | | 6.792676 | | 0.327889 | | RR Ties | |
| 695 | | -1 | | 2102.997 | | | 1 | | 21.13536 | | 186.0668 | | 30.22459 | | Mix_31 | | 69.17692 | | 162.4133 | 25 ton RR Ties, 25 ton Concrete, 6 Pallets CinderBlock | | rr ties, concrete, cinderblocks | RR, Concrete, CB | | RR, Concrete, CB | | mid | 5 | | 3 | | | 8.651119 | | 2.102997 | | RR Ties | |
| 705 | | -1 | | 34.55705 | | | 1 | | 21.52254 | | 223.7878 | | 34.57674 | | Mix_34 | | 74.69929 | | 239.8884 | 25 ton RR Ties, 25 ton Concrete, 6 Pallets CinderBlock | | rr ties, concrete, cinderblocks | RR, Concrete, CB | | RR, Concrete, CB | | mid | 5 | | 3 | | | 4.542611 | | 0.034557 | | RR Ties | |
| 706 | | -1 | | 692.542 | | | 1 | | 21.64019 | | 255.287 | | 26.02027 | | Mix_5 | | 65.91758 | | 213.643 | 25 ton RR Ties, 25 ton Concrete, 6 Pallets CinderBlock | | rr ties, concrete, cinderblocks | RR, Concrete, CB | | RR, Concrete, CB | | mid | 5 | | 3 | | | 7.540369 | | 0.692542 | | RR Ties | |
| 709 | | -1 | | 5592.453 | | | 1 | | 21.67016 | | 227.0472 | | 24.06895 | | Mix_5 | | 65.91758 | | 213.643 | 25 ton RR Ties, 25 ton Concrete, 6 Pallets CinderBlock | | rr ties, concrete, cinderblocks | RR, Concrete, CB | | RR, Concrete, CB | | mid | 5 | | 3 | | | 9.629173 | | 5.592453 | | RR Ties | |
| 711 | | -1 | | 2845.809 | | | 1 | | 22.64312 | | 395.3061 | | 39.21448 | | Mix_43 | | 98.51667 | | 508.1083 | 25 ton RR Ties, 25 ton Concrete, 6 Pallets CinderBlock | | rr ties, concrete, cinderblocks | RR, Concrete, CB | | RR, Concrete, CB | | mid | 5 | | 3 | | | 8.953603 | | 2.845809 | | RR Ties | |
| 712 | | -1 | | 46.46175 | | | 1 | | 22.57966 | | 613.3228 | | 54.97228 | | Mix_25 | | 85.41418 | | 375.2027 | 25 ton RR Ties, 25 ton Concrete, 6 Pallets CinderBlock | | rr ties, concrete, cinderblocks | RR, Concrete, CB | | RR, Concrete, CB | | mid | 5 | | 3 | | | 4.838629 | | 0.046462 | | RR Ties | |
| 713 | | -1 | | 607.2436 | | | 1 | | 22.56004 | | 625.284 | | 44.38921 | | Mix_25 | | 85.41418 | | 375.2027 | 25 ton RR Ties, 25 ton Concrete, 6 Pallets CinderBlock | | rr ties, concrete, cinderblocks | RR, Concrete, CB | | RR, Concrete, CB | | mid | 5 | | 3 | | | 7.40893 | | 0.607244 | | RR Ties | |
| 714 | | -1 | | 43.53598 | | | 1 | | 22.63893 | | 609.6303 | | 63.61884 | | Mix_25 | | 85.41418 | | 375.2027 | 25 ton RR Ties, 25 ton Concrete, 6 Pallets CinderBlock | | rr ties, concrete, cinderblocks | RR, Concrete, CB | | RR, Concrete, CB | | mid | 5 | | 3 | | | 4.773588 | | 0.043536 | | RR Ties | |
| 732 | | -1 | | 2605.997 | | | 1 | | 22.41214 | | 552.5625 | | 33.0001 | | Mix_26 | | 63.82449 | | 229.8231 | 25 ton RR Ties, 25 ton Concrete, 6 Pallets CinderBlock | | rr ties, concrete, cinderblocks | RR, Concrete, CB | | RR, Concrete, CB | | mid | 5 | | 3 | | | 8.865571 | | 2.605997 | | RR Ties | |
| 793 | | -1 | | 942.0655 | | | 47 | | 21.50042 | | 243.8403 | | 38.06578 | | Mix_5 | | 65.91758 | | 213.643 | 25 ton RR Ties, 25 ton Concrete, 6 Pallets CinderBlock | | rr ties, concrete, cinderblocks | RR, Concrete, CB | | RR, Concrete, CB | | mid | 5 | | 3 | | | 7.848075 | | 0.942065 | | RR Ties | |
| 68 | | 68 | | 13182.96 | | | 7 | | 22.07498 | | 847.4287 | | 12.84218 | | Concrete10 ton6 | | 13.46695 | | 10.89859 | 10 ton concrete | | concrete | 10T Concrete | | Concrete | | low | 7 | | 1 | | | 10.48668 | | 13.18296 | | Concrete | |
| 69 | | 69 | | 36183.53 | | | 9 | | 21.97752 | | 838.8123 | | 8.339365 | | Concrete10 ton7 | | 17.92223 | | 19.80858 | 10 ton concrete | | concrete | 10T Concrete | | Concrete | | low | 7 | | 1 | | | 11.49636 | | 36.18353 | | Concrete | |
| 70 | | 70 | | 5475.042 | | | 4 | | 21.8508 | | 870.0239 | | 20.00033 | | Concrete10 ton8 | | 26.04407 | | 43.72264 | 10 ton concrete | | concrete | 10T Concrete | | Concrete | | low | 7 | | 1 | | | 9.607955 | | 5.475042 | | Concrete | |
| 372 | | 372 | | 7057.143 | | | 4 | | 21.8088 | | 832.7754 | | 18.07346 | | Concrete10 ton8 | | 26.04407 | | 43.72264 | 10 ton concrete | | concrete | 10T Concrete | | Concrete | | low | 7 | | 1 | | | 9.861796 | | 7.057143 | | Concrete | |
| 373 | | 373 | | 786.6036 | | | 2 | | 21.86342 | | 827.6151 | | 24.24752 | | Concrete10 ton8 | | 26.04407 | | 43.72264 | 10 ton concrete | | concrete | 10T Concrete | | Concrete | | low | 7 | | 1 | | | 7.667724 | | 0.786604 | | Concrete | |
| 375 | | 375 | | 28735.3 | | | 15 | | 21.69472 | | 824.4614 | | 21.60949 | | Concrete10 ton10 | | 25.53049 | | 42.72788 | 10 ton concrete | | concrete | 10T Concrete | | Concrete | | low | 7 | | 1 | | | 11.26588 | | 28.7353 | | Concrete | |
| 461 | | -1 | | 4993.594 | | | 1 | | 21.56815 | | 846.5559 | | 17.22297 | | Concrete10 ton13 | | 30.33218 | | 44.4319 | 10 ton concrete | | concrete | 10T Concrete | | Concrete | | low | 7 | | 1 | | | 9.515911 | | 4.993594 | | Concrete | |
| 462 | | -1 | | 1658.355 | | | 1 | | 21.42475 | | 836.6996 | | 3.839867 | | Concrete10 ton13 | | 30.33218 | | 44.4319 | 10 ton concrete | | concrete | 10T Concrete | | Concrete | | low | 7 | | 1 | | | 8.413581 | | 1.658355 | | Concrete | |
| 463 | | -1 | | 1117.577 | | | 1 | | 22.62821 | | 745.7971 | | 7.489898 | | Concrete10 ton4 | | 14.97962 | | 15.87133 | 10 ton concrete | | concrete | 10T Concrete | | Concrete | | low | 7 | | 1 | | | 8.018918 | | 1.117577 | | Concrete | |
| 464 | | -1 | | 1365.591 | | | 1 | | 22.12216 | | 833.2196 | | 10.89918 | | Concrete10 ton6 | | 13.46695 | | 10.89859 | 10 ton concrete | | concrete | 10T Concrete | | Concrete | | low | 7 | | 1 | | | 8.219343 | | 1.365591 | | Concrete | |
| 465 | | -1 | | 3375.83 | | | 1 | | 21.87133 | | 839.912 | | 14.46167 | | Concrete10 ton8 | | 26.04407 | | 43.72264 | 10 ton concrete | | concrete | 10T Concrete | | Concrete | | low | 7 | | 1 | | | 9.124396 | | 3.37583 | | Concrete | |
| 466 | | -1 | | 1985.057 | | | 1 | | 21.77775 | | 855.1317 | | 18.72949 | | Concrete10 ton9 | | 20.11077 | | 14.89949 | 10 ton concrete | | concrete | 10T Concrete | | Concrete | | low | 7 | | 1 | | | 8.593403 | | 1.985057 | | Concrete | |
| 681 | | -1 | | 194.0734 | | | 1 | | 21.63522 | | 797.9763 | | 51.70838 | | Concrete10 ton12 | | 20.92202 | | 28.33798 | 10 ton concrete | | concrete | 10T Concrete | | Concrete | | low | 7 | | 1 | | | 6.268236 | | 0.194073 | | Concrete | |
| 704 | | -1 | | 1335.641 | | | 1 | | 22.55878 | | 661.307 | | 15.27932 | | Concrete10 ton3 | | 15.69201 | | 17.47769 | 10 ton concrete | | concrete | 10T Concrete | | Concrete | | low | 7 | | 1 | | | 8.197167 | | 1.335641 | | Concrete | |
| 327 | | 327 | | 538.0533 | | | 2 | | 19.79667 | | 407.3753 | | 29.58331 | | Patch 9 | | 138.6624 | | 1213.251 | low profile | | low profile | 16LP | | LP | | low | 7 | | 1 | | | 7.287958 | | 0.538053 | | Low Profile | |
| 506 | | -1 | | 1586.696 | | | 1 | | 19.1809 | | 194.9501 | | 43.98648 | | Patch 1 | | 194.0583 | | 2083.393 | low profile | | low profile | 16LP | | LP | | low | 7 | | 1 | | | 8.369409 | | 1.586696 | | Low Profile | |
| 507 | | -1 | | 1071.578 | | | 1 | | 19.1404 | | 184.3942 | | 16.31613 | | Patch 1 | | 194.0583 | | 2083.393 | low profile | | low profile | 16LP | | LP | | low | 7 | | 1 | | | 7.976888 | | 1.071578 | | Low Profile | |
| 516 | | -1 | | 485.2197 | | | 24.26099 | | 19.23973 | | 393.2282 | | 16.8061 | | Patch 22 | | 156.8225 | | 1234.879 | low profile | | low profile | 16LP | | LP | | low | 7 | | 1 | | | 7.184602 | | 0.48522 | | Low Profile | |
| 703 | | -1 | | 1181.788 | | | 1 | | 18.99157 | | 215.6505 | | 34.37837 | | Patch 1 | | 194.0583 | | 2083.393 | low profile | | low profile | 16LP | | LP | | low | 7 | | 1 | | | 8.074783 | | 1.181788 | | Low Profile | |
| 746 | | -1 | | 27.30022 | | | 1 | | 19.92361 | | 689.9417 | | 36.58094 | | Patch 34 | | 101.4893 | | 546.5121 | low profile | | low profile | 16LP | | LP | | low | 7 | | 1 | | | 4.306895 | | 0.0273 | | Low Profile | |
| 773 | | -1 | | 93.31528 | | | 5 | | 19.78859 | | 287.2136 | | 106.4294 | | Patch 9 | | 138.6624 | | 1213.251 | low profile | | low profile | 16LP | | LP | | low | 7 | | 1 | | | 5.535984 | | 0.093315 | | Low Profile | |
| 774 | | -1 | | 95.85001 | | | 5 | | 19.28471 | | 171.607 | | 66.52647 | | Patch 1 | | 194.0583 | | 2083.393 | low profile | | low profile | 16LP | | LP | | low | 7 | | 1 | | | 5.562785 | | 0.09585 | | Low Profile | |
| 775 | | -1 | | 99.27403 | | | 5 | | 19.88154 | | 270.9311 | | 120.0895 | | Patch 9 | | 138.6624 | | 1213.251 | low profile | | low profile | 16LP | | LP | | low | 7 | | 1 | | | 5.597884 | | 0.099274 | | Low Profile | |
| 779 | | -1 | | 114.8205 | | | 6 | | 19.8895 | | 454.9823 | | 71.8622 | | Patch 9 | | 138.6624 | | 1213.251 | low profile | | low profile | 16LP | | LP | | low | 7 | | 1 | | | 5.74337 | | 0.11482 | | Low Profile | |
| 780 | | -1 | | 122.3881 | | | 6 | | 19.28967 | | 68.13876 | | 125.0759 | | Patch 1 | | 194.0583 | | 2083.393 | low profile | | low profile | 16LP | | LP | | low | 7 | | 1 | | | 5.807197 | | 0.122388 | | Low Profile | |
| 783 | | -1 | | 197.7667 | | | 10 | | 19.86106 | | 649.5484 | | 94.92753 | | Patch 34 | | 101.4893 | | 546.5121 | low profile | | low profile | 16LP | | LP | | low | 7 | | 1 | | | 6.287088 | | 0.197767 | | Low Profile | |
| 784 | | -1 | | 199.1345 | | | 10 | | 19.80183 | | 280.2926 | | 111.0721 | | Patch 9 | | 138.6624 | | 1213.251 | low profile | | low profile | 16LP | | LP | | low | 7 | | 1 | | | 6.29398 | | 0.199134 | | Low Profile | |
| 791 | | -1 | | 683.6568 | | | 34 | | 20.05832 | | 452.0848 | | 42.40381 | | Patch 44 | | 150.0933 | | 1491.032 | low profile | | low profile | 16LP | | LP | | low | 7 | | 1 | | | 7.527456 | | 0.683657 | | Low Profile | |
| 799 | | -1 | | 2025.737 | | | 101 | | 19.85824 | | 252.446 | | 141.8032 | | Patch 9 | | 138.6624 | | 1213.251 | low profile | | low profile | 16LP | | LP | | low | 7 | | 1 | | | 8.613689 | | 2.025737 | | Low Profile | |
| 150 | | 150 | | 60512.32 | | | 247.4523 | | 18.88598 | | 210.9148 | | 25.27322 | | Patch 11 | | 185.4546 | | 2174.576 | mixed; pyramids, lpm | | pyramids, low profile | 16MX | | MX | | mid | 7 | | 2 | | | 12.0106 | | 60.51232 | | Pyramids | |
| 151 | | 151 | | 19820.99 | | | 9 | | 18.70017 | | 250.9362 | | 55.71085 | | Patch 11 | | 185.4546 | | 2174.576 | mixed; pyramids, lpm | | pyramids, low profile | 16MX | | MX | | mid | 7 | | 2 | | | 10.8945 | | 19.82099 | | Pyramids | |
| 159 | | 159 | | 1910.1 | | | 2 | | 19.87058 | | 524.8856 | | 33.25871 | | Patch 18 | | 200.779 | | 2703.611 | mixed; pyramids, lpm | | pyramids, low profile | 16MX | | MX | | mid | 7 | | 2 | | | 8.554911 | | 1.9101 | | Pyramids | |
| 160 | | 160 | | 3964.107 | | | 3 | | 20.16667 | | 582.2144 | | 26.58056 | | Patch 18 | | 200.779 | | 2703.611 | mixed; pyramids, lpm | | pyramids, low profile | 16MX | | MX | | mid | 7 | | 2 | | | 9.285036 | | 3.964107 | | Pyramids | |
| 161 | | 161 | | 20803.73 | | | 15 | | 20.13307 | | 559.1563 | | 5.661481 | | Patch 18 | | 200.779 | | 2703.611 | mixed; pyramids, lpm | | pyramids, low profile | 16MX | | MX | | mid | 7 | | 2 | | | 10.94289 | | 20.80373 | | Pyramids | |
| 162 | | 162 | | 1660.348 | | | 3 | | 19.54933 | | 581.2181 | | 35.38785 | | Patch 18 | | 200.779 | | 2703.611 | mixed; pyramids, lpm | | pyramids, low profile | 16MX | | MX | | mid | 7 | | 2 | | | 8.414783 | | 1.660348 | | Pyramids | |
| 163 | | 163 | | 1025.944 | | | 14.21244 | | 19.80846 | | 569.6484 | | 16.02792 | | Patch 18 | | 200.779 | | 2703.611 | mixed; pyramids, lpm | | pyramids, low profile | 16MX | | MX | | mid | 7 | | 2 | | | 7.933368 | | 1.025944 | | Pyramids | |
| 164 | | 164 | | 6348.612 | | | 2 | | 19.89317 | | 538.9703 | | 28.56048 | | Patch 18 | | 200.779 | | 2703.611 | mixed; pyramids, lpm | | pyramids, low profile | 16MX | | MX | | mid | 7 | | 2 | | | 9.755992 | | 6.348612 | | Pyramids | |
| 167 | | 167 | | 16745.48 | | | 2 | | 19.43661 | | 230.1022 | | 21.33582 | | Patch 21 | | 220.0517 | | 2852.056 | mixed; pyramids, lpm | | pyramids, low profile | 16MX | | MX | | mid | 7 | | 2 | | | 10.72588 | | 16.74548 | | Pyramids | |
| 168 | | 168 | | 762.6259 | | | 2 | | 19.56525 | | 175.4477 | | 39.25532 | | Patch 21 | | 220.0517 | | 2852.056 | mixed; pyramids, lpm | | pyramids, low profile | 16MX | | MX | | mid | 7 | | 2 | | | 7.636768 | | 0.762626 | | Pyramids | |
| 169 | | 169 | | 21751.58 | | | 5 | | 18.92188 | | 197.9048 | | 45.50852 | | Patch 21 | | 220.0517 | | 2852.056 | mixed; pyramids, lpm | | pyramids, low profile | 16MX | | MX | | mid | 7 | | 2 | | | 10.98744 | | 21.75158 | | Pyramids | |
| 188 | | 188 | | 11122.99 | | | 3 | | 20.26701 | | 288.1256 | | 25.88383 | | Patch 48 | | 194.3671 | | 2223.669 | mixed; pyramids, lpm | | pyramids, low profile | 16MX | | MX | | mid | 7 | | 2 | | | 10.31677 | | 11.12299 | | Pyramids | |
| 189 | | 189 | | 2702.993 | | | 3 | | 19.91514 | | 277.9938 | | 43.49238 | | Patch 48 | | 194.3671 | | 2223.669 | mixed; pyramids, lpm | | pyramids, low profile | 16MX | | MX | | mid | 7 | | 2 | | | 8.902115 | | 2.702993 | | Pyramids | |
| 190 | | 190 | | 20543.2 | | | 20.28667 | | 20.09696 | | 245.0975 | | 40.55233 | | Patch 48 | | 194.3671 | | 2223.669 | mixed; pyramids, lpm | | pyramids, low profile | 16MX | | MX | | mid | 7 | | 2 | | | 10.93029 | | 20.5432 | | Pyramids | |
| 191 | | 191 | | 10052.48 | | | 8 | | 20.52655 | | 220.5336 | | 50.42004 | | Patch 5 | | 177.1536 | | 2255.842 | mixed; pyramids, lpm | | pyramids, low profile | 16MX | | MX | | mid | 7 | | 2 | | | 10.21557 | | 10.05248 | | Pyramids | |
| 192 | | 192 | | 2810.911 | | | 2 | | 20.88081 | | 198.4082 | | 10.17774 | | Patch 5 | | 177.1536 | | 2255.842 | mixed; pyramids, lpm | | pyramids, low profile | 16MX | | MX | | mid | 7 | | 2 | | | 8.941264 | | 2.810911 | | Pyramids | |
| 193 | | 193 | | 7246.22 | | | 4 | | 20.79432 | | 212.0189 | | 31.2561 | | Patch 5 | | 177.1536 | | 2255.842 | mixed; pyramids, lpm | | pyramids, low profile | 16MX | | MX | | mid | 7 | | 2 | | | 9.888235 | | 7.24622 | | Pyramids | |
| 325 | | 325 | | 1842.275 | | | 23 | | 18.50671 | | 232.7427 | | 62.24645 | | Patch 11 | | 185.4546 | | 2174.576 | mixed; pyramids, lpm | | pyramids, low profile | 16MX | | MX | | mid | 7 | | 2 | | | 8.518756 | | 1.842275 | | Pyramids | |
| 331 | | 331 | | 32086.06 | | | 1398 | | 19.16465 | | 485.0248 | | 84.31418 | | Patch 18 | | 200.779 | | 2703.611 | mixed; pyramids, lpm | | pyramids, low profile | 16MX | | MX | | mid | 7 | | 2 | | | 11.37618 | | 32.08606 | | Pyramids | |
| 339 | | 339 | | 2374.554 | | | 38 | | 19.18336 | | 23.08186 | | 188.4458 | | Patch 21 | | 220.0517 | | 2852.056 | mixed; pyramids, lpm | | pyramids, low profile | 16MX | | MX | | mid | 7 | | 2 | | | 8.772565 | | 2.374554 | | Pyramids | |
| 514 | | -1 | | 4696.763 | | | 1 | | 19.00938 | | 177.8862 | | 51.53074 | | Patch 21 | | 220.0517 | | 2852.056 | mixed; pyramids, lpm | | pyramids, low profile | 16MX | | MX | | mid | 7 | | 2 | | | 9.454629 | | 4.696763 | | Pyramids | |
| 515 | | -1 | | 3356.234 | | | 1 | | 19.16716 | | 150.1826 | | 98.1052 | | Patch 21 | | 220.0517 | | 2852.056 | mixed; pyramids, lpm | | pyramids, low profile | 16MX | | MX | | mid | 7 | | 2 | | | 9.118575 | | 3.356234 | | Pyramids | |
| 520 | | -1 | | 8429.938 | | | 1 | | 20.89391 | | 834.4419 | | 29.80663 | | Patch 35 | | 209.6211 | | 2787.588 | mixed; pyramids, lpm | | pyramids, low profile | 16MX | | MX | | mid | 7 | | 2 | | | 10.03954 | | 8.429938 | | Pyramids | |
| 521 | | -1 | | 60.59057 | | | 3.029528 | | 20.81514 | | 815.0225 | | 30.78285 | | Patch 35 | | 209.6211 | | 2787.588 | mixed; pyramids, lpm | | pyramids, low profile | 16MX | | MX | | mid | 7 | | 2 | | | 5.104139 | | 0.060591 | | Pyramids | |
| 522 | | -1 | | 253.125 | | | 12.65625 | | 20.53976 | | 814.8723 | | 51.16519 | | Patch 35 | | 209.6211 | | 2787.588 | mixed; pyramids, lpm | | pyramids, low profile | 16MX | | MX | | mid | 7 | | 2 | | | 6.533883 | | 0.253125 | | Pyramids | |
| 523 | | -1 | | 50993.37 | | | 2549.668 | | 20.76401 | | 815.7297 | | 11.47193 | | Patch 35 | | 209.6211 | | 2787.588 | mixed; pyramids, lpm | | pyramids, low profile | 16MX | | MX | | mid | 7 | | 2 | | | 11.83945 | | 50.99337 | | Pyramids | |
| 532 | | -1 | | 802.8966 | | | 1 | | 19.861 | | 314.9035 | | 47.76054 | | Patch 48 | | 194.3671 | | 2223.669 | mixed; pyramids, lpm | | pyramids, low profile | 16MX | | MX | | mid | 7 | | 2 | | | 7.688226 | | 0.802897 | | Pyramids | |
| 533 | | -1 | | 535.3859 | | | 1 | | 20.57064 | | 287.5805 | | 10.62322 | | Patch 48 | | 194.3671 | | 2223.669 | mixed; pyramids, lpm | | pyramids, low profile | 16MX | | MX | | mid | 7 | | 2 | | | 7.282988 | | 0.535386 | | Pyramids | |
| 534 | | -1 | | 47.66885 | | | 2.383442 | | 20.32873 | | 273.2137 | | 9.69684 | | Patch 48 | | 194.3671 | | 2223.669 | mixed; pyramids, lpm | | pyramids, low profile | 16MX | | MX | | mid | 7 | | 2 | | | 4.864278 | | 0.047669 | | Pyramids | |
| 535 | | -1 | | 1188.952 | | | 1 | | 20.8627 | | 202.478 | | 8.979582 | | Patch 5 | | 177.1536 | | 2255.842 | mixed; pyramids, lpm | | pyramids, low profile | 16MX | | MX | | mid | 7 | | 2 | | | 8.080827 | | 1.188952 | | Pyramids | |
| 656 | | -1 | | 12782.93 | | | 1 | | 18.83574 | | 101.3036 | | 164.0245 | | Patch 21 | | 220.0517 | | 2852.056 | mixed; pyramids, lpm | | pyramids, low profile | 16MX | | MX | | mid | 7 | | 2 | | | 10.45587 | | 12.78293 | | Pyramids | |
| 658 | | -1 | | 545.0925 | | | 1 | | 20.44991 | | 814.3281 | | 84.99767 | | Patch 35 | | 209.6211 | | 2787.588 | mixed; pyramids, lpm | | pyramids, low profile | 16MX | | MX | | mid | 7 | | 2 | | | 7.300955 | | 0.545092 | | Pyramids | |
| 662 | | -1 | | 267.0073 | | | 1 | | 18.70608 | | 205.8014 | | 76.30132 | | Patch 21 | | 220.0517 | | 2852.056 | mixed; pyramids, lpm | | pyramids, low profile | 16MX | | MX | | mid | 7 | | 2 | | | 6.587276 | | 0.267007 | | Pyramids | |
| 663 | | -1 | | 2369.248 | | | 1 | | 19.16589 | | 100.0765 | | 108.7111 | | Patch 21 | | 220.0517 | | 2852.056 | mixed; pyramids, lpm | | pyramids, low profile | 16MX | | MX | | mid | 7 | | 2 | | | 8.770328 | | 2.369248 | | Pyramids | |
| 737 | | -1 | | 2350.286 | | | 1 | | 19.99942 | | 296.769 | | 87.48091 | | Patch 48 | | 194.3671 | | 2223.669 | mixed; pyramids, lpm | | pyramids, low profile | 16MX | | MX | | mid | 7 | | 2 | | | 8.762292 | | 2.350286 | | Pyramids | |
| 747 | | -1 | | 28.26721 | | | 1 | | 20.47902 | | 227.0061 | | 68.64721 | | Patch 5 | | 177.1536 | | 2255.842 | mixed; pyramids, lpm | | pyramids, low profile | 16MX | | MX | | mid | 7 | | 2 | | | 4.341702 | | 0.028267 | | Pyramids | |
| 753 | | -1 | | 34.05175 | | | 2 | | 20.10186 | | 223.1692 | | 67.47412 | | Patch 5 | | 177.1536 | | 2255.842 | mixed; pyramids, lpm | | pyramids, low profile | 16MX | | MX | | mid | 7 | | 2 | | | 4.527881 | | 0.034052 | | Pyramids | |
| 796 | | -1 | | 1325.476 | | | 66 | | 18.77761 | | 215.1508 | | 111.1193 | | Patch 21 | | 220.0517 | | 2852.056 | mixed; pyramids, lpm | | pyramids, low profile | 16MX | | MX | | mid | 7 | | 2 | | | 8.189527 | | 1.325476 | | Pyramids | |
| 166 | | 166 | | 12030.53 | | | 6 | | 20.58227 | | 743.4483 | | 28.49507 | | Patch 20 | | 245.084 | | 2535.82 | pyramids | | pyramids | 16PY | | PY | | mid | 7 | | 1 | | | 10.3952 | | 12.03053 | | Pyramids | |
| 173 | | 173 | | 18319.07 | | | 6 | | 19.7501 | | 389.4104 | | 16.89192 | | Patch 27 | | 217.3438 | | 2298.087 | pyramids | | pyramids | 16PY | | PY | | mid | 7 | | 1 | | | 10.8157 | | 18.31907 | | Pyramids | |
| 174 | | 174 | | 15635.55 | | | 3 | | 20.05146 | | 772.6909 | | 35.41487 | | Patch 29 | | 177.3662 | | 1658.73 | pyramids | | pyramids | 16PY | | PY | | mid | 7 | | 1 | | | 10.6573 | | 15.63555 | | Pyramids | |
| 184 | | 184 | | 16223.5 | | | 14 | | 18.91974 | | 212.1281 | | 45.8182 | | Patch 41 | | 191.464 | | 2212.923 | pyramids | | pyramids | 16PY | | PY | | mid | 7 | | 1 | | | 10.69422 | | 16.2235 | | Pyramids | |
| 217 | | 217 | | 39574.54 | | | 25 | | 19.76391 | | 392.9029 | | 49.92301 | | Patch 8 | | 188.1709 | | 2390.707 | pyramids | | pyramids | 16PY | | PY | | mid | 7 | | 1 | | | 11.58594 | | 39.57454 | | Pyramids | |
| 218 | | 218 | | 28445.75 | | | 61.77809 | | 20.08064 | | 380.1202 | | 13.34276 | | Patch 8 | | 188.1709 | | 2390.707 | pyramids | | pyramids | 16PY | | PY | | mid | 7 | | 1 | | | 11.25575 | | 28.44575 | | Pyramids | |
| 317 | | 317 | | 15078.67 | | | 5 | | 20.18522 | | 773.3993 | | 63.49952 | | Patch 20 | | 245.084 | | 2535.82 | pyramids | | pyramids | 16PY | | PY | | mid | 7 | | 1 | | | 10.62104 | | 15.07867 | | Pyramids | |
| 319 | | 319 | | 17398.54 | | | 43 | | 19.30625 | | 166.7113 | | 87.36599 | | Patch 41 | | 191.464 | | 2212.923 | pyramids | | pyramids | 16PY | | PY | | mid | 7 | | 1 | | | 10.76414 | | 17.39854 | | Pyramids | |
| 324 | | 324 | | 31369.88 | | | 1344 | | 19.20366 | | 463.7834 | | 109.147 | | Patch 8 | | 188.1709 | | 2390.707 | pyramids | | pyramids | 16PY | | PY | | mid | 7 | | 1 | | | 11.3536 | | 31.36988 | | Pyramids | |
| 388 | | 388 | | 2378.077 | | | 2 | | 19.30039 | | 427.2631 | | 54.19663 | | Patch 27 | | 217.3438 | | 2298.087 | pyramids | | pyramids | 16PY | | PY | | mid | 7 | | 1 | | | 8.774048 | | 2.378077 | | Pyramids | |
| 513 | | -1 | | 917.6611 | | | 1 | | 20.67227 | | 743.0278 | | 15.25101 | | Patch 20 | | 245.084 | | 2535.82 | pyramids | | pyramids | 16PY | | PY | | mid | 7 | | 1 | | | 7.821828 | | 0.917661 | | Pyramids | |
| 525 | | -1 | | 4322.714 | | | 1 | | 20.04511 | | 219.9153 | | 19.37675 | | Patch 41 | | 191.464 | | 2212.923 | pyramids | | pyramids | 16PY | | PY | | mid | 7 | | 1 | | | 9.371639 | | 4.322714 | | Pyramids | |
| 526 | | -1 | | 61.32698 | | | 3.066349 | | 19.30456 | | 241.4584 | | 44.43104 | | Patch 41 | | 191.464 | | 2212.923 | pyramids | | pyramids | 16PY | | PY | | mid | 7 | | 1 | | | 5.11622 | | 0.061327 | | Pyramids | |
| 639 | | -1 | | 4743.201 | | | 1 | | 20.28142 | | 805.1825 | | 69.82584 | | Patch 20 | | 245.084 | | 2535.82 | pyramids | | pyramids | 16PY | | PY | | mid | 7 | | 1 | | | 9.464467 | | 4.743201 | | Pyramids | |
| 654 | | -1 | | 1460.623 | | | 1 | | 20.38381 | | 832.5281 | | 101.2122 | | Patch 20 | | 245.084 | | 2535.82 | pyramids | | pyramids | 16PY | | PY | | mid | 7 | | 1 | | | 8.286619 | | 1.460623 | | Pyramids | |
| 664 | | -1 | | 68.41658 | | | 1 | | 19.19213 | | 390.887 | | 67.19131 | | Patch 27 | | 217.3438 | | 2298.087 | pyramids | | pyramids | 16PY | | PY | | mid | 7 | | 1 | | | 5.225615 | | 0.068417 | | Pyramids | |
| 702 | | -1 | | 344.1024 | | | 1 | | 19.49567 | | 405.0934 | | 33.47846 | | Patch 27 | | 217.3438 | | 2298.087 | pyramids | | pyramids | 16PY | | PY | | mid | 7 | | 1 | | | 6.840939 | | 0.344102 | | Pyramids | |
| 744 | | -1 | | 22.41657 | | | 1 | | 19.25272 | | 314.907 | | 78.14535 | | Patch 8 | | 188.1709 | | 2390.707 | pyramids | | pyramids | 16PY | | PY | | mid | 7 | | 1 | | | 4.1098 | | 0.022417 | | Pyramids | |
| 756 | | -1 | | 39.23937 | | | 2 | | 20.03635 | | 687.175 | | 93.36705 | | Patch 20 | | 245.084 | | 2535.82 | pyramids | | pyramids | 16PY | | PY | | mid | 7 | | 1 | | | 4.66968 | | 0.039239 | | Pyramids | |
| 763 | | -1 | | 48.66507 | | | 2 | | 20.18186 | | 706.7661 | | 68.57991 | | Patch 20 | | 245.084 | | 2535.82 | pyramids | | pyramids | 16PY | | PY | | mid | 7 | | 1 | | | 4.884961 | | 0.048665 | | Pyramids | |
| 778 | | -1 | | 110.9859 | | | 6 | | 20.30856 | | 815.5747 | | 75.68116 | | Patch 20 | | 245.084 | | 2535.82 | pyramids | | pyramids | 16PY | | PY | | mid | 7 | | 1 | | | 5.709403 | | 0.110986 | | Pyramids | |
| 785 | | -1 | | 215.3476 | | | 11 | | 19.77135 | | 444.106 | | 112.1083 | | Patch 8 | | 188.1709 | | 2390.707 | pyramids | | pyramids | 16PY | | PY | | mid | 7 | | 1 | | | 6.372254 | | 0.215348 | | Pyramids | |
| 17 | | 17 | | 8085.926 | | | 2 | | 21.55264 | | 239.001 | | 34.55201 | | 1-8 Block37 | | 12.39696 | | 9.507539 | 1 8 in palet cinderblocks | | cinderblocks | 1CB | | CB | | low | 7 | | 1 | | | 9.99788 | | 8.085926 | | Cinderblocks | |
| 415 | | -1 | | 835.7519 | | | 1 | | 22.10071 | | 304.1162 | | 35.10221 | | 1-6 Block16 | | 22.02182 | | 18.58669 | 1 6 in palet cinderblocks | | cinderblocks | 1CB | | CB | | low | 7 | | 1 | | | 7.728332 | | 0.835752 | | Cinderblocks | |
| 416 | | -1 | | 3112.788 | | | 1 | | 22.47386 | | 522.1066 | | 29.42901 | | 1-6 Block19 | | 58.33563 | | 118.1137 | 1 6 in palet cinderblocks | | cinderblocks | 1CB | | CB | | low | 7 | | 1 | | | 9.043274 | | 3.112788 | | Cinderblocks | |
| 419 | | -1 | | 911.4124 | | | 1 | | 21.72273 | | 234.1245 | | 5.100569 | | 1-8 Block37 | | 12.39696 | | 9.507539 | 1 8 in palet cinderblocks | | cinderblocks | 1CB | | CB | | low | 7 | | 1 | | | 7.814995 | | 0.911412 | | Cinderblocks | |
| 422 | | -1 | | 2047.018 | | | 1 | | 21.69216 | | 209.6031 | | 42.084 | | 1-8 Block37 | | 12.39696 | | 9.507539 | 1 8 in palet cinderblocks | | cinderblocks | 1CB | | CB | | low | 7 | | 1 | | | 8.624139 | | 2.047018 | | Cinderblocks | |
| 165 | | 165 | | 6284.105 | | | 4 | | 20.02347 | | 729.7345 | | 9.518752 | | Patch 19 | | 12.86559 | | 7.884214 | mixed; pyramids, lpm | | pyramids, low profile | 1MX | | MX | | mid | 7 | | 2 | | | 9.745779 | | 6.284105 | | Pyramids | |
| 176 | | 176 | | 6269.445 | | | 3 | | 19.37517 | | 207.8105 | | 8.27682 | | Patch 31 | | 21.22518 | | 28.07035 | mixed; pyramids, lpm | | pyramids, low profile | 1MX | | MX | | mid | 7 | | 2 | | | 9.743443 | | 6.269445 | | Pyramids | |
| 194 | | 194 | | 1270.46 | | | 2 | | 21.12715 | | 623.3404 | | 15.59845 | | Patch 51 | | 32.77326 | | 54.36816 | mixed; pyramids, lpm | | pyramids, low profile | 1MX | | MX | | mid | 7 | | 2 | | | 8.147134 | | 1.27046 | | Pyramids | |
| 195 | | 195 | | 3309.208 | | | 3 | | 20.9858 | | 641.3203 | | 9.179553 | | Patch 51 | | 32.77326 | | 54.36816 | mixed; pyramids, lpm | | pyramids, low profile | 1MX | | MX | | mid | 7 | | 2 | | | 9.104464 | | 3.309208 | | Pyramids | |
| 207 | | 207 | | 19443.49 | | | 13 | | 18.91784 | | 184.0138 | | 28.43848 | | Patch 6 | | 49.82873 | | 107.0489 | mixed; pyramids, lpm | | pyramids, low profile | 1MX | | MX | | mid | 7 | | 2 | | | 10.87527 | | 19.44349 | | Pyramids | |
| 330 | | 330 | | 4465.25 | | | 2 | | 18.93042 | | 61.11458 | | 152.8975 | | Patch 6 | | 49.82873 | | 107.0489 | mixed; pyramids, lpm | | pyramids, low profile | 1MX | | MX | | mid | 7 | | 2 | | | 9.40408 | | 4.46525 | | Pyramids | |
| 389 | | 389 | | 70850.42 | | | 2 | | 18.41261 | | 212.4721 | | 57.72144 | | Patch 6 | | 49.82873 | | 107.0489 | mixed; pyramids, lpm | | pyramids, low profile | 1MX | | MX | | mid | 7 | | 2 | | | 12.16833 | | 70.85042 | | Pyramids | |
| 401 | | 401 | | 256.4811 | | | 2 | | 20.65069 | | 717.3814 | | 94.79242 | | Patch 51 | | 32.77326 | | 54.36816 | mixed; pyramids, lpm | | pyramids, low profile | 1MX | | MX | | mid | 7 | | 2 | | | 6.547055 | | 0.256481 | | Pyramids | |
| 406 | | 406 | | 1503.919 | | | 74 | | 18.78608 | | 84.28666 | | 143.5963 | | Patch 6 | | 49.82873 | | 107.0489 | mixed; pyramids, lpm | | pyramids, low profile | 1MX | | MX | | mid | 7 | | 2 | | | 8.31583 | | 1.503919 | | Pyramids | |
| 641 | | -1 | | 1306.964 | | | 1 | | 19.20058 | | 206.4393 | | 14.25305 | | Patch 31 | | 21.22518 | | 28.07035 | mixed; pyramids, lpm | | pyramids, low profile | 1MX | | MX | | mid | 7 | | 2 | | | 8.175462 | | 1.306964 | | Pyramids | |
| 648 | | -1 | | 3316.34 | | | 1 | | 19.07345 | | 63.27058 | | 139.7653 | | Patch 6 | | 49.82873 | | 107.0489 | mixed; pyramids, lpm | | pyramids, low profile | 1MX | | MX | | mid | 7 | | 2 | | | 9.106617 | | 3.31634 | | Pyramids | |
| 657 | | -1 | | 1120.214 | | | 1 | | 19.33563 | | 156.8746 | | 51.54537 | | Patch 31 | | 21.22518 | | 28.07035 | mixed; pyramids, lpm | | pyramids, low profile | 1MX | | MX | | mid | 7 | | 2 | | | 8.021275 | | 1.120214 | | Pyramids | |
| 659 | | -1 | | 3911.386 | | | 1 | | 19.7475 | | 634.1698 | | 84.13816 | | Patch 39 | | 22.2182 | | 28.94309 | mixed; pyramids, lpm | | pyramids, low profile | 1MX | | MX | | mid | 7 | | 2 | | | 9.271647 | | 3.911386 | | Pyramids | |
| 726 | | -1 | | 2881.179 | | | 1 | | 20.71692 | | 705.5488 | | 103.3278 | | Patch 51 | | 32.77326 | | 54.36816 | mixed; pyramids, lpm | | pyramids, low profile | 1MX | | MX | | mid | 7 | | 2 | | | 8.965955 | | 2.881179 | | Pyramids | |
| 749 | | -1 | | 30.80977 | | | 2 | | 19.95701 | | 649.7505 | | 91.38791 | | Patch 19 | | 12.86559 | | 7.884214 | mixed; pyramids, lpm | | pyramids, low profile | 1MX | | MX | | mid | 7 | | 2 | | | 4.427832 | | 0.03081 | | Pyramids | |
| 758 | | -1 | | 39.9339 | | | 2 | | 19.99945 | | 642.8092 | | 34.80913 | | Patch 39 | | 22.2182 | | 28.94309 | mixed; pyramids, lpm | | pyramids, low profile | 1MX | | MX | | mid | 7 | | 2 | | | 4.687226 | | 0.039934 | | Pyramids | |
| 761 | | -1 | | 46.73662 | | | 2 | | 18.96405 | | 173.3524 | | 47.66076 | | Patch 31 | | 21.22518 | | 28.07035 | mixed; pyramids, lpm | | pyramids, low profile | 1MX | | MX | | mid | 7 | | 2 | | | 4.844528 | | 0.046737 | | Pyramids | |
| 767 | | -1 | | 67.82277 | | | 3 | | 19.98692 | | 645.5899 | | 111.4897 | | Patch 19 | | 12.86559 | | 7.884214 | mixed; pyramids, lpm | | pyramids, low profile | 1MX | | MX | | mid | 7 | | 2 | | | 5.216898 | | 0.067823 | | Pyramids | |
| 803 | | -1 | | 9922.108 | | | 496 | | 19.15654 | | 269.9988 | | 71.63205 | | Patch 31 | | 21.22518 | | 28.07035 | mixed; pyramids, lpm | | pyramids, low profile | 1MX | | MX | | mid | 7 | | 2 | | | 10.20252 | | 9.922108 | | Pyramids | |
| 804 | | -1 | | 26981.71 | | | 1349 | | 19.19378 | | 77.329 | | 118.1285 | | Patch 6 | | 49.82873 | | 107.0489 | mixed; pyramids, lpm | | pyramids, low profile | 1MX | | MX | | mid | 7 | | 2 | | | 11.20291 | | 26.98171 | | Pyramids | |
| 805 | | -1 | | 37800.74 | | | 1890 | | 19.15 | | 139.9181 | | 103.6546 | | Patch 31 | | 21.22518 | | 28.07035 | mixed; pyramids, lpm | | pyramids, low profile | 1MX | | MX | | mid | 7 | | 2 | | | 11.54008 | | 37.80074 | | Pyramids | |
| 152 | | 152 | | 10097.96 | | | 5 | | 19.98707 | | 586.7247 | | 47.4645 | | Patch 14 | | 13.59093 | | 9.816945 | single pyramid | | pyramid | 1PY | | PY | | mid | 7 | | 1 | | | 10.22009 | | 10.09796 | | Pyramids | |
| 153 | | 153 | | 11669.65 | | | 4 | | 20.19976 | | 570.3249 | | 26.38403 | | Patch 14 | | 13.59093 | | 9.816945 | single pyramid | | pyramid | 1PY | | PY | | mid | 7 | | 1 | | | 10.36475 | | 11.66965 | | Pyramids | |
| 186 | | 186 | | 5233.234 | | | 82.53706 | | 19.30984 | | 201.1655 | | 2.40885 | | Patch 46 | | 14.85826 | | 12.65756 | single pyramid | | pyramid | 1PY | | PY | | mid | 7 | | 1 | | | 9.562785 | | 5.233234 | | Pyramids | |
| 316 | | 316 | | 3389.913 | | | 152 | | 19.11147 | | 165.6587 | | 57.0602 | | Patch 46 | | 14.85826 | | 12.65756 | single pyramid | | pyramid | 1PY | | PY | | mid | 7 | | 1 | | | 9.12856 | | 3.389913 | | Pyramids | |
| 318 | | 318 | | 2267.452 | | | 11 | | 18.98205 | | 227.6407 | | 30.46144 | | Patch 2 | | 12.30763 | | 7.169271 | single pyramid | | pyramid | 1PY | | PY | | mid | 7 | | 1 | | | 8.726412 | | 2.267452 | | Pyramids | |
| 338 | | 338 | | 5584.415 | | | 3 | | 19.07694 | | 183.6002 | | 72.78673 | | Patch 46 | | 14.85826 | | 12.65756 | single pyramid | | pyramid | 1PY | | PY | | mid | 7 | | 1 | | | 9.627735 | | 5.584415 | | Pyramids | |
| 400 | | 400 | | 6527.948 | | | 3 | | 21.38538 | | 315.5371 | | 56.38775 | | Patch 63 | | 11.33588 | | 7.799469 | single pyramid | | pyramid | 1PY | | PY | | mid | 7 | | 1 | | | 9.783848 | | 6.527948 | | Pyramids | |
| 511 | | -1 | | 35.38238 | | | 1.769119 | | 19.90534 | | 545.4311 | | 1.393513 | | Patch 14 | | 13.59093 | | 9.816945 | single pyramid | | pyramid | 1PY | | PY | | mid | 7 | | 1 | | | 4.566214 | | 0.035382 | | Pyramids | |
| 512 | | -1 | | 86.25275 | | | 4.312638 | | 20.00007 | | 593.5906 | | 49.5611 | | Patch 14 | | 13.59093 | | 9.816945 | single pyramid | | pyramid | 1PY | | PY | | mid | 7 | | 1 | | | 5.457282 | | 0.086253 | | Pyramids | |
| 530 | | -1 | | 300.0401 | | | 1 | | 19.08082 | | 256.813 | | 70.03961 | | Patch 46 | | 14.85826 | | 12.65756 | single pyramid | | pyramid | 1PY | | PY | | mid | 7 | | 1 | | | 6.703916 | | 0.30004 | | Pyramids | |
| 637 | | -1 | | 1914.145 | | | 1 | | 19.23574 | | 84.24436 | | 131.0207 | | Patch 46 | | 14.85826 | | 12.65756 | single pyramid | | pyramid | 1PY | | PY | | mid | 7 | | 1 | | | 8.557026 | | 1.914145 | | Pyramids | |
| 646 | | -1 | | 773.598 | | | 1 | | 19.79813 | | 608.0877 | | 64.04936 | | Patch 14 | | 13.59093 | | 9.816945 | single pyramid | | pyramid | 1PY | | PY | | mid | 7 | | 1 | | | 7.651052 | | 0.773598 | | Pyramids | |
| 660 | | -1 | | 241.7253 | | | 1 | | 20.42504 | | 629.6973 | | 48.73367 | | Patch 40 | | 16.73666 | | 19.63504 | single pyramid | | pyramids | 1PY | | PY | | mid | 7 | | 1 | | | 6.487802 | | 0.241725 | | Pyramids | |
| 661 | | -1 | | 784.8236 | | | 1 | | 20.55847 | | 641.7105 | | 14.1973 | | Patch 40 | | 16.73666 | | 19.63504 | single pyramid | | pyramids | 1PY | | PY | | mid | 7 | | 1 | | | 7.665459 | | 0.784824 | | Pyramids | |
| 743 | | -1 | | 21.24945 | | | 1 | | 19.00781 | | 164.5916 | | 35.35411 | | Patch 46 | | 14.85826 | | 12.65756 | single pyramid | | pyramid | 1PY | | PY | | mid | 7 | | 1 | | | 4.056331 | | 0.021249 | | Pyramids | |
| 751 | | -1 | | 31.20082 | | | 2 | | 20.07341 | | 498.0477 | | 92.17307 | | Patch 14 | | 13.59093 | | 9.816945 | single pyramid | | pyramid | 1PY | | PY | | mid | 7 | | 1 | | | 4.440445 | | 0.031201 | | Pyramids | |
| 757 | | -1 | | 39.75531 | | | 2 | | 19.9523 | | 600.2209 | | 68.08018 | | Patch 14 | | 13.59093 | | 9.816945 | single pyramid | | pyramid | 1PY | | PY | | mid | 7 | | 1 | | | 4.682743 | | 0.039755 | | Pyramids | |
| 765 | | -1 | | 60.82621 | | | 3 | | 19.56211 | | 451.4929 | | 73.60229 | | Patch 42 | | 13.50734 | | 11.18158 | single pyramid | | pyramids | 1PY | | PY | | mid | 7 | | 1 | | | 5.108021 | | 0.060826 | | Pyramids | |
| 768 | | -1 | | 69.3813 | | | 3 | | 20.29314 | | 637.0214 | | 29.24178 | | Patch 40 | | 16.73666 | | 19.63504 | single pyramid | | pyramids | 1PY | | PY | | mid | 7 | | 1 | | | 5.239617 | | 0.069381 | | Pyramids | |
| 777 | | -1 | | 102.7869 | | | 5 | | 19.14659 | | 275.9667 | | 107.3548 | | Patch 2 | | 12.30763 | | 7.169271 | single pyramid | | pyramid | 1PY | | PY | | mid | 7 | | 1 | | | 5.632658 | | 0.102787 | | Pyramids | |
| 782 | | -1 | | 173.1656 | | | 9 | | 20.03206 | | 480.1245 | | 95.35986 | | Patch 14 | | 13.59093 | | 9.816945 | single pyramid | | pyramid | 1PY | | PY | | mid | 7 | | 1 | | | 6.154248 | | 0.173166 | | Pyramids | |
| 786 | | -1 | | 246.6854 | | | 12 | | 20.04661 | | 614.8285 | | 89.99475 | | Patch 14 | | 13.59093 | | 9.816945 | single pyramid | | pyramid | 1PY | | PY | | mid | 7 | | 1 | | | 6.508114 | | 0.246685 | | Pyramids | |
| 788 | | -1 | | 281.1635 | | | 14 | | 19.0363 | | 253.1044 | | 73.82682 | | Patch 2 | | 12.30763 | | 7.169271 | single pyramid | | pyramid | 1PY | | PY | | mid | 7 | | 1 | | | 6.638936 | | 0.281163 | | Pyramids | |
| 794 | | -1 | | 1046.683 | | | 52 | | 19.14711 | | 252.301 | | 93.513 | | Patch 46 | | 14.85826 | | 12.65756 | single pyramid | | pyramid | 1PY | | PY | | mid | 7 | | 1 | | | 7.953381 | | 1.046683 | | Pyramids | |
| 795 | | -1 | | 1299.768 | | | 65 | | 19.07109 | | 100.0008 | | 118.194 | | Patch 2 | | 12.30763 | | 7.169271 | single pyramid | | pyramid | 1PY | | PY | | mid | 7 | | 1 | | | 8.169941 | | 1.299768 | | Pyramids | |
| 801 | | -1 | | 5656.83 | | | 283 | | 19.05857 | | 99.45455 | | 104.417 | | Patch 2 | | 12.30763 | | 7.169271 | single pyramid | | pyramid | 1PY | | PY | | mid | 7 | | 1 | | | 9.640619 | | 5.65683 | | Pyramids | |
| 411 | | -1 | | 13183.77 | | | 659.1884 | | 22.43608 | | 703.7244 | | 5.082406 | | 200 | | 135.0497 | | 1189.699 | 200 rr tie | | rr ties | 200T RR | | RR | | mid-high | 7 | | 1 | | | 10.48674 | | 13.18377 | | RR Ties | |
| 367 | | 367 | | 29678.54 | | | 3 | | 22.26358 | | 813.6409 | | 34.85908 | | 25.1 | | 87.86015 | | 337.9296 | 25 rr tie | | rr ties | 25T RR | | RR | | low-mid | 7 | | 1 | | | 11.29818 | | 29.67854 | | RR Ties | |
| 409 | | -1 | | 3296.228 | | | 164.8114 | | 22.05235 | | 946.5974 | | 17.17352 | | 25.2 | | 110.5575 | | 827.4146 | 25 rr tie | | rr ties | 25T RR | | RR | | low-mid | 7 | | 1 | | | 9.100534 | | 3.296228 | | RR Ties | |
| 636 | | -1 | | 229.8904 | | | 1 | | 21.75468 | | 925.2185 | | 24.97021 | | 25.2 | | 110.5575 | | 827.4146 | 25 rr tie | | rr ties | 25T RR | | RR | | low-mid | 7 | | 1 | | | 6.437603 | | 0.22989 | | RR Ties | |
| 675 | | -1 | | 127.103 | | | 1 | | 22.42964 | | 828.1381 | | 6.525615 | | 25.1 | | 87.86015 | | 337.9296 | 25 rr tie | | rr ties | 25T RR | | RR | | low-mid | 7 | | 1 | | | 5.844998 | | 0.127103 | | RR Ties | |
| 71 | | 71 | | 4127.463 | | | 5 | | 22.59431 | | 574.5072 | | 23.33429 | | Concrete2 ton12 | | 12.70231 | | 8.784523 | 2 ton concrete | | concrete | 2T Concrete | | Concrete | | low | 7 | | 1 | | | 9.325418 | | 4.127463 | | Concrete | |
| 72 | | 72 | | 5275.303 | | | 2 | | 23.22489 | | 187.7079 | | 12.8092 | | Concrete2 ton3 | | 54.23263 | | 164.6667 | 2 ton concrete | | concrete | 2T Concrete | | Concrete | | low | 7 | | 1 | | | 9.570791 | | 5.275303 | | Concrete | |
| 73 | | 73 | | 1827.183 | | | 2 | | 21.98352 | | 362.5061 | | 6.415792 | | Concrete2 ton7 | | 54.89791 | | 76.6872 | 2 ton concrete | | concrete | 2T Concrete | | Concrete | | low | 7 | | 1 | | | 8.510531 | | 1.827183 | | Concrete | |
| 467 | | -1 | | 2165.522 | | | 1 | | 20.66373 | | 477.2735 | | 16.34809 | | Concrete2 ton10 | | 20.64839 | | 14.3246 | 2 ton concrete | | concrete | 2T Concrete | | Concrete | | low | 7 | | 1 | | | 8.680417 | | 2.165522 | | Concrete | |
| 468 | | -1 | | 3964.3 | | | 1 | | 20.69682 | | 466.3907 | | 28.89583 | | Concrete2 ton10 | | 20.64839 | | 14.3246 | 2 ton concrete | | concrete | 2T Concrete | | Concrete | | low | 7 | | 1 | | | 9.285085 | | 3.9643 | | Concrete | |
| 469 | | -1 | | 23.9497 | | | 1.197485 | | 20.88533 | | 786.5237 | | 9.982599 | | Concrete2 ton11 | | 17.6993 | | 19.65189 | 2 ton concrete | | concrete | 2T Concrete | | Concrete | | low | 7 | | 1 | | | 4.175956 | | 0.02395 | | Concrete | |
| 470 | | -1 | | 1750.014 | | | 1 | | 22.28927 | | 447.097 | | 12.89565 | | Concrete2 ton6 | | 29.66684 | | 48.396 | 2 ton concrete | | concrete | 2T Concrete | | Concrete | | low | 7 | | 1 | | | 8.467379 | | 1.750014 | | Concrete | |
| 471 | | -1 | | 2000.963 | | | 1 | | 22.2999 | | 457.4123 | | 9.363694 | | Concrete2 ton6 | | 29.66684 | | 48.396 | 2 ton concrete | | concrete | 2T Concrete | | Concrete | | low | 7 | | 1 | | | 8.601384 | | 2.000963 | | Concrete | |
| 472 | | -1 | | 3194.055 | | | 1 | | 21.83604 | | 369.0995 | | 13.99558 | | Concrete2 ton7 | | 54.89791 | | 76.6872 | 2 ton concrete | | concrete | 2T Concrete | | Concrete | | low | 7 | | 1 | | | 9.069046 | | 3.194055 | | Concrete | |
| 733 | | -1 | | 2664.906 | | | 1 | | 21.79252 | | 349.718 | | 45.22052 | | Concrete2 ton7 | | 54.89791 | | 76.6872 | 2 ton concrete | | concrete | 2T Concrete | | Concrete | | low | 7 | | 1 | | | 8.887924 | | 2.664906 | | Concrete | |
| 762 | | -1 | | 47.20598 | | | 2 | | 20.8475 | | 811.9651 | | 54.30028 | | Concrete2 ton11 | | 17.6993 | | 19.65189 | 2 ton concrete | | concrete | 2T Concrete | | Concrete | | low | 7 | | 1 | | | 4.854521 | | 0.047206 | | Concrete | |
| 39 | | 39 | | 16125.62 | | | 2 | | 21.50017 | | 978.9406 | | 4.81853 | | 30-mixed Block3 | | 68.18759 | | 323.7249 | 30 pallets mixed size cinderblocks | | cinderblocks | 30CB | | CB | | low-mid | 7 | | 1 | | | 10.68816 | | 16.12562 | | Cinderblocks | |
| 429 | | -1 | | 1890.233 | | | 1 | | 21.8344 | | 902.4608 | | 27.73404 | | 30-mixed Block1 | | 90.906 | | 564.317 | 30 pallets mixed sized cinderblocks | | cinderblocks | 30CB | | CB | | low-mid | 7 | | 1 | | | 8.544455 | | 1.890233 | | Cinderblocks | |
| 430 | | -1 | | 10680.1 | | | 1 | | 21.37684 | | 1003.83 | | 21.79767 | | 30-mixed Block3 | | 68.18759 | | 323.7249 | 30 pallets mixed size cinderblocks | | cinderblocks | 30CB | | CB | | low-mid | 7 | | 1 | | | 10.27614 | | 10.6801 | | Cinderblocks | |
| 448 | | -1 | | 1324.913 | | | 1 | | 21.55875 | | 974.4589 | | 22.51373 | | 30-mixed Block3 | | 68.18759 | | 323.7249 | 30 pallets mixed size cinderblocks | | cinderblocks | 30CB | | CB | | low-mid | 7 | | 1 | | | 8.189102 | | 1.324913 | | Cinderblocks | |
| 449 | | -1 | | 3727.932 | | | 1 | | 21.43313 | | 985.015 | | 16.17009 | | 30-mixed Block3 | | 68.18759 | | 323.7249 | 30 pallets mixed size cinderblocks | | cinderblocks | 30CB | | CB | | low-mid | 7 | | 1 | | | 9.223609 | | 3.727932 | | Cinderblocks | |
| 40 | | 40 | | 686.8596 | | | 2 | | 20.03449 | | 99.34195 | | 9.138126 | | 3-4 Block2 | | 24.75255 | | 33.85146 | 3 4 in palet cinderblocks | | cinderblocks | 3CB | | CB | | low | 7 | | 1 | | | 7.53213 | | 0.68686 | | Cinderblocks | |
| 41 | | 41 | | 7767.483 | | | 3 | | 22.87533 | | 325.6882 | | 17.14951 | | 3-4 Block6 | | 29.09947 | | 52.44356 | 3 4 in palet cinderblocks | | cinderblocks | 3CB | | CB | | low | 7 | | 1 | | | 9.957701 | | 7.767483 | | Cinderblocks | |
| 42 | | 42 | | 6789.283 | | | 5 | | 22.04528 | | 1032.589 | | 28.18529 | | 3-8 Block14 | | 60.69355 | | 222.6086 | 3 8 in pallets cinderblocks | | cinderblocks | 3CB | | CB | | low | 7 | | 1 | | | 9.823101 | | 6.789283 | | Cinderblocks | |
| 43 | | 43 | | 29696.11 | | | 26 | | 22.50327 | | 541.0446 | | 63.87367 | | 3-6 Block6 | | 83.61275 | | 298.1626 | 3 6 in pallets cinderblocks | | cinderblocks | 3CB | | CB | | low | 7 | | 1 | | | 11.29877 | | 29.69611 | | Cinderblocks | |
| 44 | | 44 | | 6069.035 | | | 4 | | 22.32813 | | 536.4231 | | 40.97929 | | 3-8 Block4 | | 42.30081 | | 120.303 | 3 8 in pallets cinderblocks | | cinderblocks | 3CB | | CB | | low | 7 | | 1 | | | 9.710955 | | 6.069035 | | Cinderblocks | |
| 45 | | 45 | | 2537.948 | | | 2 | | 22.21857 | | 529.7966 | | 94.52338 | | 3-8 Block4 | | 42.30081 | | 120.303 | 3 8 in pallets cinderblocks | | cinderblocks | 3CB | | CB | | low | 7 | | 1 | | | 8.839111 | | 2.537948 | | Cinderblocks | |
| 47 | | 47 | | 11834.57 | | | 7 | | 22.53675 | | 535.8602 | | 90.61049 | | 3-8 Block9 | | 66.83328 | | 280.6094 | 3 8 in pallets cinderblocks | | cinderblocks | 3CB | | CB | | low | 7 | | 1 | | | 10.37878 | | 11.83457 | | Cinderblocks | |
| 48 | | 48 | | 4104.1 | | | 8 | | 22.4957 | | 522.0947 | | 89.62586 | | 3-8 Block9 | | 66.83328 | | 280.6094 | 3 8 in pallets cinderblocks | | cinderblocks | 3CB | | CB | | low | 7 | | 1 | | | 9.319742 | | 4.1041 | | Cinderblocks | |
| 50 | | 50 | | 1980.778 | | | 2 | | 22.28384 | | 535.9144 | | 33.12377 | | 3-8 Block4 | | 42.30081 | | 120.303 | 3 8 in pallets cinderblocks | | cinderblocks | 3CB | | CB | | low | 7 | | 1 | | | 8.591245 | | 1.980778 | | Cinderblocks | |
| 52 | | 52 | | 10477.28 | | | 3 | | 21.49671 | | 1039.479 | | 18.33996 | | 3-mixed Block21 | | 37.05382 | | 98.80981 | 3 pallets mixed size ciderblocks | | cinderblocks | 3CB | | CB | | low | 7 | | 1 | | | 10.25696 | | 10.47728 | | Cinderblocks | |
| 53 | | 53 | | 18896.28 | | | 3 | | 21.29401 | | 802.5316 | | 14.68899 | | 3-mixed Block22 | | 33.51995 | | 78.91619 | 3 pallets mixed size ciderblocks | | cinderblocks | 3CB | | CB | | low | 7 | | 1 | | | 10.84672 | | 18.89628 | | Cinderblocks | |
| 55 | | 55 | | 4979.582 | | | 2 | | 21.70286 | | 817.5909 | | 3.273645 | | 3-mixed Block39 | | 29.46751 | | 47.73784 | 3 pallets mixed size ciderblocks | | cinderblocks | 3CB | | CB | | low | 7 | | 1 | | | 9.513101 | | 4.979582 | | Cinderblocks | |
| 234 | | 234 | | 1138.785 | | | 3 | | 22.64351 | | 600.2401 | | 86.21161 | | 3-6 Block5 | | 13.06464 | | 11.46195 | 3 6 in palet cinderblocks | | cinderblocks | 3CB | | CB | | low | 7 | | 1 | | | 8.037717 | | 1.138785 | | Cinderblocks | |
| 236 | | 236 | | 37936.39 | | | 24.2163 | | 22.7271 | | 641.1412 | | 81.15743 | | 3-6 Block5 | | 13.06464 | | 11.46195 | 3 6 in palet cinderblocks | | cinderblocks | 3CB | | CB | | low | 7 | | 1 | | | 11.54367 | | 37.93639 | | Cinderblocks | |
| 237 | | 237 | | 110388.2 | | | 405.3431 | | 22.12668 | | 750.069 | | 83.2606 | | 3-mixed Block17 | | 44.22191 | | 128.6117 | 3 pallets mixed size cinderblocks | | cinderblocks | 3CB | | CB | | low | 7 | | 1 | | | 12.61176 | | 110.3882 | | Cinderblocks | |
| 240 | | 240 | | 1968.805 | | | 2 | | 22.19851 | | 746.9513 | | 80.75921 | | 3-8 Block5 | | 55.30717 | | 140.0044 | 3 8 in palet cinderblocks | | cinderblocks | 3CB | | CB | | low | 7 | | 1 | | | 8.585182 | | 1.968805 | | Cinderblocks | |
| 246 | | 246 | | 50953.79 | | | 110.3866 | | 21.91304 | | 685.9167 | | 11.0387 | | 3-4 Block7 | | 22.43392 | | 25.57806 | 3 4 in palet cinderblocks | | cinderblocks | 3CB | | CB | | low | 7 | | 1 | | | 11.83867 | | 50.95379 | | Cinderblocks | |
| 300 | | 300 | | 2908.326 | | | 4 | | 22.21638 | | 655.427 | | 24.09495 | | 3-8 Block5 | | 55.30717 | | 140.0044 | 3 8 in palet cinderblocks | | cinderblocks | 3CB | | CB | | low | 7 | | 1 | | | 8.975333 | | 2.908326 | | Cinderblocks | |
| 315 | | 315 | | 21546.08 | | | 1031 | | 19.35636 | | 4.00077 | | 184.9023 | | 3-4 Block1 | | 14.41641 | | 12.80982 | 3 4 in palet cinderblocks | | cinderblocks | 3CB | | CB | | low | 7 | | 1 | | | 10.97795 | | 21.54608 | | Cinderblocks | |
| 336 | | 336 | | 11159.97 | | | 36 | | 20.16805 | | 67.22347 | | 32.19826 | | 3-4 Block2 | | 24.75255 | | 33.85146 | 3 4 in palet cinderblocks | | cinderblocks | 3CB | | CB | | low | 7 | | 1 | | | 10.32009 | | 11.15997 | | Cinderblocks | |
| 337 | | 337 | | 3692.652 | | | 2 | | 19.18818 | | 137.6163 | | 89.83412 | | 3-4 Block1 | | 14.41641 | | 12.80982 | 3 4 in palet cinderblocks | | cinderblocks | 3CB | | CB | | low | 7 | | 1 | | | 9.2141 | | 3.692652 | | Cinderblocks | |
| 358 | | 358 | | 170.6918 | | | 2 | | 21.85576 | | 677.7375 | | 17.35544 | | 3-mixed Block38 | | 32.69013 | | 55.83413 | 3 pallets mixed size ciderblocks | | cinderblocks | 3CB | | CB | | low | 7 | | 1 | | | 6.139859 | | 0.170692 | | Cinderblocks | |
| 359 | | 359 | | 428.5147 | | | 2 | | 22.09807 | | 732.9749 | | 39.54616 | | 3-4 Block7 | | 22.43392 | | 25.57806 | 3 4 in palet cinderblocks | | cinderblocks | 3CB | | CB | | low | 7 | | 1 | | | 7.060325 | | 0.428515 | | Cinderblocks | |
| 360 | | 360 | | 755.9186 | | | 2 | | 22.0318 | | 710.9747 | | 66.21158 | | 3-8 Block15 | | 42.74515 | | 77.57916 | 3 8 in palet cinderblocks | | cinderblocks | 3CB | | CB | | low | 7 | | 1 | | | 7.627934 | | 0.755919 | | Cinderblocks | |
| 366 | | 366 | | 3513.565 | | | 50 | | 21.78773 | | 736.3909 | | 75.08073 | | 3-mixed Block38 | | 32.69013 | | 55.83413 | 3 pallets mixed size ciderblocks | | cinderblocks | 3CB | | CB | | low | 7 | | 1 | | | 9.164387 | | 3.513565 | | Cinderblocks | |
| 370 | | 370 | | 4401.579 | | | 4 | | 22.72011 | | 555.6604 | | 62.15904 | | 3-6 Block5 | | 13.06464 | | 11.46195 | 3 6 in palet cinderblocks | | cinderblocks | 3CB | | CB | | low | 7 | | 1 | | | 9.389719 | | 4.401579 | | Cinderblocks | |
| 397 | | 397 | | 137.1343 | | | 2 | | 21.99815 | | 496.8355 | | 45.71329 | | 3-4 Block10 | | 16.86357 | | 21.12677 | 3 4 in palet cinderblocks | | cinderblocks | 3CB | | CB | | low | 7 | | 1 | | | 5.920961 | | 0.137134 | | Cinderblocks | |
| 431 | | -1 | | 3317.084 | | | 1 | | 21.99159 | | 445.3654 | | 16.88792 | | 3-4 Block10 | | 16.86357 | | 21.12677 | 3 4 in palet cinderblocks | | cinderblocks | 3CB | | CB | | low | 7 | | 1 | | | 9.106841 | | 3.317084 | | Cinderblocks | |
| 432 | | -1 | | 1063.544 | | | 1 | | 22.12824 | | 595.7934 | | 56.07933 | | 3-8 Block4 | | 42.30081 | | 120.303 | 3 8 in pallets cinderblocks | | cinderblocks | 3CB | | CB | | low | 7 | | 1 | | | 7.969362 | | 1.063544 | | Cinderblocks | |
| 433 | | -1 | | 1438.096 | | | 1 | | 22.99101 | | 373.0847 | | 6.344448 | | 3-4 Block5 | | 33.98552 | | 68.77628 | 3 4 in palet cinderblocks | | cinderblocks | 3CB | | CB | | low | 7 | | 1 | | | 8.271075 | | 1.438096 | | Cinderblocks | |
| 434 | | -1 | | 1151.055 | | | 1 | | 22.86916 | | 354.6317 | | 23.56844 | | 3-4 Block8 | | 24.70665 | | 43.63768 | 3 4 in palet cinderblocks | | cinderblocks | 3CB | | CB | | low | 7 | | 1 | | | 8.048434 | | 1.151055 | | Cinderblocks | |
| 435 | | -1 | | 1275.922 | | | 1 | | 21.75564 | | 396.0064 | | 22.30349 | | 3-6 Block10 | | 65.14892 | | 214.6843 | 3 6 in palet cinderblocks | | cinderblocks | 3CB | | CB | | low | 7 | | 1 | | | 8.151424 | | 1.275922 | | Cinderblocks | |
| 436 | | -1 | | 44.60028 | | | 2.230014 | | 20.9258 | | 101.3308 | | 8.906478 | | 3-6 Block2 | | 88.98341 | | 310.1671 | 3 6 in palet cinderblocks | | cinderblocks | 3CB | | CB | | low | 7 | | 1 | | | 4.79774 | | 0.0446 | | Cinderblocks | |
| 437 | | -1 | | 1032.793 | | | 1 | | 22.88637 | | 295.0365 | | 52.80055 | | 3-6 Block8 | | 71.29125 | | 270.8589 | 3 6 in pallets cinderblocks | | cinderblocks | 3CB | | CB | | low | 7 | | 1 | | | 7.940023 | | 1.032793 | | Cinderblocks | |
| 438 | | -1 | | 2790.237 | | | 1 | | 21.45866 | | 913.6118 | | 37.23154 | | 3-6 Block9 | | 36.57225 | | 53.73859 | 3 6 in palet cinderblocks | | cinderblocks | 3CB | | CB | | low | 7 | | 1 | | | 8.933882 | | 2.790237 | | Cinderblocks | |
| 439 | | -1 | | 1376.752 | | | 1 | | 22.7155 | | 398.5192 | | 17.60674 | | 3-8 Block10 | | 56.35394 | | 198.3655 | 3 8 in pallets cinderblocks | | cinderblocks | 3CB | | CB | | low | 7 | | 1 | | | 8.227483 | | 1.376752 | | Cinderblocks | |
| 440 | | -1 | | 2155.51 | | | 1 | | 22.68734 | | 404.4364 | | 18.8001 | | 3-8 Block10 | | 56.35394 | | 198.3655 | 3 8 in pallets cinderblocks | | cinderblocks | 3CB | | CB | | low | 7 | | 1 | | | 8.675783 | | 2.15551 | | Cinderblocks | |
| 441 | | -1 | | 5851.445 | | | 1 | | 22.75176 | | 443.3395 | | 23.99732 | | 3-8 Block11 | | 42.96258 | | 102.7157 | 3 8 in pallets cinderblocks | | cinderblocks | 3CB | | CB | | low | 7 | | 1 | | | 9.674444 | | 5.851445 | | Cinderblocks | |
| 443 | | -1 | | 1908.639 | | | 1 | | 22.25649 | | 522.883 | | 64.07548 | | 3-8 Block4 | | 42.30081 | | 120.303 | 3 8 in pallets cinderblocks | | cinderblocks | 3CB | | CB | | low | 7 | | 1 | | | 8.554146 | | 1.908639 | | Cinderblocks | |
| 444 | | -1 | | 836.4511 | | | 1 | | 21.23988 | | 339.8528 | | 4.34901 | | 3-mixed Block24 | | 19.27627 | | 19.03456 | 3 pallets mixed size ciderblocks | | cinderblocks | 3CB | | CB | | low | 7 | | 1 | | | 7.729168 | | 0.836451 | | Cinderblocks | |
| 445 | | -1 | | 7601.332 | | | 1 | | 21.19791 | | 340.5204 | | 33.89589 | | 3-mixed Block24 | | 19.27627 | | 19.03456 | 3 pallets mixed size ciderblocks | | cinderblocks | 3CB | | CB | | low | 7 | | 1 | | | 9.936079 | | 7.601332 | | Cinderblocks | |
| 446 | | -1 | | 4263.663 | | | 1 | | 22.29338 | | 342.1496 | | 17.21378 | | 3-mixed Block27 | | 103.5768 | | 672.5635 | 3 pallets mixed size cinderblocks | | cinderblocks | 3CB | | CB | | low | 7 | | 1 | | | 9.357884 | | 4.263663 | | Cinderblocks | |
| 450 | | -1 | | 79.92898 | | | 1 | | 22.71892 | | 336.6892 | | 3.776561 | | 3-mixed Block30 | | 73.51531 | | 362.758 | 3 pallets mixed size cinderblocks | | cinderblocks | 3CB | | CB | | low | 7 | | 1 | | | 5.381138 | | 0.079929 | | Cinderblocks | |
| 451 | | -1 | | 2282.33 | | | 1 | | 21.70769 | | 838.0264 | | 25.8106 | | 3-mixed Block39 | | 29.46751 | | 47.73784 | 3 pallets mixed size ciderblocks | | cinderblocks | 3CB | | CB | | low | 7 | | 1 | | | 8.732952 | | 2.28233 | | Cinderblocks | |
| 544 | | -1 | | 81.22128 | | | 1 | | 21.20848 | | 277.9222 | | 87.01546 | | 3-mixed Block24 | | 19.27627 | | 19.03456 | 3 pallets mixed size ciderblocks | | cinderblocks | 3CB | | CB | | low | 7 | | 1 | | | 5.397177 | | 0.081221 | | Cinderblocks | |
| 577 | | -1 | | 1987.455 | | | 1 | | 22.7777 | | 664.7755 | | 90.26353 | | 3-6 Block5 | | 13.06464 | | 11.46195 | 3 6 in palet cinderblocks | | cinderblocks | 3CB | | CB | | low | 7 | | 1 | | | 8.59461 | | 1.987455 | | Cinderblocks | |
| 578 | | -1 | | 2780.259 | | | 1 | | 22.68965 | | 688.498 | | 78.56564 | | 3-8 Block6 | | 43.51419 | | 96.51487 | 3 8 in pallets cinderblocks | | cinderblocks | 3CB | | CB | | low | 7 | | 1 | | | 8.930299 | | 2.780259 | | Cinderblocks | |
| 618 | | -1 | | 802.2704 | | | 1 | | 22.08456 | | 541.6642 | | 136.7003 | | 3-mixed Block17 | | 44.22191 | | 128.6117 | 3 pallets mixed size cinderblocks | | cinderblocks | 3CB | | CB | | low | 7 | | 1 | | | 7.687446 | | 0.80227 | | Cinderblocks | |
| 619 | | -1 | | 1576.593 | | | 1 | | 22.14802 | | 577.3668 | | 90.88091 | | 3-mixed Block17 | | 44.22191 | | 128.6117 | 3 pallets mixed size cinderblocks | | cinderblocks | 3CB | | CB | | low | 7 | | 1 | | | 8.363021 | | 1.576593 | | Cinderblocks | |
| 620 | | -1 | | 608.9512 | | | 1 | | 22.26402 | | 674.8246 | | 19.70113 | | 3-8 Block5 | | 55.30717 | | 140.0044 | 3 8 in palet cinderblocks | | cinderblocks | 3CB | | CB | | low | 7 | | 1 | | | 7.411738 | | 0.608951 | | Cinderblocks | |
| 676 | | -1 | | 497.0394 | | | 1 | | 21.54278 | | 724.606 | | 77.46891 | | 3-4 Block11 | | 18.6368 | | 19.13105 | 3 4 in palet cinderblocks | | cinderblocks | 3CB | | CB | | low | 7 | | 1 | | | 7.208669 | | 0.497039 | | Cinderblocks | |
| 678 | | -1 | | 293.2277 | | | 1 | | 22.49321 | | 560.9995 | | 57.30195 | | 3-6 Block6 | | 83.61275 | | 298.1626 | 3 6 in pallets cinderblocks | | cinderblocks | 3CB | | CB | | low | 7 | | 1 | | | 6.68095 | | 0.293228 | | Cinderblocks | |
| 679 | | -1 | | 730.9902 | | | 1 | | 22.48692 | | 560.4114 | | 69.83906 | | 3-6 Block6 | | 83.61275 | | 298.1626 | 3 6 in pallets cinderblocks | | cinderblocks | 3CB | | CB | | low | 7 | | 1 | | | 7.5944 | | 0.73099 | | Cinderblocks | |
| 680 | | -1 | | 1355.94 | | | 1 | | 21.47081 | | 1020.573 | | 63.86991 | | 3-mixed Block21 | | 37.05382 | | 98.80981 | 3 pallets mixed size ciderblocks | | cinderblocks | 3CB | | CB | | low | 7 | | 1 | | | 8.21225 | | 1.35594 | | Cinderblocks | |
| 718 | | -1 | | 5496.715 | | | 1 | | 21.50374 | | 1032.702 | | 12.95695 | | 3-mixed Block21 | | 37.05382 | | 98.80981 | 3 pallets mixed size ciderblocks | | cinderblocks | 3CB | | CB | | low | 7 | | 1 | | | 9.611906 | | 5.496715 | | Cinderblocks | |
| 720 | | -1 | | 458.4673 | | | 1 | | 21.67078 | | 964.4229 | | 43.6247 | | 3-mixed Block20 | | 41.90559 | | 91.41175 | 3 pallets mixed size ciderblocks | | cinderblocks | 3CB | | CB | | low | 7 | | 1 | | | 7.127889 | | 0.458467 | | Cinderblocks | |
| 721 | | -1 | | 32.0308 | | | 1 | | 22.3446 | | 343.7431 | | 36.06282 | | 3-mixed Block27 | | 103.5768 | | 672.5635 | 3 pallets mixed size cinderblocks | | cinderblocks | 3CB | | CB | | low | 7 | | 1 | | | 4.466698 | | 0.032031 | | Cinderblocks | |
| 766 | | -1 | | 67.14719 | | | 3 | | 22.92052 | | 289.5773 | | 39.93844 | | 3-8 Block13 | | 47.72747 | | 115.4474 | 3 8 in pallets cinderblocks | | cinderblocks | 3CB | | CB | | low | 7 | | 1 | | | 5.206887 | | 0.067147 | | Cinderblocks | |
| 798 | | -1 | | 1635.62 | | | 82 | | 20.67154 | | 112.0886 | | 44.12731 | | 3-6 Block2 | | 88.98341 | | 310.1671 | 3 6 in palet cinderblocks | | cinderblocks | 3CB | | CB | | low | 7 | | 1 | | | 8.399777 | | 1.63562 | | Cinderblocks | |
| 800 | | -1 | | 3789.344 | | | 189 | | 22.75218 | | 482.7797 | | 76.57922 | | 3-8 Block12 | | 66.92699 | | 188.0087 | 3 8 in pallets cinderblocks | | cinderblocks | 3CB | | CB | | low | 7 | | 1 | | | 9.239948 | | 3.789344 | | Cinderblocks | |
| 100 | | 100 | | 124234.4 | | | 4676.694 | | 22.36063 | | 777.4906 | | 17.8207 | | 400 | | 130.5063 | | 966.1556 | 400 rr tie | | rr ties | 400T RR | | RR | | high | 7 | | 1 | | | 12.72993 | | 124.2344 | | RR Ties | |
| 101 | | 101 | | 48060.26 | | | 2403.013 | | 22.26641 | | 791.4871 | | 20.51381 | | 400 | | 130.5063 | | 966.1556 | 400 rr tie | | rr ties | 400T RR | | RR | | high | 7 | | 1 | | | 11.78021 | | 48.06026 | | RR Ties | |
| 154 | | 154 | | 1685.752 | | | 3 | | 20.18907 | | 535.7435 | | 61.61308 | | Patch 15 | | 91.68124 | | 510.356 | low profile | | low profile | 4LP | | LP | | low | 7 | | 1 | | | 8.429967 | | 1.685752 | | Low Profile | |
| 187 | | 187 | | 2764.814 | | | 2 | | 19.21339 | | 250.4736 | | 64.57396 | | Patch 47 | | 88.31676 | | 367.8783 | low profile | | low profile | 4LP | | LP | | low | 7 | | 1 | | | 8.924729 | | 2.764814 | | Low Profile | |
| 529 | | -1 | | 238.879 | | | 1 | | 19.25494 | | 244.6953 | | 83.87591 | | Patch 47 | | 88.31676 | | 367.8783 | low profile | | low profile | 4LP | | LP | | low | 7 | | 1 | | | 6.475957 | | 0.238879 | | Low Profile | |
| 531 | | -1 | | 549.6793 | | | 1 | | 19.29656 | | 299.0916 | | 15.76155 | | Patch 47 | | 88.31676 | | 367.8783 | low profile | | low profile | 4LP | | LP | | low | 7 | | 1 | | | 7.309335 | | 0.549679 | | Low Profile | |
| 655 | | -1 | | 1703.964 | | | 1 | | 19.42478 | | 502.9995 | | 131.981 | | Patch 12 | | 79.91582 | | 363.6088 | low profile | | low profile | 4LP | | LP | | low | 7 | | 1 | | | 8.440713 | | 1.703964 | | Low Profile | |
| 736 | | -1 | | 1569.413 | | | 1 | | 19.5982 | | 301.7161 | | 81.24162 | | Patch 47 | | 88.31676 | | 367.8783 | low profile | | low profile | 4LP | | LP | | low | 7 | | 1 | | | 8.358457 | | 1.569413 | | Low Profile | |
| 740 | | -1 | | 20.19801 | | | 1 | | 20.22792 | | 583.2229 | | 39.76075 | | Patch 15 | | 91.68124 | | 510.356 | low profile | | low profile | 4LP | | LP | | low | 7 | | 1 | | | 4.005584 | | 0.020198 | | Low Profile | |
| 755 | | -1 | | 35.98706 | | | 2 | | 20.30906 | | 589.4804 | | 57.63015 | | Patch 15 | | 91.68124 | | 510.356 | low profile | | low profile | 4LP | | LP | | low | 7 | | 1 | | | 4.583159 | | 0.035987 | | Low Profile | |
| 772 | | -1 | | 83.95761 | | | 4 | | 20.31668 | | 520.242 | | 82.05868 | | Patch 15 | | 91.68124 | | 510.356 | low profile | | low profile | 4LP | | LP | | low | 7 | | 1 | | | 5.430312 | | 0.083958 | | Low Profile | |
| 145 | | 145 | | 2622.611 | | | 2 | | 20.48765 | | 374.8077 | | 12.53726 | | Patch 10 | | 93.80683 | | 525.4204 | mixed; pyramids, lpm | | pyramids, low profile | 4MX | | MX | | mid | 7 | | 2 | | | 8.871926 | | 2.622611 | | Pyramids | |
| 146 | | 146 | | 7152.92 | | | 2 | | 20.4596 | | 378.2051 | | 3.852963 | | Patch 10 | | 93.80683 | | 525.4204 | mixed; pyramids, lpm | | pyramids, low profile | 4MX | | MX | | mid | 7 | | 2 | | | 9.875276 | | 7.15292 | | Pyramids | |
| 155 | | 155 | | 3281.256 | | | 12.7632 | | 19.57223 | | 195.7254 | | 18.56043 | | Patch 16 | | 124.8235 | | 620.7212 | mixed; pyramids, lpm | | pyramids, low profile | 4MX | | MX | | mid | 7 | | 2 | | | 9.095981 | | 3.281256 | | Pyramids | |
| 156 | | 156 | | 24912.04 | | | 21 | | 19.28091 | | 219.9643 | | 8.49327 | | Patch 16 | | 124.8235 | | 620.7212 | mixed; pyramids, lpm | | pyramids, low profile | 4MX | | MX | | mid | 7 | | 2 | | | 11.12311 | | 24.91204 | | Pyramids | |
| 157 | | 157 | | 2709.914 | | | 3 | | 19.48361 | | 172.7987 | | 41.27822 | | Patch 16 | | 124.8235 | | 620.7212 | mixed; pyramids, lpm | | pyramids, low profile | 4MX | | MX | | mid | 7 | | 2 | | | 8.904672 | | 2.709914 | | Pyramids | |
| 171 | | 171 | | 33956.77 | | | 11 | | 20.50417 | | 942.1482 | | 21.48437 | | Patch 25 | | 105.5926 | | 744.7434 | mixed; pyramids, lpm | | pyramids, low profile | 4MX | | MX | | mid | 7 | | 2 | | | 11.43284 | | 33.95677 | | Pyramids | |
| 172 | | 172 | | 1083.834 | | | 5.931372 | | 20.78847 | | 910.0886 | | 18.09873 | | Patch 25 | | 105.5926 | | 744.7434 | mixed; pyramids, lpm | | pyramids, low profile | 4MX | | MX | | mid | 7 | | 2 | | | 7.98826 | | 1.083834 | | Pyramids | |
| 177 | | 177 | | 16938.54 | | | 8 | | 19.69993 | | 371.1442 | | 11.33215 | | Patch 32 | | 109.7367 | | 744.9834 | mixed; pyramids, lpm | | pyramids, low profile | 4MX | | MX | | mid | 7 | | 2 | | | 10.73735 | | 16.93854 | | Pyramids | |
| 178 | | 178 | | 1154.742 | | | 2 | | 19.60149 | | 360.8198 | | 23.08212 | | Patch 32 | | 109.7367 | | 744.9834 | mixed; pyramids, lpm | | pyramids, low profile | 4MX | | MX | | mid | 7 | | 2 | | | 8.051632 | | 1.154742 | | Pyramids | |
| 179 | | 179 | | 6898.834 | | | 2 | | 19.40795 | | 397.0573 | | 16.09042 | | Patch 32 | | 109.7367 | | 744.9834 | mixed; pyramids, lpm | | pyramids, low profile | 4MX | | MX | | mid | 7 | | 2 | | | 9.839108 | | 6.898834 | | Pyramids | |
| 211 | | 211 | | 66625.47 | | | 617.7443 | | 19.12519 | | 367.1964 | | 17.31809 | | Patch 7 | | 110.3538 | | 720.7243 | mixed; pyramids, lpm | | pyramids, low profile | 4MX | | MX | | mid | 7 | | 2 | | | 12.10684 | | 66.62547 | | Pyramids | |
| 340 | | 340 | | 14668.79 | | | 627 | | 19.48249 | | 70.15417 | | 144.0074 | | Patch 16 | | 124.8235 | | 620.7212 | mixed; pyramids, lpm | | pyramids, low profile | 4MX | | MX | | mid | 7 | | 2 | | | 10.59348 | | 14.66879 | | Pyramids | |
| 508 | | -1 | | 5827.75 | | | 1 | | 20.41423 | | 379.0783 | | 17.71106 | | Patch 10 | | 93.80683 | | 525.4204 | mixed; pyramids, lpm | | pyramids, low profile | 4MX | | MX | | mid | 7 | | 2 | | | 9.670386 | | 5.82775 | | Pyramids | |
| 517 | | -1 | | 7089.066 | | | 1 | | 20.39695 | | 944.4401 | | 38.5784 | | Patch 25 | | 105.5926 | | 744.7434 | mixed; pyramids, lpm | | pyramids, low profile | 4MX | | MX | | mid | 7 | | 2 | | | 9.866309 | | 7.089066 | | Pyramids | |
| 640 | | -1 | | 483.4837 | | | 1 | | 19.24926 | | 244.0121 | | 30.17851 | | Patch 16 | | 124.8235 | | 620.7212 | mixed; pyramids, lpm | | pyramids, low profile | 4MX | | MX | | mid | 7 | | 2 | | | 7.181018 | | 0.483484 | | Pyramids | |
| 644 | | -1 | | 252.5713 | | | 1 | | 20.27017 | | 864.7256 | | 75.06973 | | Patch 25 | | 105.5926 | | 744.7434 | mixed; pyramids, lpm | | pyramids, low profile | 4MX | | MX | | mid | 7 | | 2 | | | 6.531694 | | 0.252571 | | Pyramids | |
| 650 | | -1 | | 10146.18 | | | 1 | | 19.1596 | | 441.6953 | | 93.59429 | | Patch 7 | | 110.3538 | | 720.7243 | mixed; pyramids, lpm | | pyramids, low profile | 4MX | | MX | | mid | 7 | | 2 | | | 10.22485 | | 10.14618 | | Pyramids | |
| 742 | | -1 | | 21.01496 | | | 1 | | 20.332 | | 841.0158 | | 104.5221 | | Patch 25 | | 105.5926 | | 744.7434 | mixed; pyramids, lpm | | pyramids, low profile | 4MX | | MX | | mid | 7 | | 2 | | | 4.045235 | | 0.021015 | | Pyramids | |
| 752 | | -1 | | 32.07656 | | | 2 | | 20.35563 | | 842.6477 | | 88.82502 | | Patch 25 | | 105.5926 | | 744.7434 | mixed; pyramids, lpm | | pyramids, low profile | 4MX | | MX | | mid | 7 | | 2 | | | 4.468126 | | 0.032077 | | Pyramids | |
| 158 | | 158 | | 50255.74 | | | 14 | | 19.61365 | | 381.1091 | | 1.612217 | | Patch 17 | | 78.79132 | | 371.8953 | pyramids | | pyramids | 4PY | | PY | | mid | 7 | | 1 | | | 11.82488 | | 50.25574 | | Pyramids | |
| 170 | | 170 | | 28073.41 | | | 12 | | 20.15241 | | 747.6596 | | 14.81587 | | Patch 24 | | 75.66856 | | 302.1624 | pyramids | | pyramids | 4PY | | PY | | mid | 7 | | 1 | | | 11.24258 | | 28.07341 | | Pyramids | |
| 175 | | 175 | | 24262.22 | | | 163.3002 | | 19.58459 | | 205.7323 | | 19.04248 | | Patch 3 | | 98.82283 | | 475.9369 | pyramids | | pyramids | 4PY | | PY | | mid | 7 | | 1 | | | 11.09668 | | 24.26222 | | Pyramids | |
| 180 | | 180 | | 62888.25 | | | 4 | | 20.0597 | | 572.6132 | | 7.713519 | | Patch 33 | | 57.59992 | | 164.5146 | pyramids | | pyramids | 4PY | | PY | | mid | 7 | | 1 | | | 12.04911 | | 62.88825 | | Pyramids | |
| 181 | | 181 | | 8706.659 | | | 4 | | 19.61173 | | 196.665 | | 21.01863 | | Patch 36 | | 95.28025 | | 549.0141 | pyramids | | pyramids | 4PY | | PY | | mid | 7 | | 1 | | | 10.07184 | | 8.706659 | | Pyramids | |
| 182 | | 182 | | 12465.51 | | | 2 | | 19.39764 | | 234.0094 | | 34.16964 | | Patch 36 | | 95.28025 | | 549.0141 | pyramids | | pyramids | 4PY | | PY | | mid | 7 | | 1 | | | 10.43072 | | 12.46551 | | Pyramids | |
| 183 | | 183 | | 1088.507 | | | 2 | | 19.78415 | | 197.3189 | | 11.09901 | | Patch 36 | | 95.28025 | | 549.0141 | pyramids | | pyramids | 4PY | | PY | | mid | 7 | | 1 | | | 7.992562 | | 1.088507 | | Pyramids | |
| 185 | | 185 | | 6453.721 | | | 3 | | 20.17655 | | 465.1187 | | 44.07774 | | Patch 45 | | 76.10071 | | 360.4427 | pyramids | | pyramids | 4PY | | PY | | mid | 7 | | 1 | | | 9.772412 | | 6.453721 | | Pyramids | |
| 199 | | 199 | | 2920.247 | | | 2 | | 20.94468 | | 264.8232 | | 35.76902 | | Patch 53 | | 100.4747 | | 648.5333 | pyramids | | pyramids | 4PY | | PY | | mid | 7 | | 1 | | | 8.979424 | | 2.920247 | | Pyramids | |
| 200 | | 200 | | 623.5867 | | | 2 | | 20.50788 | | 459.8128 | | 25.13448 | | Patch 54 | | 127.0256 | | 888.9146 | pyramids | | pyramids | 4PY | | PY | | mid | 7 | | 1 | | | 7.435488 | | 0.623587 | | Pyramids | |
| 320 | | 320 | | 63212.81 | | | 3013 | | 20.43272 | | 518.2298 | | 111.9281 | | Patch 45 | | 76.10071 | | 360.4427 | pyramids | | pyramids | 4PY | | PY | | mid | 7 | | 1 | | | 12.05426 | | 63.21281 | | Pyramids | |
| 323 | | 323 | | 6440.496 | | | 2 | | 20.16366 | | 813.5946 | | 103.8002 | | Patch 24 | | 75.66856 | | 302.1624 | pyramids | | pyramids | 4PY | | PY | | mid | 7 | | 1 | | | 9.770361 | | 6.440496 | | Pyramids | |
| 326 | | 326 | | 1517.68 | | | 2 | | 19.59907 | | 266.0483 | | 104.9929 | | Patch 3 | | 98.82283 | | 475.9369 | pyramids | | pyramids | 4PY | | PY | | mid | 7 | | 1 | | | 8.324938 | | 1.51768 | | Pyramids | |
| 335 | | 335 | | 21404.79 | | | 4 | | 19.93896 | | 581.2188 | | 19.10227 | | Patch 33 | | 57.59992 | | 164.5146 | pyramids | | pyramids | 4PY | | PY | | mid | 7 | | 1 | | | 10.97137 | | 21.40479 | | Pyramids | |
| 390 | | 390 | | 2193.035 | | | 3 | | 20.73973 | | 214.1451 | | 40.80238 | | Patch 53 | | 100.4747 | | 648.5333 | pyramids | | pyramids | 4PY | | PY | | mid | 7 | | 1 | | | 8.693042 | | 2.193035 | | Pyramids | |
| 518 | | -1 | | 200.4951 | | | 1 | | 20.11132 | | 561.2809 | | 5.930206 | | Patch 33 | | 57.59992 | | 164.5146 | pyramids | | pyramids | 4PY | | PY | | mid | 7 | | 1 | | | 6.30079 | | 0.200495 | | Pyramids | |
| 519 | | -1 | | 2311.371 | | | 1 | | 19.95255 | | 585.5213 | | 20.04998 | | Patch 33 | | 57.59992 | | 164.5146 | pyramids | | pyramids | 4PY | | PY | | mid | 7 | | 1 | | | 8.745596 | | 2.311371 | | Pyramids | |
| 524 | | -1 | | 450.1519 | | | 1 | | 19.42337 | | 201.932 | | 27.58057 | | Patch 36 | | 95.28025 | | 549.0141 | pyramids | | pyramids | 4PY | | PY | | mid | 7 | | 1 | | | 7.109585 | | 0.450152 | | Pyramids | |
| 527 | | -1 | | 2708.621 | | | 1 | | 20.41792 | | 480.8313 | | 38.14282 | | Patch 45 | | 76.10071 | | 360.4427 | pyramids | | pyramids | 4PY | | PY | | mid | 7 | | 1 | | | 8.904195 | | 2.708621 | | Pyramids | |
| 528 | | -1 | | 130.758 | | | 6.537898 | | 20.54719 | | 435.2255 | | 28.01088 | | Patch 45 | | 76.10071 | | 360.4427 | pyramids | | pyramids | 4PY | | PY | | mid | 7 | | 1 | | | 5.873348 | | 0.130758 | | Pyramids | |
| 545 | | -1 | | 2466.754 | | | 1 | | 20.88455 | | 255.0675 | | 23.62857 | | Patch 53 | | 100.4747 | | 648.5333 | pyramids | | pyramids | 4PY | | PY | | mid | 7 | | 1 | | | 8.810659 | | 2.466754 | | Pyramids | |
| 546 | | -1 | | 3561.453 | | | 1 | | 20.99331 | | 257.7764 | | 9.499559 | | Patch 53 | | 100.4747 | | 648.5333 | pyramids | | pyramids | 4PY | | PY | | mid | 7 | | 1 | | | 9.177924 | | 3.561453 | | Pyramids | |
| 547 | | -1 | | 152.3372 | | | 7.61686 | | 20.82132 | | 440.8714 | | 32.93245 | | Patch 54 | | 127.0256 | | 888.9146 | pyramids | | pyramids | 4PY | | PY | | mid | 7 | | 1 | | | 6.026096 | | 0.152337 | | Pyramids | |
| 638 | | -1 | | 3043.105 | | | 1 | | 19.13849 | | 184.6168 | | 82.78497 | | Patch 36 | | 95.28025 | | 549.0141 | pyramids | | pyramids | 4PY | | PY | | mid | 7 | | 1 | | | 9.020634 | | 3.043105 | | Pyramids | |
| 642 | | -1 | | 3829.069 | | | 1 | | 20.2866 | | 491.7819 | | 53.42975 | | Patch 45 | | 76.10071 | | 360.4427 | pyramids | | pyramids | 4PY | | PY | | mid | 7 | | 1 | | | 9.250377 | | 3.829069 | | Pyramids | |
| 645 | | -1 | | 81.80646 | | | 1 | | 20.08401 | | 785.0593 | | 104.6697 | | Patch 24 | | 75.66856 | | 302.1624 | pyramids | | pyramids | 4PY | | PY | | mid | 7 | | 1 | | | 5.404356 | | 0.081806 | | Pyramids | |
| 647 | | -1 | | 509.0244 | | | 1 | | 19.7084 | | 115.0112 | | 190.9389 | | Patch 3 | | 98.82283 | | 475.9369 | pyramids | | pyramids | 4PY | | PY | | mid | 7 | | 1 | | | 7.232496 | | 0.509024 | | Pyramids | |
| 741 | | -1 | | 20.83341 | | | 1 | | 19.59352 | | 251.4292 | | 80.13525 | | Patch 3 | | 98.82283 | | 475.9369 | pyramids | | pyramids | 4PY | | PY | | mid | 7 | | 1 | | | 4.036558 | | 0.020833 | | Pyramids | |
| 748 | | -1 | | 29.08896 | | | 1 | | 19.19352 | | 228.2919 | | 41.8137 | | Patch 36 | | 95.28025 | | 549.0141 | pyramids | | pyramids | 4PY | | PY | | mid | 7 | | 1 | | | 4.370359 | | 0.029089 | | Pyramids | |
| 750 | | -1 | | 31.16959 | | | 2 | | 19.85494 | | 94.31022 | | 198.8068 | | Patch 3 | | 98.82283 | | 475.9369 | pyramids | | pyramids | 4PY | | PY | | mid | 7 | | 1 | | | 4.439443 | | 0.03117 | | Pyramids | |
| 759 | | -1 | | 41.65367 | | | 2 | | 19.19605 | | 231.5434 | | 52.69394 | | Patch 3 | | 98.82283 | | 475.9369 | pyramids | | pyramids | 4PY | | PY | | mid | 7 | | 1 | | | 4.729389 | | 0.041654 | | Pyramids | |
| 764 | | -1 | | 50.31047 | | | 3 | | 19.1089 | | 168.9831 | | 57.20329 | | Patch 3 | | 98.82283 | | 475.9369 | pyramids | | pyramids | 4PY | | PY | | mid | 7 | | 1 | | | 4.918213 | | 0.05031 | | Pyramids | |
| 806 | | -1 | | 38132.73 | | | 1907 | | 19.93522 | | 27.61259 | | 235.4225 | | Patch 3 | | 98.82283 | | 475.9369 | pyramids | | pyramids | 4PY | | PY | | mid | 7 | | 1 | | | 11.54883 | | 38.13273 | | Pyramids | |
| 1 | | 1 | | 2145.579 | | | 2.099574 | | 22.18251 | | 902.1543 | | 22.49382 | | 50.1 | | 105.3767 | | 615.0631 | 50 rr tie | | rr ties | 50T RR | | RR | | low-mid | 7 | | 1 | | | 8.671165 | | 2.145579 | | RR Ties | |
| 410 | | -1 | | 1153.462 | | | 1 | | 22.16318 | | 926.0255 | | 2.519855 | | 50.1 | | 105.3767 | | 615.0631 | 50 rr tie | | rr ties | 50T RR | | RR | | low-mid | 7 | | 1 | | | 8.050523 | | 1.153462 | | RR Ties | |
| 357 | | 357 | | 26294.49 | | | 584 | | 21.68088 | | 860.2147 | | 53.43298 | | 67.1 | | 83.66273 | | 479.9671 | 67 rr tie | | rr ties | 67T RR | | RR | | low-mid | 7 | | 1 | | | 11.17711 | | 26.29449 | | RR Ties | |
| 362 | | 362 | | 21708.69 | | | 7 | | 21.89573 | | 846.6121 | | 13.93061 | | 67.1 | | 83.66273 | | 479.9671 | 67 rr tie | | rr ties | 67T RR | | RR | | low-mid | 7 | | 1 | | | 10.98547 | | 21.70869 | | RR Ties | |
| 363 | | 363 | | 4934.909 | | | 2 | | 21.85619 | | 839.8601 | | 49.4485 | | 67.1 | | 83.66273 | | 479.9671 | 67 rr tie | | rr ties | 67T RR | | RR | | low-mid | 7 | | 1 | | | 9.50409 | | 4.934909 | | RR Ties | |
| 364 | | 364 | | 56702.43 | | | 11 | | 22.32309 | | 763.4017 | | 27.08829 | | 67.2 | | 94.36919 | | 501.0762 | 67 rr tie | | rr ties | 67T RR | | RR | | low-mid | 7 | | 1 | | | 11.94557 | | 56.70243 | | RR Ties | |
| 715 | | -1 | | 11828.92 | | | 1 | | 22.76871 | | 682.4234 | | 99.17243 | | 67.2 | | 94.36919 | | 501.0762 | 67 rr tie | | rr ties | 67T RR | | RR | | low-mid | 7 | | 1 | | | 10.3783 | | 11.82892 | | RR Ties | |
| 716 | | -1 | | 1136.915 | | | 1 | | 21.70264 | | 893.8697 | | 53.40429 | | 67.1 | | 83.66273 | | 479.9671 | 67 rr tie | | rr ties | 67T RR | | RR | | low-mid | 7 | | 1 | | | 8.036074 | | 1.136915 | | RR Ties | |
| 60 | | 60 | | 31230.79 | | | 165.3408 | | 22.53987 | | 313.6131 | | 20.13081 | | Big Pile | | 235.1358 | | 3248.283 | big pile | | big pile | Big Pile | | Big Pile | | high | 7 | | 3 | | | 11.34916 | | 31.23079 | | Big Pile | |
| 61 | | 61 | | 160538.4 | | | 675.1207 | | 23.10888 | | 338.6254 | | 54.48294 | | Big Pile | | 235.1358 | | 3248.283 | big pile | | big pile | Big Pile | | Big Pile | | high | 7 | | 3 | | | 12.98629 | | 160.5384 | | Big Pile | |
| 341 | | 341 | | 73398.8 | | | 12 | | 23.07354 | | 323.4841 | | 46.3296 | | Big Pile | | 235.1358 | | 3248.283 | big pile | | big pile | Big Pile | | Big Pile | | high | 7 | | 3 | | | 12.20366 | | 73.3988 | | Big Pile | |
| 342 | | 342 | | 1694.074 | | | 2 | | 22.9943 | | 336.5961 | | 43.2313 | | Big Pile | | 235.1358 | | 3248.283 | big pile | | big pile | Big Pile | | Big Pile | | high | 7 | | 3 | | | 8.434892 | | 1.694074 | | Big Pile | |
| 343 | | 343 | | 382939.1 | | | 14443 | | 23.15079 | | 269.4531 | | 32.4138 | | Big Pile | | 235.1358 | | 3248.283 | big pile | | big pile | Big Pile | | Big Pile | | high | 7 | | 3 | | | 13.85563 | | 382.9391 | | Big Pile | |
| 453 | | -1 | | 2775722 | | | 138786.1 | | 23.17039 | | 298.6266 | | 20.26202 | | Big Pile | | 235.1358 | | 3248.283 | big pile | | big pile | Big Pile | | Big Pile | | high | 7 | | 3 | | | 15.83642 | | 2775.722 | | Big Pile | |
| 621 | | -1 | | 1508.393 | | | 1 | | 23.0031 | | 256.3631 | | 58.78422 | | Big Pile | | 235.1358 | | 3248.283 | big pile | | big pile | Big Pile | | Big Pile | | high | 7 | | 3 | | | 8.3188 | | 1.508393 | | Big Pile | |
| 622 | | -1 | | 2034.779 | | | 1 | | 23.04831 | | 231.1025 | | 67.90989 | | Big Pile | | 235.1358 | | 3248.283 | big pile | | big pile | Big Pile | | Big Pile | | high | 7 | | 3 | | | 8.618143 | | 2.034779 | | Big Pile | |
| 623 | | -1 | | 6346.964 | | | 1 | | 23.21816 | | 245.3162 | | 56.99098 | | Big Pile | | 235.1358 | | 3248.283 | big pile | | big pile | Big Pile | | Big Pile | | high | 7 | | 3 | | | 9.755732 | | 6.346964 | | Big Pile | |
| 255 | | 255 | | 10202.91 | | | 7 | | 21.68537 | | 116.0858 | | 38.86152 | | Block UK3 | | 35.98888 | | 49.34789 | unknown size pallet of cinderblocks | | cinderblocks | CB | | CB | | low | 7 | | 1 | | | 10.23043 | | 10.20291 | | Cinderblocks | |
| 296 | | 296 | | 75247.42 | | | 3 | | 21.46667 | | 230.9104 | | 27.02817 | | Block UK4 | | 30.55053 | | 60.50174 | unknown size pallet of cinderblocks | | cinderblocks | CB | | CB | | low | 7 | | 1 | | | 12.22854 | | 75.24742 | | Cinderblocks | |
| 298 | | 298 | | 148466.6 | | | 4 | | 21.67134 | | 132.8578 | | 21.51944 | | Block UK1 | | 12.95602 | | 9.570499 | unknown size pallet of cinderblocks | | cinderblocks | CB | | CB | | low | 7 | | 1 | | | 12.90812 | | 148.4666 | | Cinderblocks | |
| 454 | | -1 | | 1664.859 | | | 1 | | 21.49033 | | 255.2103 | | 14.96662 | | Block UK4 | | 30.55053 | | 60.50174 | unknown size pallet of cinderblocks | | cinderblocks | CB | | CB | | low | 7 | | 1 | | | 8.417495 | | 1.664859 | | Cinderblocks | |
| 455 | | -1 | | 3754.062 | | | 1 | | 21.50226 | | 258.7323 | | 20.80145 | | Block UK4 | | 30.55053 | | 60.50174 | unknown size pallet of cinderblocks | | cinderblocks | CB | | CB | | low | 7 | | 1 | | | 9.230594 | | 3.754062 | | Cinderblocks | |
| 457 | | -1 | | 812.5641 | | | 1 | | 21.47116 | | 295.7819 | | 63.90464 | | Block UK4 | | 30.55053 | | 60.50174 | unknown size pallet of cinderblocks | | cinderblocks | CB | | CB | | low | 7 | | 1 | | | 7.700195 | | 0.812564 | | Cinderblocks | |
| 583 | | -1 | | 447.8105 | | | 1 | | 21.4926 | | 246.9078 | | 22.99426 | | Block UK4 | | 30.55053 | | 60.50174 | unknown size pallet of cinderblocks | | cinderblocks | CB | | CB | | low | 7 | | 1 | | | 7.10437 | | 0.447811 | | Cinderblocks | |
| 613 | | -1 | | 490.8505 | | | 1 | | 21.41939 | | 190.4295 | | 38.23932 | | Block UK1 | | 12.95602 | | 9.570499 | unknown size pallet of cinderblocks | | cinderblocks | CB | | CB | | low | 7 | | 1 | | | 7.19614 | | 0.49085 | | Cinderblocks | |
| 615 | | -1 | | 510.8842 | | | 1 | | 21.6222 | | 143.9664 | | 9.595277 | | Block UK1 | | 12.95602 | | 9.570499 | unknown size pallet of cinderblocks | | cinderblocks | CB | | CB | | low | 7 | | 1 | | | 7.236143 | | 0.510884 | | Cinderblocks | |
| 617 | | -1 | | 435.2558 | | | 1 | | 21.98475 | | 520.1711 | | 167.2053 | | Block UK6 | | 14.49552 | | 14.6002 | unknown size pallet of cinderblocks | | cinderblocks | CB | | CB | | low | 7 | | 1 | | | 7.075934 | | 0.435256 | | Cinderblocks | |
| 790 | | -1 | | 537.0403 | | | 27 | | 21.4919 | | 279.5234 | | 34.72252 | | Block UK4 | | 30.55053 | | 60.50174 | unknown size pallet of cinderblocks | | cinderblocks | CB | | CB | | low | 7 | | 1 | | | 7.286073 | | 0.53704 | | Cinderblocks | |
| 46 | | 46 | | 1807.292 | | | 2 | | 22.47253 | | 531.5972 | | 43.11302 | | 3mb ridge | | 641.3316 | | 5951.165 | 3 pallets mixed size cinderblocks in line | | cinderblocks | CB Ridge | | CB | | low | 7 | | 1 | | | 8.499585 | | 1.807292 | | Cinderblocks | |
| 49 | | 49 | | 2327.916 | | | 2 | | 22.38196 | | 532.6798 | | 34.27765 | | 3mb ridge | | 641.3316 | | 5951.165 | 3 pallets mixed size cinderblocks in line | | cinderblocks | CB Ridge | | CB | | low | 7 | | 1 | | | 8.752729 | | 2.327916 | | Cinderblocks | |
| 51 | | 51 | | 2871.749 | | | 3 | | 22.46781 | | 533.5125 | | 24.74033 | | 3mb ridge | | 641.3316 | | 5951.165 | 3 pallets mixed size cinderblocks in line | | cinderblocks | CB Ridge | | CB | | low | 7 | | 1 | | | 8.962676 | | 2.871749 | | Cinderblocks | |
| 442 | | -1 | | 951.4133 | | | 1 | | 22.53394 | | 531.7843 | | 92.80628 | | 3mb ridge | | 641.3316 | | 5951.165 | 3 pallets mixed size cinderblocks in line | | cinderblocks | CB Ridge | | CB | | low | 7 | | 1 | | | 7.857949 | | 0.951413 | | Cinderblocks | |
| 108 | | 108 | | 659.984 | | | 2.096664 | | 21.59699 | | 597.6025 | | 4.405383 | | LS15 | | 9.043823 | | 5.535933 | One Gulf LP | | limestone | Limestone | | Limestone | | low | 7 | | 1 | | | 7.492216 | | 0.659984 | | Concrete | |
| 399 | | 399 | | 465.9785 | | | 2 | | 21.68196 | | 556.5726 | | 24.96559 | | LS11 | | 8.903525 | | 5.889918 | One Gulf LP | | limestone | Limestone | | Limestone | | low | 7 | | 1 | | | 7.144139 | | 0.465978 | | Concrete | |
| 492 | | -1 | | 343.1634 | | | 1 | | 21.59835 | | 588.8728 | | 19.84576 | | LS15 | | 9.043823 | | 5.535933 | One Gulf LP | | limestone | Limestone | | Limestone | | low | 7 | | 1 | | | 6.838207 | | 0.343163 | | Concrete | |
| 493 | | -1 | | 899.3828 | | | 1 | | 21.33055 | | 451.457 | | 8.158099 | | LS2 | | 6.470833 | | 2.872527 | One Gulf LP | | limestone | Limestone | | Limestone | | low | 7 | | 1 | | | 7.801709 | | 0.899383 | | Concrete | |
| 491 | | -1 | | 198.3266 | | | 1 | | 21.27729 | | 580.6095 | | 22.58472 | | LPM2 | | 9.08543 | | 5.128091 | One Gulf LP | | low profile | Low Profile | | LP | | low | 7 | | 1 | | | 6.289915 | | 0.198327 | | Low Profile | |
| 707 | | -1 | | 30.85158 | | | 1 | | 21.29607 | | 602.5153 | | 47.62562 | | LPM2 | | 9.08543 | | 5.128091 | One Gulf LP | | low profile | Low Profile | | LP | | low | 7 | | 1 | | | 4.429188 | | 0.030852 | | Low Profile | |
| 708 | | -1 | | 64.38273 | | | 1 | | 21.28193 | | 598.8201 | | 70.48353 | | LPM2 | | 9.08543 | | 5.128091 | One Gulf LP | | low profile | Low Profile | | LP | | low | 7 | | 1 | | | 5.164845 | | 0.064383 | | Low Profile | |
| 235 | | 235 | | 67330.97 | | | 30 | | 22.47039 | | 735.5898 | | 39.01376 | | rr tie ridge | | 732.1532 | | 4589.931 | rr tie ridge | | rr tie ridge | RR Ridge | | RR | | mid | 7 | | 1 | | | 12.11738 | | 67.33097 | | RR Ties | |
| 238 | | 238 | | 3882.622 | | | 2 | | 22.37615 | | 760.2034 | | 17.13001 | | rr tie ridge | | 732.1532 | | 4589.931 | rr tie ridge | | rr tie ridge | RR Ridge | | RR | | mid | 7 | | 1 | | | 9.264266 | | 3.882622 | | RR Ties | |
| 239 | | 239 | | 39138.46 | | | 11 | | 22.59997 | | 701.9136 | | 71.6794 | | rr tie ridge | | 732.1532 | | 4589.931 | rr tie ridge | | rr tie ridge | RR Ridge | | RR | | mid | 7 | | 1 | | | 11.57486 | | 39.13846 | | RR Ties | |
| 241 | | 241 | | 7078.94 | | | 2 | | 22.49778 | | 725.9309 | | 47.0807 | | rr tie ridge | | 732.1532 | | 4589.931 | rr tie ridge | | rr tie ridge | RR Ridge | | RR | | mid | 7 | | 1 | | | 9.864879 | | 7.07894 | | RR Ties | |
| 242 | | 242 | | 26164.47 | | | 8 | | 22.42343 | | 749.9895 | | 3.235204 | | rr tie ridge | | 732.1532 | | 4589.931 | rr tie ridge | | rr tie ridge | RR Ridge | | RR | | mid | 7 | | 1 | | | 11.17216 | | 26.16447 | | RR Ties | |
| 576 | | -1 | | 1138.747 | | | 1 | | 22.30993 | | 746.9371 | | 63.67357 | | rr tie ridge | | 732.1532 | | 4589.931 | rr tie ridge | | rr tie ridge | RR Ridge | | RR | | mid | 7 | | 1 | | | 8.037684 | | 1.138747 | | RR Ties | |
| 579 | | -1 | | 4169.226 | | | 1 | | 22.56816 | | 707.8141 | | 64.67481 | | rr tie ridge | | 732.1532 | | 4589.931 | rr tie ridge | | rr tie ridge | RR Ridge | | RR | | mid | 7 | | 1 | | | 9.335486 | | 4.169226 | | RR Ties | |
| 149 | | 149 | | 39575.23 | | | 21.19444 | | 22.3118 | | 362.37 | | 9.681654 | | Patch 104 | | 24.27072 | | 30.63663 | mixed patch w/ 2 8 in palet cinderblocks   low profile chunks of concrete | | cinderblocks, concrete | CB, Concrete | | CB, Concrete | | low | 8 | | 2 | | | 11.58596 | | 39.57523 | | Other / Mixed | |
| 221 | | 221 | | 2248.724 | | | 2 | | 23.39028 | | 96.00932 | | 28.63701 | | Patch 87 | | 86.17518 | | 372.7075 | mixed patch w/ 2 8 in palet cinderblocks  low profile chunks of concrete  low profile chunks of concrete | | cinderblocks, concrete | CB, Concrete | | CB, Concrete | | low | 8 | | 2 | | | 8.718118 | | 2.248724 | | Other / Mixed | |
| 222 | | 222 | | 2826.772 | | | 2 | | 23.44825 | | 120.2889 | | 19.64382 | | Patch 87 | | 86.17518 | | 372.7075 | mixed patch w/ 2 8 in palet cinderblocks  low profile chunks of concrete  low profile chunks of concrete | | cinderblocks, concrete | CB, Concrete | | CB, Concrete | | low | 8 | | 2 | | | 8.946891 | | 2.826772 | | Other / Mixed | |
| 223 | | 223 | | 16908.3 | | | 7 | | 23.37787 | | 63.26686 | | 62.63484 | | Patch 87 | | 86.17518 | | 372.7075 | mixed patch w/ 2 8 in palet cinderblocks  low profile chunks of concrete  low profile chunks of concrete | | cinderblocks, concrete | CB, Concrete | | CB, Concrete | | low | 8 | | 2 | | | 10.73556 | | 16.9083 | | Other / Mixed | |
| 224 | | 224 | | 22907.83 | | | 4 | | 23.48682 | | 91.49779 | | 24.79274 | | Patch 87 | | 86.17518 | | 372.7075 | mixed patch w/ 2 8 in palet cinderblocks  low profile chunks of concrete  low profile chunks of concrete | | cinderblocks, concrete | CB, Concrete | | CB, Concrete | | low | 8 | | 2 | | | 11.03923 | | 22.90783 | | Other / Mixed | |
| 225 | | 225 | | 14224.13 | | | 5 | | 23.33302 | | 74.18584 | | 48.18194 | | Patch 87 | | 86.17518 | | 372.7075 | mixed patch w/ 2 8 in palet cinderblocks  low profile chunks of concrete  low profile chunks of concrete | | cinderblocks, concrete | CB, Concrete | | CB, Concrete | | low | 8 | | 2 | | | 10.5627 | | 14.22413 | | Other / Mixed | |
| 510 | | -1 | | 7601.777 | | | 1 | | 22.28628 | | 363.2837 | | 13.33667 | | Patch 104 | | 24.27072 | | 30.63663 | mixed patch w/ 2 8 in palet cinderblocks   low profile chunks of concrete | | cinderblocks, concrete | CB, Concrete | | CB, Concrete | | low | 8 | | 2 | | | 9.936137 | | 7.601777 | | Other / Mixed | |
| 572 | | -1 | | 1392.105 | | | 1 | | 23.31483 | | 117.069 | | 33.58831 | | Patch 87 | | 86.17518 | | 372.7075 | mixed patch w/ 2 8 in palet cinderblocks  low profile chunks of concrete  low profile chunks of concrete | | cinderblocks, concrete | CB, Concrete | | CB, Concrete | | low | 8 | | 2 | | | 8.238572 | | 1.392105 | | Other / Mixed | |
| 592 | | -1 | | 186558.9 | | | 1 | | 23.13588 | | 199.8598 | | 23.05716 | | Patch 92 | | 27.24881 | | 48.83047 | mixed patch w/ 2 8 in palet cinderblocks   low profile chunks of concrete   low profile chunks of concrete | | cinderblocks, concrete | CB, Concrete | | CB, Concrete | | low | 8 | | 2 | | | 13.1365 | | 186.5589 | | Other / Mixed | |
| 230 | | 230 | | 10785.39 | | | 2 | | 23.33348 | | 137.2322 | | 7.033603 | | Patch 94 | | 53.00958 | | 167.6276 | mixed patch w/ 1 6 in palet cinderblocks  2 8 in palet cinderblocks  5 round culverts  low profile chunks of concrete  low profile chunks of concrete | | cinderblocks, culverts, concrete | CB, Culvert, Concrete | | CB, Culvert, HD, Concrete | | mid-high | 8 | | 3 | | | 10.28595 | | 10.78539 | | Other / Mixed | |
| 278 | | 278 | | 89493.94 | | | 24 | | 23.19171 | | 92.83877 | | 47.30429 | | Patch 94 | | 53.00958 | | 167.6276 | mixed patch w/ 1 6 in palet cinderblocks  2 8 in palet cinderblocks  5 round culverts  low profile chunks of concrete  low profile chunks of concrete | | cinderblocks, culverts, concrete | CB, Culvert, Concrete | | CB, Culvert, HD, Concrete | | mid-high | 8 | | 3 | | | 12.40193 | | 89.49394 | | Other / Mixed | |
| 279 | | 279 | | 46214.85 | | | 12 | | 23.1805 | | 107.3441 | | 27.23607 | | Patch 94 | | 53.00958 | | 167.6276 | mixed patch w/ 1 6 in palet cinderblocks  2 8 in palet cinderblocks  5 round culverts  low profile chunks of concrete  low profile chunks of concrete | | cinderblocks, culverts, concrete | CB, Culvert, Concrete | | CB, Culvert, HD, Concrete | | mid-high | 8 | | 3 | | | 11.74106 | | 46.21485 | | Other / Mixed | |
| 281 | | 281 | | 2741.9 | | | 2 | | 23.21056 | | 84.38982 | | 50.62256 | | Patch 94 | | 53.00958 | | 167.6276 | mixed patch w/ 1 6 in palet cinderblocks  2 8 in palet cinderblocks  5 round culverts  low profile chunks of concrete  low profile chunks of concrete | | cinderblocks, culverts, concrete | CB, Culvert, Concrete | | CB, Culvert, HD, Concrete | | mid-high | 8 | | 3 | | | 8.916406 | | 2.7419 | | Other / Mixed | |
| 219 | | 219 | | 11029.23 | | | 6.717453 | | 22.62274 | | 288.1559 | | 9.287442 | | Patch 83 | | 79.78842 | | 374.0976 | mixed patch w/ 1 6 in palet cinderblocks  2 8 in palet cinderblocks  2 10x10 box culverts  3 pieces concrete highway divider | | cinderblocks, culverts, highway divider | CB, Culvert, HD | | CB, Culvert, HD, Concrete | | mid-high | 8 | | 3 | | | 10.3083 | | 11.02923 | | Other / Mixed | |
| 447 | | -1 | | 22.72513 | | | 1.136256 | | 22.56307 | | 330.8819 | | 67.56284 | | Patch 83 | | 79.78842 | | 374.0976 | mixed patch w/ 1 6 in palet cinderblocks  2 8 in palet cinderblocks  2 10x10 box culverts  3 pieces concrete highway divider | | cinderblocks, culverts, highway divider | CB, Culvert, HD | | CB, Culvert, HD, Concrete | | mid-high | 8 | | 3 | | | 4.123471 | | 0.022725 | | Other / Mixed | |
| 589 | | -1 | | 665.1988 | | | 1 | | 22.57585 | | 208.7831 | | 78.88066 | | Patch 83 | | 79.78842 | | 374.0976 | mixed patch w/ 1 6 in palet cinderblocks  2 8 in palet cinderblocks  2 10x10 box culverts  3 pieces concrete highway divider | | cinderblocks, culverts, highway divider | CB, Culvert, HD | | CB, Culvert, HD, Concrete | | mid-high | 8 | | 3 | | | 7.500086 | | 0.665199 | | Other / Mixed | |
| 722 | | -1 | | 8594.852 | | | 1 | | 22.56188 | | 242.4003 | | 38.96972 | | Patch 83 | | 79.78842 | | 374.0976 | mixed patch w/ 1 6 in palet cinderblocks  2 8 in palet cinderblocks  2 10x10 box culverts  3 pieces concrete highway divider | | cinderblocks, culverts, highway divider | CB, Culvert, HD | | CB, Culvert, HD, Concrete | | mid-high | 8 | | 3 | | | 10.05892 | | 8.594852 | | Other / Mixed | |
| 16 | | 16 | | 4556.109 | | | 2 | | 21.5448 | | 251.7839 | | 53.67452 | | Patch 78 | | 103.6715 | | 576.7736 | mixed patch w/ 1 6 in palet cinderblocks  2 8 in palet cinderblocks  2 10x10 box culverts  3 pieces concrete highway divider  low profile chunks of concrete | | cinderblocks, culverts, highway divider, concrete | CB, Culvert, HD, Concrete | | CB, Culvert, HD, Concrete | | mid-high | 8 | | 4 | | | 9.424224 | | 4.556109 | | Other / Mixed | |
| 18 | | 18 | | 1910.252 | | | 2 | | 22.11999 | | 227.2168 | | 58.77644 | | Patch 80 | | 89.20766 | | 478.0113 | mixed patch w/ 1 6 in palet cinderblocks  2 8 in palet cinderblocks  2 10x10 box culverts  3 pieces concrete highway divider  low profile chunks of concrete | | cinderblocks, culverts, highway divider, concrete | CB, Culvert, HD, Concrete | | CB, Culvert, HD, Concrete | | mid-high | 8 | | 4 | | | 8.55499 | | 1.910252 | | Other / Mixed | |
| 19 | | 19 | | 6143.731 | | | 2 | | 22.29005 | | 247.8613 | | 49.70548 | | Patch 82 | | 85.81274 | | 380.9502 | mixed patch w/ 1 6 in palet cinderblocks  2 8 in palet cinderblocks  2 10x10 box culverts  3 pieces concrete highway divider  3 pieces concrete highway divider  low profile chunks of concrete | | cinderblocks, culverts, highway divider, concrete | CB, Culvert, HD, Concrete | | CB, Culvert, HD, Concrete | | mid-high | 8 | | 4 | | | 9.723187 | | 6.143731 | | Other / Mixed | |
| 141 | | 141 | | 147364.2 | | | 6 | | 23.3183 | | 151.4181 | | 13.11097 | | Patch 99 | | 83.97865 | | 405.0499 | mixed patch w/ 1 6 in palet cinderblocks  2 8 in palet cinderblocks  3 pieces concrete highway divider  5 round culverts  low profile chunks of concrete | | cinderblocks, culverts, highway divider, concrete | CB, Culvert, HD, Concrete | | CB, Culvert, HD, Concrete | | mid-high | 8 | | 4 | | | 12.90066 | | 147.3642 | | Other / Mixed | |
| 147 | | 147 | | 67964.11 | | | 4 | | 22.98248 | | 137.0918 | | 5.792801 | | Patch 100 | | 71.85541 | | 338.5854 | mixed patch w/ 1 6 in palet cinderblocks, 2 8 in palet cinderblocks , 2 10x10 box culverts, 3 pieces concrete highway divider, low profile chunks of concrete | | cinderblocks, culverts, highway divider, concrete | CB, Culvert, HD, Concrete | | CB, Culvert, HD, Concrete | | mid-high | 8 | | 4 | | | 12.12674 | | 67.96411 | | Other / Mixed | |
| 148 | | 148 | | 9303.93 | | | 2 | | 22.95559 | | 128.9934 | | 25.54769 | | Patch 100 | | 71.85541 | | 338.5854 | mixed patch w/ 1 6 in palet cinderblocks, 2 8 in palet cinderblocks , 2 10x10 box culverts, 3 pieces concrete highway divider, low profile chunks of concrete | | cinderblocks, culverts, highway divider, concrete | CB, Culvert, HD, Concrete | | CB, Culvert, HD, Concrete | | mid-high | 8 | | 4 | | | 10.13819 | | 9.30393 | | Other / Mixed | |
| 212 | | 212 | | 13988.95 | | | 2 | | 20.98819 | | 619.5575 | | 14.27188 | | Patch 72 | | 67.7088 | | 245.8627 | mixed patch w/ 1 6 in palet cinderblocks  2 8 in palet cinderblocks  2 10x10 box culverts  3 pieces concrete highway divider  low profile chunks of concrete | | cinderblocks, culverts, highway divider, concrete | CB, Culvert, HD, Concrete | | CB, Culvert, HD, Concrete | | mid-high | 8 | | 4 | | | 10.54602 | | 13.98895 | | Other / Mixed | |
| 213 | | 213 | | 12253.12 | | | 2 | | 21.25929 | | 402.2882 | | 38.62027 | | Patch 74 | | 69.87934 | | 336.0509 | mixed patch w/ 2 8 in palet cinderblocks  2 10x10 box culverts  3 pieces concrete highway divider  low profile chunks of concrete  low profile chunks of concrete | | cinderblocks, culverts, highway divider, concrete | CB, Culvert, HD, Concrete | | CB, Culvert, HD, Concrete | | mid-high | 8 | | 4 | | | 10.41354 | | 12.25312 | | Other / Mixed | |
| 214 | | 214 | | 10851.01 | | | 2 | | 21.55938 | | 414.9924 | | 8.310256 | | Patch 74 | | 69.87934 | | 336.0509 | mixed patch w/ 2 8 in palet cinderblocks  2 10x10 box culverts  3 pieces concrete highway divider  low profile chunks of concrete  low profile chunks of concrete | | cinderblocks, culverts, highway divider, concrete | CB, Culvert, HD, Concrete | | CB, Culvert, HD, Concrete | | mid-high | 8 | | 4 | | | 10.29201 | | 10.85101 | | Other / Mixed | |
| 215 | | 215 | | 32710.96 | | | 7 | | 21.74982 | | 281.6994 | | 12.41074 | | Patch 77 | | 94.90258 | | 520.2692 | mixed patch w/ 1 6 in palet cinderblocks  2 8 in palet cinderblocks  2 10x10 box culverts  3 pieces concrete highway divider  low profile chunks of concrete | | cinderblocks, culverts, highway divider, concrete | CB, Culvert, HD, Concrete | | CB, Culvert, HD, Concrete | | mid-high | 8 | | 4 | | | 11.39547 | | 32.71096 | | Other / Mixed | |
| 216 | | 216 | | 956.9973 | | | 6.194211 | | 21.76919 | | 279.0206 | | 16.01782 | | Patch 78 | | 103.6715 | | 576.7736 | mixed patch w/ 1 6 in palet cinderblocks  2 8 in palet cinderblocks  2 10x10 box culverts  3 pieces concrete highway divider  low profile chunks of concrete | | cinderblocks, culverts, highway divider, concrete | CB, Culvert, HD, Concrete | | CB, Culvert, HD, Concrete | | mid-high | 8 | | 4 | | | 7.863801 | | 0.956997 | | Other / Mixed | |
| 220 | | 220 | | 4083.683 | | | 2 | | 22.796 | | 289.7826 | | 18.98983 | | Patch 85 | | 41.99004 | | 98.44135 | mixed patch w/ 1 6 in palet cinderblocks  2 8 in palet cinderblocks  2 10x10 box culverts  low profile chunks of concrete  low profile chunks of concrete | | cinderblocks, culverts, highway divider, concrete | CB, Culvert, HD, Concrete | | CB, Culvert, HD, Concrete | | mid-high | 8 | | 4 | | | 9.314755 | | 4.083683 | | Other / Mixed | |
| 227 | | 227 | | 22148.67 | | | 5 | | 23.15577 | | 163.9655 | | 18.95944 | | Patch 89 | | 74.98651 | | 344.2749 | mixed patch w/ 2 8 in palet cinderblocks  3 pieces concrete highway divider  5 round culverts  low profile chunks of concrete | | cinderblocks, culverts, highway divider, concrete | CB, Culvert, HD, Concrete | | CB, Culvert, HD, Concrete | | mid-high | 8 | | 4 | | | 11.00553 | | 22.14867 | | Other / Mixed | |
| 228 | | 228 | | 5434842 | | | 15 | | 23.26229 | | 118.9682 | | 17.15999 | | Patch 91 | | 64.30997 | | 234.7095 | mixed patch w/ 2 8 in palet cinderblocks  2 10x10 box culverts  3 pieces concrete highway divider  low profile chunks of concrete | | cinderblocks, culverts, highway divider, concrete | CB, Culvert, HD, Concrete | | CB, Culvert, HD, Concrete | | mid-high | 8 | | 4 | | | 16.50834 | | 5434.842 | | Other / Mixed | |
| 267 | | 267 | | 5241.854 | | | 5 | | 22.95098 | | 260.1985 | | 37.6655 | | Patch 86 | | 96.87672 | | 423.9392 | mixed patch w/ 1 6 in palet cinderblocks  2 8 in palet cinderblocks  2 10x10 box culverts  3 pieces concrete highway divider  low profile chunks of concrete  low profile chunks of concrete | | cinderblocks, culverts, highway divider, concrete | CB, Culvert, HD, Concrete | | CB, Culvert, HD, Concrete | | mid-high | 8 | | 4 | | | 9.564431 | | 5.241854 | | Other / Mixed | |
| 269 | | 269 | | 9669.928 | | | 3 | | 23.31956 | | 161.2687 | | 12.28169 | | Patch 89 | | 74.98651 | | 344.2749 | mixed patch w/ 2 8 in palet cinderblocks  3 pieces concrete highway divider  5 round culverts  low profile chunks of concrete | | cinderblocks, culverts, highway divider, concrete | CB, Culvert, HD, Concrete | | CB, Culvert, HD, Concrete | | mid-high | 8 | | 4 | | | 10.17678 | | 9.669928 | | Other / Mixed | |
| 273 | | 273 | | 11918.81 | | | 6 | | 23.0516 | | 131.5683 | | 19.48186 | | Patch 101 | | 91.16628 | | 452.2679 | mixed patch w/ 1 6 in palet cinderblocks  2 8 in palet cinderblocks  3 pieces concrete highway divider  5 round culverts  low profile chunks of concrete | | cinderblocks, culverts, highway divider, concrete | CB, Culvert, HD, Concrete | | CB, Culvert, HD, Concrete | | mid-high | 8 | | 4 | | | 10.38587 | | 11.91881 | | Other / Mixed | |
| 274 | | 274 | | 15099.08 | | | 6 | | 22.99689 | | 52.09887 | | 84.56326 | | Patch 101 | | 91.16628 | | 452.2679 | mixed patch w/ 1 6 in palet cinderblocks  2 8 in palet cinderblocks  3 pieces concrete highway divider  5 round culverts  low profile chunks of concrete | | cinderblocks, culverts, highway divider, concrete | CB, Culvert, HD, Concrete | | CB, Culvert, HD, Concrete | | mid-high | 8 | | 4 | | | 10.62239 | | 15.09908 | | Other / Mixed | |
| 275 | | 275 | | 3002.983 | | | 3 | | 22.99069 | | 61.22751 | | 79.8576 | | Patch 101 | | 91.16628 | | 452.2679 | mixed patch w/ 1 6 in palet cinderblocks  2 8 in palet cinderblocks  3 pieces concrete highway divider  5 round culverts  low profile chunks of concrete | | cinderblocks, culverts, highway divider, concrete | CB, Culvert, HD, Concrete | | CB, Culvert, HD, Concrete | | mid-high | 8 | | 4 | | | 9.007361 | | 3.002983 | | Other / Mixed | |
| 276 | | 276 | | 4206.115 | | | 2 | | 23.036 | | 42.59364 | | 96.58123 | | Patch 101 | | 91.16628 | | 452.2679 | mixed patch w/ 1 6 in palet cinderblocks  2 8 in palet cinderblocks  3 pieces concrete highway divider  5 round culverts  low profile chunks of concrete | | cinderblocks, culverts, highway divider, concrete | CB, Culvert, HD, Concrete | | CB, Culvert, HD, Concrete | | mid-high | 8 | | 4 | | | 9.344295 | | 4.206115 | | Other / Mixed | |
| 277 | | 277 | | 29231.4 | | | 4 | | 22.98703 | | 79.84274 | | 63.07888 | | Patch 98 | | 53.3389 | | 161.3216 | mixed patch w/ 1 6 in palet cinderblocks   2 8 in palet cinderblocks   2 10x10 box culverts  3 pieces concrete highway divider  low profile chunks of concrete | | cinderblocks, culverts, highway divider, concrete | CB, Culvert, HD, Concrete | | CB, Culvert, HD, Concrete | | mid-high | 8 | | 4 | | | 11.283 | | 29.2314 | | Other / Mixed | |
| 280 | | 280 | | 6973.579 | | | 3 | | 23.1601 | | 171.1408 | | 28.00784 | | Patch 95 | | 59.2387 | | 210.5578 | mixed patch w/ 1 6 in palet cinderblocks  2 8 in palet cinderblocks  2 10x10 box culverts  3 pieces concrete highway divider  low profile chunks of concrete | | cinderblocks, culverts, highway divider, concrete | CB, Culvert, HD, Concrete | | CB, Culvert, HD, Concrete | | mid-high | 8 | | 4 | | | 9.849884 | | 6.973579 | | Other / Mixed | |
| 283 | | 283 | | 29712.94 | | | 3 | | 23.36762 | | 29.24249 | | 126.8156 | | Patch 99 | | 83.97865 | | 405.0499 | mixed patch w/ 1 6 in palet cinderblocks  2 8 in palet cinderblocks  3 pieces concrete highway divider  5 round culverts  low profile chunks of concrete | | cinderblocks, culverts, highway divider, concrete | CB, Culvert, HD, Concrete | | CB, Culvert, HD, Concrete | | mid-high | 8 | | 4 | | | 11.29934 | | 29.71294 | | Other / Mixed | |
| 284 | | 284 | | 52918.99 | | | 2 | | 23.16021 | | 177.7039 | | 11.6407 | | Patch 97 | | 84.18344 | | 398.9363 | mixed patch w/ 2 8 in palet cinderblocks  3 pieces concrete highway divider  5 round culverts  low profile chunks of concrete  low profile chunks of concrete | | cinderblocks, culverts, highway divider, concrete | CB, Culvert, HD, Concrete | | CB, Culvert, HD, Concrete | | mid-high | 8 | | 4 | | | 11.87652 | | 52.91899 | | Other / Mixed | |
| 294 | | 294 | | 17324.91 | | | 4 | | 21.54873 | | 225.637 | | 35.12935 | | Patch 76 | | 83.97047 | | 419.7981 | mixed patch w/ 1 6 in palet cinderblocks  2 8 in palet cinderblocks  2 10x10 box culverts  3 pieces concrete highway divider  low profile chunks of concrete | | cinderblocks, culverts, highway divider, concrete | CB, Culvert, HD, Concrete | | CB, Culvert, HD, Concrete | | mid-high | 8 | | 4 | | | 10.7599 | | 17.32491 | | Other / Mixed | |
| 299 | | 299 | | 3163.059 | | | 3 | | 21.54234 | | 230.7718 | | 48.37414 | | Patch 77 | | 94.90258 | | 520.2692 | mixed patch w/ 1 6 in palet cinderblocks  2 8 in palet cinderblocks  2 10x10 box culverts  3 pieces concrete highway divider  low profile chunks of concrete | | cinderblocks, culverts, highway divider, concrete | CB, Culvert, HD, Concrete | | CB, Culvert, HD, Concrete | | mid-high | 8 | | 4 | | | 9.059295 | | 3.163059 | | Other / Mixed | |
| 328 | | 328 | | 4878.432 | | | 4 | | 20.73699 | | 569.7204 | | 39.10388 | | Patch 66 | | 100.4173 | | 600.443 | mixed patch w/ 1 6 in palet cinderblocks  2 8 in palet cinderblocks  2 10x10 box culverts  3 pieces concrete highway divider  low profile chunks of concrete | | cinderblocks, culverts, highway divider, concrete | CB, Culvert, HD, Concrete | | CB, Culvert, HD, Concrete | | mid-high | 8 | | 4 | | | 9.492579 | | 4.878432 | | Other / Mixed | |
| 334 | | 334 | | 447.4081 | | | 3 | | 20.6545 | | 671.444 | | 85.58802 | | Patch 67 | | 103.545 | | 770.5823 | mixed patch w/ 1 6 in palet cinderblocks  2 8 in palet cinderblocks  2 10x10 box culverts  3 pieces concrete highway divider  low profile chunks of concrete | | cinderblocks, culverts, highway divider, concrete | CB, Culvert, HD, Concrete | | CB, Culvert, HD, Concrete | | mid-high | 8 | | 4 | | | 7.103471 | | 0.447408 | | Other / Mixed | |
| 371 | | 371 | | 2906.386 | | | 2 | | 23.32114 | | 29.86329 | | 103.4034 | | Patch 91 | | 64.30997 | | 234.7095 | mixed patch w/ 2 8 in palet cinderblocks  2 10x10 box culverts  3 pieces concrete highway divider  low profile chunks of concrete | | cinderblocks, culverts, highway divider, concrete | CB, Culvert, HD, Concrete | | CB, Culvert, HD, Concrete | | mid-high | 8 | | 4 | | | 8.974666 | | 2.906386 | | Other / Mixed | |
| 376 | | 376 | | 1810.409 | | | 2 | | 20.86228 | | 813.8068 | | 36.784 | | Patch 69 | | 139.6161 | | 1025.023 | mixed patch w/ 1 6 in palet cinderblocks  2 8 in palet cinderblocks  2 10x10 box culverts  3 pieces concrete highway divider  low profile chunks of concrete | | cinderblocks, culverts, highway divider, concrete | CB, Culvert, HD, Concrete | | CB, Culvert, HD, Concrete | | mid-high | 8 | | 4 | | | 8.501308 | | 1.810409 | | Other / Mixed | |
| 417 | | -1 | | 1819.251 | | | 1 | | 21.598 | | 231.1546 | | 41.68251 | | Patch 77 | | 94.90258 | | 520.2692 | mixed patch w/ 1 6 in palet cinderblocks  2 8 in palet cinderblocks  2 10x10 box culverts  3 pieces concrete highway divider  low profile chunks of concrete | | cinderblocks, culverts, highway divider, concrete | CB, Culvert, HD, Concrete | | CB, Culvert, HD, Concrete | | mid-high | 8 | | 4 | | | 8.50618 | | 1.819251 | | Other / Mixed | |
| 418 | | -1 | | 5733.981 | | | 1 | | 21.58765 | | 235.7903 | | 40.03439 | | Patch 78 | | 103.6715 | | 576.7736 | mixed patch w/ 1 6 in palet cinderblocks  2 8 in palet cinderblocks  2 10x10 box culverts  3 pieces concrete highway divider  low profile chunks of concrete | | cinderblocks, culverts, highway divider, concrete | CB, Culvert, HD, Concrete | | CB, Culvert, HD, Concrete | | mid-high | 8 | | 4 | | | 9.654165 | | 5.733981 | | Other / Mixed | |
[truncated: 57,553 more chars]
