# Supplementary material for: Comparison of fish biomass and fish carbon content associated with reef sites at the Rio Grande Valley artificial reef in the Gulf of Mexico
Source: PLoS One. 2026 Jun 4;21(6):e0350204. doi: 10.1371/journal.pone.0350204 (PMC13235911; doi:10.1371/journal.pone.0350204)
Supplement: S5 Table — (DOCX) [file pone.0350204.s010.docx]

**S5 Table. Dataset used to for total biomass and fish carbon calculations for the entire RGV Reef area presented in Figure 8.**

| Figure 8a |  |  |  |  |
| --- | --- | --- | --- | --- |
| Structure type | Replicates in Reef | Average biomass | Total biomass (kg) |  |
| Big Pile | 1 | 2852.40 | 2852.40 |  |
| Boat | 7 | 769.31 | 5385.16 |  |
| CB | 150 | 7.23 | 1083.95 |  |
| Concrete | 49 | 6.54 | 320.26 |  |
| Low Profile | 20 | 1.73 | 34.69 |  |
| Other/Mixed | 67 | 62.32 | 4175.36 |  |
| Pyramid | 52 | 27.55 | 1432.54 |  |
| RR | 131 | 32.29 | 4230.55 |  |
|  |  |  | 19514.92 |  |
|  |  |  |  |  |
| Figure 8b | Biomass (kg) | Dry Weight (kg) | Carbon (kg) | Carbon (tons) |
| Structure Associated | 19514.92 | 5151.94 | 2251.40 | 2.25 |
| Between Structure | 4881.56 | 1288.73 | 563.18 | 0.56 |
|  | 24396.48 |  |  | 2.81 |
