# Supplementary material for: Comparison of fish biomass and fish carbon content associated with reef sites at the Rio Grande Valley artificial reef in the Gulf of Mexico
Source: PLoS One. 2026 Jun 4;21(6):e0350204. doi: 10.1371/journal.pone.0350204 (PMC13235911; doi:10.1371/journal.pone.0350204)
Supplement: S1 File — (PDF) [file pone.0350204.s011.pdf]

S1 File. ArcGIS Spatial Analysis Workflow for Fish Biomass Analysis

1. Input data
    - Fish detections (tabular XY data)
    - Reef structures (polygon layer)
    - Bathymetry raster (IDW interpolation)
    - Reef boundary (polygon)
    - Reef structure attributes (tabular data)

2. Fish point creation and data preparation

    Fish detections were converted from tabular form to spatial points

    - Tool: *XY Table to Point*
    - Output: fish point feature class

    All fish points were:

    - Merged into a single dataset
        - Tool: *Merge*
    - Filtered to include only observations within the reef boundary
        - Tool: *Select by Location* (within reef boundary)

3. Coordinate system standardization

    All spatial layers (fish points, reef structures, bathymetry, reef boundary) were projected into:

    - Tool**:** *Project*
    - Coordinate system: State Plane NAD83 Texas South Central

4. Reef structure attributes

    Reef structure attributes were joined to reef structure polygons

    - Tool: *Join Field* or *Add Join*

5. Fish clustering (DBSCAN)

    Fish detections were grouped into spatial clusters representing aggregations using density-based clustering:

    - Tool: *Density-Based Clustering (DBSCAN)*
    - Minimum features per cluster: 2
    - Search distance: 10 m

    Cluster IDs were assigned to fish points.

6. Cluster identification and attribute assignment

    Cluster IDs were spatially joined back to fish points:

- Tool: *Spatial Join*
- Join type: ONE_TO_ONE
- Match option: INTERSECT

Fish not assigned to clusters were retained with Cluster ID = -1

7. Depth extraction

   Depth values at each fish cluster were extracted from the bathymetry raster:
   - Tool: *Extract Values to Points*

8. Cluster-level summary statistics

   Summary statics for fish clusters were calculated to get total biomass, total fish count, and average depth

   - Tool: *Summary Statistics*
   - Metrics:
     - Sum fish biomass
     - Sum fish count
     - Mean depth

9. Spatial representation of fish clusters

   9.1 Clustered Fish

   Centroids representing each fish cluster were calculated:

   - Tool: *Mean Center*

   9.2 Structure Centroids

   Centroids were generated for reef structures:

   - Tool: *Feature to Point*
   - Option: INSIDE

   9.3 Non-clustered fish

   Fish not assigned to clusters (Cluster ID = -1) were retained as individual points and analyzed separately.

10. Distance calculation

    Distance from each fish group centroid (and individual fish) to the nearest structure:

    - Tool: *Near*

    Distance from each fish group centroid (and individual fish) to the reef boundary:

    - Tool: *Near* (boundary polygon as target)

11. Assignment of structure attributes to fish

Attributes from the nearest reef structure were assigned:

- Tool: *Join Field*
- Join key: NEAR_FID

Applied to fish cluster centroids and individual fish points (Cluster ID = -1)

12. Dataset export

All processed spatial outputs were exported as excel files and combined into a single dataset for statistical analysis.
