# Supplementary material for: Comparison of fish biomass and fish carbon content associated with reef sites at the Rio Grande Valley artificial reef in the Gulf of Mexico
Source: PLoS One. 2026 Jun 4;21(6):e0350204. doi: 10.1371/journal.pone.0350204 (PMC13235911; doi:10.1371/journal.pone.0350204)
Supplement: S2 File — (PDF) [file pone.0350204.s012.pdf]

S2 File. R Scripts for Statistical Analyses, Modelling, and Figure Generation.

```
#load packages
library(ggplot2)
library(Hmisc)
library(pscl)
library(car)
library(vegan)
library(mosaic)
library(lmtest)
library(rcompanion)
library(lsmeans)
library(multcomp)
library(multcompView)
library(boot)
library(plyr)
library(AER)
library(fitdistrplus)
library(mgcv)
library(visreg)
library(car)
library(gratia)
library(dplyr)
library(mgcv)
library(MuMIn)
library(vcd)
library(patchwork)
library(ggbreak)
library(itsadug)
```

```
# structure polygon center point data

df3 <- read.csv("/Users/awhite1/Grad School/Kline Lab/Thesis/Data Analysis/NEW site center point all fish spatial analysis.csv")

str(df3)



#Lab computer

setwd("C:/Users/pdf606/Desktop/R projects/Revised data")

df3 <- read.csv("C:/Users/pdf606/Desktop/R projects/Revised data/NEW site center point all fish spatial analysis.csv")



df3$Near_site <- as.factor(df3$Near_site)

df3$material_category <- as.factor(df3$material_category)

df3$material <- as.factor(df3$material)

df3$structure <- as.factor(df3$structure)

df3$material_category_number <- as.factor(df3$material_category_number)

df3$relief_category <- factor(df3$relief_category, levels = c("low", "low-mid", "mid", "mid-high", "high"))


#weight plot

p1c <- ggplot(df3, aes(x=Weight_g)) +

  geom_histogram(bins = 30, fill = "skyblue", color = "black") +

  geom_density(aes(y = ..count..), color = "red", linewidth = 1) +

  ggtitle("Fish Weight (g)")


#count plot

p2c <- ggplot(df3, aes(x=Count_fish)) +

  geom_histogram(bins = 30, fill = "orange", color = "black") +

  geom_density(aes(y = ..count..), color = "red", linewidth = 1) +

  ggtitle("Fish Counts")
```

```
#depth plot
p3c <- ggplot(df3, aes(x=Avg_depth_m)) +
  geom_histogram(bins = 30, fill = "lightgreen", color = "black") +
  geom_density(aes(y = ..count..), color = "red", linewidth = 1) +
  ggtitle("Avg Depth (m)")

#distance to nearest site plot
p4c <- ggplot(df3, aes(x=Dist_near_site_m)) +
  geom_histogram(bins = 30, fill = "plum", color = "black") +
  geom_density(aes(y = ..count..), color = "red", linewidth = 1) +
  ggtitle("Distance to Nearest Site")

#distance to reef edge
p5c <- ggplot(df3, aes(x=Dist_boundary_m)) +
  geom_histogram(bins = 30, fill = "gray", color = "black") +
  geom_density(aes(y = ..count..), color = "red", linewidth = 1) +
  ggtitle("Distance to Boundary")

#show
(p1c| p2c) / (p3c| p4c) / p5c
p1c
p2c
p3c
p4c
p5c



#material
p6c <- ggplot(df3, aes(x=material_category)) +
  geom_bar(fill = "steelblue") +
```

```
  theme_minimal() +
  ggtitle("Material Type")

#relief
p7c <- ggplot(df3, aes(x=relief_category)) +
  geom_bar(fill = "coral") +
  theme_minimal() +
  ggtitle("Relief Category")

#structure category
p8c <- ggplot(df3, aes(x=structure_category)) +
  geom_bar(fill = "mediumseagreen") +
  theme_minimal() +
  ggtitle("Structure Category")

#show
(p6c| p7c) / p8c
p6c
p7c
p8c

#show all
(p1c| p2c) / (p3c| p4c) / (p5c|p6c) / (p7c|p8c)


#fish count by material
ggplot(df3, aes(x = material_category, y = Count_fish)) +
  geom_boxplot(fill="gold") +
  theme_minimal() +
  ggtitle("Fish Count by Material")
```

```
#fish weight by material
ggplot(df3, aes(x = material_category, y = Weight_g)) +
  geom_boxplot(fill="darkblue") +
  theme_minimal() +
  ggtitle("Fish Weight by Material")

#fish count by relief category
ggplot(df3, aes(x = relief_category, y = Count_fish)) +
  geom_boxplot(fill="lightblue") +
  theme_minimal() +
  ggtitle("Fish Count by Relief")

#fish weight by relief category
ggplot(df3, aes(x = relief_category, y = Weight_g)) +
  geom_boxplot(fill="lightblue") +
  theme_minimal() +
  ggtitle("Fish Weight by Relief")




#check distribution
mean(df3$Count_fish)
var(df3$Count_fish)

mean(df3$Weight_g)
var(df3$Weight_g)

#variation > mean so data is overdispersed af
```

```
##log transformation

df3$log_weight <- log(df3$Weight_g) + 1

model2 <- lm(log_weight ~ Avg_depth_m + Dist_near_site_m + Dist_boundary_m +
             material_category + relief_category + site_age, data = df3)
cooksD <- cooks.distance(model2)
which(cooksD > 4 / nrow(df3))
plot(model2)
summary(model2)


##################### DREDGE ##################################################

table(df3$structure)
length(unique(df3$structure))   # number of unique categories
unique(df3$structure)           # list of categories

# Fit a global model
global_gam <- gam(log_weight ~
                s(Avg_depth_m, k = 20) +
                s(Dist_near_site_m, k = 20) +
                s(Dist_boundary_m, k = 20) +
                structure_area_m +
                structure +
                material_category +
                material_category_number +
                material +
                relief_category +
```

```
          site_age +
          number_materials,
         data = df3,
         method = "REML")

# Allow MuMIn to access the full model structure
options(na.action = "na.fail")

# Run dredge to test all subsets of predictors
model_set <- dredge(global_gam)

# View top models by AIC
head(model_set)

# Extract best model
best_gam <- get.models(model_set, subset = 1)[[1]]
summary(best_gam)

plot(best_gam, pages = 1, shade = TRUE, seWithMean = TRUE)
draw(best_gam)

# Basic diagnostics
gam.check(best_gam)

# Better diagnostic plots
appraise(best_gam) +
  theme_classic() +
  theme(
    panel.grid = element_blank(),   # remove all grid lines
    panel.background = element_blank(), # white background
```

```
    plot.background = element_blank()   # clean outer background
  )
theme_set(theme_classic(base_size = 14))
appraise(best_gam)
diag_plots <- appraise(best_gam)
ggsave("gam_diagnostics.png", diag_plots, width = 10, height = 8, dpi = 300)

# Plot residuals
par(mfrow = c(2, 2))
plot(best_gam, residuals = TRUE, pch = 16, cex = 0.5)

# Standard diagnostic plots
par(mfrow = c(2, 2))
plot(best_gam, all.terms = TRUE)



par(mfrow = c(1,1))
plot(best_gam, select = 3, shade = TRUE, seWithMean = TRUE)




  ## Prettier plots please

  # avg depth smooth term
  avg_depth_smooth <- draw(best_gam, select = "s(Avg_depth_m)") +
  theme_minimal(base_size = 14) +  # Increase base font size
  theme(
    axis.text = element_text(size = 12),
    axis.title = element_text(size = 14),
    plot.title = element_text(size = 16, face = "bold"),
```

```
    panel.grid.major = element_blank(),  # Remove major gridlines
    panel.grid.minor = element_blank(),  # Remove minor gridlines
    axis.line = element_line(color = "black"),  # Add black axis lines
    panel.border = element_blank())  # Ensure no border overrides axis lines

print(avg_depth_smooth)
# Save plot
ggsave("gam_response_avg_depth.png", avg_depth_smooth, width = 6, height = 4, dpi = 300)

# dist boundary smooth
dist_bound_smooth <- draw(best_gam, select = "s(Dist_boundary_m)") +
  theme_minimal(base_size = 14) +  # Increase base font size
  theme(
    axis.text = element_text(size = 12),
    axis.title = element_text(size = 14),
    plot.title = element_text(size = 16, face = "bold"),
    panel.grid.major = element_blank(),  # Remove major gridlines
    panel.grid.minor = element_blank(),  # Remove minor gridlines
    axis.line = element_line(color = "black"),  # Add black axis lines
    panel.border = element_blank())  # Ensure no border overrides axis lines

print(dist_bound_smooth)
# Save plot
ggsave("gam_response_dist_boundary.png", dist_bound_smooth, width = 6, height = 4, dpi = 300)

#dist near site smooth
dist_near_smooth <- draw(best_gam, select = "s(Dist_near_site_m)") +
  theme_minimal(base_size = 14) +  # Increase base font size
  theme(
    axis.text = element_text(size = 12),
```

```
    axis.title = element_text(size = 14),
    plot.title = element_text(size = 16, face = "bold"),
    panel.grid.major = element_blank(),  # Remove major gridlines
    panel.grid.minor = element_blank(),  # Remove minor gridlines
    axis.line = element_line(color = "black"),  # Add black axis lines
    panel.border = element_blank())  # Ensure no border overrides axis lines

print(dist_near_smooth)
# Save plot
ggsave("gam_response_dist_near_site.png", dist_near_smooth, width = 6, height = 4, dpi = 300)

#relief category response plot
relief_df <- data.frame(relief_category = levels(df3$relief_category))
relief_pred <- predict(best_gam, relief_df = relief_df, type = "terms", se.fit = TRUE)
relief_term <- "relief_category"
plot_relief_df <- data.frame(
  relief_category = df3$relief_category,
  fit = relief_pred$fit[, relief_term],
  se = relief_pred$se.fit[, relief_term]
)
#plot
relief_response <- ggplot(plot_relief_df, aes( x = relief_category, y = fit)) +
  geom_point(size = 3, color = "orangered") +
  geom_errorbar(aes(ymin = fit - se, ymax = fit + se), width = 0.2, color = "orangered") +
  labs (title = "Relief Category Response",
        y = "Partial Effect", x = "Relief Category") +
  theme(
    axis.text = element_text(size = 12),
    axis.title = element_text(size = 14),
    plot.title = element_text(size = 16, face = "bold"),
```

```
    panel.grid.major = element_blank(),  # Remove major gridlines
    panel.grid.minor = element_blank(),  # Remove minor gridlines
    axis.line = element_line(color = "black"),  # Add black axis lines
    panel.border = element_blank())  # Ensure no border overrides axis lines

print(relief_response)
ggsave("gam_response_relief.png", relief_response, width = 6, height = 4, dpi = 300)

# relief response trying again
relief_smooth <- draw(best_gam, select = "relief_category") +
  theme_minimal(base_size = 14) +  # Increase base font size
  theme(
    axis.text = element_text(size = 12),
    axis.title = element_text(size = 14),
    plot.title = element_text(size = 16, face = "bold"),
    panel.grid.major = element_blank(),  # Remove major gridlines
    panel.grid.minor = element_blank(),  # Remove minor gridlines
    axis.line = element_line(color = "black"),  # Add black axis lines
    panel.border = element_blank())  # Ensure no border overrides axis lines

print(relief_smooth)
# Save plot
ggsave("gam_response_relief_smooth.png", relief_smooth, width = 6, height = 4, dpi = 300)



## Prettier plots please


# avg depth smooth term
avg_depth_smooth <- draw(best_GAM2, select = "s(Avg_depth_m)") +
  theme_minimal(base_size = 14) +  # Increase base font size
```

```
  theme(
    axis.text = element_text(size = 12),
    axis.title = element_text(size = 14),
    plot.title = element_text(size = 16, face = "bold"),
    panel.grid.major = element_blank(),  # Remove major gridlines
    panel.grid.minor = element_blank(),  # Remove minor gridlines
    axis.line = element_line(color = "black"),  # Add black axis lines
    panel.border = element_blank())  # Ensure no border overrides axis lines

print(avg_depth_smooth)
# Save plot
ggsave("gam_response_avg_depth.png", avg_depth_smooth, width = 6, height = 4, dpi = 300)

# dist boundary smooth
dist_bound_smooth <- draw(best_GAM2, select = "s(Dist_boundary_m)") +
  theme_minimal(base_size = 14) +  # Increase base font size
  theme(
    axis.text = element_text(size = 12),
    axis.title = element_text(size = 14),
    plot.title = element_text(size = 16, face = "bold"),
    panel.grid.major = element_blank(),  # Remove major gridlines
    panel.grid.minor = element_blank(),  # Remove minor gridlines
    axis.line = element_line(color = "black"),  # Add black axis lines
    panel.border = element_blank())  # Ensure no border overrides axis lines

print(dist_bound_smooth)
# Save plot
ggsave("gam_response_dist_boundary.png", dist_bound_smooth, width = 6, height = 4, dpi = 300)

#dist near site smooth
```

```
dist_near_smooth <- draw(best_GAM2, select = "s(Dist_near_site_m)") +
  theme_minimal(base_size = 14) +  # Increase base font size
  theme(
    axis.text = element_text(size = 12),
    axis.title = element_text(size = 14),
    plot.title = element_text(size = 16, face = "bold"),
    panel.grid.major = element_blank(),  # Remove major gridlines
    panel.grid.minor = element_blank(),  # Remove minor gridlines
    axis.line = element_line(color = "black"),  # Add black axis lines
    panel.border = element_blank())  # Ensure no border overrides axis lines

print(dist_near_smooth)
# Save plot
ggsave("gam_response_dist_near_site.png", dist_near_smooth, width = 6, height = 4, dpi = 300)

#relief category response plot
relief_df <- data.frame(relief_category = levels(df3$relief_category))
relief_pred <- predict(best_GAM2, relief_df = relief_df, type = "terms", se.fit = TRUE)
relief_term <- "relief_category"
plot_relief_df <- data.frame(
  relief_category = df3$relief_category,
  fit = relief_pred$fit[, relief_term],
  se = relief_pred$se.fit[, relief_term]
)
#plot
relief_response <- ggplot(plot_relief_df, aes( x = relief_category, y = fit)) +
  geom_point(size = 3, color = "orangered") +
  geom_errorbar(aes(ymin = fit - se, ymax = fit + se), width = 0.2, color = "orangered") +
  labs (title = "Relief Category Response",
        y = "Partial Effect", x = "Relief Category") +
```

```
  theme(
    axis.text = element_text(size = 12),
    axis.title = element_text(size = 14),
    plot.title = element_text(size = 16, face = "bold"),
    panel.grid.major = element_blank(),  # Remove major gridlines
    panel.grid.minor = element_blank(),  # Remove minor gridlines
    axis.line = element_line(color = "black"),  # Add black axis lines
    panel.border = element_blank(), # Ensure no border overrides axis lines
    panel.background = element_blank()    # Remove grey background
  )

print(relief_response)
ggsave("gam_response_relief_colored.png", relief_response, width = 6, height = 4, dpi = 300)

# relief response trying again
relief_smooth <- draw(best_GAM2, select = "relief_category") +
  theme_minimal(base_size = 14) +  # Increase base font size
  theme(
    axis.text = element_text(size = 12),
    axis.title = element_text(size = 14),
    plot.title = element_text(size = 16, face = "bold"),
    panel.grid.major = element_blank(),  # Remove major gridlines
    panel.grid.minor = element_blank(),  # Remove minor gridlines
    axis.line = element_line(color = "black"),  # Add black axis lines
    panel.border = element_blank())  # Ensure no border overrides axis lines

print(relief_smooth)
# Save plot
ggsave("gam_response_relief_smooth.png", relief_smooth, width = 6, height = 4, dpi = 300)
```

```
###################### NEW WITH DIFF MATERIAL
GROUPS######################################################

# time to simplify the materials to reduce autocorrelation
df3$material2 <- with(df3, case_when(
  # Pyramids (includes octoreef or pyramid)
  grepl("pyramid|octoreef", material, ignore.case = TRUE) ~ "Pyramids",

  # RR Ties (but not if spools are also mentioned)
  grepl("rr tie", material, ignore.case = TRUE) & !grepl("spool", material, ignore.case = TRUE) ~ "RR Ties",

  # Spools mixed with other materials → Other / Mixed
  grepl("spool", material, ignore.case = TRUE) &
    grepl("rr tie|culvert|highway divider|cinderblock|concrete|water mill", material, ignore.case = TRUE) ~
"Other / Mixed",

  # Cinderblocks ONLY (no other materials mentioned)
  grepl("^cinderblocks$", material, ignore.case = TRUE) ~ "Cinderblocks",

  # Combinations with cinderblocks + concrete/water mill/highway divider → Other / Mixed
  grepl("cinderblock", material, ignore.case = TRUE) &
    grepl("water mill|concrete|highway divider", material, ignore.case = TRUE) ~ "Other / Mixed",

  # Water mill and concrete together → Other / Mixed
  grepl("water mill", material, ignore.case = TRUE) &
    grepl("concrete", material, ignore.case = TRUE) ~ "Other / Mixed",

  # Concrete ONLY or limestone (standalone) → Concrete
```

```
  grepl("^concrete$", material, ignore.case = TRUE) |
    grepl("limestone", material, ignore.case = TRUE) ~ "Concrete",

  # Low profile only → Low Profile
  grepl("^low profile$", material, ignore.case = TRUE) ~ "Low Profile",

  # Boats
  grepl("boat", material, ignore.case = TRUE) ~ "Boat",

  # Big pile
  grepl("big pile", material, ignore.case = TRUE) ~ "Big Pile",

  # Catch-all for culvert, highway divider, and other mixed combos
  grepl("culvert|highway divider", material, ignore.case = TRUE) ~ "Other / Mixed",

  # Everything else
  TRUE ~ "Other / Mixed"
))

df3$material2 <- as.factor(df3$material2)
table(df3$material2)
length(unique(df3$material2))   # number of unique categories
unique(df3$material2)           # list of categories
df3$material2 <- relevel(df3$material2, ref = "Concrete") # concrete as intercept

###### CHECHING FOR COLLINEARITY between relief and material

#contingency table
table(df3$relief_category, df3$material)
```

```
#chi squared (low p-value (< 0.05) suggests a statistical association between the two variables)
chisq.test(table(df3$relief_category, df3$material))

#cramers v (~0.1 weak, ~0.3 moderate, ~0.5 strong association)
assocstats(table(df3$relief_category, df3$material))

#multicollinearity model

# Dummy encode both if needed
df_dummy <- model.matrix(~ material + relief_category, data = df3)[, -1]  # drop intercept

# Combine with log_weight to create a new data frame
df_temp <- cbind(log_weight = df3$log_weight, as.data.frame(df_dummy))

# Fit a model and check VIF
library(car)
lm_test <- lm(log_weight ~ ., data = df_temp)
vif(lm_test)

# CHECHING FOR COLLINEARITY between relief and material

#contingency table
table(df3$relief_category, df3$material2)

#chi squared (low p-value (< 0.05) suggests a statistical association between the two variables)
chisq.test(table(df3$relief_category, df3$material2))

#cramers v (~0.1 weak, ~0.3 moderate, ~0.5 strong association)
assocstats(table(df3$relief_category, df3$material2))
```

```
#multicollinearity model

# Dummy encode both if needed
df_dummy2 <- model.matrix(~ material2 + relief_category, data = df3)[, -1]  # drop intercept

# Combine with log_weight to create a new data frame
df_temp2 <- cbind(log_weight = df3$log_weight, as.data.frame(df_dummy2))

# Fit a model and check VIF
library(car)
lm_test2 <- lm(log_weight ~ ., data = df_temp2)
vif(lm_test2)

# PCA

library(fastDummies)
library(dplyr)

df_pca <- df3[, !(names(df3) %in% c("structure", "material_category",
                                    "material", "Near_site", "structure_length_m",
                                    "material_category_number","CLUSTER_ID",
                                    "OBJECTID", "Weight_g", "Count_fish"))]
names(df_pca)
str(df_pca)

# Example: selecting relevant predictors (adjust as needed)
predictors <- df_pca %>%
  dplyr::select(Avg_depth_m, Dist_near_site_m, Dist_boundary_m,
         structure_area_m, site_age, number_materials)  # keep only numeric variables
```

```
# Optional: One-hot encode factors if needed
library(fastDummies)
predictors <- dummy_cols(df_pca, select_columns = c("material2", "relief_category"),
                         remove_selected_columns = TRUE) %>%
  dplyr::select(-log_weight)  # remove response



predictors_scaled <- scale(predictors)


pca <- prcomp(predictors_scaled, center = TRUE, scale. = TRUE)


summary(pca)
pca$rotation         # Loadings (how variables relate to PCs)
pca$x                # Scores (observations in PC space)


biplot(pca, scale = 0)


library(factoextra)


# Scree plot (variance explained)
fviz_eig(pca)


# PCA plot of individuals (colored by material, optional)
fviz_pca_ind(pca,
             geom.ind = "point",
             col.ind = df3$material2,  # or another grouping
             palette = "Dark2",
             addEllipses = TRUE,
             legend.title = "Material")
```

```
# PCA plot of variable loadings
fviz_pca_var(pca,
        col.var = "contrib", # color by contributions to PCs
        gradient.cols = c("#00AFBB", "#E7B800", "#FC4E07"),
        repel = TRUE)

library(corrplot)

corrplot(cor(predictors), method = "color", tl.cex = 0.8, addCoef.col = "black")

# PCA without material

df_pca2 <- df3[, !(names(df3) %in% c("structure", "material_category",
                    "material", "material2", "Near_site", "structure_length_m",
                    "material_category_number","CLUSTER_ID",
                    "OBJECTID", "Weight_g", "Count_fish", "log_weight"))]
names(df_pca2)
str(df_pca2)

# Example: selecting relevant predictors (adjust as needed)
predictors <- df_pca2 %>%
  dplyr::select(Avg_depth_m, Dist_near_site_m, Dist_boundary_m,
         structure_area_m, site_age)  # keep only numeric variables

# Optional: One-hot encode factors if needed
library(fastDummies)

relief_dummies <- dummy_cols(df_pca2["relief_category"],
              remove_selected_columns = TRUE) #dummy relief
```

```
predictors2 <- cbind(predictors, relief_dummies) #combine numeric and dummies

predictors2 <- predictors2 %>% dplyr::select(where(is.numeric)) #all numeric

predictors_scaled2 <- scale(predictors2)

pca2 <- prcomp(predictors_scaled2, center = TRUE, scale. = TRUE)

summary(pca2)
pca2$rotation         # Loadings (how variables relate to PCs)
pca2$x                # Scores (observations in PC space)

biplot(pca2, scale = 0)

library(factoextra)

# Scree plot (variance explained)
fviz_eig(pca2)

# PCA plot of individuals (colored by material, optional)
fviz_pca_ind(pca2,
        geom.ind = "point",
        col.ind = df3$material2,  #or another grouping
        palette = "Dark2",
        addEllipses = TRUE,
        legend.title = "Material")

# PCA plot of variable loadings
fviz_pca_var(pca2,
        col.var = "contrib", # color by contributions to PCs
```

```
            gradient.cols = c("#00AFBB", "#E7B800", "#FC4E07"),
            repel = TRUE,
            title = "PCA - Variable Loadings")

library(corrplot)

corrplot(cor(predictors), method = "color", tl.cex = 0.8, addCoef.col = "black")

##### Combining low and low-mid

df_pca3 <- df3[, !(names(df3) %in% c("structure", "material_category",
                                   "material", "material2", "Near_site", "structure_length_m",
                                   "material_category_number","CLUSTER_ID",
                                   "OBJECTID", "Weight_g", "Count_fish", "log_weight"))]

df_pca3$relief_category <- as.character(df_pca3$relief_category)
df_pca3$relief_category[df_pca3$relief_category %in% c("low", "low-mid")] <- "low"
df_pca3$relief_category <- factor(df_pca3$relief_category)

names(df_pca3)
str(df_pca3)

library(fastDummies)

relief_dummies2 <- model.matrix(~ relief_category - 1, data = df_pca3)

predictors3 <- df_pca3 %>%
  dplyr::select(Avg_depth_m, Dist_near_site_m, Dist_boundary_m,
               structure_area_m, site_age)
```

```r
predictors3 <- cbind(predictors3, relief_dummies2)

predictors3 <- predictors3[, !duplicated(colnames(predictors3))]

predictors_scaled3 <- scale(predictors3)
pca3 <- prcomp(predictors_scaled3, center = TRUE, scale. = TRUE)

summary(pca3)
pca3$rotation         # Loadings (how variables relate to PCs)
pca3$x                # Scores (observations in PC space)

biplot(pca3, scale = 0)

library(factoextra)

# Scree plot (variance explained)
fviz_eig(pca3)

# PCA plot of individuals (colored by material, optional)
fviz_pca_ind(pca3,
        geom.ind = "point",
        col.ind = df3$relief_category,  # or another grouping
        palette = "Dark2",
        addEllipses = TRUE,
        legend.title = "Relief Category")

# PCA plot of variable loadings
fviz_pca_var(pca3,
        col.var = "contrib", # color by contributions to PCs
```

```
          gradient.cols = c("#00AFBB", "#E7B800", "#FC4E07"),
          repel = TRUE,
          title = "PCA - Variable Loadings")

library(corrplot)

corrplot(cor(predictors3), method = "color", tl.cex = 0.8, addCoef.col = "black")

## culuative variance explained increased when material removed and relief grouped

########################################################################################
#####

# Fit a global model without material or structure
global_gam2 <- gam(log_weight ~
                s(Avg_depth_m, k = 20) +
                s(Dist_near_site_m, k = 20) +
                s(Dist_boundary_m, k = 20) +
                structure_area_m +
                relief_category +
                site_age +
                number_materials,
              data = df3,
              method = "REML")

# Allow MuMIn to access the full model structure
options(na.action = "na.fail")

# Run dredge to test all subsets of predictors
model_set2 <- dredge(global_gam2)
```

```
# View top models by AIC
head(model_set2)

# Extract best model
best_gam2 <- get.models(model_set2, subset = 1)[[1]]
summary(best_gam2)

plot(best_gam2, pages = 1, shade = TRUE, seWithMean = TRUE)
draw(best_gam2)

# Basic diagnostics
gam.check(best_gam2)

# Plot residuals
par(mfrow = c(2, 2))
plot(best_gam2, residuals = TRUE, pch = 16, cex = 0.5)

# Standard diagnostic plots
par(mfrow = c(2, 2))
plot(best_gam2, all.terms = TRUE)



par(mfrow = c(1,1))
plot(best_gam2, select = 3, shade = TRUE, seWithMean = TRUE)



## MODEL FROM DREDGE ##

best_gam2
```

```
## Prettier plots please

# avg depth smooth term
avg_depth_smooth_mat <- draw(best_gam2, select = "s(Avg_depth_m)") +
  theme_minimal(base_size = 14) +  # Increase base font size
  theme(
    axis.text = element_text(size = 12),
    axis.title = element_text(size = 14),
    plot.title = element_text(size = 16, face = "bold"),
    panel.grid.major = element_blank(),  # Remove major gridlines
    panel.grid.minor = element_blank(),  # Remove minor gridlines
    axis.line = element_line(color = "black"),  # Add black axis lines
    panel.border = element_blank())  # Ensure no border overrides axis lines

print(avg_depth_smooth_mat)
# Save plot
ggsave("gam_response_avg_depth_mat.png", avg_depth_smooth_mat, width = 6, height = 4, dpi = 300)



### even prettier
smooth_df <- smooth_estimates(best_gam2, smooth = "s(Avg_depth_m)")
depth_smooth_resp <- ggplot(smooth_df, aes(x = Avg_depth_m, y = .estimate)) +
  geom_ribbon(
    aes(ymin = .estimate - 2*.se, ymax = .estimate + 2*.se),
    fill = "lightblue", alpha = 0.4
  ) +
  geom_line(color = "blue", size = 1) +
  ggtitle("Avg Depth (m)") +
  xlab("Avg Depth (m)") +
  ylab("Partial effect") +
```

```
  theme_minimal() +
  theme(
    base_size = 14,
    axis.text = element_text(size = 12),
    axis.title = element_text(size = 14),
    plot.title = element_text(size = 16, face = "bold"),
    panel.grid.major = element_blank(),
    panel.grid.minor = element_blank(),
    axis.line = element_line(color = "black"),
    panel.border = element_blank()
  )

depth_smooth_resp
ggsave("gam_response_avg_depth.png", depth_smooth_resp, width = 6, height = 4, dpi = 300)

# dist boundary smooth
dist_bound_smooth_mat <- draw(best_gam2, select = "s(Dist_boundary_m)") +
  theme_minimal(base_size = 14) +  # Increase base font size
  theme(
    axis.text = element_text(size = 12),
    axis.title = element_text(size = 14),
    plot.title = element_text(size = 16, face = "bold"),
    panel.grid.major = element_blank(),  # Remove major gridlines
    panel.grid.minor = element_blank(),  # Remove minor gridlines
    axis.line = element_line(color = "black"),  # Add black axis lines
    panel.border = element_blank())  # Ensure no border overrides axis lines

print(dist_bound_smooth_mat)
# Save plot
ggsave("gam_response_dist_boundary_mat.png", dist_bound_smooth_mat, width = 6, height = 4, dpi =
300)
```

```
##prettiiierrr

# Extract smooth estimates

smooth_df_boundary <- smooth_estimates(gam_model, smooth = "s(Dist_boundary_m)")


dist_bound_resp <- ggplot(smooth_df_boundary,
              aes(x = Dist_boundary_m, y = .estimate)) +
  geom_ribbon(
    aes(ymin = .estimate - 2*.se, ymax = .estimate + 2*.se),
    fill = "lightgreen", alpha = 0.4
  ) +
  geom_line(color = "forestgreen", size = 1) +
  ggtitle("Distance to Boundary (m)") +
  xlab("Distance to Boundary (m)") +
  ylab("Partial effect") +
  theme_minimal() +
  theme(
    base_size = 14,
    axis.text = element_text(size = 12),
    axis.title = element_text(size = 14),
    plot.title = element_text(size = 16, face = "bold"),
    panel.grid.major = element_blank(),
    panel.grid.minor = element_blank(),
    axis.line = element_line(color = "black"),
    panel.border = element_blank()
  )

dist_bound_resp

ggsave("gam_response_dist_boundary.png", dist_bound_resp, width = 6, height = 4, dpi = 300)
```

```
#dist near site smooth

dist_near_smooth_mat <- draw(best_gam2, select = "s(Dist_near_site_m)") +

  theme_minimal(base_size = 14) +  # Increase base font size

  theme(

    axis.text = element_text(size = 12),

    axis.title = element_text(size = 14),

    plot.title = element_text(size = 16, face = "bold"),

    panel.grid.major = element_blank(),  # Remove major gridlines

    panel.grid.minor = element_blank(),  # Remove minor gridlines

    axis.line = element_line(color = "black"),  # Add black axis lines

    panel.border = element_blank())  # Ensure no border overrides axis lines


print(dist_near_smooth_mat)

# Save plot

ggsave("gam_response_dist_near_site_mat.png", dist_near_smooth_mat, width = 6, height = 4, dpi =
300)




## prettier

smooth_df_near <- smooth_estimates(gam_model, smooth = "s(Dist_near_site_m)")


dist_near_resp <- ggplot(smooth_df_near,

              aes(x = Dist_near_site_m, y = .estimate)) +

  geom_ribbon(

    aes(ymin = .estimate - 2*.se, ymax = .estimate + 2*.se),

    fill = "lightcoral", alpha = 0.4

  ) +

  geom_line(color = "red3", size = 1) +

  ggtitle("Distance to Nearest Structure (m)") +

  xlab("Distance to Nearest Structure (m)") +

  ylab("Partial effect") +
```

```
  theme_minimal() +
  theme(
    base_size = 14,
    axis.text = element_text(size = 12),
    axis.title = element_text(size = 14),
    plot.title = element_text(size = 16, face = "bold"),
    panel.grid.major = element_blank(),
    panel.grid.minor = element_blank(),
    axis.line = element_line(color = "black"),
    panel.border = element_blank()
  )

dist_near_resp

ggsave("gam_response_dist_near_site.png", dist_near_resp, width = 6, height = 4, dpi = 300)

#relief category response plot
relief_df <- data.frame(relief_category = levels(df3$relief_category))
relief_pred <- predict(best_gam2, relief_df = relief_df, type = "terms", se.fit = TRUE)
relief_term <- "relief_category"
plot_relief_df <- data.frame(
  relief_category = df3$relief_category,
  fit = relief_pred$fit[, relief_term],
  se = relief_pred$se.fit[, relief_term]
)
#plot
relief_response <- ggplot(plot_relief_df, aes(x = relief_category, y = fit)) +
  geom_point(size = 3, color = "orangered") +
  geom_errorbar(aes(ymin = fit - se, ymax = fit + se), width = 0.2, color = "orangered") +
  labs(title = "Relief Category Response",
```

```
     y = "Partial Effect", x = "Relief Category") +
  theme(
    axis.text = element_text(size = 12),
    axis.title = element_text(size = 14),
    plot.title = element_text(size = 16, face = "bold"),
    panel.grid.major = element_blank(),   # Remove major gridlines
    panel.grid.minor = element_blank(),   # Remove minor gridlines
    axis.line = element_line(color = "black"),  # Add black axis lines
    panel.border = element_blank(),       # Ensure no border overrides axis lines
    panel.background = element_blank()    # Remove grey background
  )

print(relief_response)
ggsave("gam_response_relief.png", relief_response, width = 6, height = 4, dpi = 300)


############################SUBSET##########################################

# Subset the data to only fish within 20m of a structure
df_within20 <- df3 %>%
  filter(Dist_near_site_m <= 20)



# dredge fish within 20m
# Fit a global model
global_gam20 <- gam(log_weight ~
                      s(Avg_depth_m, k = 20) +
                      s(Dist_near_site_m, k = 20) +
                      s(Dist_boundary_m, k = 20) +
                      structure_area_m +
```

```
                    relief_category +
                    site_age +
                    number_materials,
                  data = df_within20,
                  method = "REML")

# Allow MuMIn to access the full model structure
options(na.action = "na.fail")

# Run dredge to test all subsets of predictors
model_set20 <- dredge(global_gam20)

# View top models by AIC
head(model_set20)

# Extract best model
best_gam20 <- get.models(model_set20, subset = 1)[[1]]
summary(best_gam20)

plot(best_gam20, pages = 1, shade = TRUE, seWithMean = TRUE)
draw(best_gam20)

# Basic diagnostics
gam.check(best_gam20)
appraise(best_gam20)
diag_plots_best_gam20 <- appraise(best_gam20)
ggsave("gam_diagnostics_bestgam20.png", diag_plots_best_gam20, width = 10, height = 8, dpi = 300)

# avg depth smooth term
avg_depth_smooth_near20 <- draw(best_gam20, select = "s(Avg_depth_m)") +
```

```
  geom_ribbon(
    aes(ymin = .estimate - 2*.se, ymax = .estimate + 2*.se),
    fill = "lightblue", alpha = 0.4
  ) +
  geom_line(color = "blue", size = 1) +
  ggtitle("Avg Depth (m) (≤ 20 m from Structure)") +
  xlab("Avg Depth (m)") +
  ylab("Partial effect") +
  theme_minimal(base_size = 14) +  # Increase base font size
  theme(
    axis.text = element_text(size = 12),
    axis.title = element_text(size = 14),
    plot.title = element_text(size = 16, face = "bold"),
    panel.grid.major = element_blank(),  # Remove major gridlines
    panel.grid.minor = element_blank(),  # Remove minor gridlines
    axis.line = element_line(color = "black"),  # Add black axis lines
    panel.border = element_blank())  # Ensure no border overrides axis lines

print(avg_depth_smooth_near20)
# Save plot
ggsave("gam_response_avg_depth_near20_colored.png", avg_depth_smooth_near20, width = 6, height = 4, dpi = 300)

# Define desired category order
relief_levels <- c("low", "low-mid", "mid", "mid-high", "high")

# Create prediction dataset with correct order
newdat <- data.frame(
  relief_category = relief_levels,
  Avg_depth_m = mean(best_gam20$model$Avg_depth_m, na.rm = TRUE)
)
```

```
# Ensure the factor has the specified order
newdat$relief_category <- factor(newdat$relief_category, levels = relief_levels)

# Predict partial effects
pred <- predict(best_gam20, newdata = newdat, se.fit = TRUE)

# Combine predictions with newdat
newdat <- newdat %>%
  mutate(
    fit = pred$fit - mean(pred$fit),  # center around 0 to show partial effects
    se = pred$se.fit,
    lower = fit - 1.96 * se,
    upper = fit + 1.96 * se
  )

# Plot to match your full-model style
relief_near20 <- ggplot(newdat, aes(x = relief_category, y = fit)) +
  geom_point(size = 3, color = "orangered3") +
  geom_errorbar(aes(ymin = lower, ymax = upper), width = 0.1, color = "orangered") +
  theme_classic(base_size = 14) +
  labs(
    x = "Relief Category",
    y = "Partial Effect",
    title = "Relief Category Response (≤ 20 m from Structure)"
  ) +
  theme(
    plot.title = element_text(face = "bold", size = 16),
    axis.title = element_text(size = 14),
    axis.text = element_text(size = 12)
```

```
  )

# Display and save
print(relief_near20)
ggsave("gam_response_relief_near20_colored.png", relief_near20, width = 6, height = 4, dpi = 300)


####### Subset only fish farther than 20m from a structure
df_away20 <- df3 %>%
  filter(Dist_near_site_m > 20)


# dredge fish farther than 20m
# Fit a global model
global_gamaway20 <- gam(log_weight ~
                          s(Avg_depth_m, k = 20) +
                          s(Dist_near_site_m, k = 20) +
                          s(Dist_boundary_m, k = 20) +
                          structure_area_m +
                          relief_category +
                          site_age +
                          number_materials,
                        data = df_away20,
                        method = "REML")

# Allow MuMIn to access the full model structure
options(na.action = "na.fail")

# Run dredge to test all subsets of predictors
model_setaway20 <- dredge(global_gamaway20)
```

```
# View top models by AIC
head(model_setaway20)

# Extract best model
best_gamaway20 <- get.models(model_setaway20, subset = 1)[[1]]
summary(best_gamaway20)

plot(best_gamaway20, pages = 1, shade = TRUE, seWithMean = TRUE)
draw(best_gamaway20)

# Basic diagnostics
gam.check(best_gamaway20)

# avg depth smooth term
avg_depth_smooth_away20 <- draw(best_gamaway20, select = "s(Avg_depth_m)") +
  geom_ribbon(
    aes(ymin = .estimate - 2*.se, ymax = .estimate + 2*.se),
    fill = "lightblue", alpha = 0.4
  ) +
  geom_line(color = "blue", size = 1) +
  ggtitle("Avg Depth (m) (> 20 m from Structure)") +
  xlab("Avg Depth (m)") +
  ylab("Partial effect") +
  theme_minimal(base_size = 14) +  # Increase base font size
  theme(
    axis.text = element_text(size = 12),
    axis.title = element_text(size = 14),
    plot.title = element_text(size = 16, face = "bold"),
    panel.grid.major = element_blank(),  # Remove major gridlines
```

```
    panel.grid.minor = element_blank(),  # Remove minor gridlines
    axis.line = element_line(color = "black"),  # Add black axis lines
    panel.border = element_blank())  # Ensure no border overrides axis lines

print(avg_depth_smooth_away20)
# Save plot
ggsave("gam_response_avg_depth_away20_colored.png", avg_depth_smooth_away20, width = 6,
height = 4, dpi = 300)

# dist boundary smooth
dist_bound_smooth_away20 <- draw(best_gamaway20, select = "s(Dist_boundary_m)") +
  geom_ribbon(
    aes(ymin = .estimate - 2*.se, ymax = .estimate + 2*.se),
    fill = "lightgreen", alpha = 0.4
  ) +
  geom_line(color = "forestgreen", size = 1) +
  ggtitle("Distance to Boundary (m) (> 20 m from Structure)") +
  xlab("Distance to Boundary (m)") +
  ylab("Partial effect") +
  theme_minimal(base_size = 14) +  # Increase base font size
  theme(
    axis.text = element_text(size = 12),
    axis.title = element_text(size = 14),
    plot.title = element_text(size = 16, face = "bold"),
    panel.grid.major = element_blank(),  # Remove major gridlines
    panel.grid.minor = element_blank(),  # Remove minor gridlines
    axis.line = element_line(color = "black"),  # Add black axis lines
    panel.border = element_blank())  # Ensure no border overrides axis lines

print(dist_bound_smooth_away20)
# Save plot
```

```
ggsave("gam_response_dist_boundary_away20_colored.png", dist_bound_smooth_away20, width = 6,
height = 4, dpi = 300)


#dist near site smooth

dist_near_smooth_away20 <- draw(best_gamaway20, select = "s(Dist_near_site_m)") +
  geom_ribbon(
    aes(ymin = .estimate - 2*.se, ymax = .estimate + 2*.se),
    fill = "lightcoral", alpha = 0.4
  ) +
  geom_line(color = "red3", size = 1) +
  ggtitle("Distance to Nearest Structure (m)\n(> 20 m from Structure)") +
  xlab("Distance to Nearest Structure (m)") +
  ylab("Partial effect") +
  theme_minimal(base_size = 14) +  # Increase base font size
  theme(
    axis.text = element_text(size = 12),
    axis.title = element_text(size = 14),
    plot.title = element_text(size = 16, face = "bold"),
    panel.grid.major = element_blank(),  # Remove major gridlines
    panel.grid.minor = element_blank(),  # Remove minor gridlines
    axis.line = element_line(color = "black"),  # Add black axis lines
    panel.border = element_blank())  # Ensure no border overrides axis lines


print(dist_near_smooth_away20)

# Save plot

ggsave("gam_response_dist_near_site_away20_colored.png", dist_near_smooth_away20, width = 6,
height = 4, dpi = 300)


# Define desired category order

relief_levels <- c("low", "low-mid", "mid", "mid-high", "high")
```

```
# Create prediction dataset with correct order
newdat_away <- data.frame(
  relief_category = relief_levels,
  Avg_depth_m = mean(best_gamaway20$model$Avg_depth_m, na.rm = TRUE),
  Dist_boundary_m = mean(best_gamaway20$model$Dist_boundary_m, na.rm = TRUE),
  Dist_near_site_m = mean(best_gamaway20$model$Dist_near_site_m, na.rm = TRUE),
  structure_area_m = mean(best_gamaway20$model$structure_area_m, na.rm = TRUE)
)

# Ensure the factor has the specified order
newdat_away$relief_category <- factor(newdat_away$relief_category, levels = relief_levels)

# Predict partial effects
pred_away <- predict(best_gamaway20, newdata = newdat_away, se.fit = TRUE)

# Combine predictions with newdat
newdat_away <- newdat_away %>%
  mutate(
    fit = pred_away$fit - mean(pred_away$fit),  # center around 0 for partial effect
    se = pred_away$se.fit,
    lower = fit - 1.96 * se,
    upper = fit + 1.96 * se
  )

# Plot to match full model style
relief_away20 <- ggplot(newdat_away, aes(x = relief_category, y = fit)) +
  geom_point(size = 3, color = "orangered3") +
  geom_errorbar(aes(ymin = lower, ymax = upper), width = 0.1, color = "orangered") +
  theme_classic(base_size = 14) +
  labs(
```

```
    x = "Relief Category",
    y = "Partial Effect",
    title = "Relief Category Response (> 20 m from Structure)"
  ) +
  theme(
    plot.title = element_text(face = "bold", size = 16),
    axis.title = element_text(size = 14),
    axis.text = element_text(size = 12)
  )

# Display and save
print(relief_away20)
ggsave("gam_response_relief_away20_colored.png", relief_away20, width = 6, height = 4, dpi = 300)

# Create a sequence of structure area values across the observed range
newdat_area <- data.frame(
  structure_area_m = seq(
    from = min(best_gamaway20$model$structure_area_m, na.rm = TRUE),
    to = max(best_gamaway20$model$structure_area_m, na.rm = TRUE),
    length.out = 100
  ),
  relief_category = "low",  # reference category (or most common)
  Avg_depth_m = mean(best_gamaway20$model$Avg_depth_m, na.rm = TRUE),
  Dist_boundary_m = mean(best_gamaway20$model$Dist_boundary_m, na.rm = TRUE),
  Dist_near_site_m = mean(best_gamaway20$model$Dist_near_site_m, na.rm = TRUE)
)

# Get predictions and standard errors
pred_area <- predict(best_gamaway20, newdata = newdat_area, se.fit = TRUE)
```

```
# Combine results into data frame
newdat_area <- newdat_area %>%
  mutate(
    fit = pred_area$fit - mean(pred_area$fit),  # center to show partial effect
    se = pred_area$se.fit,
    lower = fit - 1.96 * se,
    upper = fit + 1.96 * se
  )

# Plot
area_response_away20 <- ggplot(newdat_area, aes(x = structure_area_m, y = fit)) +
  geom_line(linewidth = 1, color = "navy") +
  geom_ribbon(aes(ymin = lower, ymax = upper), alpha = 0.3, fill = "navyblue") +
  theme_classic(base_size = 14) +
  labs(
    x = "Structure Area (m²)",
    y = "Partial Effect",
    title = "Structure Area (> 20 m from Structure)"
  ) +
  theme(
    plot.title = element_text(face = "bold", size = 16),
    axis.title = element_text(size = 14),
    axis.text = element_text(size = 12)
  )

print(area_response_away20)
ggsave("gam_response_area_away20_colored.png", area_response_away20, width = 6, height = 4, dpi = 300)


######################### Prediction models!!!! ############################
```

```
# Predict effect of Avg_depth_m across models
depth_seq <- seq(min(df3$Avg_depth_m, na.rm = TRUE),
                 max(df3$Avg_depth_m, na.rm = TRUE),
                 length.out = 100)

# For categorical variables, pick a reference level or most common level
newdata_full <- data.frame(
  Avg_depth_m = depth_seq,
  Dist_boundary_m = mean(df3$Dist_boundary_m, na.rm = TRUE),
  Dist_near_site_m = mean(df3$Dist_near_site_m, na.rm = TRUE),
  structure_area_m = mean(df3$structure_area_m, na.rm = TRUE),
  material2 = "RR Ties",  # adjust to your actual factor levels
  relief_category = "low",
  site_age = mean(df3$site_age, na.rm = TRUE)
)

pred_full <- predict(best_gam2, newdata = newdata_full, se.fit = TRUE)
newdata_full$fit <- pred_full$fit
newdata_full$lower <- pred_full$fit - 1.96 * pred_full$se.fit
newdata_full$upper <- pred_full$fit + 1.96 * pred_full$se.fit



predict_full <- ggplot(newdata_full, aes(x = Avg_depth_m, y = fit)) +
  geom_line(color = "#2c7bb6", linewidth = 1.2) +
  geom_ribbon(aes(ymin = lower, ymax = upper), alpha = 0.2, fill = "#2c7bb6") +
  labs(title = "All Fish", x = "Average Depth (m)", y = "Predicted log(Biomass)") +
  theme_classic(base_size = 10) +
  theme(
    axis.text.x = element_text(angle = 45, hjust = 1),
```

```
    axis.line = element_line(color = "black"),  # Add black axis lines
    panel.border = element_blank(), # Ensure no border overrides axis lines
  )


predict_full



# Load required packages
library(mgcv)
library(ggplot2)
library(dplyr)
library(patchwork)  # for combining plots

# ============================================
# PREDICTION: Average_depth_m
# ============================================

depth_seq <- seq(min(df3$Avg_depth_m, na.rm = TRUE),
                 max(df3$Avg_depth_m, na.rm = TRUE),
                 length.out = 100)

# ------------------------------
# 1. All Fish (best_gam)
# ------------------------------
new_full <- data.frame(
  Avg_depth_m = depth_seq,
  Dist_boundary_m = mean(df3$Dist_boundary_m, na.rm = TRUE),
  Dist_near_site_m = mean(df3$Dist_near_site_m, na.rm = TRUE),
  structure_area_m = mean(df3$structure_area_m, na.rm = TRUE),
```

```
  relief_category = "low",  # adjust to your dataset
  material = "RR Ties",
  site_age = mean(df3$site_age, na.rm = TRUE)
)

pred_full <- predict(best_gam2, newdata = newdata_full, se.fit = TRUE)
newdata_full$fit <- pred_full$fit
newdata_full$lower <- pred_full$fit - 1.96 * pred_full$se.fit
newdata_full$upper <- pred_full$fit + 1.96 * pred_full$se.fit

p9 <- ggplot(new_full, aes(x = Avg_depth_m, y = fit)) +
  geom_line(color = "#1f77b4", size = 1.2) +
  geom_ribbon(aes(ymin = lower, ymax = upper), fill = "#1f77b4", alpha = 0.2) +
  labs(title = "All Fish", x = "Average Depth (m)", y = "Predicted log(Biomass)") +
  theme_classic(base_size = 14) +
  theme(
    axis.text.x = element_text(angle = 10, hjust = 1),
    panel.grid.major = element_blank(),  # Remove major gridlines
    panel.grid.minor = element_blank(),  # Remove minor gridlines
    axis.line = element_line(color = "black"),  # Add black axis lines
    panel.border = element_blank(), # Ensure no border overrides axis lines
  )

p9

# ------------------------------
# 2. Fish within 20 m (best_gam20)
# ------------------------------
new_20 <- data.frame(
  Avg_depth_m = depth_seq,
```

```
  Dist_boundary_m = mean(df_within20$Dist_boundary_m, na.rm = TRUE),
  Dist_near_site_m = mean(df_within20$Dist_near_site_m, na.rm = TRUE),
  relief_category = "low"  # adjust if needed
)

pred_20 <- predict(best_gam20, newdata = new_20, se.fit = TRUE)
new_20$fit <- pred_20$fit
new_20$lower <- pred_20$fit - 1.96 * pred_20$se.fit
new_20$upper <- pred_20$fit + 1.96 * pred_20$se.fit

p10 <- ggplot(new_20, aes(x = Avg_depth_m, y = fit)) +
  geom_line(color = "#2ca02c", size = 1.2) +
  geom_ribbon(aes(ymin = lower, ymax = upper), fill = "#2ca02c", alpha = 0.2) +
  labs(title = "Fish Within 20 m", x = "Average Depth (m)", y = "Predicted log(Biomass)") +
  theme_classic(base_size = 10) +
  theme(
    axis.text.x = element_text(angle = 45, hjust = 1),
    panel.grid.major = element_blank(),  # Remove major gridlines
    panel.grid.minor = element_blank(),  # Remove minor gridlines
    axis.line = element_line(color = "black"),  # Add black axis lines
    panel.border = element_blank(), # Ensure no border overrides axis lines
  )

p10

# ------------------------------
# 3. Fish > 20 m (best_gamaway20)
# ------------------------------
new_away <- data.frame(
  Avg_depth_m = depth_seq,
```

```
  Dist_boundary_m = mean(df_away20$Dist_boundary_m, na.rm = TRUE),
  Dist_near_site_m = mean(df_away20$Dist_near_site_m, na.rm = TRUE),
  structure_area_m = mean(df_away20$structure_area_m, na.rm = TRUE),
  relief_category = "low"
)

pred_away <- predict(best_gamaway20, newdata = new_away, se.fit = TRUE)
new_away$fit <- pred_away$fit
new_away$lower <- pred_away$fit - 1.96 * pred_away$se.fit
new_away$upper <- pred_away$fit + 1.96 * pred_away$se.fit

p11 <- ggplot(new_away, aes(x = Avg_depth_m, y = fit)) +
  geom_line(color = "#d62728", size = 1.2) +
  geom_ribbon(aes(ymin = lower, ymax = upper), fill = "#d62728", alpha = 0.2) +
  labs(title = "Fish > 20 m", x = "Average Depth (m)", y = "Predicted log(Biomass)") +
  theme_classic(base_size = 10) +
  theme(
    axis.text.x = element_text(angle = 45, hjust = 1),
    panel.grid.major = element_blank(),  # Remove major gridlines
    panel.grid.minor = element_blank(),  # Remove minor gridlines
    axis.line = element_line(color = "black"),  # Add black axis lines
    panel.border = element_blank(), # Ensure no border overrides axis lines
  )
p11

# ------------------------------
# Combine all plots
# ------------------------------
combined_plot <- predict_full + p10 + p11 +
  plot_layout(ncol = 1) +
```

```
  plot_annotation(title = "Predicted Fish Biomass Across Depth by Group",
                  theme = theme(plot.title = element_text(hjust = 0.5, size = 16)))

# View the plot
print(combined_plot)

# ------------------------------
# Save the figure for publication
# ------------------------------
ggsave("fish_biomass_predictions_depth_by_group.png", plot = combined_plot,
       width = 8, height = 10, dpi = 300)



# ============================================
# PREDICTION: Dist_near_site_m
# ============================================

# Set up a prediction range for Dist_near_site_m
dist_seq <- seq(min(df3$Dist_near_site_m, na.rm = TRUE),
                max(df3$Dist_near_site_m, na.rm = TRUE),
                length.out = 100)

## 1. All Fish (best_gam)
new_dist_all <- data.frame(
  Dist_near_site_m = dist_seq,
  Avg_depth_m = mean(df3$Avg_depth_m, na.rm = TRUE),
  Dist_boundary_m = mean(df3$Dist_boundary_m, na.rm = TRUE),
  structure_area_m = mean(df3$structure_area_m, na.rm = TRUE),
  relief_category = "low",
  material = "RR Ties",
```

```
  site_age = mean(df3$site_age, na.rm = TRUE)
)

pred_dist_all <- predict(best_gam2, newdata = new_dist_all, se.fit = TRUE)
new_dist_all$fit <- pred_dist_all$fit
new_dist_all$lower <- pred_dist_all$fit - 1.96 * pred_dist_all$se.fit
new_dist_all$upper <- pred_dist_all$fit + 1.96 * pred_dist_all$se.fit

p_dist_all <- ggplot(new_dist_all, aes(x = Dist_near_site_m, y = fit)) +
  geom_line(color = "#1f77b4", size = 1.2) +
  geom_ribbon(aes(ymin = lower, ymax = upper), fill = "#1f77b4", alpha = 0.2) +
  labs(title = "All Fish", x = "Distance to Structure (m)", y = "Predicted log(Biomass)") +
  theme_classic(base_size = 10) +
  theme(
    axis.text.x = element_text(angle = 45, hjust = 1),
    panel.grid.major = element_blank(),  # Remove major gridlines
    panel.grid.minor = element_blank(),  # Remove minor gridlines
    axis.line = element_line(color = "black"),  # Add black axis lines
    panel.border = element_blank(), # Ensure no border overrides axis lines
  )

p_dist_all

## 2. Fish within 20 m (best_gam20)
dist_seq20 <- seq(min(df_within20$Dist_near_site_m, na.rm = TRUE),
                  max(df_within20$Dist_near_site_m, na.rm = TRUE), length.out = 100)

new_dist_20 <- data.frame(
  Dist_near_site_m = dist_seq20,
  Avg_depth_m = mean(df_within20$Avg_depth_m, na.rm = TRUE),
```

```
  Dist_boundary_m = mean(df_within20$Dist_boundary_m, na.rm = TRUE),
  relief_category = "low"
)

pred_dist_20 <- predict(best_gam20, newdata = new_dist_20, se.fit = TRUE)
new_dist_20$fit <- pred_dist_20$fit
new_dist_20$lower <- pred_dist_20$fit - 1.96 * pred_dist_20$se.fit
new_dist_20$upper <- pred_dist_20$fit + 1.96 * pred_dist_20$se.fit

p_dist_20 <- ggplot(new_dist_20, aes(x = Dist_near_site_m, y = fit)) +
  geom_line(color = "#2ca02c", size = 1.2) +
  geom_ribbon(aes(ymin = lower, ymax = upper), fill = "#2ca02c", alpha = 0.2) +
  labs(title = "Fish Within 20 m", x = "Distance to Structure (m)", y = "Predicted log(Biomass)") +
  theme_classic(base_size = 10) +
  theme(
    axis.text.x = element_text(angle = 45, hjust = 1),
    panel.grid.major = element_blank(),  # Remove major gridlines
    panel.grid.minor = element_blank(),  # Remove minor gridlines
    axis.line = element_line(color = "black"),  # Add black axis lines
    panel.border = element_blank(), # Ensure no border overrides axis lines
  )

p_dist_20

## 3. Fish > 20 m (best_gamaway20)
dist_seq_away <- seq(min(df_away20$Dist_near_site_m, na.rm = TRUE),
                     max(df_away20$Dist_near_site_m, na.rm = TRUE), length.out = 100)

new_dist_away <- data.frame(
  Dist_near_site_m = dist_seq_away,
```

```
  Avg_depth_m = mean(df_away20$Avg_depth_m, na.rm = TRUE),
  Dist_boundary_m = mean(df_away20$Dist_boundary_m, na.rm = TRUE),
  structure_area_m = mean(df_away20$structure_area_m, na.rm = TRUE),
  relief_category = "low"
)

pred_dist_away <- predict(best_gamaway20, newdata = new_dist_away, se.fit = TRUE)
new_dist_away$fit <- pred_dist_away$fit
new_dist_away$lower <- pred_dist_away$fit - 1.96 * pred_dist_away$se.fit
new_dist_away$upper <- pred_dist_away$fit + 1.96 * pred_dist_away$se.fit

p_dist_away <- ggplot(new_dist_away, aes(x = Dist_near_site_m, y = fit)) +
  geom_line(color = "#d62728", size = 1.2) +
  geom_ribbon(aes(ymin = lower, ymax = upper), fill = "#d62728", alpha = 0.2) +
  labs(title = "Fish > 20 m", x = "Distance to Structure (m)", y = "Predicted log(Biomass)") +
  theme_classic(base_size = 10) +
  theme(
    axis.text.x = element_text(angle = 45, hjust = 1),
    panel.grid.major = element_blank(),  # Remove major gridlines
    panel.grid.minor = element_blank(),  # Remove minor gridlines
    axis.line = element_line(color = "black"),  # Add black axis lines
    panel.border = element_blank(), # Ensure no border overrides axis lines
  )

p_dist_away

# Combine Distance Plots
combined_dist <- p_dist_all + p_dist_20 + p_dist_away +
  plot_layout(ncol = 1) +
  plot_annotation(title = "Predicted Fish Biomass by Distance to Structure",
```

```
                  theme = theme(plot.title = element_text(hjust = 0.5, size = 16)))


# View the plot
print(combined_dist)

ggsave("predicted_biomass_by_distance.png", combined_dist, width = 8, height = 10, dpi = 300)

# ============================================
# PREDICTION: Dist_boundary_m (best_gam2)
# ============================================

# Create a sequence across the range of Dist_boundary_m
boundary_seq <- seq(min(df3$Dist_boundary_m, na.rm = TRUE),
                    max(df3$Dist_boundary_m, na.rm = TRUE),
                    length.out = 100)

## 1. All Fish (best_gam2)
new_boundary_all <- data.frame(
  Dist_boundary_m = boundary_seq,
  Dist_near_site_m = mean(df3$Dist_near_site_m, na.rm = TRUE),
  Avg_depth_m = mean(df3$Avg_depth_m, na.rm = TRUE),
  structure_area_m = mean(df3$structure_area_m, na.rm = TRUE),
  relief_category = "low",
  material = "RR Ties",
  site_age = mean(df3$site_age, na.rm = TRUE)
)

pred_boundary_all <- predict(best_gam2, newdata = new_boundary_all, se.fit = TRUE)
new_boundary_all$fit <- pred_boundary_all$fit
```

```r
new_boundary_all$lower <- pred_boundary_all$fit - 1.96 * pred_boundary_all$se.fit
new_boundary_all$upper <- pred_boundary_all$fit + 1.96 * pred_boundary_all$se.fit

p_boundary_all <- ggplot(new_boundary_all, aes(x = Dist_boundary_m, y = fit)) +
  geom_line(color = "#1f77b4", size = 1.2) +
  geom_ribbon(aes(ymin = lower, ymax = upper), fill = "#1f77b4", alpha = 0.2) +
  labs(title = "All Fish", x = "Distance to Boundary (m)", y = "Predicted log(Biomass)") +
  theme_classic(base_size = 10) +
  theme(
    axis.text.x = element_text(angle = 45, hjust = 1),
    panel.grid = element_blank(),
    axis.line = element_line(color = "black"),
    panel.border = element_blank()
  )

## 2. Fish within 20 m (best_gam20)
boundary_seq20 <- seq(min(df_within20$Dist_boundary_m, na.rm = TRUE),
                      max(df_within20$Dist_boundary_m, na.rm = TRUE), length.out = 100)

new_boundary_20 <- data.frame(
  Dist_boundary_m = boundary_seq20,
  Dist_near_site_m = mean(df_within20$Dist_near_site_m, na.rm = TRUE),
  Avg_depth_m = mean(df_within20$Avg_depth_m, na.rm = TRUE),
  relief_category = "low"
)

pred_boundary_20 <- predict(best_gam20, newdata = new_boundary_20, se.fit = TRUE)
new_boundary_20$fit <- pred_boundary_20$fit
new_boundary_20$lower <- pred_boundary_20$fit - 1.96 * pred_boundary_20$se.fit
new_boundary_20$upper <- pred_boundary_20$fit + 1.96 * pred_boundary_20$se.fit
```

```
p_boundary_20 <- ggplot(new_boundary_20, aes(x = Dist_boundary_m, y = fit)) +
  geom_line(color = "#2ca02c", size = 1.2) +
  geom_ribbon(aes(ymin = lower, ymax = upper), fill = "#2ca02c", alpha = 0.2) +
  labs(title = "Fish Within 20 m", x = "Distance to Boundary (m)", y = "Predicted log(Biomass)") +
  theme_classic(base_size = 10) +
  theme(
    axis.text.x = element_text(angle = 45, hjust = 1),
    panel.grid = element_blank(),
    axis.line = element_line(color = "black"),
    panel.border = element_blank()
  )

## 3. Fish > 20 m (best_gamaway20)
boundary_seq_away <- seq(min(df_away20$Dist_boundary_m, na.rm = TRUE),
                         max(df_away20$Dist_boundary_m, na.rm = TRUE), length.out = 100)

new_boundary_away <- data.frame(
  Dist_boundary_m = boundary_seq_away,
  Dist_near_site_m = mean(df_away20$Dist_near_site_m, na.rm = TRUE),
  Avg_depth_m = mean(df_away20$Avg_depth_m, na.rm = TRUE),
  structure_area_m = mean(df_away20$structure_area_m, na.rm = TRUE),
  relief_category = "low"
)

pred_boundary_away <- predict(best_gamaway20, newdata = new_boundary_away, se.fit = TRUE)
new_boundary_away$fit <- pred_boundary_away$fit
new_boundary_away$lower <- pred_boundary_away$fit - 1.96 * pred_boundary_away$se.fit
new_boundary_away$upper <- pred_boundary_away$fit + 1.96 * pred_boundary_away$se.fit
```

```
p_boundary_away <- ggplot(new_boundary_away, aes(x = Dist_boundary_m, y = fit)) +
  geom_line(color = "#d62728", size = 1.2) +
  geom_ribbon(aes(ymin = lower, ymax = upper), fill = "#d62728", alpha = 0.2) +
  labs(title = "Fish > 20 m", x = "Distance to Boundary (m)", y = "Predicted log(Biomass)") +
  theme_classic(base_size = 10) +
  theme(
    axis.text.x = element_text(angle = 45, hjust = 1),
    panel.grid = element_blank(),
    axis.line = element_line(color = "black"),
    panel.border = element_blank()
  )

# Combine Boundary Plots
combined_boundary <- p_boundary_all + p_boundary_20 + p_boundary_away +
  plot_layout(ncol = 1) +
  plot_annotation(title = "Predicted Fish Biomass by Distance to Boundary",
                  theme = theme(plot.title = element_text(hjust = 0.5, size = 16)))

# Save and view
print(combined_boundary)
ggsave("predicted_biomass_by_boundary.png", combined_boundary, width = 8, height = 10, dpi = 300)

# ==============================================
# PREDICTION: structure_area_m (best_gam2)
# ==============================================

area_seq <- seq(min(df3$structure_area_m, na.rm = TRUE),
                max(df3$structure_area_m, na.rm = TRUE),
                length.out = 100)
```

```
## 1. All Fish (best_gam2)
new_area_all <- data.frame(
  structure_area_m = area_seq,
  Dist_near_site_m = mean(df3$Dist_near_site_m, na.rm = TRUE),
  Dist_boundary_m = mean(df3$Dist_boundary_m, na.rm = TRUE),
  Avg_depth_m = mean(df3$Avg_depth_m, na.rm = TRUE),
  relief_category = "low",
  material = "RR Ties",
  site_age = mean(df3$site_age, na.rm = TRUE)
)

pred_area_all <- predict(best_gam2, newdata = new_area_all, se.fit = TRUE)
new_area_all$fit <- pred_area_all$fit
new_area_all$lower <- pred_area_all$fit - 1.96 * pred_area_all$se.fit
new_area_all$upper <- pred_area_all$fit + 1.96 * pred_area_all$se.fit

p_area_all <- ggplot(new_area_all, aes(x = structure_area_m, y = fit)) +
  geom_line(color = "#1f77b4", size = 1.2) +
  geom_ribbon(aes(ymin = lower, ymax = upper), fill = "#1f77b4", alpha = 0.2) +
  labs(title = "All Fish", x = "Structure Area (m²)", y = "Predicted log(Biomass)") +
  theme_classic(base_size = 10) +
  theme(
    axis.text.x = element_text(angle = 45, hjust = 1),
    panel.grid = element_blank(),
    axis.line = element_line(color = "black"),
    panel.border = element_blank()
  )

## 2. Fish within 20 m (best_gam20)
area_seq20 <- seq(min(df_within20$structure_area_m, na.rm = TRUE),
```

```r
                  max(df_within20$structure_area_m, na.rm = TRUE), length.out = 100)

new_area_20 <- data.frame(
  structure_area_m = area_seq20,
  Dist_near_site_m = mean(df_within20$Dist_near_site_m, na.rm = TRUE),
  Dist_boundary_m = mean(df_within20$Dist_boundary_m, na.rm = TRUE),
  Avg_depth_m = mean(df_within20$Avg_depth_m, na.rm = TRUE),
  relief_category = "low"
)

pred_area_20 <- predict(best_gam20, newdata = new_area_20, se.fit = TRUE)
new_area_20$fit <- pred_area_20$fit
new_area_20$lower <- pred_area_20$fit - 1.96 * pred_area_20$se.fit
new_area_20$upper <- pred_area_20$fit + 1.96 * pred_area_20$se.fit

p_area_20 <- ggplot(new_area_20, aes(x = structure_area_m, y = fit)) +
  geom_line(color = "#2ca02c", size = 1.2) +
  geom_ribbon(aes(ymin = lower, ymax = upper), fill = "#2ca02c", alpha = 0.2) +
  labs(title = "Fish Within 20 m", x = "Structure Area (m²)", y = "Predicted log(Biomass)") +
  theme_classic(base_size = 10) +
  theme(
    axis.text.x = element_text(angle = 45, hjust = 1),
    panel.grid = element_blank(),
    axis.line = element_line(color = "black"),
    panel.border = element_blank()
  )

## 3. Fish > 20 m (best_gamaway20)
area_seq_away <- seq(min(df_away20$structure_area_m, na.rm = TRUE),
                     max(df_away20$structure_area_m, na.rm = TRUE), length.out = 100)
```

```
new_area_away <- data.frame(
  structure_area_m = area_seq_away,
  Dist_near_site_m = mean(df_away20$Dist_near_site_m, na.rm = TRUE),
  Dist_boundary_m = mean(df_away20$Dist_boundary_m, na.rm = TRUE),
  Avg_depth_m = mean(df_away20$Avg_depth_m, na.rm = TRUE),
  relief_category = "low"
)

pred_area_away <- predict(best_gamaway20, newdata = new_area_away, se.fit = TRUE)
new_area_away$fit <- pred_area_away$fit
new_area_away$lower <- pred_area_away$fit - 1.96 * pred_area_away$se.fit
new_area_away$upper <- pred_area_away$fit + 1.96 * pred_area_away$se.fit

p_area_away <- ggplot(new_area_away, aes(x = structure_area_m, y = fit)) +
  geom_line(color = "#d62728", size = 1.2) +
  geom_ribbon(aes(ymin = lower, ymax = upper), fill = "#d62728", alpha = 0.2) +
  labs(title = "Fish > 20 m", x = "Structure Area (m²)", y = "Predicted log(Biomass)") +
  theme_classic(base_size = 10) +
  theme(
    axis.text.x = element_text(angle = 45, hjust = 1),
    panel.grid = element_blank(),
    axis.line = element_line(color = "black"),
    panel.border = element_blank()
  )

print(p_area_away)
ggsave("predicted_biomass_by_structure_area_awayonly.png", p_area_away, width = 10, height = 8, dpi
= 300)

# Combine Structure Area Plots
```

```
combined_area <- p_area_all + p_area_20 + p_area_away +
  plot_layout(ncol = 1) +
  plot_annotation(title = "Predicted Fish Biomass by Structure Area",
                  theme = theme(plot.title = element_text(hjust = 0.5, size = 16)))

# Save and view
print(combined_area)
ggsave("predicted_biomass_by_structure_area.png", combined_area, width = 8, height = 10, dpi = 300)



# ============================================
# PREDICTION: Relief Category
# ============================================

relief_levels <- levels(factor(df3$relief_category))

#changing order of cateogies
desired_order <- c("low", "low-mid", "mid", "mid-high", "high")
df3$relief_category <- factor(df3$relief_category, levels = desired_order)
df_within20$relief_category <- factor(df_within20$relief_category, levels = desired_order)
df_away20$relief_category <- factor(df_away20$relief_category, levels = desired_order)





# 1. All Fish
new_relief_all <- expand.grid(
  relief_category = relief_levels,
  Avg_depth_m = mean(df3$Avg_depth_m, na.rm = TRUE),
  Dist_near_site_m = mean(df3$Dist_near_site_m, na.rm = TRUE),
```

```
  Dist_boundary_m = mean(df3$Dist_boundary_m, na.rm = TRUE),
  structure_area_m = mean(df3$structure_area_m, na.rm = TRUE),
  material = "Concrete",
  site_age = mean(df3$site_age, na.rm = TRUE)
)

pred_relief_all <- predict(best_gam2, newdata = new_relief_all, se.fit = TRUE)
new_relief_all$fit <- pred_relief_all$fit
new_relief_all$lower <- pred_relief_all$fit - 1.96 * pred_relief_all$se.fit
new_relief_all$upper <- pred_relief_all$fit + 1.96 * pred_relief_all$se.fit
new_relief_all$relief_category <- factor(new_relief_all$relief_category, levels = desired_order)

p_relief_all <- ggplot(new_relief_all, aes(x = relief_category, y = fit)) +
  geom_point(size = 3, color = "#1f77b4") +
  geom_errorbar(aes(ymin = lower, ymax = upper), width = 0.2, color = "#1f77b4") +
  labs(title = "All Fish", x = "Relief category", y = "Predicted log(Biomass)") +
  theme_classic(base_size = 10) +
  theme(
    axis.text.x = element_text(angle = 45, hjust = 1),
    panel.grid.major = element_blank(),  # Remove major gridlines
    panel.grid.minor = element_blank(),  # Remove minor gridlines
    axis.line = element_line(color = "black"),  # Add black axis lines
    panel.border = element_blank(), # Ensure no border overrides axis lines
  )

p_relief_all

# 2. Within 20 m
new_relief_20 <- expand.grid(
  relief_category = relief_levels,
```

```
  Avg_depth_m = mean(df_within20$Avg_depth_m, na.rm = TRUE),
  Dist_boundary_m = mean(df_within20$Dist_boundary_m, na.rm = TRUE),
  Dist_near_site_m = mean(df_within20$Dist_near_site_m, na.rm = TRUE)
)

pred_relief_20 <- predict(best_gam20, newdata = new_relief_20, se.fit = TRUE)
new_relief_20$fit <- pred_relief_20$fit
new_relief_20$lower <- pred_relief_20$fit - 1.96 * pred_relief_20$se.fit
new_relief_20$upper <- pred_relief_20$fit + 1.96 * pred_relief_20$se.fit
new_relief_20$relief_category <- factor(new_relief_20$relief_category, levels = desired_order)


p_relief_20 <- ggplot(new_relief_20, aes(x = relief_category, y = fit)) +
  geom_point(size = 3, color = "#2ca02c") +
  geom_errorbar(aes(ymin = lower, ymax = upper), width = 0.2, color = "#2ca02c") +
  labs(title = "Fish Within 20 m", x = "Relief category", y = "Predicted log(Biomass)") +
  theme_classic(base_size = 10) +
  theme(
    axis.text.x = element_text(angle = 45, hjust = 1),
    panel.grid.major = element_blank(),  # Remove major gridlines
    panel.grid.minor = element_blank(),  # Remove minor gridlines
    axis.line = element_line(color = "black"),  # Add black axis lines
    panel.border = element_blank(), # Ensure no border overrides axis lines
  )

p_relief_20

# 3. Away >20 m
new_relief_away <- expand.grid(
  relief_category = relief_levels,
```

```
  Avg_depth_m = mean(df_away20$Avg_depth_m, na.rm = TRUE),
  Dist_near_site_m = mean(df_away20$Dist_near_site_m, na.rm = TRUE),
  Dist_boundary_m = mean(df_away20$Dist_boundary_m, na.rm = TRUE),
  structure_area_m = mean(df_away20$structure_area_m, na.rm = TRUE)
)

pred_relief_away <- predict(best_gamaway20, newdata = new_relief_away, se.fit = TRUE)
new_relief_away$fit <- pred_relief_away$fit
new_relief_away$lower <- pred_relief_away$fit - 1.96 * pred_relief_away$se.fit
new_relief_away$upper <- pred_relief_away$fit + 1.96 * pred_relief_away$se.fit
new_relief_away$relief_category <- factor(new_relief_away$relief_category, levels = desired_order)

p_relief_away <- ggplot(new_relief_away, aes(x = relief_category, y = fit)) +
  geom_point(size = 3, color = "#d62728") +
  geom_errorbar(aes(ymin = lower, ymax = upper), width = 0.2, color = "#d62728") +
  labs(title = "Fish > 20 m", x = "Relief category", y = "Predicted log(Biomass)") +
  theme_classic(base_size = 10) +
  theme(
    axis.text.x = element_text(angle = 45, hjust = 1),
    panel.grid.major = element_blank(),  # Remove major gridlines
    panel.grid.minor = element_blank(),  # Remove minor gridlines
    axis.line = element_line(color = "black"),  # Add black axis lines
    panel.border = element_blank(), # Ensure no border overrides axis lines
  )
p_relief_away



# Combine Relief Category Plots
combined_relief <- p_relief_all + p_relief_20 + p_relief_away +
```

```
  plot_layout(ncol = 1) +
  plot_annotation(
                theme = theme(plot.title = element_text(hjust = 0.5, size = 16)))

# View the plot
print(combined_relief)

ggsave("predicted_biomass_by_relief_category.png", combined_relief, width = 8, height = 10, dpi = 300)



###################### ANOVA ###################################

anovadata_noBP <- read.csv("C:/Users/pdf606/Desktop/R projects/max day per site anova data.csv")
anovadata <- read.csv("C:/Users/pdf606/Desktop/R projects/max day per site anova data w big pile.csv")

mean(anovadata_noBP$Weight_kg)
var(anovadata_noBP$Weight_kg)

#variation > mean so data is overdispersed af

##log transformation
anovadata_noBP$log_weight <- log(anovadata_noBP$Weight_kg) + 1
anovadata$log_weight <- log(anovadata$Weight_kg) + 1

##sqrt transformation
anovadata_noBP$sqrt_weight <- sqrt(anovadata_noBP$Weight_kg)



anova_model <- aov(Weight_kg ~ Material.new, data = anovadata_noBP)
summary(anova_model)
```

```
# Get residuals
residuals_anova <- residuals(anova_model)

# Histogram of residuals
hist(residuals_anova, main = "Residuals Histogram", xlab = "Residuals")

# Q-Q plot
qqnorm(residuals_anova)
qqline(residuals_anova, col = "red")

# Shapiro-Wilk test (p > 0.05 means residuals are not significantly different from normal)
shapiro.test(residuals_anova)

# Tukey’s Honest Significant Difference
TukeyHSD(anova_model)

#visualize
boxplot(Weight_kg ~ Material.new, data = anovadata_noBP,
        main = "Fish Biomass by Structure Type",
        xlab = "Structure Category",
        ylab = "Fish Biomass")



## ANOVA LOG TRANSFORMED
anova_log <- aov(log_weight ~ Material.new, data = anovadata_noBP)
summary(anova_log)

# Get residuals
residuals_anova_log <- residuals(anova_log)
```

```
# Histogram of residuals
hist(residuals_anova_log, main = "Residuals Histogram", xlab = "Residuals")

# Q-Q plot
qqnorm(residuals_anova_log)
qqline(residuals_anova_log, col = "red")

# Shapiro-Wilk test (p > 0.05 means residuals are not significantly different from normal)
shapiro.test(residuals_anova_log)

# Bartlett Test
bartlett.test(log_weight ~ Material.new, data = anovadata_noBP)

# Tukey's Honest Significant Difference
TukeyHSD(anova_log)

# Residuals vs fitted values plot
plot(anova_log, which = 1)   # Look for equal spread across groups



# Simple anova relief
anova_relief <- aov(log_weight ~ Relief, data = anovadata)
summary(anova_relief)
#assumptions
car::leveneTest(Weight_kg ~ Material.new, data = anovadata) #homogeneity of variance
plot(anova_relief, which = 2)  # Q-Q plot
shapiro.test(residuals(anova_relief))  # Shapiro-Wilk test
TukeyHSD(anova_relief)
```

```
## ANOVA SQRT TRANSFORMED
anova_sqrt <- aov(sqrt_weight ~ Material.new, data = anovadata_noBP)
summary(anova_sqrt)

# Get residuals
residuals_anova_sqrt <- residuals(anova_sqrt)

# Histogram of residuals
hist(residuals_anova_sqrt, main = "Residuals Histogram", xlab = "Residuals")

# Q-Q plot
qqnorm(residuals_anova_sqrt)
qqline(residuals_anova_sqrt, col = "red")

# Shapiro-Wilk test (p > 0.05 means residuals are not significantly different from normal)
shapiro.test(residuals_anova_sqrt)

# Bartlett Test
bartlett.test(log_weight ~ Material.new, data = anovadata_noBP)

# Tukey's Honest Significant Difference
TukeyHSD(anova_sqrt)

# Residuals vs fitted values plot
plot(anova_sqrt, which = 1)   # Look for equal spread across groups

### Kruskal Wallis
kruskal.test(Weight_kg ~ Material.new, data = anovadata_noBP)

# Install if needed
```

```
install.packages("FSA")
library(FSA)

# Dunn's test with Benjamini-Hochberg correction
dunnTest(Weight_kg ~ Material.new, data = anovadata_noBP, method = "bh")

boxplot(Weight_kg ~ Material.new, data = anovadata_noBP,
        xlab = "Material Type", ylab = "Fish Biomass",
        main = "Kruskal-Wallis: Fish Biomass by Structure Type")

write.csv(df3, "df3_export.csv", row.names = FALSE)
write.csv(df_away20, "df_away20_export_NEW.csv", row.names = FALSE)
write.csv(df_within20, "df_within20_export_NEW.csv", row.names = FALSE)
```
